# Magic cluster sizes of cationic and anionic sodium chloride clusters explained by statistical modeling of the complete phase space†

**DOI:** 10.1039/d4cp00357h

**Published:** 2024-03-11

**Authors:** Jessica C. Hartmann, Sarah J. Madlener, Christian van der Linde, Milan Ončák, Martin K. Beyer

**Affiliations:** a Universität Innsbruck, Institut für Ionenphysik und Angewandte Physik Technikerstraße 25 6020 Innsbruck Austria milan.oncak@uibk.ac.at martin.beyer@uibk.ac.at

## Abstract

As one of the main components of sea salt aerosols, sodium chloride is involved in numerous atmospheric processes. Gas-phase clusters are ideal models to study fundamental physical and chemical properties of sodium chloride, which are significantly affected by the cluster size. Of particular interest are magic cluster sizes, which exhibit high intensities in mass spectra. In order to understand the origin of these magic cluster sizes, quantum chemical calculations at the CCSD(T)//DFT level are performed, yielding structures and binding energies of neutral (NaCl)_*x*_, anionic (NaCl)_*x*_Cl^−^ and cationic (NaCl)_*x*_Na^+^ clusters up to *x* = 8. Our calculations show that the clusters can easily isomerize, enabling dissociation into the lowest-energy isomers of the fragments. Energetics can explain the special stability of (NaCl)_4_Cl^−^, but (NaCl)_4_Na^+^ actually offers low-lying dissociation channels, despite being a magic cluster size. Collision-induced dissociation experiments reveal that the loss of neutral clusters (NaCl)_*x*_, *x* = 2, 4, is in most cases more favorable than the loss of NaCl or the atomic ion, *i.e.* sodium chloride clusters actually fragment *via* the cleavage of the entire cluster, not by evaporating small cluster building blocks. This is rationalized by the calculated high stability of even-numbered neutral clusters (NaCl)_*x*_, especially *x* = 2, 4. Analysis of the density of states and rate constants calculated with a modified Rice–Ramsperger–Kassel–Marcus (RRKM) equation called AWATAR – considering all energetically accessible isomers of reactants and fragments – shows that entropic effects are responsible for the magic cluster character of (NaCl)_4_Na^+^. In particular, low-lying vibrational modes provide a high density of states of the near-planar cluster. Together with the small contribution of an atomic ion to the sum of states in a loose transition state for dissociation, this leads to a very small unimolecular rate constant for dissociation into (NaCl)_4_ and Na^+^, which is the lowest energy fragmentation pathway. Thus, entropic effects may override energetics for certain magic cluster sizes.

## Abstract 2

Sodium chloride clusters show rich structural diversity. Magic cluster sizes like the (NaCl)_4_Na^+^ cluster are not always energetically favoured. Their high abundance in mass spectra is explained by their kinetic stability against dissociation.

## Graphical abstract

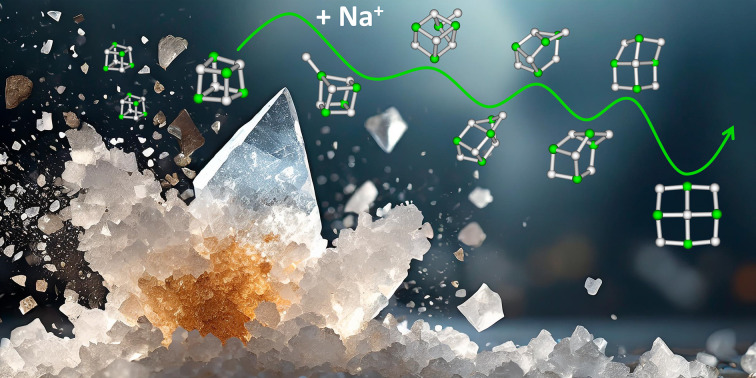

## Introduction

As one of the most important aerosols in Earth's atmosphere, sea salt aerosols significantly affect the climate.^1–6^ Sodium chloride – as a main component of sea salt aerosols^7,8^ – is involved in many atmospheric processes such as cloud formation,^9^ photochemical reactions^10,11^ as well as reactions with atmospheric trace gases.^12–16^ Gas-phase clusters are ideal models to study fundamental physical and chemical properties of pure and doped salts.^17–19^ Mass spectrometric studies of alkali halide clusters date back to the 1970s, utilizing secondary ion mass spectrometry (SIMS) and gas aggregation followed by electron ionization (EI), as comprehensively reviewed by Martin.^20^ Electrospray ionization has become the dominant way of preparing positively as well as negatively charged salt clusters.^21–24^

Very intriguing aspects are associated with multiply charged salt clusters. Kappes and co-workers studied the collision induced dissociation (CID) of doubly charged Na_*n*_Cl_*n*+2_^2−^ clusters and found fission into stoichiometric fragments Na_*m*_Cl_*m*+1_^−^ as the lowest energy dissociation pathways.^25^ Using high salt concentrations, they observed metastable salt cluster dianions as small as *n* = 6. Jarrold and co-workers investigated anionic clusters Na_*n*_Cl_*n*+1_^−^, *n* ≤ 49, by ion mobility, which allowed them to assign cuboid structures that resemble fcc nanocrystals.^26^ For *n* > 30, several families of isomers were resolved at room temperature. However, at ambient or slightly elevated temperatures, high-energy structures can be annealed, *i.e.* transformed to more stable ones, with activation energies of less than 0.6 eV.^27^

Kebarle and co-workers reported peaks of unusually high intensities in the electrospray ionization mass spectra of Na_*n*+1_Cl_*n*_^+^ – commonly referred to as “magic numbers” in cluster science – at *n* = 4, 13, and 22,^28^ matching those identified earlier by Martin^20^ and Twu *et al.*^29^ Jarrold and co-workers have shown that magic number clusters are associated with the highest melting temperatures, 850 K for Na_23_Cl_22_^+^ and Na_38_Cl_37_^+^, while defect-rich clusters melt at up to 100 K lower tempertures.^30^ Konermann and co-workers recently studied the dissociation behavior of sodium chloride clusters by CID and molecular dynamics (MD) simulations.^21^ In these simulations, the clusters predominantly fragment by loss of Na_*m*_Cl_*m*_ units with *m* = 1–3, which is preceded by significant structural rearrangements. Experimentally, the doubly-charged magic number cluster Na_28_Cl_26_^2+^ dissociates into two Na_14_Cl_13_^+^ products, which requires a major rearrangement.^21^

Cluster structure and energetics – in particular the origin of magic numbers – has been addressed in a series of computational works on neutral and ionic sodium chloride clusters, employing Born–Meyer potential based methods,^31,32^ the inversion pair potential^33^ and the *ab initio* perturbed-ion model.^34^ Quantum chemical calculations have been performed using self-consistent-field (SCF), second-order Møller–Plesset perturbation theory (MP2) and CCSD(T) methods.^35–37^ Assuming the loss of single NaCl units or the atomic ion (Cl^−^ or Na^+^) to be the main fragmentation pathway of sodium chloride cluster ions, it was found that the binding energies of single NaCl units or the respective atomic ion to the cluster is highest for the magic cluster sizes, which would explain the magic numbers.^20,33,37^ However, possible structural changes before fragmentation were not considered, which means that the cluster fragment is usually an isomer with relatively high energy. Since MD simulations by the Konermann group^21^ as well as ion mobility studies by the Jarrold group^27^ showed that isomerization is possible and efficient well below the dissociation threshold, low-lying isomers should be accessible already before fragmentation takes place. Thus, the actual activation energies for loss of single NaCl units or an atomic ion may be significantly smaller than the previously reported values,^20,33,37^ if one assumes that the cluster fragments into the lowest-energy isomers of the products. Moreover, experimental CID studies indicate the preferential loss of larger neutral units (NaCl)_*n*_ from sodium chloride clusters (NaCl)_*x*_Na^+^.^21,38^ It is thus not at all obvious how sodium chloride clusters fragment, and whether the formation of magic number clusters really is driven by their energetic stability alone.

In this study, we use state of the art quantum chemical calculations to obtain structure and energetics of a variety of isomers for neutral, cationic and anionic sodium chloride clusters, (NaCl)_*x*_, (NaCl)_*x*_Na^+^ and (NaCl)_*x*_Cl^−^, respectively, with *x* = 1–8. For the ionic magic cluster size *x* = 4, we obtain the minimum energy paths for loss of NaCl or the atomic ion and show that the lowest-energy isomers of the products are easily accessible. We further analyze the energetics for all stoichiometric dissociation pathways and show that loss of larger neutral fragments, in particular (NaCl)_2_ and (NaCl)_4_, is often energetically preferred. Sustained off-resonance irradiation collision-induced dissociation (SORI CID) experiments with (NaCl)_*x*_Na^+^ and (NaCl)_*x*_Cl^−^, *x* = 3–8, confirm that dissociation indeed occurs *via* multiple pathways, losing (NaCl)_*n*_, but the energetically preferred pathway is not always dominant in the experiment. We therefore employ a statistical analysis, calculating the density of states as a function of internal energy to understand the population of isomers in the SORI-CID experiment. We also introduce a modification of Rice–Ramsperger–Kassel–Marcus (RRKM) theory to describe the kinetics of cluster fragmentation. We find that in some cases, the preferred fragmentation pathway and thus, the origin of magic cluster sizes, is actually driven by entropy rather than internal energy.

## Experimental and theoretical details

### Experiments

The experiments were performed on a Bruker Apex Qe 9.4 T Fourier-Transform Ion Cyclotron Resonance Mass Spectrometer (FT-ICR MS) equipped with an Apollo electrospray ionization/MALDI Dual Source II and a Nanobay Console.^39^ Sodium chloride cluster ions are produced *via* electrospray ionization of a 10 mM NaCl solution, using a 1 : 1 mixture of CH_3_OH : H_2_O as solvent. While hydrated salt clusters can be obtained by spraying from 100% aqueous solution and lowering the drying gas temperature,^40^ we did not observe any solvent-containing clusters under the conditions used here, see representative mass spectra in ESI,† Fig. S23 and S24. Isotopically enriched Na^35^Cl powder (99% ^35^Cl, Sigma Aldrich) is used to improve the signal-to-noise ratio of the cluster ion signal. HPLC grade solvents are purchased from Carl Roth. The ions are transferred into the ICR cell of the FT-ICR MS, where the cluster size of interest is isolated. Sustained off-resonance irradiation (SORI)^41^ collision induced dissociation (CID) experiments are performed^42–44^ using argon (99.999%) as collision gas at constant pressures between 0.8 × 10^−8^ mbar and 1.0 × 10^−8^ mbar. The parameters for the SORI CID experiment were set to a pulse length of 1.0 s and a frequency offset of 500.0 Hz. The collision energy is controlled *via* the parameter “SORI Power” in % values *via* the software Compass apexControl 3.0.0 of the instrument. An increase of the SORI Power leads to a non-linear shift of the average ion kinetic energy to higher values, and to a significant broadening of the kinetic energy distribution. Thus, this experiment yields qualitative fragmentation curves for a rising collision energy, but does not provide quantitative information.

### Quantum chemistry

For the best compromise of reliability and computational tractability, single-point CCSD(T)/aug-cc-pVTZ calculations are performed with DFT-optimized structures. To reduce the risk of possible DFT artefacts, two different functionals are employed for structure optimization, B3LYP including the D3 version of Grimme's dispersion correction,^45^ further denoted as B3LYP-D3, and the ωB97XD functional that already includes a dispersion correction per default. Both functionals are used together with the aug-cc-pVDZ basis set for structure optimization. In the following, single-point CCSD(T)/aug-cc-pVTZ calculations performed with B3LYP-D3/aug-cc-pVDZ or ωB97XD/aug-cc-pVDZ optimized structures are denoted as CCSD(T)/TZ//B3LYP-D3 and CCSD(T)/TZ//ωB97XD, respectively; for larger clusters and transition states, single-point CCSD/aug-cc-pVDZ calculations are used, denoted as CCSD/DZ. All reported energies are zero-point corrected. For benchmarking, we provide a broad comparison of various methods in the ESI,† showing differences within 0.05 eV upon change in the computational method and/or basis set. All quantum chemical calculations are performed using the Gaussian software package.^46^

### Density of states and RRKM calculations

To aid the interpretation of the fragmentation curves from SORI CID experiments, we calculate the density of states of individual isomers, as well as unimolecular dissociation rate constants *k*^+/−^_*x*,*n*_(*E*) for the dissociation of cluster size *x* by loss of (NaCl)_*n*_ as a function of internal energy *E* according to eqn (1).1
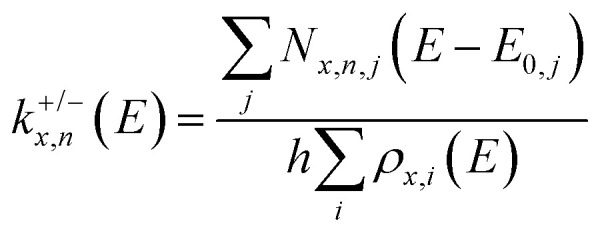
Here, we use a modification of Rice–Ramsperger–Kassel–Marcus (RRKM) theory,^47–49^ where we take into account all energetically accessible isomers *i* of cluster size *x* found in our quantum chemical calculations as well as all energetically accessible products *j*. We call this modification AWATAR for All Wells And Transition states Are Relevant. The denominator of eqn (1) contains Planck's constant *h* times the rovibrational density of states (DOS) *ρ*_*x*,*i*_(*E*) as function of internal energy *E*, summed over all isomers *i* of cluster size *x*. The sum of states (SOS) *N*_*x,n,j*_(*E* − *E*_0,*j*_) in the numerator of eqn (1) is calculated using a loose transition state (TS) model for the loss of neutral clusters (NaCl)_*n*_, which has previously been found to yield superior results over a tight TS model for unimolecular dissociation of ionic complexes.^48,50,51^ In this loose TS model, all rotational and vibrational degrees of freedom of the two fragments contribute to the sum of states. The sum runs over all combinations *j* of neutral and charged fragments that are accessible at energy *E* > *E*_0*,j*_, where *E*_0*,j*_ is the sum of the zero-point corrected energies of the two fragments. Chiral structures are considered with double degeneracy in DOS and SOS calculations.

Traditional RRKM theory is limited to the density of states of one isomer and the sum of states of one transition state representation. In the modified AWATAR version, we obtain a better representation of the actual phase space. The idea behind eqn (1) is that with an internal energy *E* above the dissociation threshold, the cluster explores all local minima, with rapid interconversions, before it dissociates. Likewise, while the cluster is dissociating, the emerging fragments explore all energetically accessible isomers. eqn (1) is thus a rigorous application of the key idea of RRKM theory to a cluster with multiple energetically accessible reactant and fragment isomers.

The DOS and AWATAR calculations are performed with the program developed for master equation modeling,^47^ which employs the Beyer–Swinehart algorithm^52^ as described by Gilbert and Smith.^53^ Single-point CCSD(T)/TZ//B3LYP-D3 and CCSD(T)/TZ//ωB97XD energies as well as unscaled B3LYP-D3/aug-cc-pVDZ and ωB97XD/aug-cc-pVDZ vibrational frequencies are used as input for the program, respectively. Benchmarking with other theory levels is included in the ESI.†

## Results and discussion

### Optimized structures

**Fig. 1:**
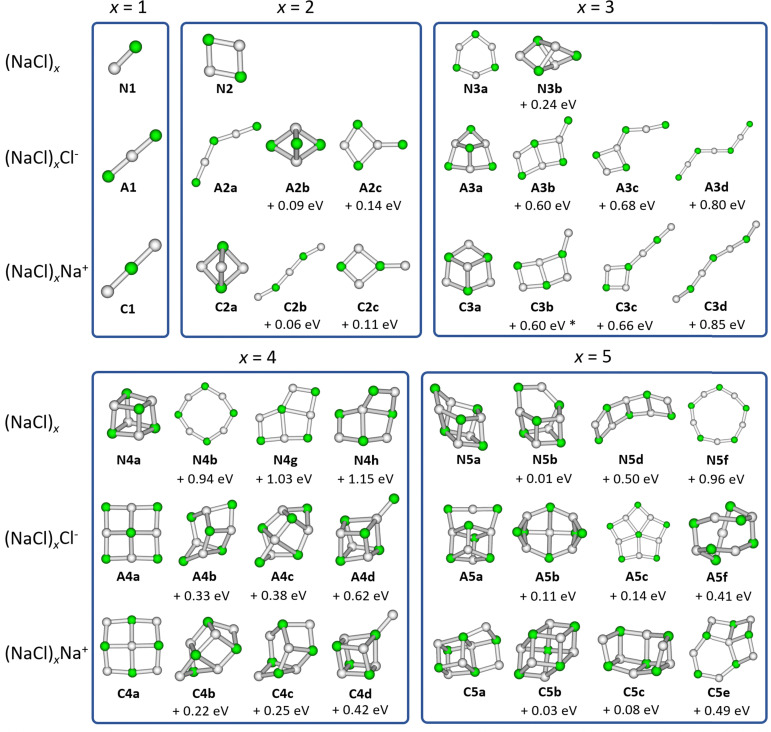
Selected low-lying isomers of the neutral sodium chloride clusters (NaCl)_*x*_, the cations (NaCl)_*x*_Na^+^ as well as the anions (NaCl)_*x*_Cl^−^ for cluster sizes *x* = 1–5. Relative energies compared to the lowest energy isomer are calculated at the CCSD(T)/TZ//B3LYP-D3 level of theory. Energies marked with * are obtained at the CCSD(T)/TZ//ωB97XD level due to the presence of an imaginary frequency when using the B3LYP-D3 functional.

**Fig. 2:**
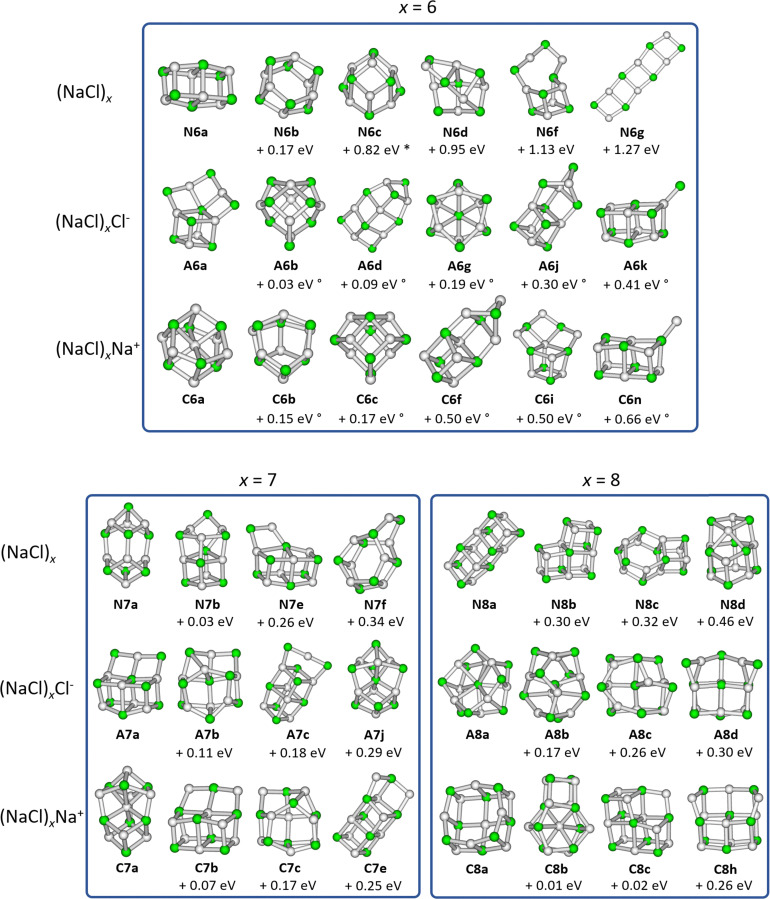
Selected low-lying isomers of the neutral sodium chloride clusters (NaCl)_*x*_, the cations (NaCl)_*x*_Na^+^ as well as the anions (NaCl)_*x*_Cl^−^ for cluster sizes *x* = 6–8. Relative energies compared to the lowest energy isomer are calculated at the CCSD/DZ//B3LYP-D3 level of theory. Energies marked with * are obtained at the CCSD(T)/TZ//ωB97XD level due to the presence of an imaginary frequency when using the B3LYP-D3 functional. Energies marked with ° were calculated at the CCSD(T)/TZ//B3LYP-D3 level of theory.

We consider isomers of the neutral sodium chloride clusters (NaCl)_*x*_, the cations (NaCl)_*x*_Na^+^ as well as the anions (NaCl)_*x*_Cl^−^ for *x* = 1–8. The most relevant structures, including the minimum energy structure, are shown in Fig. 1 and 2. A complete overview of all isomers found in this study can be found in Fig. S1–S4 and Tables S1–S3 (ESI†), together with their symmetry groups and relative energies calculated at different levels of theory.

The most stable isomers of the neutral clusters (NaCl)_*x*_ for *x* = 1–4 and *x* = 7 are similar to those found in previous studies, with slightly different bond lengths and angles.^20,31,33–37^ Born–Meyer potential based methods^31^ as well as Hartree–Fock (HF) and second order Møller–Plesset perturbation theory (MP2)^37^ predict a distorted hexagonal double-ring structure similar to N6b (+0.17 eV) as lowest energy structure for *x* = 6. According to HF and MP2 methods,^37^ the lowest energy structure for *x* = 8 is a distorted octagonal double-ring, which is not a stable minimum in our calculations. Isomer N5b (+0.01 eV), earlier calculated to be the lowest energy isomer for *x* = 5,^31,33–37^ is nearly isoenergetic with the energetically preferred structure N5a according to all methods used in our study (see ESI†).

The minimum energy isomers of the even-numbered neutral cluster sizes, N2a, N4a, N6a and N8a, resemble the main building blocks of the bulk salt crystal. The N2a isomer itself is the main building block of the larger minimum energy isomers N4a, N6a and N8a. This suggests that larger cluster sizes follow this trend and thus, consist of N2a, N4a, N6a and N8a building blocks. For the even-numbered neutral cluster sizes, the minimum energy isomer is clearly very stable (*e.g.*, the energy difference between N4a and N4b is 0.94 eV) while for the odd-numbered ones, several almost iso-energetic minimum energy isomers exist (see Fig. 1 and 2).

Regarding the anions (NaCl)_*x*_Cl^−^, the lowest energy isomers for cluster sizes *x* = 1,3,4,7 resemble those found before.^31–33^ Born–Meyer potential based methods^31,32^ and the inversion pair potential^33^ predict a linear structure to be energetically preferred for *x* = 2, which yields one imaginary frequency in our calculations. Born–Meyer potential based methods^31,32^ predict A5c (+0.14 eV) and the inversion pair potential^33^A5b (+0.11 eV) to be the global minimum for *x* = 5, in significant disagreement with our calculations. The same methods^32,33^ identify A6d (+0.09 eV) as the lowest energy configuration of *x* = 6. Furthermore, a structure similar to C8a was found as global minimum for anions with *x* = 8, which in our calculations is not stable. Optimizing this structure yields A8a, with the central chloride ion interacting with both eight-membered rings, resulting in *D*_4d_ symmetry.

For cationic clusters (NaCl)_*x*_Na^+^, our calculations result in similar minimum energy isomers as previous works for *x* = 1, 3–5, 8.^31,33,37^ Similar to the anions, Born–Meyer potential based methods,^31^ the inversion pair potential,^33^ HF and MP2 methods^37^ predict a straight line as energetically preferred for *x* = 2. In our calculations, this straight line yields one imaginary frequency using the B3LYP-D3 functional, but is a minimum using the ωB97XD functional (CCSD(T)/TZ//ωB97XD: +0.03 eV). Moreover, the distorted linear structure C2b lies 0.06 eV (CCSD(T)/TZ//B3LYP-D3) above the trigonal bipyramid C2a. Deviations are also found for larger clusters, with isomers C6b (HF and MP2)^37^ (+0.15 eV) or C6c (Born–Meyer potential)^31^ (+0.17 eV) found as lowest energy isomer for *x* = 6 and C7b (HF and MP2)^33,37^ (+0.07 eV) for *x* = 7.

With the exception of the cluster sizes *x* = 1, 3, 4, cations and anions do not share the same lowest energy isomer. But for most calculated local minima, interchanging Na^+^ and Cl^−^ ions produces similar isomers after re-optimization. However, the energy of equivalent isomers relative to the respective global minimum may differ significantly, see *e.g.*A4d and C4d. Furthermore, the relative energies of equivalent isomers do not necessarily follow the same order for cations and anions, *e.g.*A2a–A2c and C2a–C2c. For cluster sizes up to *x* = 4, each cationic isomer has an anionic counterpart with similar structure. For *x* > 4, the number of isomers increases rapidly, and anionic or cationic structures that are unique to their charge state are more frequently found (see Fig. S1–S4, ESI†).

### Cluster stability and dissociation energies

The high intensity of the magic clusters (NaCl)_4_Na^+^ and (NaCl)_4_Cl^−^ in mass spectra was previously explained by the energy needed to remove NaCl or the respective atomic ion Na^+^ and Cl^−^ from the cluster, which exhibited a local maximum for the magic cluster sizes.^20,33,37^ The underlying assumption in these studies was that NaCl or the atomic ion is eliminated from the minimum energy structure of the precursor, leading to an energetically higher-lying isomer of the product, as plotted in Fig. S5 (ESI†) for the cationic magic cluster (NaCl)_4_Na^+^. Furthermore, it was implicitly assumed that the clusters preferentially fragment *via* one of these pathways. The energies for this scenario can be found in Fig. S6 (ESI†) (dashed line) for the ionic clusters. However, Konermann and co-workers have shown that structural changes likely occur before the loss of small neutrals (NaCl)_*n*_ with *n* = 1–3, based on CID experiments and MD simulations.^18^

**Fig. 3:**
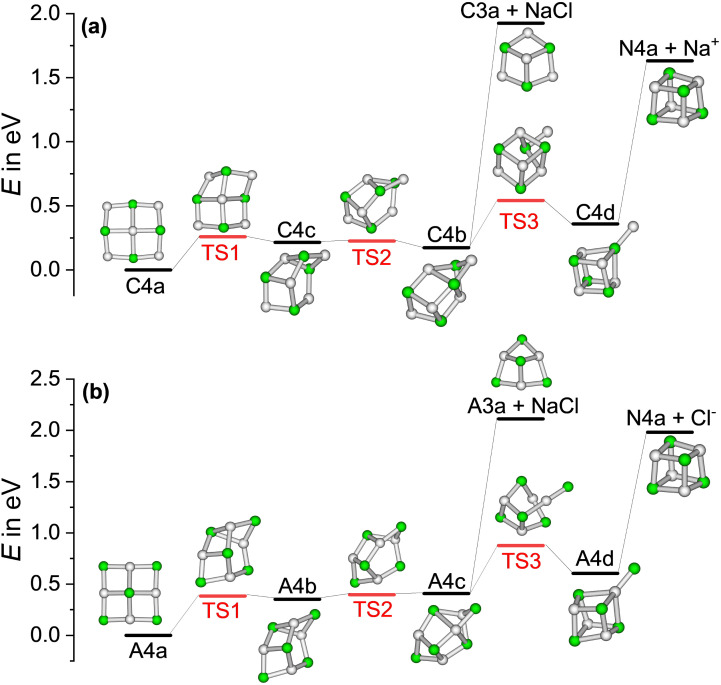
The potential energy surface (PES) for the loss of NaCl or Na^+^ and Cl^−^ from the (a) cationic and (b) anionic magic cluster *x* = 4, respectively. Calculations are performed at the CCSD/DZ//B3LYP-D3 level of theory.

This prompted us to analyze the minimum energy path for the loss of NaCl or the atomic ions Na^+^ and Cl^−^ for the magic clusters (NaCl)_4_Na^+^ and (NaCl)_4_Cl^−^, respectively. The calculated potential energy surfaces shown in Fig. 3 reveal that all rearrangements to higher-lying isomers of the precursor, which afford dissociation into the lowest energy structure of the fragments, are energetically well below the dissociation threshold. *E.g.* to eliminate NaCl from C4a, it is energetically more favorable for the cluster to rearrange *via*C4c to C4b, which contains the C3a structural motif as a subunit and can be directly reached *via* the loss of NaCl. Alternatively, it may rearrange to C4d, which can directly dissociate into the N4a cube and Na^+^. For comparison, the energy needed to dissociate Na^+^ directly from the minimum energy structure C4a of the cationic magic cluster (NaCl)_4_Na^+^ to form N4h is 1.19 eV higher than the minimum energy path, as shown in Fig. S5 (ESI†). These findings confirm previous studies,^21,27^ which suggested isomerization before fragmentation.

The calculated binding energies of NaCl or the atomic ion – assuming no isomerization before fragmentation (dashed line) *vs.* isomerization before fragmentation to afford dissociation into the lowest energy isomer of the products (solid line) – are plotted in Fig. S6 (ESI†) for cluster sizes *x* = 1–8. The data show that the ionic magic clusters with *x* = 4 do not exhibit a particular energetic stability with respect to these two loss channels, indicating that the energy arguments invoked before to explain this magic cluster size^20,33,37^ are not plausible.

**Fig. 4:**
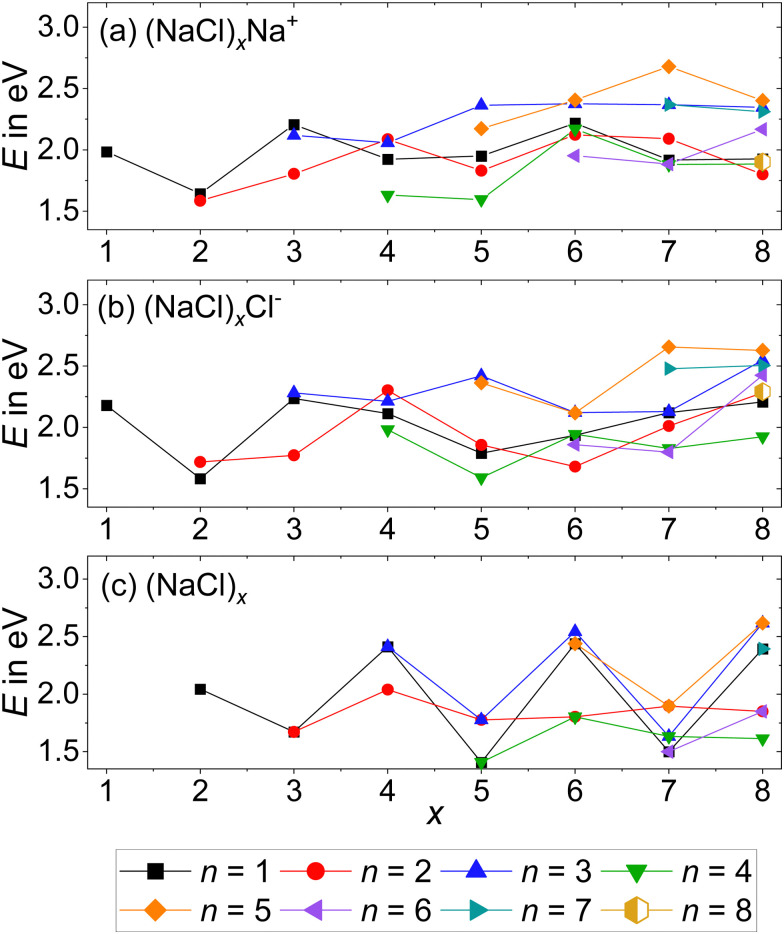
Dissociation energies of the (a) cations, reaction (2); (b) anions, reaction (3) and (c) neutrals, reaction (4) as a function of cluster size *x*. Dissociation from the lowest energy isomer of the precursor to the lowest energy isomers of the products is assumed. Calculations are performed at the CCSD/DZ//B3LYP-D3 level.

Since previous CID studies of sodium chloride clusters suggested the loss of small neutrals (NaCl)_*n*_ with *n* > 1,^21,38^ we performed a systematic assessment of energetic stability, considering all stoichiometric fragmentation pathways, *i.e.* the loss of neutral clusters (NaCl)_*n*_ with the stoichiometry of the bulk salt. In other words, the thermochemistry of reactions (2)–(4) is calculated with the lowest-lying isomer for all species, with the results summarized in Fig. 4 and Table S4 (ESI†).

2 (NaCl)_*x*_Na^+^ → (NaCl)_*x*–*n*_Na^+^ + (NaCl)_*n*_

3 (NaCl)_*x*_Cl^−^ → (NaCl)_*x*–*n*_Cl^−^ + (NaCl)_*n*_

4 (NaCl)_*x*_ → (NaCl)_*x*–*n*_ + (NaCl)_*n*_

Benchmarking calculations can be found in Fig. S7 and Table S4 (ESI†), showing differences within 0.05 eV upon change in the computational method and/or basis set. The calculations indicate that in general, loss of even-numbered neutral clusters (NaCl)_*n*_ is preferred over odd-numbered neutrals, which is reflected in the pronounced odd–even oscillations of the binding energies of neutral (NaCl)_*x*_ (Fig. 4c). The high stability of the neutral *x* = 4 cluster with *T*_d_ symmetry (see Table S3, ESI†) – which corresponds closely to the unit cell of the sodium chloride crystal structure – is responsible for the relatively low energy required to remove Na^+^ and Cl^−^ from (NaCl)_4_Na^+^ and (NaCl)_4_Cl^−^, respectively (Fig. 4a and b).

The anionic magic cluster (NaCl)_*x*_Cl^−^ with *x* = 4 indeed dissociates at higher energies than the neighboring cluster sizes *x* = 2, 3, 5–8, explaining its high abundance. For its cationic counterpart (NaCl)_*x*_Na^+^ with *x* = 4, however, the *n* = 4 loss channel lies energetically only slightly above the corresponding value for *x* = 5, and is lower than the minimum energy dissociation channel of most other cluster sizes. This shows that for (NaCl)_4_Na^+^, the dissociation energies cannot be the reason for the high abundance of this magic cluster size.

### Collision induced dissociation experiments

**Fig. 5:**
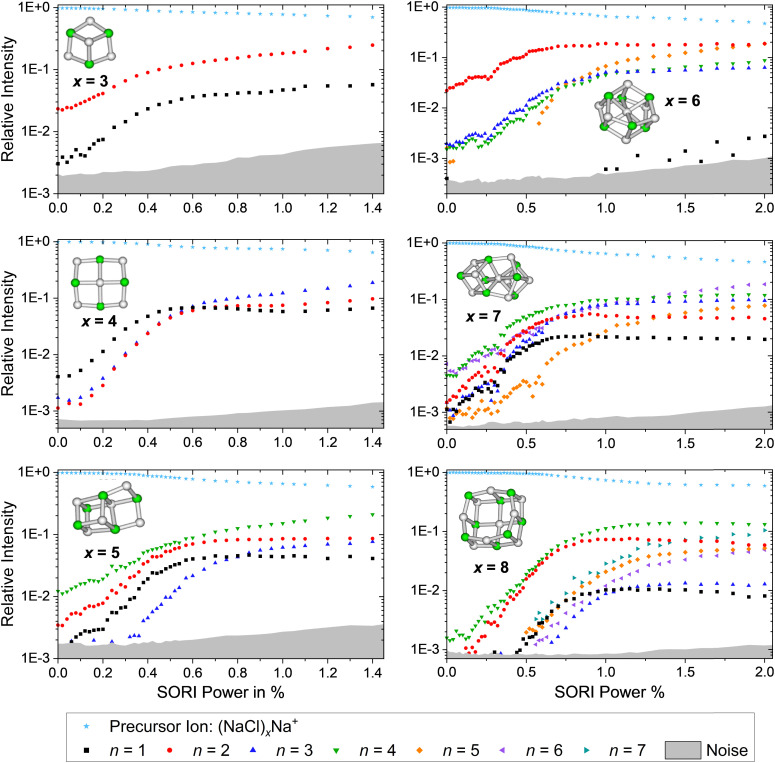
SORI CID spectra of the sodium chloride cluster ions (NaCl)_*x*_Na^+^ with *x* = 3–8. The stoichiometric loss channels (2) are observed.

SORI CID experiments are performed in order to get further insight into the dissociation behavior of sodium chloride cluster ions and to test whether there is a correlation with the calculated dissociation energies from Fig. 4. The SORI CID breakdown curves of the cations (NaCl)_*x*_Na^+^ with *x* = 3–8 are shown in Fig. 5, representative mass spectra in Fig. S25–S30 (ESI†). Unfortunately, Na^+^ lies below the lower mass limit of our FT-ICR MS instrument and cannot be detected. Due to the overlap of the precursor ion (NaCl)_6_Na^+^ with the doubly charged cluster (NaCl)_12_Na_2_^2+^, a correction has been made in the CID spectrum of the cluster size *x* = 6 based on the intensities of the fragment peaks originating from the doubly charged species, which occur at higher mass to charge ratios than the precursor ion peak, see Fig. S8 (ESI†). Analyzing the heights of the isotopic peaks – originating from singly charged (NaCl)_6_Na^+^ and doubly charged (NaCl)_12_Na_2_^2+^ – yields 58(5)% (NaCl)_6_Na^+^ and 42(5)% (NaCl)_12_Na_2_^2+^.

As predicted by the calculations in Fig. 4, the stoichiometric dissociation channels, reaction (2), are observed. Except for the magic cluster with *x* = 4, the loss of even-numbered neutral clusters (NaCl)_*n*_ with *n* = 2, 4, 6 is dominant, even if the magic cluster can be reached *via* the loss of odd-numbered neutrals, as is the case for *x* = 5 and *x* = 7. The main loss channel for cluster sizes *x* = 5, 7, 8 is the loss of the cube (NaCl)_4_, while the loss of the rhombus (NaCl)_2_ is dominant for *x* = 3, 6. For the cluster size *x* = 6, the relatively high contribution of the *n* = 3 loss channel compared to *n* = 4 might originate from shortcomings of the correction due to the overlap with doubly charged (NaCl)_12_Na_2_^2+^, *i.e.* it may in part be due to loss of (NaCl)_6_ from (NaCl)_12_Na_2_^2+^. Likewise, the loss of (NaCl)_4_ from (NaCl)_12_Na_2_^2+^ – contributing to the *n* = 2 loss channel from (NaCl)_6_Na^+^ – cannot be ruled out. The magic cluster (NaCl)_4_Na^+^ is the only cationic cluster in our experiment, for which the loss of NaCl is dominant. The loss of the neutral cube (NaCl)_4_ from (NaCl)_4_Na^+^ – which is the lowest energy dissociation channel (Fig. 4) – cannot be detected because the Na^+^ ion lies outside the *m*/*z* range of our FT-ICR MS experiment.

Neglecting the Na^+^ loss, the SORI CID results (Fig. 5) agree with the reaction energies (Fig. 4) for cluster sizes *x* = 3–5. For cluster size *x* = 6, reaction energies indicate a high contribution of (NaCl)_4_ and (NaCl)_6_ loss from (NaCl)_12_Na_2_^2+^, increasing the intensity of the *n* = 2 and *n* = 3 loss channel, respectively. Apart from loss of NaCl, *n* = 1, which according to the energetics should be more prominent, reaction energies agree well with the relative fragment abundance of cluster size *x* = 7 and the dominant loss channels *n* = 2, 4 of cluster size *x* = 8.

**Fig. 6:**
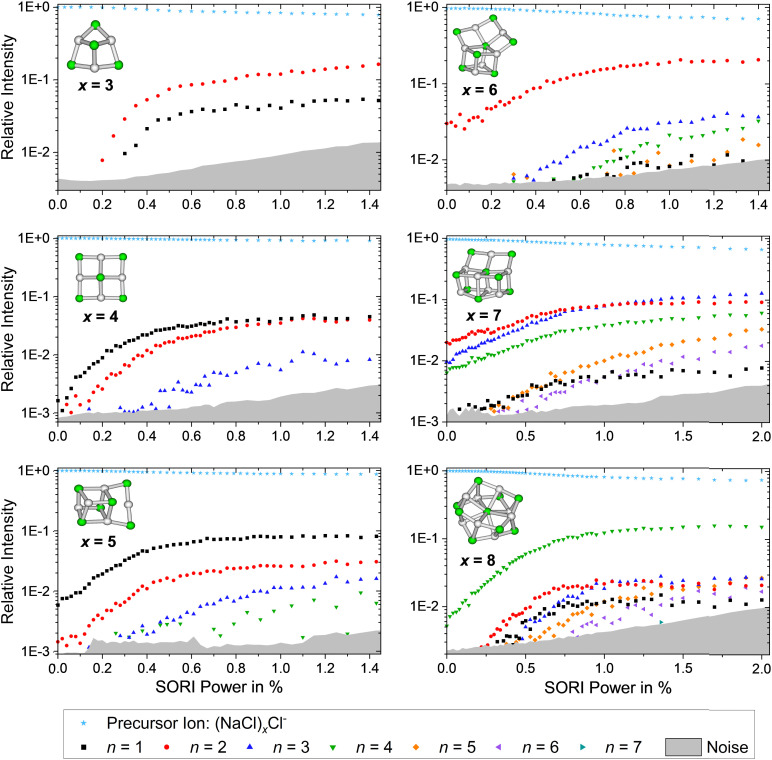
SORI CID spectra of the sodium chloride cluster ions (NaCl)_*x*_Cl^−^ with *x* = 3–8. The stoichiometric loss channels (3) are observed.

The SORI CID breakdown curves of the anions (NaCl)_*x*_Cl^−^ with *x* = 3–8 are shown in Fig. 6. As already observed for the cations, the stoichiometric fragmentation channels, reaction (3), are present. However, for the anions, both *x* = 4 and *x* = 5 predominantly lose a single NaCl unit while (NaCl)_2_ is preferentially lost from *x* = 3, 6, 7. Only (NaCl)_8_Cl^−^ eliminates (NaCl)_4_, forming the magic cluster (NaCl)_4_Cl^−^ as the dominant channel. In contrast to the cations, however, loss of (NaCl)_4_ does not play a major role for the smaller anionic clusters.

Reaching the magic cluster (NaCl)_4_Cl^−^ seems to be a significant factor in the dissociation behavior of the anionic species. For *x* = 5, 6, 8, the dominant fragmentation channel leads to the magic cluster *x* = 4, and the branching ratio of this dominant channel is much larger than for the cationic species. For the anionic cluster size *x* = 7, the loss of the odd neutral cluster *n* = 3 is the second appearing loss channel, quite competitive with the dominant loss of (NaCl)_2_, while for the cationic counterpart, the loss of even-numbered neutral clusters (NaCl)_*n*_ with *n* = 2, 4, 6 predominates. Overall, the anions slightly prefer losing even-numbered neutral clusters (NaCl)_*n*_ with *n* = 2, 4, but they are more prone to decay to the magic cluster size *x* = 4 than the cations. Similar to the cationic species, the magic cluster (NaCl)_4_Cl^−^ preferentially loses NaCl, although the energetically preferred pathway is loss of Cl^−^ (*n* = 4 loss channel, Fig. 4). Since *m*/*z* 35 lies above the lower mass limit of our instrument, we should have observed this fragment if it was formed. However, we did not observe the Cl^−^ fragment in any of the anionic cluster SORI CID experiments. The energetically preferred *n* = 4 loss channel of cluster size *x* = 5 is by far the least abundant in the experiment. Only for cluster sizes *x* = 3, 6, 8, the observed dominant loss channels are also energetically preferred according to the calculations.

The dominant loss of even-numbered neutral cluster sizes (NaCl)_*n*_ – mostly *n* = 2, 4 – by anions as well as cations can be explained by the favorable stability of these neutrals (Fig. 4) which have rhombic and nearly cubic structure, resembling sections of the sodium chloride crystal lattice (Fig. 1). Our SORI CID results are in sharp contrast to the assumption that sodium chloride clusters preferentially fragment *via* the loss of single NaCl units or the respective atomic ion.^20,33,37^ In most cases, other loss channels are dominant, which are often accessible at significantly lower energies than the loss of NaCl or the atomic ion. As a consequence, the binding energy of NaCl or the atomic ion is not a meaningful indicator for relative cluster stability or magic cluster sizes.

### Statistical analysis

Since the energetics alone do not explain the observed dissociation behavior – in particular the missing Cl^−^ fragment and the origin of the magic (NaCl)_4_Na^+^ cluster – we conducted a statistical analysis of the population of different isomers. This will provide an idea how readily the clusters rearrange, and which cluster structures are actually populated at elevated energies in a CID experiment. To this end, the DOS of the low-lying isomers is calculated with the Beyer–Swinehart^52^ algorithm as described by Gilbert and Smith.^53^

**Fig. 7:**
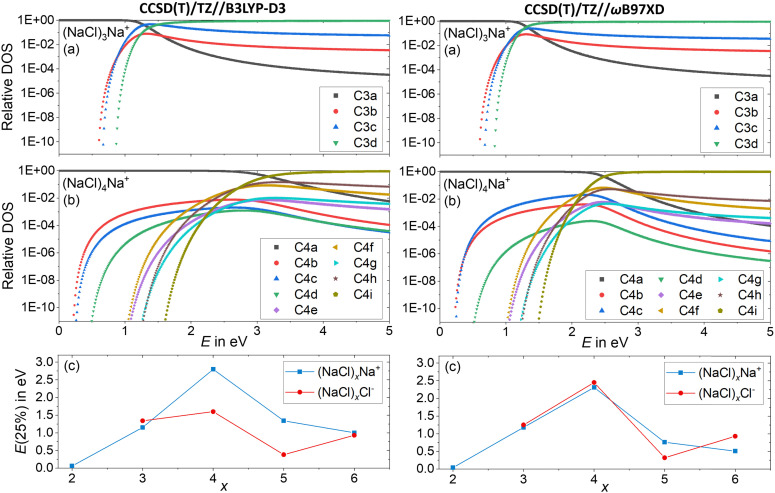
Relative density of states (DOS) of the cationic clusters (NaCl)_*x*_Na^+^ with (a) *x* = 3 and (b) *x* = 4. The isomers considered are shown in Fig. 1 and Fig. S1 (ESI†). (c) The energy needed to redistribute 25% of the lowest energy isomer into higher-lying isomers as a function of the cluster size *x* for anions and cations.

Fig. 7a and b shows the relative DOS as a function of internal energy of the cationic clusters (NaCl)_*x*_Na^+^ with *x* = 3 and *x* = 4, which describes the population of the different isomers.^54^ The relative DOS for the cationic and anionic clusters with *x* = 2–6 is plotted in Fig. S9–S12 (ESI†). The DOS of the C3a and the C4a populations (Fig. 7a and b) shows that for the magic cluster size *x* = 4, a higher energy is needed to significantly populate higher-lying isomers than for the neighboring cluster size *x* = 3. This is not what one would expect based on the relative energies of the different isomers of these two cluster sizes. While the relative energy between the two lowest energy isomers C3a and C3b for *x* = 3 is 0.60 eV, that of C4a and C4b for *x* = 4 is only 0.22 eV (Fig. 1). The highest TS connecting C4a and C4b*via*C4c lies only 0.26 eV above C4a (Fig. 3a) indicating that interconversion between these isomers is facile at energies below 0.5 eV.

Consistent with these energies, the higher-lying isomers of *x* = 4 start being populated at smaller energies than those of *x* = 3. Yet, the population of C4b–C4e and C4g never exceeds 1%. The much higher-lying isomer C4i with a relative energy of 1.44 eV is the first isomer surpassing C4a at an energy of 3.2 eV according to CCSD(T)/TZ//B3LYP-D3 and 2.5 eV according to CCSD(T)/TZ//ωB97XD. The reason for this peculiar behavior lies in the structure of C4a with *C*_2v_ symmetry: the almost planar, square structure of the cluster leads to low-lying vibrational modes (lowest vibrational modes: 8 cm^−1^, 18 cm^−1^), which are the reason for the high density of states of this isomer. Only the higher-energy isomers C4e–C4i with relative energies between 0.89 eV and 1.44 eV have even lower-lying vibrational modes, which allows these isomers to surpass C4a at higher internal energies. The chain structure C4i has by far the lowest-lying vibrational modes (lowest vibrational modes: 5 cm^−1^, 6 cm^−1^) and thus dominates the population at higher energies. The energetically lower lying isomers C4b–C4d – with relative energies between 0.17 eV and 0.36 eV – never exceed the C4a population due to their higher lying vibrational modes (lowest vibrational modes: 19 cm^−1^ (C4b), 30 cm^−1^ (C4c) and 25 cm^−1^ (C4d)) leading to a small DOS for all energies.

For a comparison, the minimum energy isomer C3a has higher-lying vibrational modes (lowest vibrational modes: 55 cm^−1^, 84 cm^−1^) than the energetically higher lying isomers C3b–C3d (lowest vibrational modes: 10 cm^−1^ (C3b), 8 cm^−1^ (C3c), 6 cm^−1^ (C3d)) leading to this rapid increase of the C3b–C3d populations with increasing energy, as shown in Fig. 7a.

Low-lying vibrational modes increase the number of possibilities for the distribution of the internal energy into the different vibrational modes, increasing the DOS. Fig. 7a and b shows that for small energies, the population of the different isomers is mainly influenced by the relative energy of the isomers. With increasing internal energy (above about 1 eV), the DOS increase outweighs relative energies, until the isomer population is shifted towards isomers featuring low-frequency vibrational modes.

Near the threshold energy for dissociation around 2 eV, the magic cluster size *x* = 4 is the only cluster size, which mainly (relative DOS of C4a > 99%) populates the minimum energy isomer C4a, whereas for the neighboring cluster sizes (between *x* = 2 and *x* = 6) the population of higher lying isomers already surpassed that of the respective minimum energy isomer C*x*a significantly (Fig. S9 and S10, ESI†). For a compact comparison of the different cluster sizes, the energy needed to redistribute 25% of the lowest energy isomer into higher-lying isomers is plotted as a function of cluster size *x* in Fig. 7c. Thus, for the cationic as well as the anionic magic cluster *x* = 4, a higher energy is needed to populate higher-lying isomers to a significant extent than for the neighboring cluster sizes. Using CCSD(T)/TZ//B3LYP-D3, this effect is less pronounced for the anions. Overall, the DOS calculations provide a qualitative idea how readily the clusters leave their minimum geometry with increasing internal energy.

**Fig. 8:**
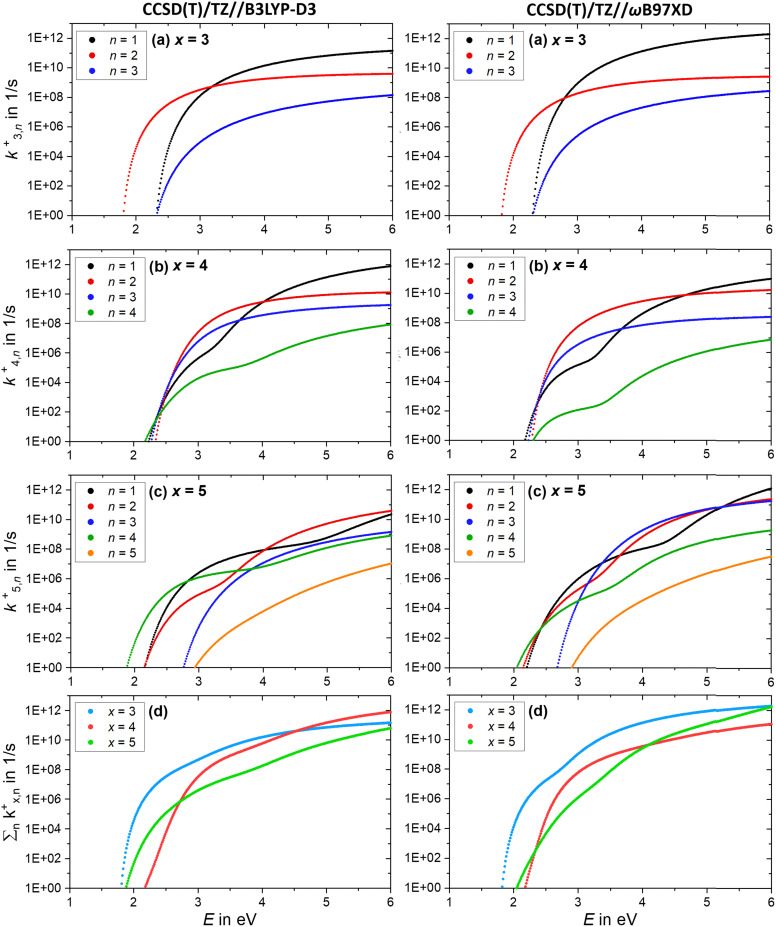
(a)–(c) Calculated RRKM rate constants *k*^+^_*x,n*_ for the loss channels (2) of the precursor ions (NaCl)_*x*_Na^+^ with *x* = 3–5. Note that the loss channels producing Na^+^ ions (*k*^+^_*x*,*n*=*x*_), which cannot be detected in the experiment, are always the slowest at higher energies. (d) Total fragmentation rate constants 
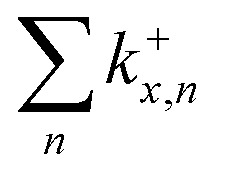
 as sum of all loss channels *n* for cluster sizes *x* = 3–5.

Further insight is obtained from the unimolecular dissociation rate constants *k*^+/−^_*x*,*n*_ calculated with the AWATAR version of RRKM theory, eqn (1), which accounts for the population of higher lying isomers. Fig. 8a–c shows the results for cationic clusters (NaCl)_*x*_Na^+^ with *x* = 3–5 for all stoichiometric loss channels (2). Method benchmarking can be found in the ESI,† Fig. S13–S17. While the curves in Fig. 8a show the typical energy dependence of RRKM rate constants, the *n* = 1, 4 curves in Fig. 8b and *n* = 1, 2, 4 in Fig. 8c exhibit marked dents, which arise from the contribution of higher-lying isomers in the sum of states term of the AWATAR eqn (1).

For all three cluster sizes, loss of Na^+^ is the slowest channel over the most part of the energy range. Although we cannot detect Na^+^ in the experiment, these results indicate that we do not miss a dominant dissociation channel. Even for the CCSD(T)/TZ//B3LYP-D3 calculations, the lowest-energy loss channel *n* = 4 of the magic cluster size *x* = 4 quickly levels off, Fig. 8b, left column, and is surpassed by the other loss channels at slightly higher energies. This behavior of the Na^+^ loss channel (*k*^+^_*x*,*n*=*x*_) results from the relatively small contribution of a monatomic fragment to the sum of states in the loose transition state calculation.

Near threshold, the AWATAR rate constants as a function of energy reflect the behavior of the experimentally measured intensities of the corresponding CID channels, Fig. 5, quite well for *x* = 3, 5. For *x* = 4, all channels compete near threshold in the AWATAR calculations, and the preferential loss of NaCl is not mirrored in the rate constants. However, a comparison of the SORI CID results with the calculated rate constants is not straightforward, since the kinetic energy distribution is quite broad in SORI CID. Guided ion beam experiments^50^ would allow for a more quantitative comparison over the entire energy range.

The comparison of the overall rate constant for dissociation, Fig. 8d, gives a clear indication why the *x* = 4 cluster is magic. Independent of the theory level, the AWATAR dissociation rate constants rise only at higher internal energies than for *x* = 3, 5. If we assume that the (NaCl)_4_Na^+^ cluster is formed by attaching a Na^+^ ion to a thermalized (NaCl)_4_ neutral, followed by rearrangement to the near-planar geometry, the available energy in the system is around 1.8 eV. At this energy, the lifetime of the cluster exceeds 1 s, and there is plenty of time to stabilize the cluster either by collisions or emission of infrared radiation.

**Fig. 9:**
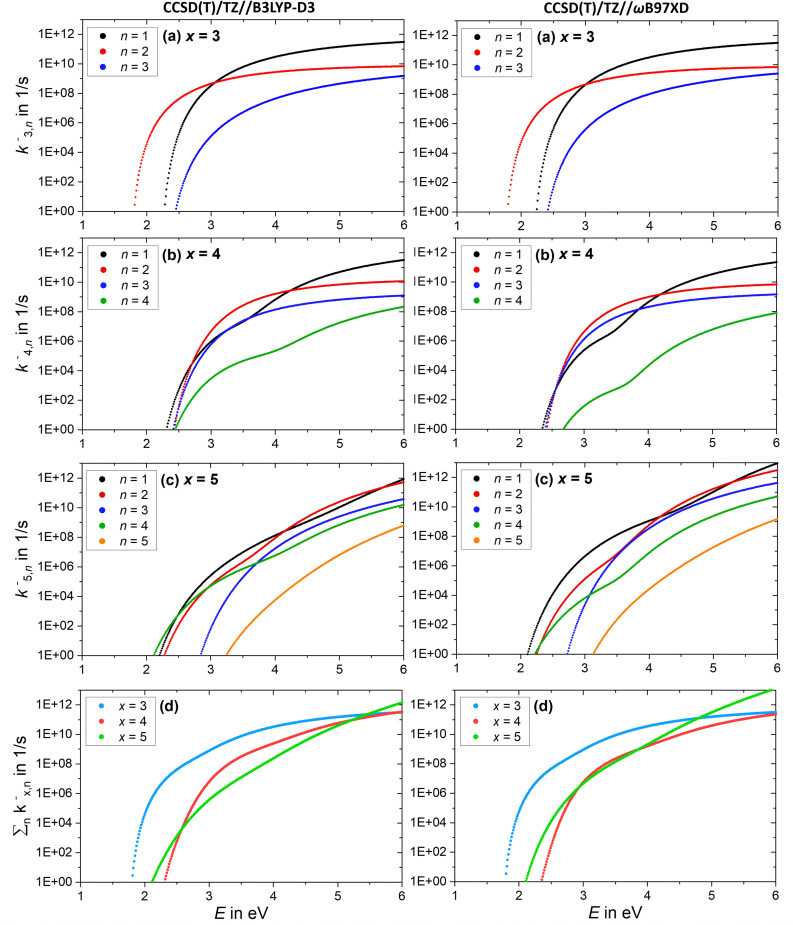
(a)–(c) Calculated RRKM rate constants *k*^−^_*x,n*_ for the loss channels (3) of the precursor ions (NaCl)_*x*_Cl^−^ with *x* = 3–5. Note that the loss channels producing Cl^−^ ions (*k*^−^_*x*,*n*=*x*_) are always the slowest. (d) Total fragmentation rate constants 
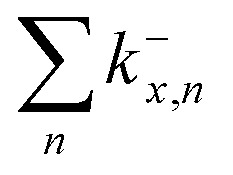
 as sum of all loss channels *n* for cluster sizes *x* = 3–5.

The AWATAR rate constants *k*^−^_*x,n*_ of the anionic clusters (NaCl)_*x*_Cl^−^ with *x* = 3–5 for the stoichiometric loss channels (3) are plotted in Fig. 9a–c. Method benchmarking can again be found in the ESI,† Fig. S18–S22. As discussed above, the mass to charge ratio of Cl^−^ lies within the measurable range of our FT-ICR MS, thus this ion should be observed if formed. However, for the anionic clusters, Cl^−^ loss (*k*^−^_*x*,*n*=*x*_) is the slowest dissociation channel for all cluster sizes over the full energy range, and for the most part, several orders of magnitude slower than all other loss channels. Similar to the cations, this is due to the small contribution of an atomic ion to the sum of states in the loose transition state calculation. The absence of the Cl^−^ signal in the SORI CID experiments (Fig. 6) is thus fully consistent with the AWATAR calculations, despite loss of Cl^−^ being the lowest-energy fragmentation channel, Fig. 4b, for the magic cluster size *x* = 4.

Near threshold, the calculated rate constants as a function of energy reproduce the behavior of the experimentally measured intensities of the corresponding CID channels (Fig. 5) quite well. Only the *n* = 4 loss channel of the cluster size *x* = 5 is overestimated at small energies for the CCSD(T)/TZ//B3LYP-D3 results (Fig. 9c), which predict the *n* = 4 loss channel to be dominant.

The total fragmentation rate constant as sum of all loss channels for cluster sizes *x* = 3–5, Fig. 9d, is again the smallest for the magic cluster size *x* = 4 at energies around 2 eV. Applying similar arguments as for its cationic counterpart, Cl^−^ attaching to neutral (NaCl)_4_ induces the isomerization to the near-planar geometry, and the lifetime at the available energy of about 2 eV in the cluster is again so high that the cluster can be stabilized more readily than the neighboring cluster sizes.

## Conclusions

For the optimized geometries and relative energies of sodium chloride cluster isomers, the main discrepancies with respect to previous studies are found for works using empirical potentials, such as Born–Meyer potential based methods^31,32^ and the inversion pair potential,^33^ while our results are in very good agreement with previous CCSD(T) calculations.^35^ Benchmarking with several high-level methods shows that the results are very robust, with deviations below 0.05 eV between the different methods.

Our calculations show that even the smallest sodium chloride clusters exhibit a rich structural variety, which carry structural motifs from bulk salt. Rearrangements between different isomers of a specific cluster size have transition states well below the dissociation threshold, allowing for isomerization before fragmentation, as already suggested by MD simulations and ion mobility studies.^21,27^ This indicates that the fragmentation pathways leading to the lowest energy product isomers are accessible. Our findings are in stark contrast to earlier theoretical studies,^20,33,37^ where fragmentation was assumed to occur from the minimum energy structure of the precursor cluster ion, resulting in energetically higher-lying isomers of the products.

The SORI CID experiments show that anionic as well as cationic sodium chloride cluster ions preferentially lose even-numbered neutral clusters (NaCl)_*n*_, mostly *n* = 2, 4, with the notable exception of the magic clusters (NaCl)_4_Na^+^ and (NaCl)_4_Cl^−^. According to our calculations, neutral clusters with *n* = 2, 4 exhibit an especially high stability, with their rhombic or near-cubic structure resembling sections of the sodium chloride crystal lattice. These results complement CID studies previously performed by Konermann and co-workers,^21^ who suggested a sequential loss of small neutrals (NaCl)_*n*_ with *n* = 1–3, but could not rule out the loss of larger neutral clusters. As a consequence, all stoichiometric loss channels (2), (3) have to be considered when searching for the energetically lowest dissociation channel of a particular cluster. Analysis of the energetics of all possible loss channels, however, does not show a particular energetic stability of the magic clusters (NaCl)_4_Na^+^ and (NaCl)_4_Cl^−^, which is at odds with the traditional view that dissociation energies are the origin of magic cluster sizes.

Statistical analysis of the isomer population reveals that at threshold energy, the anionic as well as cationic magic cluster *x* = 4 will mainly populate the lowest energy isomer (C4a and A4a with *C*_2v_ symmetry) due to low-lying vibrational modes, while the neighboring cluster sizes already populate a variety of higher-lying isomers at moderate internal energy. The low-lying vibrational modes of these near-planar magic clusters lead to a high density of states, which suggests an entropic contribution to their magic properties.

The calculated AWATAR rate constants *k*^+^_*x*,*n*_ and *k*^−^_*x*,*n*_ for cationic and anionic clusters largely agree with our experimental results – especially near threshold –, providing a helpful tool for further analysis. In particular, the total absence of the atomic Cl^−^ fragment in the experiment is reflected in the low rate constants of this loss channel, and suggests that also Na^+^ loss does not take place to a significant extent. Particularly interesting is the Na^+^ loss channel from (NaCl)_4_Na^+^, which has a relatively low energetic barrier. Here, the small contribution of the atomic fragment to the sum of states in the loose transition state together with the relatively high-frequency of the vibrations in the (NaCl)_4_ cubic structure and the low-lying modes of the near-planar (NaCl)_4_Na^+^ are responsible for the exceedingly low rate constant. Similar arguments apply to the loss of Cl^−^ from (NaCl)_4_Cl^−^. The high intensity of (NaCl)_4_Na^+^ and (NaCl)_4_Cl^−^ is thus a largely entropic effect, in case of (NaCl)_4_Cl^−^ enhanced by the energetics.

## Data availability

Coordinates of optimized structures, SORI-CID fragment intensities and all numerical data shown in Fig. 4–9 are available as ESI.†

## Author contributions

Jessica C. Hartmann: data curation; formal analysis; investigation; visualization; writing – original draft; Sarah J. Madlener: investigation; validation; writing – review & editing; Christian van der Linde: data curation; formal analysis; methodology; project administration; resources; supervision; writing – review & editing; Milan Ončák: conceptualization; funding acquisition; methodology; project administration; resources; supervision; validation; writing – review & editing; Martin K. Beyer: conceptualization; funding acquisition; methodology; project administration; resources; software; supervision; validation; writing – review & editing.

## Conflicts of interest

The authors have no conflicts to disclose.

## Supplementary Material

CP-026-D4CP00357H-s001

CP-026-D4CP00357H-s002

CP-026-D4CP00357H-s003
