# Supplementary material for: Magic cluster sizes of cationic and anionic sodium chloride clusters explained by statistical modeling of the complete phase space
Source: Phys Chem Chem Phys. 2024 Mar 11;26(14):10904–18. doi: 10.1039/d4cp00357h (PMC10989714; doi:10.1039/d4cp00357h)
Supplement: CP-026-D4CP00357H-s001 [file CP-026-D4CP00357H-s001.pdf]

Electronic Supplementary Material (ESI) for Physical Chemistry Chemical Physics.
This journal is © the Owner Societies 2024

Magic Cluster Sizes of Cationic and Anionic Sodium Chloride Clusters Explained by Statistical Modeling of the Complete Phase Space

Jessica C. Hartmann, Sarah J. Madlener, Christian van der Linde, Milan Ončák, Martin K. Beyer**

Universität Innsbruck, Institut für Ionenphysik und Angewandte Physik, Technikerstraße 25, 6020 Innsbruck, Austria

Email: milan.oncak@uibk.ac.at; martin.beyer@uibk.ac.at

Supplementary Material

1. Optimized Structures

1.1 Cationic Clusters

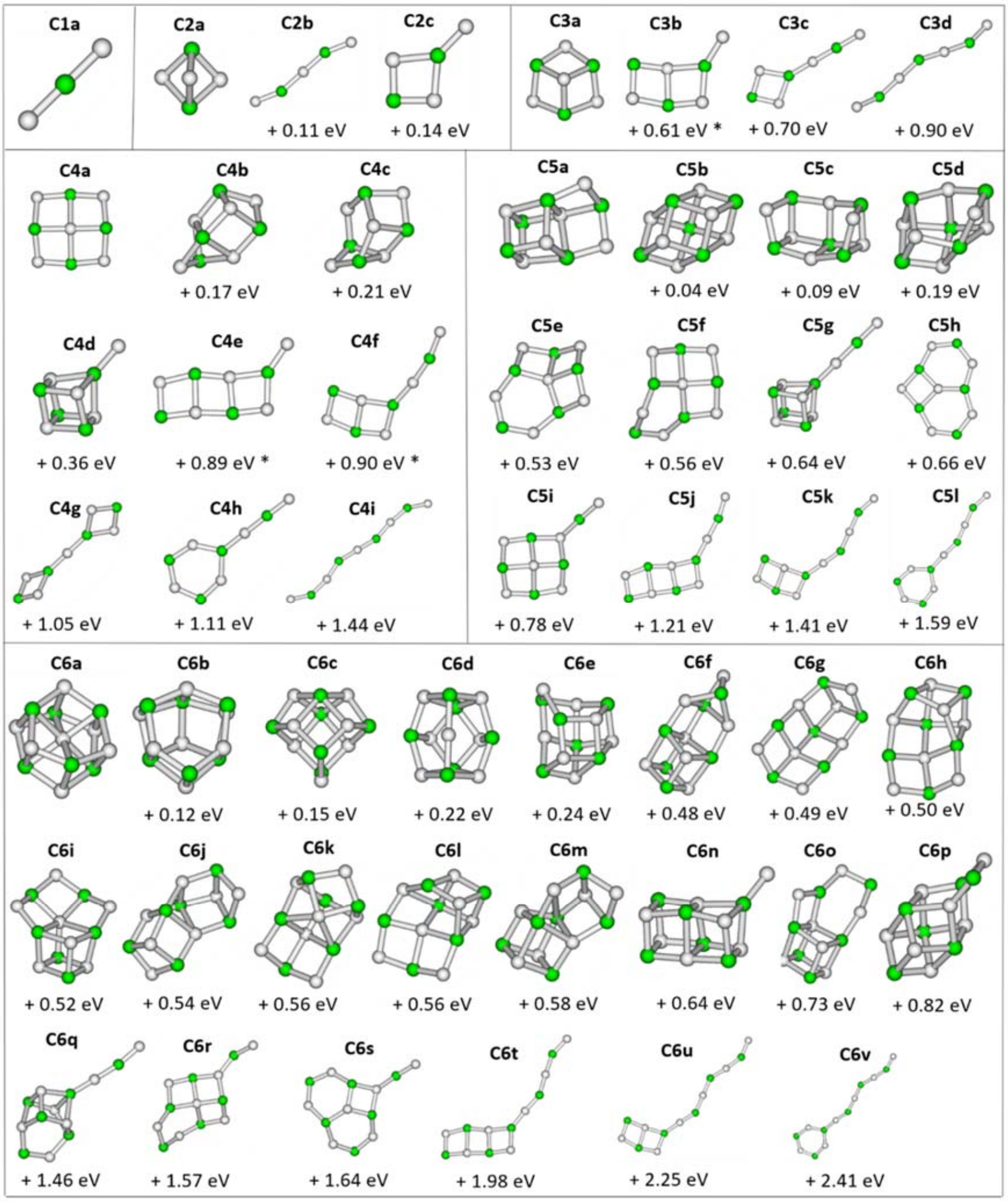

Figure S1: Overview of the lowest-energy isomers with relative energies calculated at the CCSD/DZ//B3LYP-D3 level of theory for the cationic clusters $(NaCl)_xNa^+$, x = 1–6. Energies marked with * are obtained by using the ωB97XD functional for the geometrical optimization due to the presence of an imaginary frequency using the B3LYP-D3 functional.

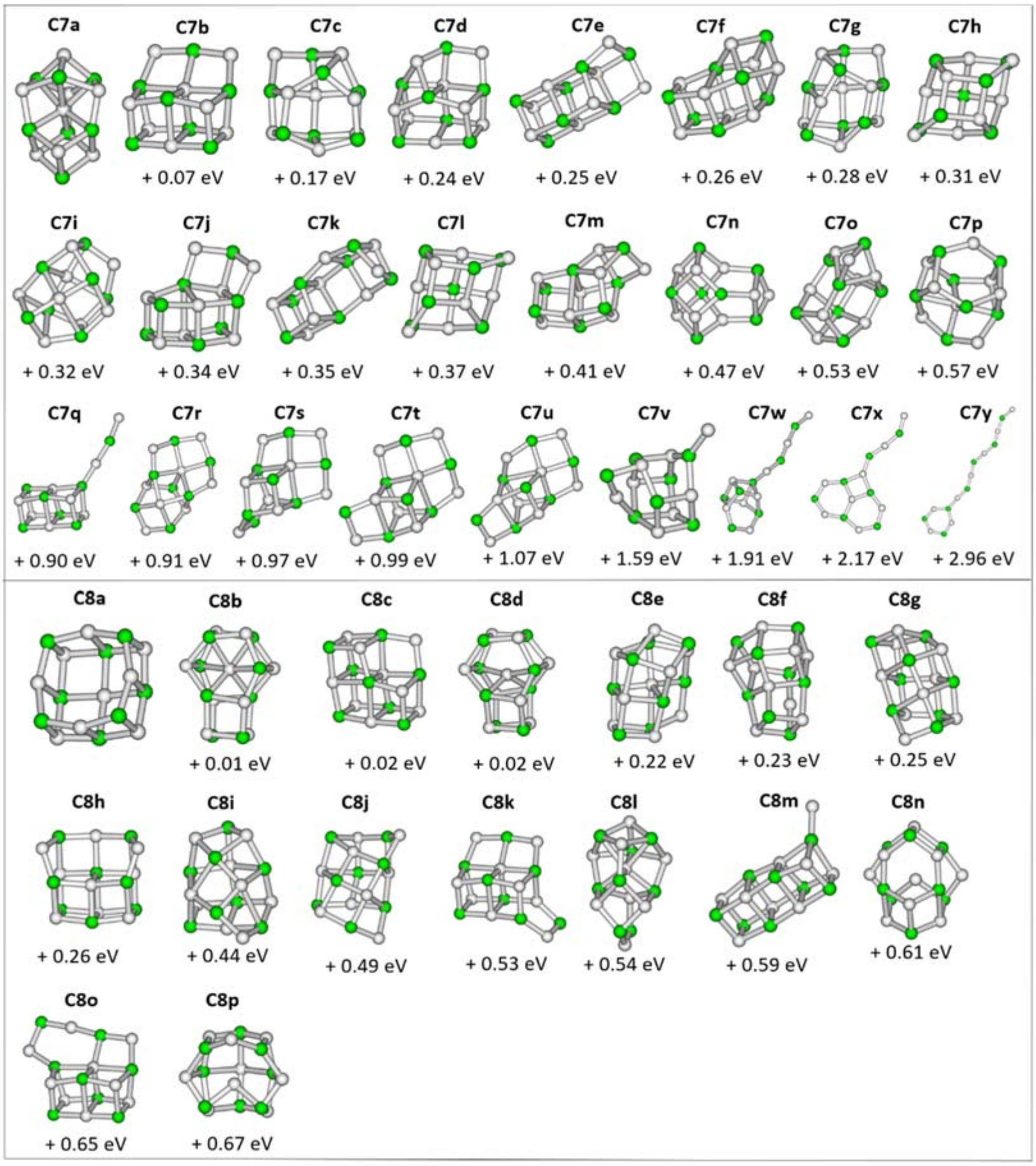

Figure S2: Overview of the lowest-energy isomers with relative energies calculated at the CCSD/DZ//B3LYP-D3 level of theory for the cationic clusters $(NaCl)_xNa^+$ with x = 7,8.

Table S1: Overview of the lowest-energy isomers including symmetry groups and relative energies of $(NaCl)_xNa^+$. Isomers whose energy is marked with * have one imaginary frequency within the listed symmetry group and thus, the listed energy corresponds to a structure with lower symmetry. Imaginary frequency for given functional: i. f.

Isomer	Symmetry Group	B3LYP-D3/ aug-cc-pVDZ	CCSD/DZ// B3LYP-D3	CCSD/TZ// B3LYP-D3	CCSD(T)/TZ/ /B3LYP-D3	ωB97XD/ aug-cc-pVDZ	CCSD/DZ// ωB97XD	CCSD/TZ// ωB97XD	CCSD(T)/TZ/ /ωB97XD
C1a	$D_{\infty h}$	0.00	0.00	0.00	0.00	0.00		0.00	0.00
C2a	D_{3h}	0.00	0.00	0.00	0.00	0.00		0.00	0.00
C2b	C_2 (°$D_{\infty h}$)	0.19	0.11	0.04	0.06	0.20°		0.01°	0.03°
C2c	C_{2v}	0.16	0.14	0.10	0.11	0.19		0.09	0.10
C3a	C_{3v}	0.00	0.00	0.00	0.00	0.00	0.00	0.00	0.00
C3b	C_1	i. f.				0.65	0.61	0.59	0.60
C3c	C_{2v}	0.84	0.70	0.64	0.66	0.77	0.70	0.66	0.67
C3d	C_2	1.07	0.90	0.83	0.85	0.99	0.87	0.79	0.81
C4a	C_{2v}	0.02	0.00	0.00	0.00	0.00	0.00	0.00	0.00
C4b	C_s	0.00	0.17	0.24	0.22	0.06	0.17	0.24	0.23
C4c	C_2	0.02	0.21	0.26	0.25	0.15	0.20	0.25	0.24
C4d	C_{3v}	0.23	0.36	0.44	0.42	0.27	0.37	0.45	0.44
C4e	C_s	i. f.				0.89	0.89	0.90	0.91
C4f	C_s	i. f.				0.93	0.90	0.89	0.91
C4g	D_{2d}	1.11	1.05	1.06	1.07	i. f.			
C4h	C_{2v}	1.11	1.11	1.10	1.12	i. f.			
C4i	C_s	1.58	1.44	1.41	1.44	1.50		1.35	1.38
C5a	C_s	0.00	0.00	0.00	0.00	0.01	0.00	0.00	0.00
C5b	D_{3h}	0.05	0.04	0.04	0.03	0.00	0.09	0.08	0.08
C5c	C_{2v}	0.13	0.09	0.08	0.08	0.15	0.09	0.07	0.08
C5d	C_1	0.14	0.19	0.19	0.19	i. f.			
C5e	C_2	0.68	0.53	0.46	0.49	0.67		0.44	0.46
C5f	C_1	0.72	0.56	0.50	0.52	0.67		0.50	0.52
C5g	C_{3v}	0.83	0.64	0.61	0.62	0.71		0.62	0.64
C5h	C_{2v}	0.80	0.66	0.57	0.61	i. f.			
C5i	C_1	1.05	0.78	0.70	0.72	0.91		0.66	0.68
C5j	C_1	1.54	1.21	1.11	1.15	1.37		1.12	1.15
C5k	C_s	1.77	1.41	1.31	1.35	1.58*		1.29*	1.33*
C5l	C_s	1.87	1.59	1.48	1.54	i. f.			
C6a	D_{3d}	0.00	0.00	0.00	0.00	0.00		0.00	0.00
C6b	C_{3v}	0.10	0.12	0.12	0.15	0.29		0.09	0.12
C6c	C_{2v}	0.13	0.15	0.14	0.17	0.36		0.10	0.13
C6d	C_{2v}	0.14	0.22	0.21	0.23	0.32		0.21	0.23
C6e	C_1	0.17	0.24	0.23	0.25	0.38		0.21	0.23
C6f	C_s	0.48	0.48	0.48	0.50	0.60		0.47	0.49
C6g	C_s	0.69	0.49	0.43	0.47	0.75		0.40	0.44
C6h	C_1	0.59	0.50	0.47	0.50	0.68		0.43	0.46
C6i	C_s	0.58	0.52	0.47	0.50	0.72		0.42	0.45
C6j	C_1	0.56	0.54	0.52	0.55	0.69		0.49	0.52
C6k	C_s	0.59	0.56	0.52	0.54	0.68		0.47	0.49
C6l	C_1	0.65	0.56	0.51	0.54	0.70		0.49	0.51
C6m	C_1	0.53	0.58	0.56	0.59	i. f.			
C6n	C_s	0.67	0.64	0.64	0.66	0.79		0.63	0.65
C6o	C_1	0.77	0.73	0.70	0.73	0.90		0.67	0.71
C6p	C_s	0.84	0.82	0.79	0.82	0.91		0.77	0.79
C6q	C_s	1.60	1.46	1.39	1.44	i. f.			

Isomer	Symmetry Group	B3LYP-D3/ aug-cc-pVDZ	CCSD/DZ// B3LYP-D3	CCSD/TZ// B3LYP-D3	CCSD(T)/TZ/ /B3LYP-D3	ωB97XD/ aug-cc-pVDZ	CCSD/DZ// ωB97XD	CCSD/TZ// ωB97XD	CCSD(T)/TZ/ /ωB97XD
C6r	C_1	1.87	1.57	1.48	1.54	1.87		1.43	1.49
C6s	C_s	1.89	1.64	1.54	1.61	2.00		1.44	1.51
C6t	C_s	2.44	1.98	1.85	1.92	2.32*		1.79*	1.87*
C6u	C_1	2.72	2.25	2.11	2.19	2.60		2.04	2.12
C6v	C_s	2.81	2.41	2.27	2.37	i. f.			
C7a	C_{3v}	0.00	0.00			0.00			
C7b	C_{2v}	0.10	0.07			0.20			
C7c	C_1	0.08	0.17			0.24			
C7d	C_1	0.14	0.24			0.24			
C7e	C_s	0.26	0.25			0.35			
C7f	C_s	0.22	0.26			0.28			
C7g	C_1	0.16	0.28			i. f.			
C7h	C_{2v}	0.26	0.31			i. f.			
C7i	C_1	0.12	0.32			0.25			
C7j	C_1	0.27	0.34			0.42			
C7k	C_s	0.40	0.35			0.47			
C7l	C_s	0.19	0.37			0.42			
C7m	C_1	0.31	0.41			0.28			
C7n	C_s	0.40	0.47			0.53			
C7o	C_s	0.53	0.53			i. f.			
C7p	C_2	0.45	0.57			0.56			
C7q	C_s	1.08	0.90			1.05			
C7r	C_s	0.95	0.91			1.05			
C7s	C_s	1.00	0.97			1.12			
C7t	C_1	1.02	0.99			i. f.			
C7u	C_1	1.14	1.07			1.13			
C7v	C_s	1.54	1.59			i. f.			
C7w	C_s	2.17	1.91			2.17			
C7x	C_s	2.54	2.17			2.54*			
C7y	C_s	3.46	2.96			i. f.			
C8a	C_{4v}	0.10	0.00			0.23	0.00		
C8b	C_2	0.04	0.01			0.00	0.02		
C8c	C_s	0.00	0.02			0.14	0.02		
C8d	C_2	0.02	0.02			i. f.			
C8e	C_1	0.29	0.22			0.31			
C8f	C_s	0.21	0.23			0.33			
C8g	C_1	0.27	0.25			0.38			
C8h	C_s	0.24	0.26			0.38			
C8i	C_1	0.37	0.44			0.42			
C8j	C_2	0.42	0.49			0.48			
C8k	C_1	0.70	0.53			0.75			
C8l	C_s	0.56	0.54			i. f.			
C8m	C_s	0.77	0.59			0.75			
C8n	C_s	0.40	0.61			i. f.			
C8o	C_s	0.79	0.65			0.86			
C8p	C_s	0.49	0.67			i. f.			

1.2 Anionic Clusters

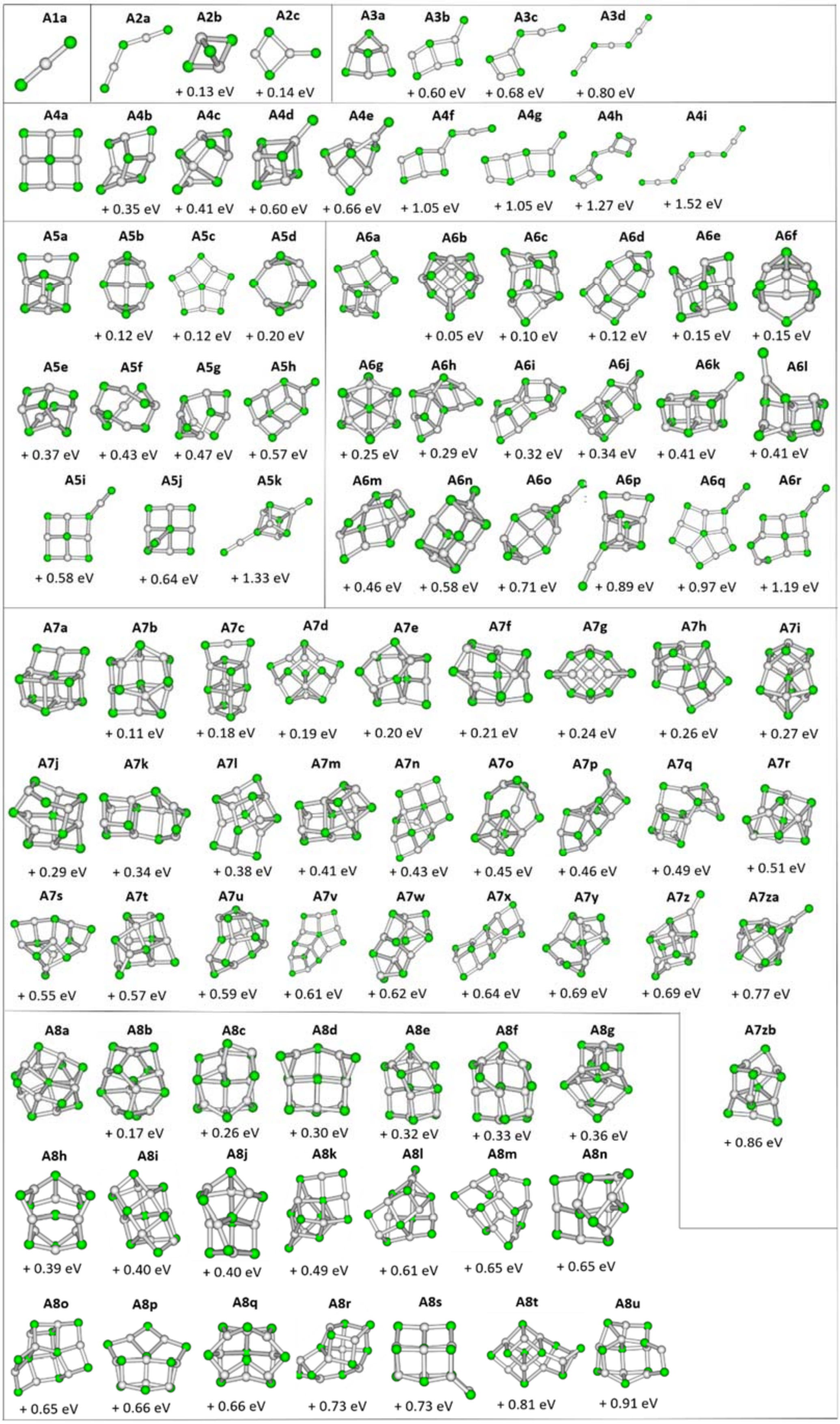

Figure S3: Overview of the lowest-energy isomers with relative energies calculated at the CCSD/DZ//B3LYP-D3 level of theory for the anionic clusters $(NaCl)_xNa^+$ with x = 1–8.

Table S2: Overview of the lowest-energy isomers including symmetry groups and relative energies of $(NaCl)_xCl^-$. Isomers whose energy is marked with * have one imaginary frequency within the listed symmetry group and thus, the listed energy corresponds to a structure with lower symmetry. Imaginary frequency for given functional: i. f.

Isomer	Symmetry Group	B3LYP-D3/ aug-cc-pVDZ	CCSD/DZ// B3LYP-D3	CCSD/TZ// B3LYP-D3	CCSD(T)/TZ/ /B3LYP-D3	ωB97XD/ aug-cc-pVDZ	CCSD/DZ// ωB97XD	CCSD/TZ// ωB97XD	CCSD(T)/TZ/ /ωB97XD
A1a	$D_{\infty h}$	0.00	0.00	0.00	0.00	0.00		0.00	0.00
A2a	C_{2v}	0.00	0.00	0.00	0.00	0.00		0.00	0.00
A2b	D_{3h}	0.15	0.13	0.11	0.09	0.05		0.14	0.12
A2c	C_{2v}	0.13	0.14	0.15	0.14	0.11		0.17	0.16
A3a	C_{3v}	0.00	0.00	0.00	0.00	0.00		0.00	0.00
A3b	C_s	0.58	0.60	0.59	0.60	0.64		0.58	0.59
A3c	C_s	0.68	0.68	0.66	0.68	0.72		0.64	0.65
A3d	C_{2h}	0.80	0.80	0.77	0.80	0.87		0.73	0.75
A4a	C_{4v}	0.00	0.00	0.00	0.00	0.00		0.00	0.00
A4b	C_2	0.27	0.35	0.34	0.33	0.31		0.35	0.34
A4c	C_s	0.28	0.41	0.39	0.38	0.28		0.37	0.35
A4d	C_{3v}	0.47	0.60	0.63	0.62	0.51		0.63	0.62
A4e	C_s	0.62	0.66	0.67	0.66	0.61		0.68	0.68
A4f	C_s	1.09	1.05	1.02	1.03	1.07*		1.01*	1.02*
A4g	C_s	1.05	1.05	1.03	1.04	1.05*		1.02*	1.03*
A4h	C_s	1.31	1.27	1.24	1.25	1.27*		1.23*	1.24*
A4i	C_{2v}	1.58	1.52	1.48	1.51	1.59		1.44	1.47
A5a	C_{2v}	0.00	0.00	0.00	0.00	0.00		0.00	0.00
A5b	C_{2v}	0.21	0.12	0.09	0.11	0.20		0.06	0.08
A5c	D_{5h}	0.21	0.12	0.10	0.14	0.28		0.09	0.13
A5d	C_s	0.25	0.20	0.17	0.19	i. f.			
A5e	C_1	0.28	0.37	0.35	0.35	0.35		0.33	0.32
A5f	D_3	0.29	0.43	0.42	0.41	0.29		0.32	0.30
A5g	C_1	0.40	0.47	0.44	0.43	0.34		0.39	0.38
A5h	C_s	0.63	0.57	0.55	0.58	0.70		0.53	0.56
A5i	C_s	0.73	0.58	0.54	0.58	0.75		0.51	0.54
A5j	C_s	0.71	0.64	0.62	0.65	0.77		0.62	0.65
A5k	C_{3v}	1.32	1.33	1.30	1.33	1.40		1.28	1.30
A6a	C_s	0.07	0.00	0.00	0.00	0.05	0.00	0.00	0.00
A6b	C_{2v}	0.00	0.05	0.04	0.03	0.09	0.06	0.05	0.04
A6c	C_s	0.02	0.10	0.09	0.07	0.00	0.09	0.09	0.08
A6d	C_s	0.30	0.12	0.08	0.09	0.20		0.08	0.08
A6e	C_1	0.05	0.15	0.13	0.11	0.03		0.13	0.10
A6f	C_{3v}	0.03	0.15	0.14	0.12	0.09		0.10	0.08
A6g	D_{3d}	0.22	0.25	0.25	0.19	0.12		0.26	0.21
A6h	C_2	0.28	0.29	0.26	0.24	0.19		0.27	0.25
A6i	C_1	0.32	0.32	0.29	0.29	0.27		0.24	0.24
A6j	C_s	0.33	0.34	0.32	0.30	0.27		0.30	0.28
A6k	C_s	0.36	0.41	0.42	0.41	0.37		0.42	0.40
A6l	C_1	0.40	0.41	0.42	0.41	i. f.			
A6m	C_1	0.41	0.46	0.44	0.42	0.37		0.43	0.41
A6n	C_s	0.50	0.58	0.58	0.58	i. f.			
A6o	C_s	0.83	0.71	0.69	0.69	0.74		0.68	0.68
A6p	C_s	0.88	0.89	0.87	0.87	0.84		0.85	0.84
A6q	C_s	1.10	0.97	0.93	0.96	1.07*		0.89*	0.92*
A6r	C_1	1.31	1.19	1.14	1.16	i. f.			

Isomer	Symmetry Group	B3LYP-D3/ aug-cc-pVDZ	CCSD/DZ// B3LYP-D3	CCSD/TZ// B3LYP-D3	CCSD(T)/TZ/ /B3LYP-D3	ωB97XD/ aug-cc-pVDZ	CCSD/DZ// ωB97XD	CCSD/TZ// ωB97XD	CCSD(T)/TZ/ /ωB97XD
A7a	C_{2v}	0.00	0.00			0.00			
A7b	C_1	0.09	0.11			0.07			
A7c	C_{2v}	0.23	0.18			0.12			
A7d	C_s	0.11	0.19			0.08*			
A7e	C_1	0.11	0.20			i. f.			
A7f	C_1	0.17	0.21			0.08			
A7g	C_{2v}	0.24	0.24			i. f.			
A7h	C_1	0.28	0.26			0.24			
A7i	C_{3v}	0.23	0.27			0.16			
A7j	C_s	0.23	0.29			0.25			
A7k	C_s	0.49	0.34			0.34			
A7l	C_1	0.32	0.38			i. f.			
A7m	C_s	0.40	0.41			i. f.			
A7n	C_s	0.55	0.43			0.48			
A7o	C_s	0.41	0.45			0.30			
A7p	C_1	0.62	0.46			0.45			
A7q	C_1	0.52	0.49			0.48			
A7r	C_1	0.48	0.51			0.41			
A7s	C_1	0.56	0.55			0.52			
A7t	C_1	0.53	0.57			0.44			
A7u	C_1	0.56	0.59			0.54			
A7v	C_1	0.92	0.61			0.75			
A7w	C_s	0.62	0.62			0.58			
A7x	C_1	0.77	0.64			0.60			
A7y	C_1	0.62	0.69			0.67			
A7z	C_{2v}	0.73	0.69			0.78			
A7za	C_s	0.78	0.77			0.78			
A7zb	C_s	0.83	0.86			0.84			
A8a	D_{4d}	0.00	0.00			0.00			
A8b	S_4	0.02	0.17			0.14			
A8c	C_1	0.17	0.26			0.29			
A8d	C_s	0.27	0.30			0.29			
A8e	C_s	0.25	0.32			0.31			
A8f	C_1	0.24	0.33			0.36			
A8g	C_s	0.35	0.36			0.37			
A8h	C_s	0.29	0.39			0.45			
A8i	C_1	0.45	0.40			0.50			
A8j	C_1	0.39	0.40			0.31			
A8k	C_{2v}	0.58	0.49			0.57			
A8l	C_1	0.60	0.61			0.58			
A8m	C_2	0.55	0.65			0.56			
A8n	C_1	0.66	0.65			0.57			
A8o	C_1	0.80	0.65			0.85			
A8p	C_{2v}	0.71	0.66			0.78			
A8q	C_s	0.50	0.66			0.63			
A8r	C_1	0.77	0.73			i. f.			
A8s	C_1	0.82	0.73			0.89			
A8t	C_1	0.85	0.81			0.88			
A8u	C_1	1.00	0.91			1.05			

1.3 Neutral Clusters

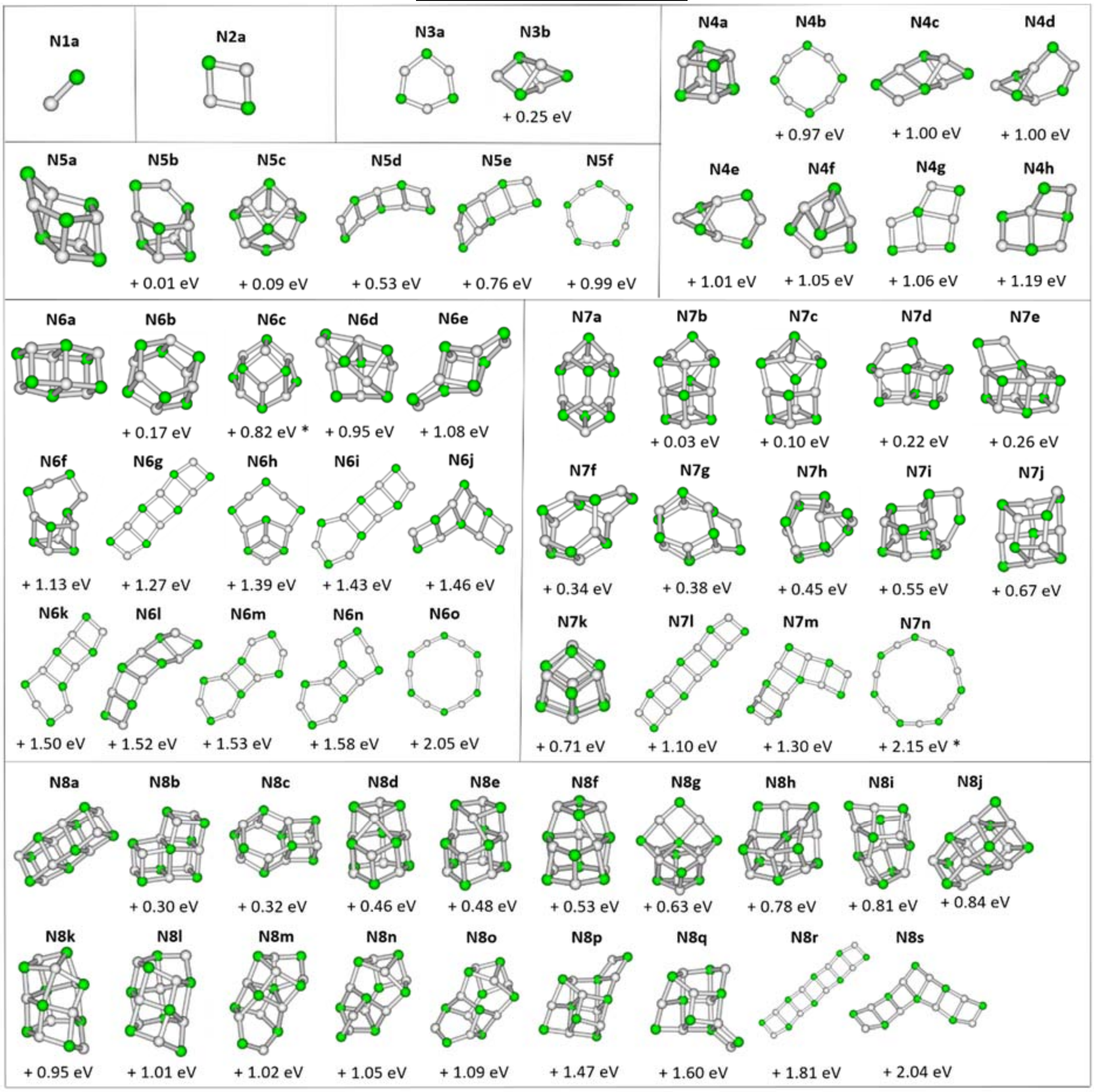

Figure S4: Overview of the lowest energy isomers with relative energies calculated at the CCSD/DZ//B3LYP-D3 level of theory for the neutral clusters $(NaCl)_x$ with x = 1–8. Energies marked with * are obtained by using the ωB97XD functional for the geometrical optimization due to the presence of an imaginary frequency using the B3LYP-D3 functional.

Table S3: Overview of the lowest energy isomers including symmetry groups and relative energies of $(NaCl)_x$. Isomers whose energy is marked with * have one imaginary frequency within the listed symmetry group and thus, the listed energy corresponds to a structure with lower symmetry. Imaginary frequency for given functional: i. f.

**Isomer**	**Symmetry Group**	**B3LYP-D3/ aug-cc-pVDZ**	**CCSD/DZ// B3LYP-D3**	**CCSD/TZ// B3LYP-D3**	**CCSD(T)/TZ/ /B3LYP-D3**	***ω*B97XD/ aug-cc-pVDZ**	**CCSD/DZ// *ω*B97XD**	**CCSD/TZ// *ω*B97XD**	**CCSD(T)/TZ/ /*ω*B97XD**
N1a	$C_{\infty v}$	0.00	0.00	0.00	0.00	0.00		0.00	0.00
N2a	D_{2h}	0.00	0.00	0.00	0.00	0.00		0.00	0.00
N3a	D_{3h}	0.00	0.00	0.00	0.00	0.00		0.00	0.00
N3b	C_{2v}	0.27	0.25	0.26	0.24	0.14		0.28	0.26
N4a	T_d	0.00	0.00	0.00	0.00	0.00		0.00	0.00
N4b	D_{4h}	0.97	0.97	0.90	0.94	1.09		0.82	0.86
N4c	C_s	0.97	1.00	0.95	0.95	0.96		0.89	0.90
N4d	C_s	0.97	1.00	0.96	0.97	0.95		0.89	0.90
N4e	C_{2v}	0.97	1.01	0.98	0.99	1.02		0.93	0.94
N4f	C_s	0.98	1.05	1.02	1.02	i. f.			
N4g	C_{2v}	1.00	1.06	1.00	1.03	1.09		0.94	0.96
N4h	C_2	1.09	1.19	1.13	1.15	i. f.			
N5a	C_s	0.00	0.00	0.00	0.00	0.00		0.01	0.00
N5b	C_s	0.02	0.01	0.00	0.01	0.06*		0.00*	0.00*
N5c	C_{2v}	0.04	0.09	0.10	0.09	0.01		0.10	0.08
N5d	C_s	0.73	0.53	0.48	0.50	0.62		0.44	0.46
N5e	C_1	0.87	0.76	0.72	0.73	0.76		0.68	0.68
N5f	D_{5h}	1.19	0.99	0.91	0.96	1.19		0.86	0.90
N6a	D_{2h}	0.00	0.00			0.00	0.00		
N6b	D_{3d}	0.14	0.17			0.25	0.13		
N6c	C_1	i. f.				0.88	0.82		
N6d	C_1	0.84	0.95			0.81			
N6e	C_s	1.00	1.08			1.16			
N6f	C_s	1.12	1.13			1.18			
N6g	C_2	1.53	1.27			1.40			
N6h	C_1	1.40	1.39			1.47			
N6i	C_s	1.62	1.43			i. f.			
N6j	C_2	1.65	1.46			1.58			
N6k	C_s	1.66	1.50			1.63			
N6l	C_1	1.67	1.52			1.55			
N6m	C_s	1.66	1.53			i. f.			
N6n	C_s	1.68	1.58			1.74			
N6o	D_{6h}	2.34	2.05			2.30			
N7a	C_{3v}	0.01	0.00			0.08	0.00		
N7b	C_s	0.00	0.03			0.00	0.06		
N7c	C_s	0.00	0.10			0.00	0.13		
N7d	C_s	0.24	0.22			0.24			
N7e	C_s	0.26	0.26			0.26			
N7f	C_s	0.32	0.34			i. f.			
N7g	C_1	0.43	0.38			i. f.			
N7h	C_s	0.35	0.45			0.39			
N7i	C_s	0.48	0.55			0.54			
N7j	C_1	0.52	0.67			0.54			
N7k	C_{3v}	0.41	0.71			0.29			

Isomer	Symmetry Group	B3LYP-D3/ aug-cc-pVDZ	CCSD/DZ// B3LYP-D3	CCSD/TZ// B3LYP-D3	CCSD(T)/TZ/ /B3LYP-D3	ωB97XD/ aug-cc-pVDZ	CCSD/DZ// ωB97XD	CCSD/TZ// ωB97XD	CCSD(T)/TZ/ /ωB97XD
N7l	C_{2v}	1.50	1.10			1.25			
N7m	C_1	1.62	1.30			1.42			
N7n	C_s					2.49			
N8a	D_{2d}	0.00	0.00			0.00			
N8b	C_s	0.17	0.30			0.27			
N8c	C_2	0.25	0.32			i. f.			
N8d	C_s	0.35	0.46			0.48			
N8e	C_1	0.36	0.48			0.47			
N8f	C_1	0.41	0.53			0.53			
N8g	C_{2v}	0.53	0.63			0.59			
N8h	C_s	0.61	0.78			0.74			
N8i	C_1	0.65	0.81			i. f.			
N8j	C_s	0.64	0.84			0.74			
N8k	C_1	0.71	0.95			i. f.			
N8l	C_1	0.81	1.01			0.90			
N8m	C_1	0.87	1.02			1.04			
N8n	C_s	0.82	1.05			1.01			
N8o	C_1	0.87	1.09			1.06			
N8p	C_1	1.32	1.47			1.40			
N8q	C_1	1.38	1.60			1.51			
N8r	C_{2h}	2.24	1.81			2.01*			
N8s	C_2	2.36	2.04			2.21			

2. Cluster Stability and Dissociation Energies

2.1 Dissociation of NaCl and the Atomic Ion

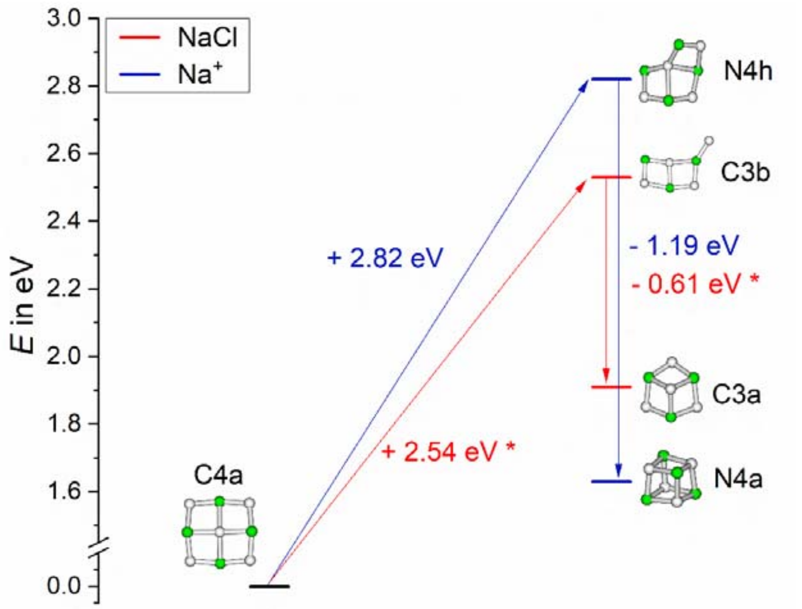

Figure S5: Energy scheme for the cationic magic cluster $(NaCl)_4Na^+$ when no isomerization before fragmentation is allowed. Thus, dissociation of NaCl or Na^+ occurs directly from the minimum energy structure C4a and proceeds via a higher lying isomer of the product. Given energies are calculated at the CCSD/DZ//B3LYP-D3 level of theory. Energies marked with * are obtained by using the ωB97XD functional for the geometrical optimization due to the presence of an imaginary frequency using the B3LYP-D3 functional.

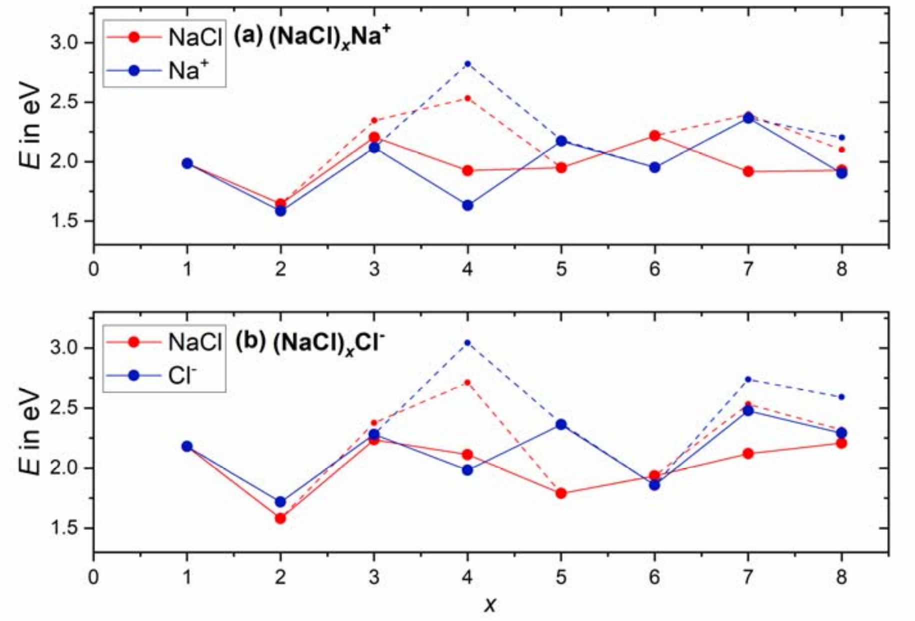

Figure S6: Energy needed to remove NaCl or the respective atomic ion (Cl^- and Na^+) from the ionic clusters $(NaCl)_xCl^-$ (a) and $(NaCl)_xNa^+$ (b). Solid lines, large symbols: isomerization before dissociation and thus, fragmentation into lowest lying product isomers, see Figure 3 for x = 4. Dashed lines, small symbols: no isomerization before dissociation, resulting in higher lying product isomers, see Figure S5 for x = 4. Given energies are calculated at the CCSD/DZ//B3LYP-D3 level of theory.

2.1 Benchmarking Dissociation Energies

The dissociation energies of the loss channels described by equation (2), (3) and (4) are plotted in Figure S7. All Methods used in Table S4, Figure S7 and Figure 4 provide similar results. The dissociation energies calculated with CCSD(T)/TZ//B3LYP-D3 (Figure S7: lower-left) and CCSD(T)/TZ//ωB97XD (Figure S7: lower-right) are almost identical, reflecting the insensitivity of the results to the method employed. For CCSD(T), only ions up to x = 6 and neutrals up to x = 5 are calculated.

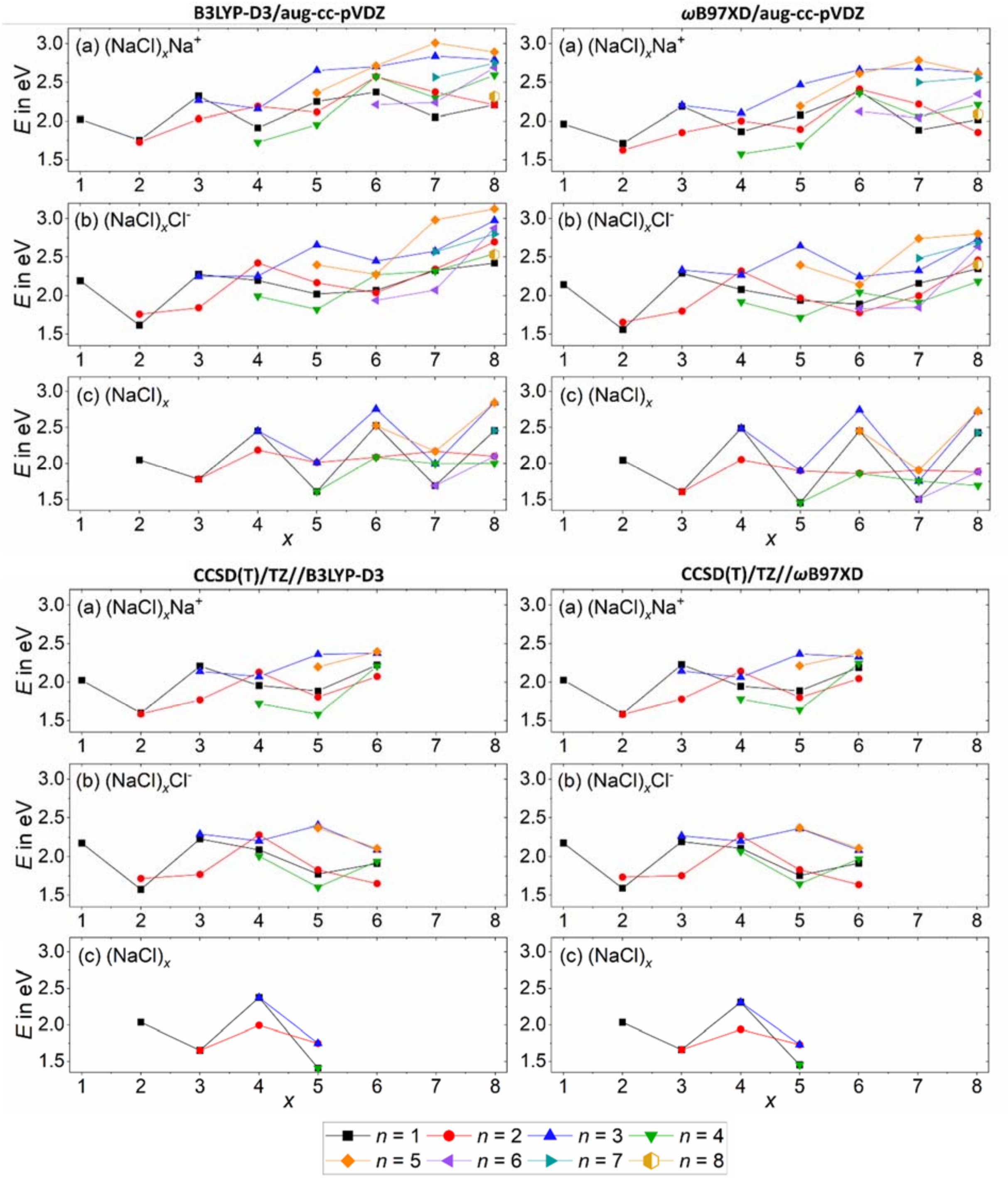

Figure S7: Dissociation energies of the a) cations, reaction (2); b) anions, reaction (3) and c) neutrals, reaction (4) as a function of cluster size x. Dissociation from the lowest energy isomer of the precursor to the lowest energy isomer of the products is assumed, including isomerization of the precursor. Calculations are performed at the B3LYP-D3/aug-cc-pVDZ (upper-left), the ωB97XD/aug-cc-pVDZ

(upper-right), the CCSD(T)/TZ//B3LYP-D3 (lower-left) and the CCSD(T)/TZ//ωB97XD (lower-right) level of theory.

Table S4: Dissociation energies for the loss channels (2), (3) and (4) of the cationic, anionic and neutral clusters, respectively. The energies are calculated by considering the minimum energy structures of reactant and products.

Charge	*x*	*n*	B3LYP-D3/ aug-cc-pVDZ	CCSD/DZ// B3LYP-D3	CCSD/TZ// B3LYP-D3	CCSD(T)/TZ/ /B3LYP-D3	ωB97XD/ aug-cc-pVDZ	CCSD/TZ// ωB97XD	CCSD(T)/TZ/ /ωB97XD
$(NaCl)_xNa^+$	1	1	2.02	1.98	2.03	2.03	1.96	2.03	2.03
	2	1	1.75	1.64	1.58	1.60	1.71	1.57	1.59
		2	1.73	1.58	1.58	1.58	1.63	1.57	1.58
	3	1	2.32	2.21	2.21	2.21	2.19	2.23	2.23
		2	2.03	1.81	1.75	1.77	1.85	1.76	1.78
		3	2.27	2.12	2.13	2.14	2.20	2.13	2.14
	4	1	1.91	1.92	1.96	1.96	1.86	1.95	1.95
		2	2.19	2.09	2.13	2.13	2.00	2.14	2.14
		3	2.16	2.06	2.05	2.07	2.10	2.04	2.06
		4	1.73	1.63	1.74	1.72	1.58	1.80	1.78
	5	1	2.25	1.95	1.86	1.89	2.08	1.86	1.89
		2	2.11	1.83	1.78	1.81	1.89	1.77	1.80
		3	2.65	2.36	2.33	2.36	2.47	2.34	2.37
		4	1.95	1.60	1.57	1.58	1.69	1.62	1.64
		5	2.36	2.17	2.20	2.20	2.19	2.21	2.21
	6	1	2.37	2.22	2.19	2.22	2.38	2.15	2.19
		2	2.57	2.12	2.01	2.07	2.41	1.98	2.04
		3	2.70	2.38	2.31	2.38	2.66	2.26	2.33
		4	2.57	2.17	2.17	2.21	2.36	2.21	2.25
		5	2.71	2.41	2.35	2.40	2.61	2.33	2.38
		6	2.21	1.95			2.12		
	7	1	2.05	1.92			1.88		
		2	2.37	2.09			2.22		
		3	2.84	2.37			2.68		
		4	2.30	1.88			2.06		
		5	3.01	2.68			2.78		
		6	2.24	1.88			2.04		
		7	2.57	2.37			2.50		
	8	1	2.21	1.93			2.02		
		2	2.21	1.80			1.85		
		3	2.79	2.35			2.62		

Charge	*x*	*n*	B3LYP-D3/ aug-cc-pVDZ	CCSD/DZ// B3LYP-D3	CCSD/TZ// B3LYP-D3	CCSD(T)/TZ/ /B3LYP-D3	ωB97XD/ aug-cc-pVDZ	CCSD/TZ// ωB97XD	CCSD(T)/TZ/ /ωB97XD
		4	2.59	1.88			2.21		
		5	2.89	2.40			2.62		
		6	2.69	2.17			2.35		
		7	2.75	2.31			2.56		
		8	2.31	1.90			2.09		
$(NaCl)_xCl^-$	1	1	2.19	2.18	2.18	2.18	2.14	2.18	2.18
	2	1	1.62	1.58	1.58	1.58	1.56	1.59	1.59
		2	1.76	1.72	1.73	1.72	1.65	1.74	1.74
	3	1	2.28	2.24	2.21	2.23	2.29	2.17	2.20
		2	1.84	1.77	1.75	1.77	1.80	1.74	1.75
		3	2.25	2.28	2.27	2.29	2.33	2.25	2.27
	4	1	2.20	2.11	2.09	2.09	2.08	2.11	2.11
		2	2.42	2.30	2.26	2.28	2.32	2.25	2.27
		3	2.25	2.21	2.18	2.21	2.27	2.18	2.20
		4	1.99	1.98	2.02	2.01	1.92	2.08	2.07
	5	1	2.02	1.79	1.74	1.77	1.94	1.72	1.76
		2	2.17	1.86	1.80	1.83	1.97	1.80	1.83
		3	2.66	2.42	2.34	2.40	2.64	2.31	2.37
		4	1.82	1.59	1.58	1.60	1.71	1.62	1.65
		5	2.40	2.36	2.36	2.37	2.40	2.35	2.37
	6	1	2.07	1.94	1.92	1.91	1.89	1.92	1.91
		2	2.04	1.68	1.62	1.65	1.78	1.61	1.64
		3	2.45	2.12	2.05	2.09	2.24	2.05	2.09
		4	2.27	1.94	1.91	1.94	2.04	1.94	1.97
		5	2.27	2.12	2.09	2.11	2.14	2.09	2.11
		6	1.94	1.86			1.83		
	7	1	2.33	2.12			2.16		
		2	2.34	2.01			2.00		
		3	2.58	2.13			2.32		
		4	2.32	1.83			1.91		
		5	2.98	2.66			2.74		
		6	2.07	1.80			1.85		
		7	2.57	2.48			2.48		
	8	1	2.42	2.21			2.35		
		2	2.69	2.28			2.46		
		3	2.98	2.55			2.73		
		4	2.54	1.92			2.18		

Charge	x	n	B3LYP-D3/ aug-cc-pVDZ	CCSD/DZ// B3LYP-D3	CCSD/TZ// B3LYP-D3	CCSD(T)/TZ/ /B3LYP-D3	ωB97XD/ aug-cc-pVDZ	CCSD/TZ// ωB97XD	CCSD(T)/TZ/ /ωB97XD
		5	3.13	2.63			2.80		
		6	2.88	2.42			2.64		
		7	2.80	2.50			2.69		
		8	2.53	2.29			2.40		
**(NaCl)$_x$**	2	1	2.05	2.04	2.04	2.04	2.05	2.03	2.03
	3	1	1.79	1.67	1.66	1.65	1.61	1.67	1.66
		2	1.79	1.67	1.66	1.65	1.61	1.67	1.66
	4	1	2.45	2.41	2.34	2.38	2.49	2.28	2.31
		2	2.19	2.04	1.97	1.99	2.05	1.91	1.94
		3	2.45	2.41	2.34	2.38	2.49	2.28	2.31
	5	1	1.61	1.41	1.40	1.41	1.46	1.45	1.45
		2	2.02	1.78	1.71	1.75	1.90	1.70	1.73
		3	2.02	1.78	1.71	1.75	1.90	1.70	1.73
		4	1.61	1.41	1.40	1.41	1.46	1.45	1.45
	6	1	2.53	2.44			2.45		
		2	2.09	1.80			1.86		
		3	2.76	2.54			2.74		
		4	2.09	1.80			1.86		
		5	2.53	2.44			2.45		
	7	1	1.70	1.50			1.51		
		2	2.17	1.90			1.91		
		3	2.00	1.63			1.76		
		4	2.00	1.63			1.76		
		5	2.17	1.90			1.91		
		6	1.70	1.50			1.51		
	8	1	2.46	2.39			2.43		
		2	2.10	1.85			1.89		
		3	2.84	2.62			2.73		
		4	2.00	1.61			1.70		
		5	2.84	2.62			2.73		
		6	2.10	1.85			1.89		
		7	2.46	2.39			2.43		

3. Collision Induced Dissociation Experiments

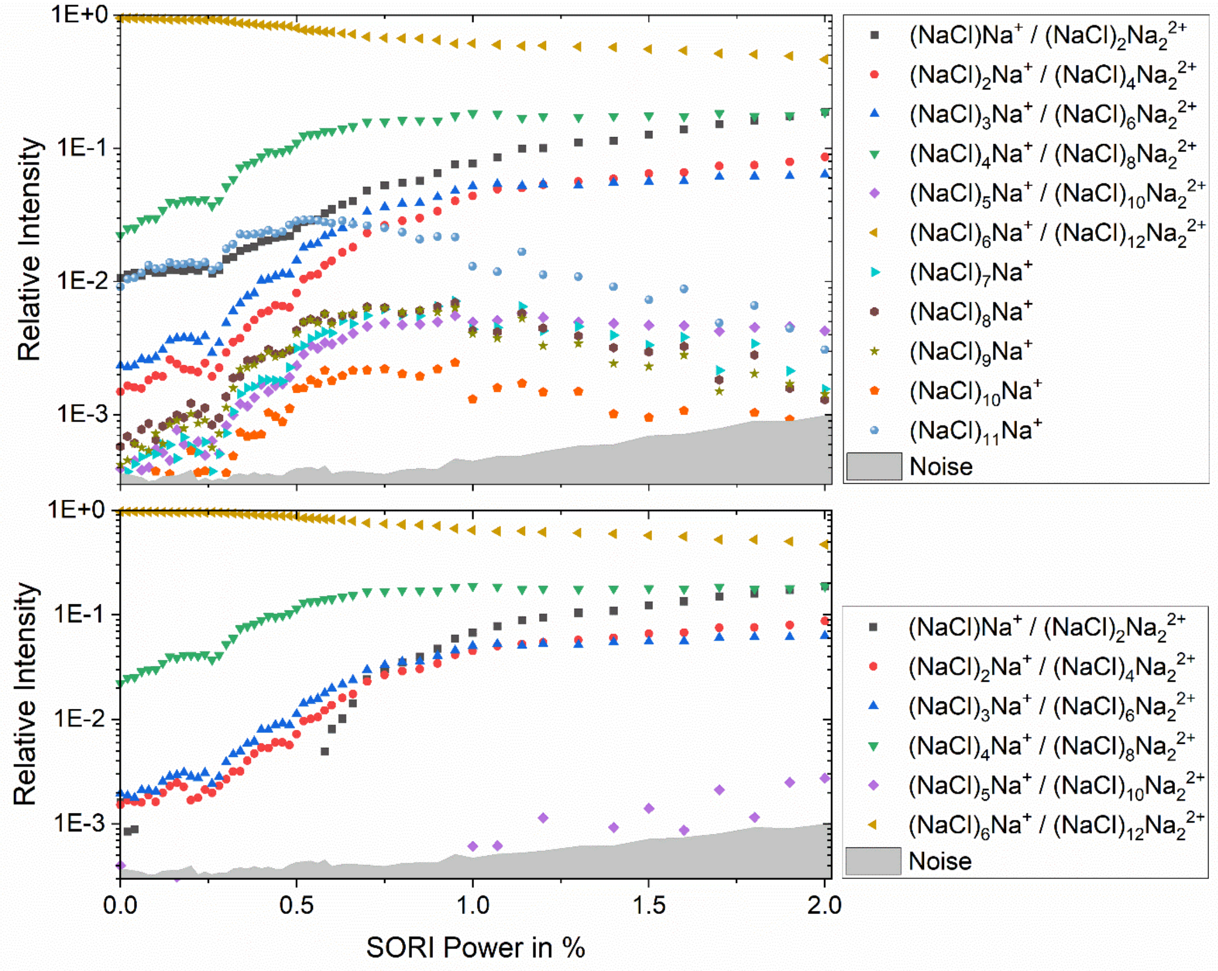

Figure S8: Due to an overlap of the precursor ion $(NaCl)_6Na^+$ with the doubly charged cluster $(NaCl)_{12}Na_2^{2+}$, a correction has been made in the CID spectrum of the cluster size x = 6 based on the intensities of the fragment peaks originating from the doubly charged species, which occur at higher mass to charge ratios than the precursor ion peak $(NaCl)_6Na^+$ / $(NaCl)_{12}Na_2^{2+}$. The loss of odd-numbered neutrals from $(NaCl)_{12}Na_2^{2+}$ is not observed, while the loss of even-numbered neutrals from $(NaCl)_{12}Na_2^{2+}$ would be overlapped with the fragment peaks of $(NaCl)_6Na^+$ and thus, cannot be ruled out. However, we assume main fragmentation of $(NaCl)_{12}Na_2^{2+}$ into two singly charged species, e.g. $(NaCl)_{12}Na_2^{2+} \longrightarrow (NaCl)_8Na^+ + (NaCl)_4Na^+$, whereby the intensity of the $(NaCl)_8Na^+$ peak should equal the intensity of the corresponding second product $(NaCl)_4Na^+$ originating from $(NaCl)_{12}Na_2^{2+}$. By subtracting the intensity of one product (e.g. $(NaCl)_8Na^+$) - originating from $(NaCl)_{12}Na_2^{2+}$ - from the corresponding second product peak (e.g. $(NaCl)_4Na^+$), we correct for the contribution of the doubly charged species. Since we cannot rule out the loss of even-numbered neutrals from $(NaCl)_{12}Na_2^{2+}$, the fragment peaks might be slightly overestimated, whereby the single contribution to each peak cannot be determined.

4. Statistical Analysis

4.1 DOS: Cations

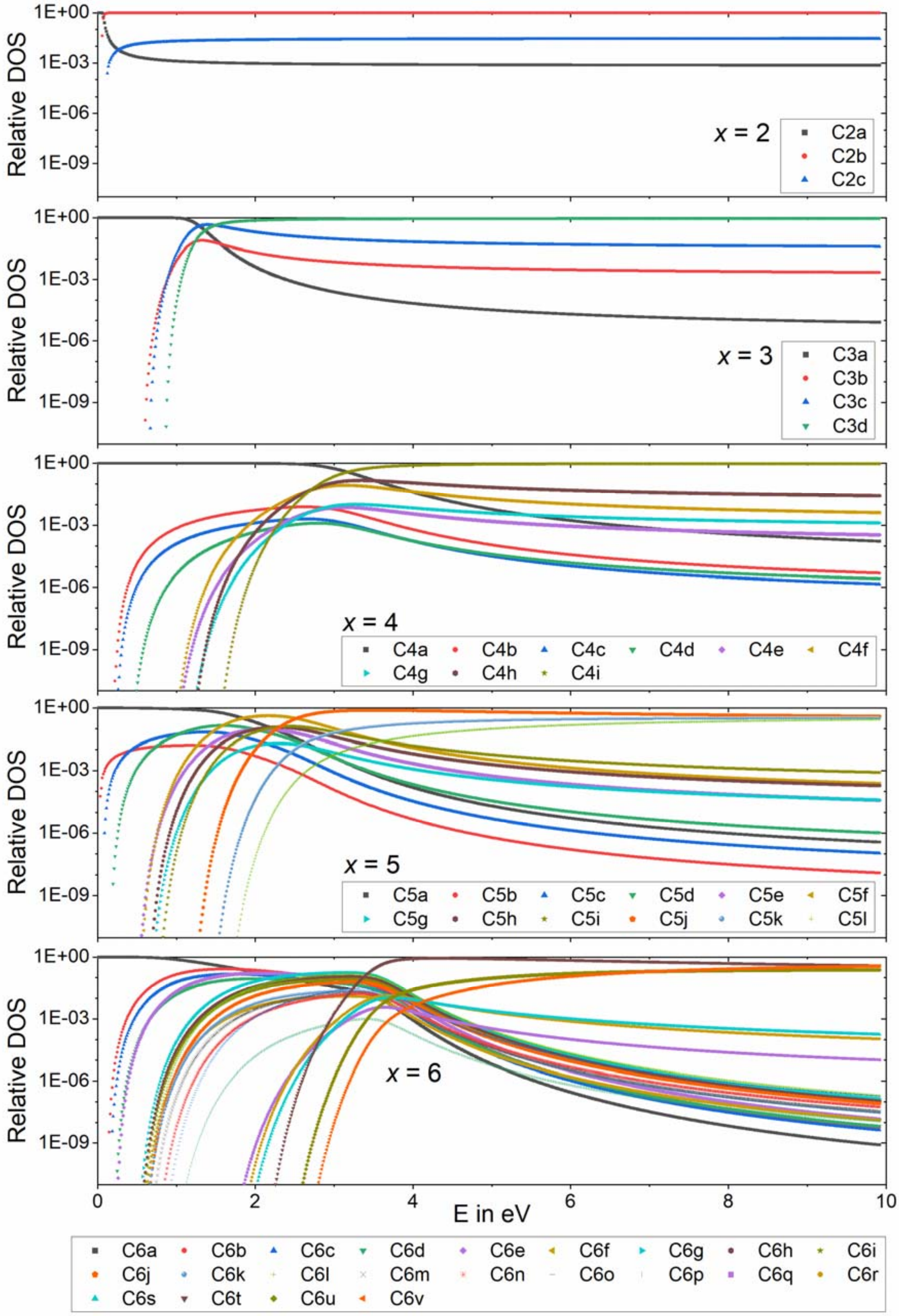

Figure S9: Relative DOS for the cationic clusters $(NaCl)_xNa^+$ with $x = 2$–6, calculated at the CCSD(T)/TZ//B3LYP-D3 level of theory. The isomers C3b, C4e and C4f have one imaginary frequency using the B3LYP-D3 functional and thus, the ωB97XD optimized frequencies and energies are used for an approximation of the DOS of these isomers.

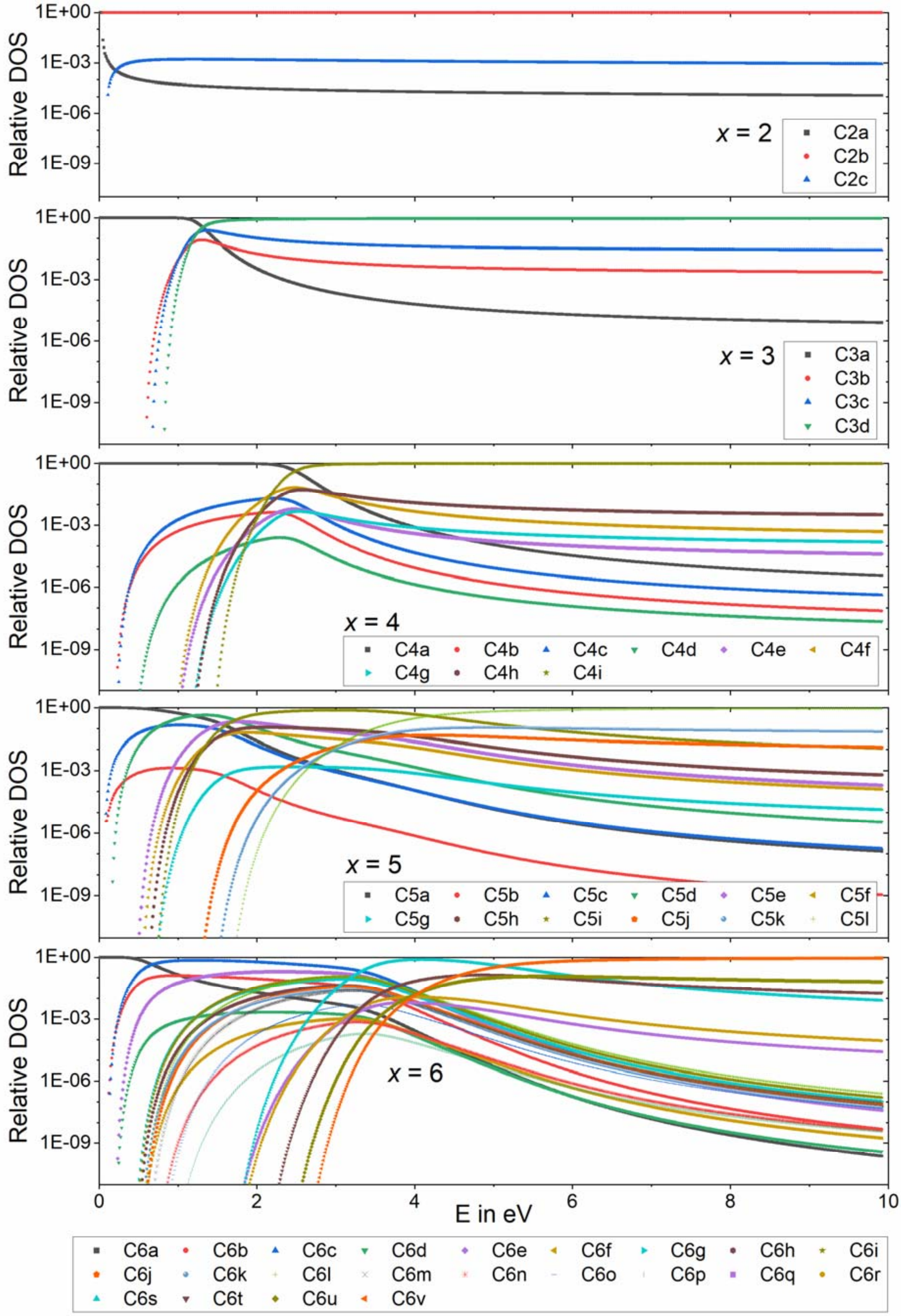

Figure S10: Relative DOS for the cationic clusters $(NaCl)_xNa^+$ with $x = 2–6$, calculated at the CCSD(T)/TZ//ωB97XD level of theory. The isomers C4g, C4h, C5d, C5h, C5l, C6m, C6q and C6v have one imaginary frequency using the ωB97XD functional and thus, the B3LYP-D3 optimized frequencies and energies are used for an approximation of the DOS of these isomers.

4.2 DOS: Anions

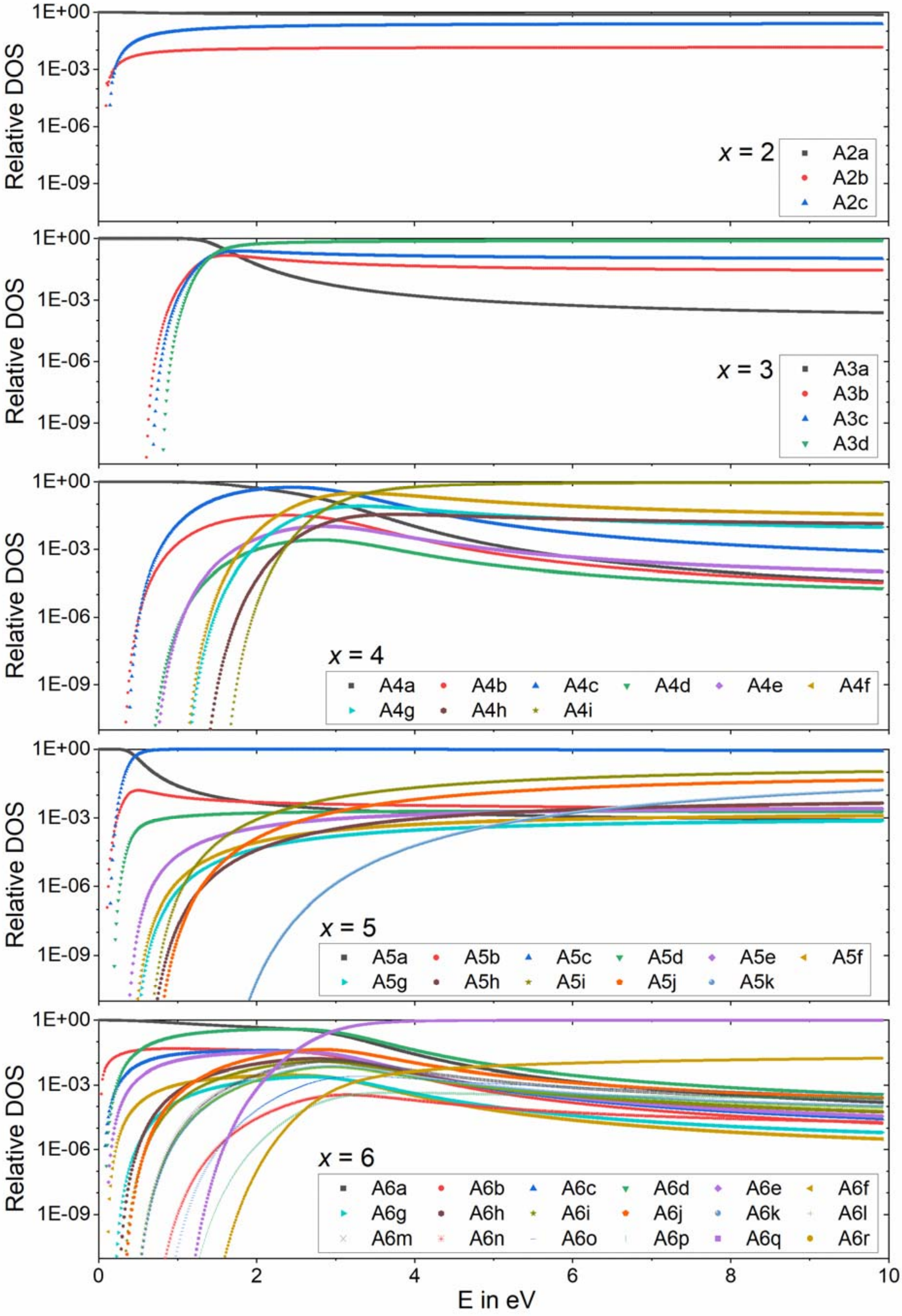

Figure S11: Relative DOS for the anionic clusters $(NaCl)_xCl^-$ with $x = 2–6$, calculated at the CCSD(T)/TZ//B3LYP-D3 level of theory.

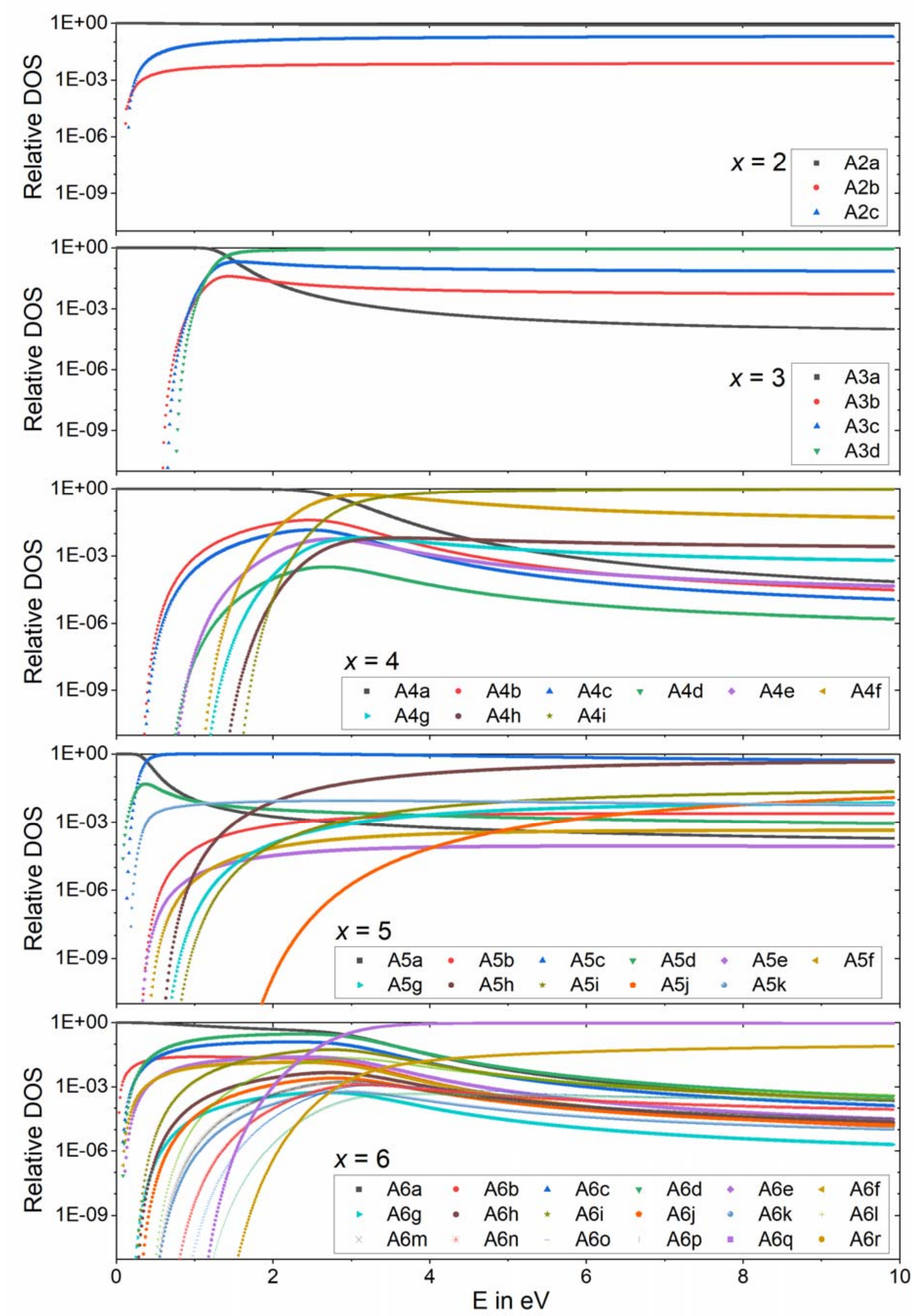

Figure S12: Relative DOS for the anionic clusters $(NaCl)_xCl^-$ with $x = 2–6$, calculated at the CCSD(T)/TZ//ωB97XD level of theory. The isomers A5d, A6l, A6n and A6r have one imaginary frequency using the ωB97XD functional and thus, the B3LYP-D3 optimized frequencies and energies are used for an approximation of the DOS of these isomers.

4.3 Benchmarking RRKM Rate Constants: Cations

Figures S13-S17 show the calculated RRKM rate constants $k^{+}_{x,n}$ for the loss channels (2) of the precursor ions $(NaCl)_xNa^+$ with $x = 3$–5 (a)–(c) and the total fragmentation rate constants $\sum_n k^{+}_{x,n}$ as sum of all loss channels n for cluster sizes $x = 3$–5 (d), calculated at different levels of theory. The results are similar for aug-cc-pVDZ and aug-cc-pVTZ basis sets, see Figures S13 and S14. Furthermore, single-point calculations employing CCSD and CCSD(T) also provide similar results, see Figures S14–S17. The only factor that causes slightly different results is the functional used for the frequency and geometry optimization, see again Figures S14–S17.

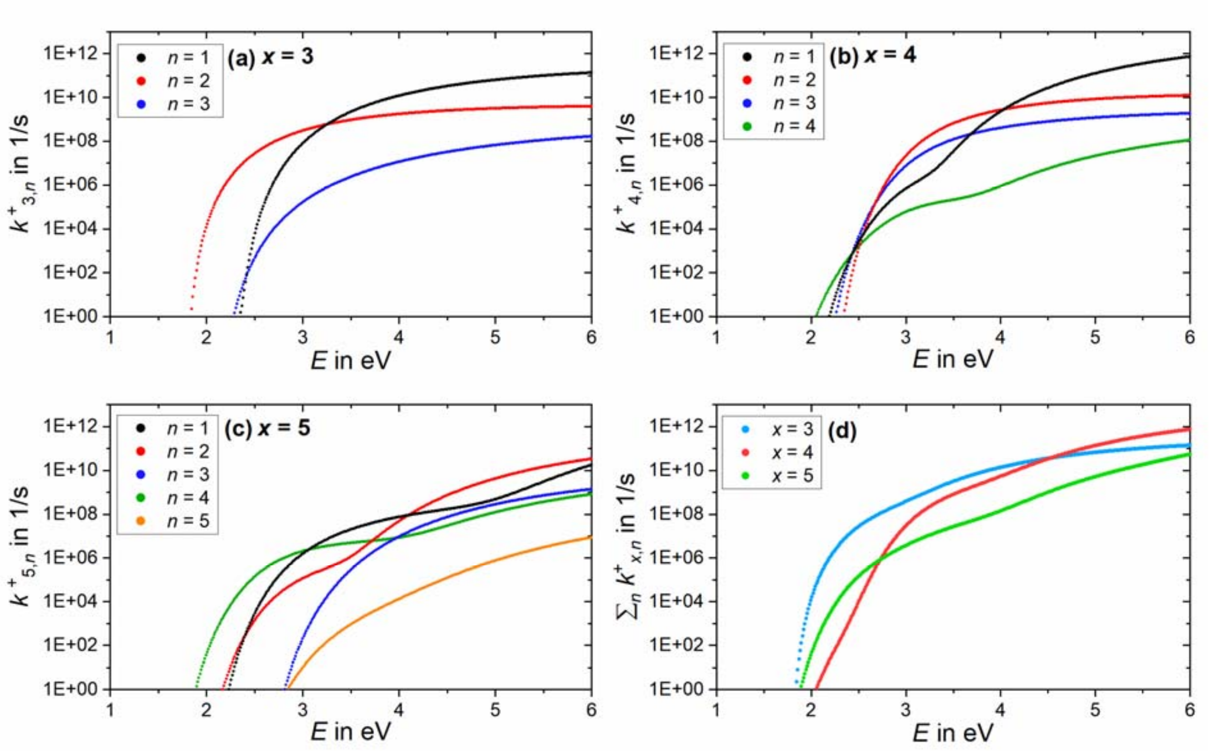

Figure S13: Method: CCSD/DZ//B3LYP-D3. (a)–(c) Calculated RRKM rate constants $k^{+}_{x,n}$ for the loss channels (2) of the precursor ions $(NaCl)_xNa^+$ with $x = 3$–5. (d) Total fragmentation rate constants $\sum_n k^{+}_{x,n}$ as sum of all loss channels n for cluster sizes $x = 3$–5.

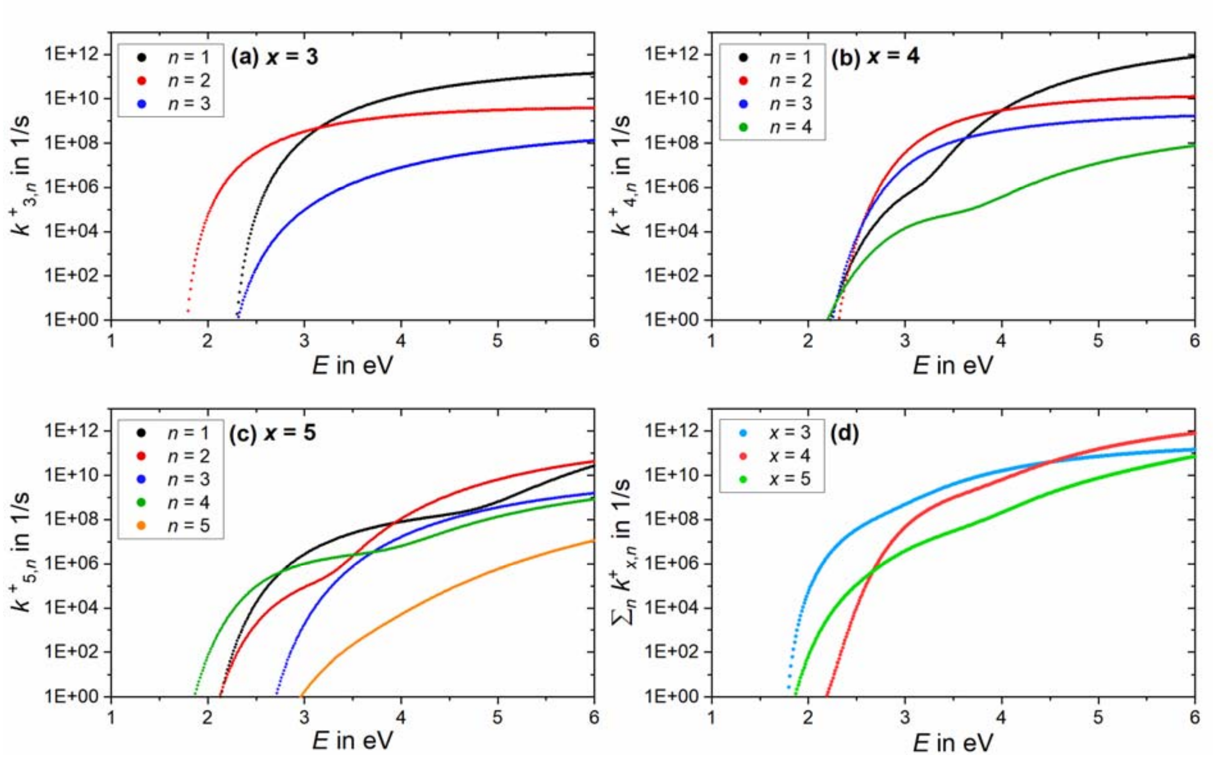

Figure S14: Method: CCSD/TZ//B3LYP-D3. (a)–(c) Calculated RRKM rate constants $k^{+}_{x,n}$ for the loss channels (2) of the precursor ions $(NaCl)_xNa^+$ with $x = 3–5$. (d) Total fragmentation rate constants $\sum_n k^{+}_{x,n}$ as sum of all loss channels n for cluster sizes $x = 3–5$.

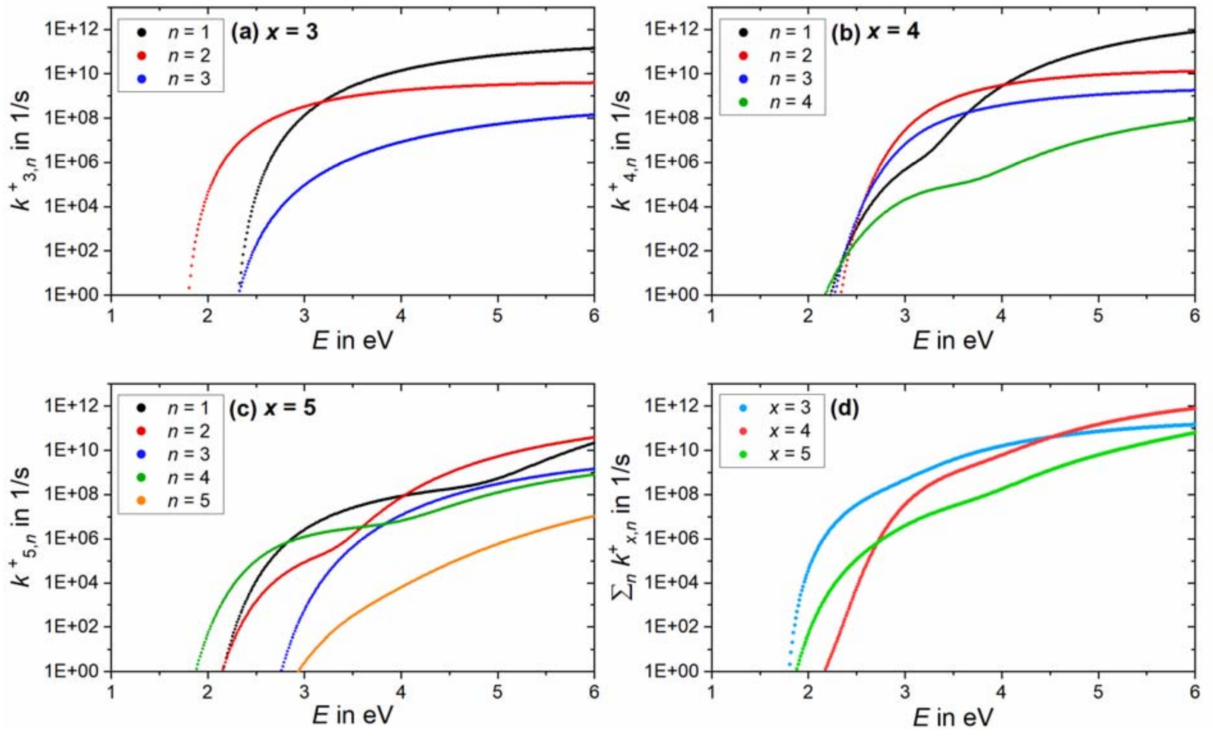

Figure S15: Method: CCSD(T)/TZ//B3LYP-D3. (a)–(c) Calculated RRKM rate constants $k^{+}_{x,n}$ for the loss channels (2) of the precursor ions $(NaCl)_xNa^+$ with $x = 3–5$. (d) Total fragmentation rate constants $\sum_n k^{+}_{x,n}$ as sum of all loss channels n for cluster sizes $x = 3–5$.

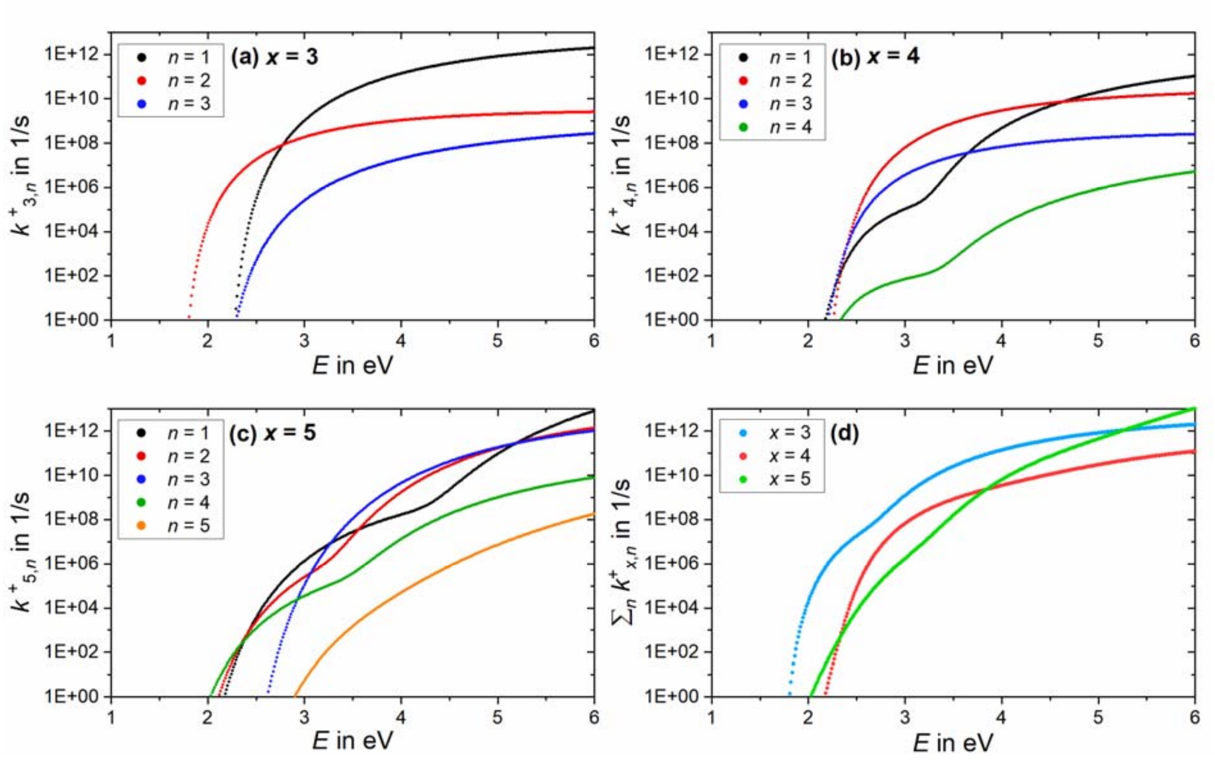

Figure S16: Method: CCSD/TZ//ωB97XD (a)–(c) Calculated RRKM rate constants $k^+_{x,n}$ for the loss channels (2) of the precursor ions $(NaCl)_xNa^+$ with x = 3–5. (d) Total fragmentation rate constants $\sum_n k^+_{x,n}$ as sum of all loss channels n for cluster sizes x = 3–5.

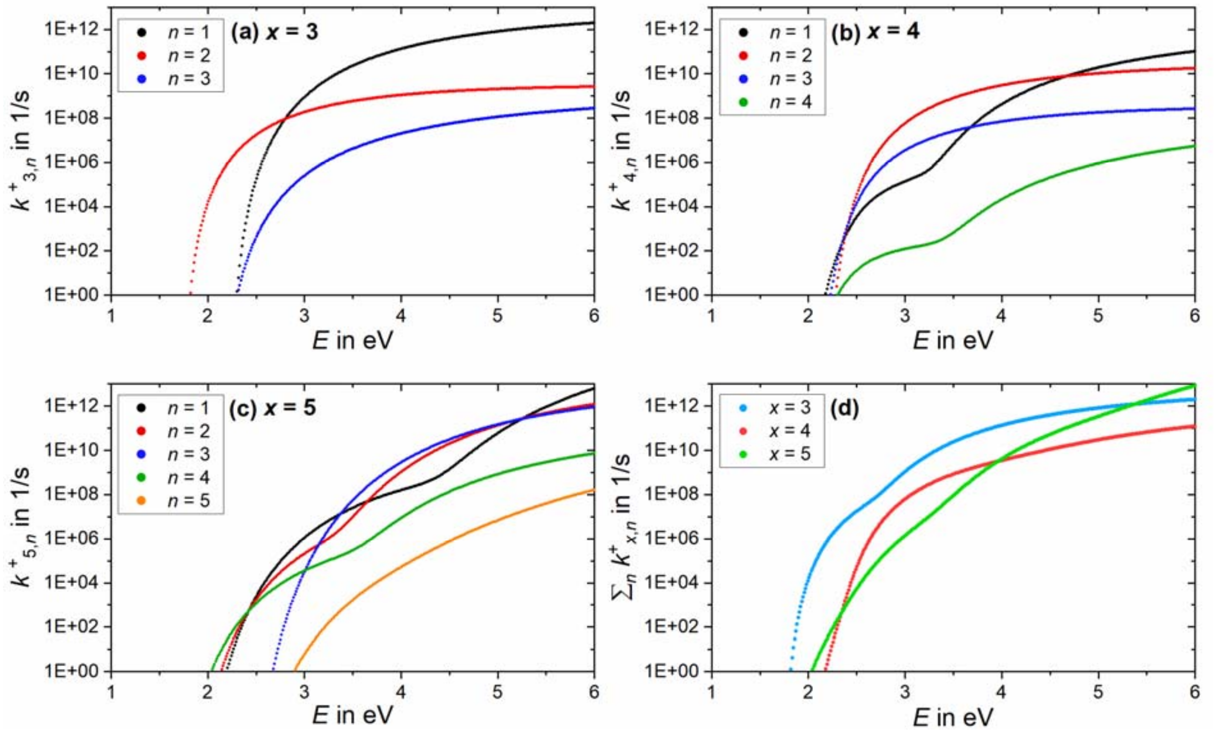

Figure S17: Method: CCSD(T)/TZ//ωB97XD. (a)–(c) Calculated RRKM rate constants $k^+_{x,n}$ for the loss channels (2) of the precursor ions $(NaCl)_xNa^+$ with x = 3–5. (d) Total fragmentation rate constants $\sum_n k^+_{x,n}$ as sum of all loss channels n for cluster sizes x = 3–5.

Figures S18-S22 show the calculated RRKM rate constants $k^{-}_{x,n}$ for the loss channels (3) of the precursor ions $(NaCl)_xNa^-$ with x = 3–5 (a)–(c) and the total fragmentation rate constants $\sum_n k^{-}_{x,n}$ as sum of all loss channels n for cluster sizes x = 3–5 (d), calculated at different levels of theory. The results are similar for the basis sets aug-cc-pVDZ and aug-cc-pVTZ, see Figures S18 and S19. Furthermore, single-point calculations employing CCSD and CCSD(T) also provide similar results, see Figures S19–S22. The only factor that causes slightly different results is the functional used for the frequency and geometry optimization, see again Figures S19–S22.

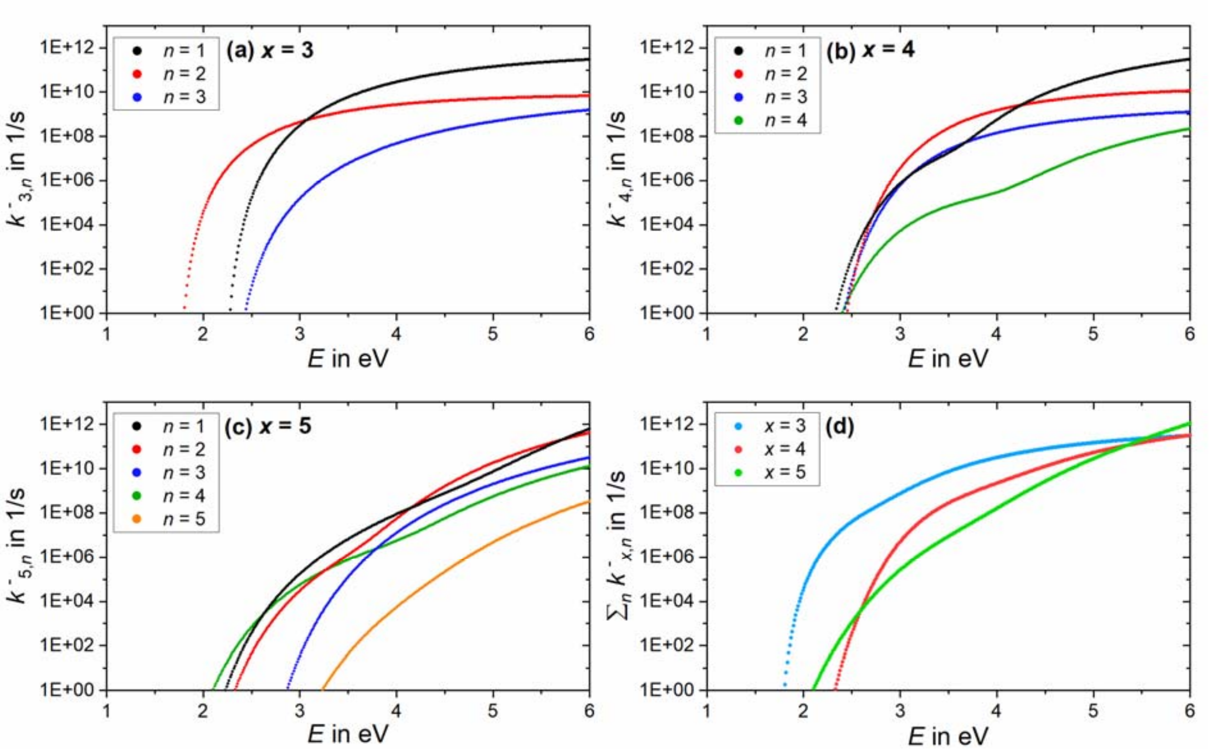

Figure S18: Method: CCSD/DZ//B3LYP-D3. (a)–(c) Calculated RRKM rate constants $k^{-}_{x,n}$ for the loss channels (3) of the precursor ions $(NaCl)_xCl^-$ with x = 3–5. (d) Total fragmentation rate constants $\sum_n k^{-}_{x,n}$ as sum of all loss channels n for cluster sizes x = 3–5.

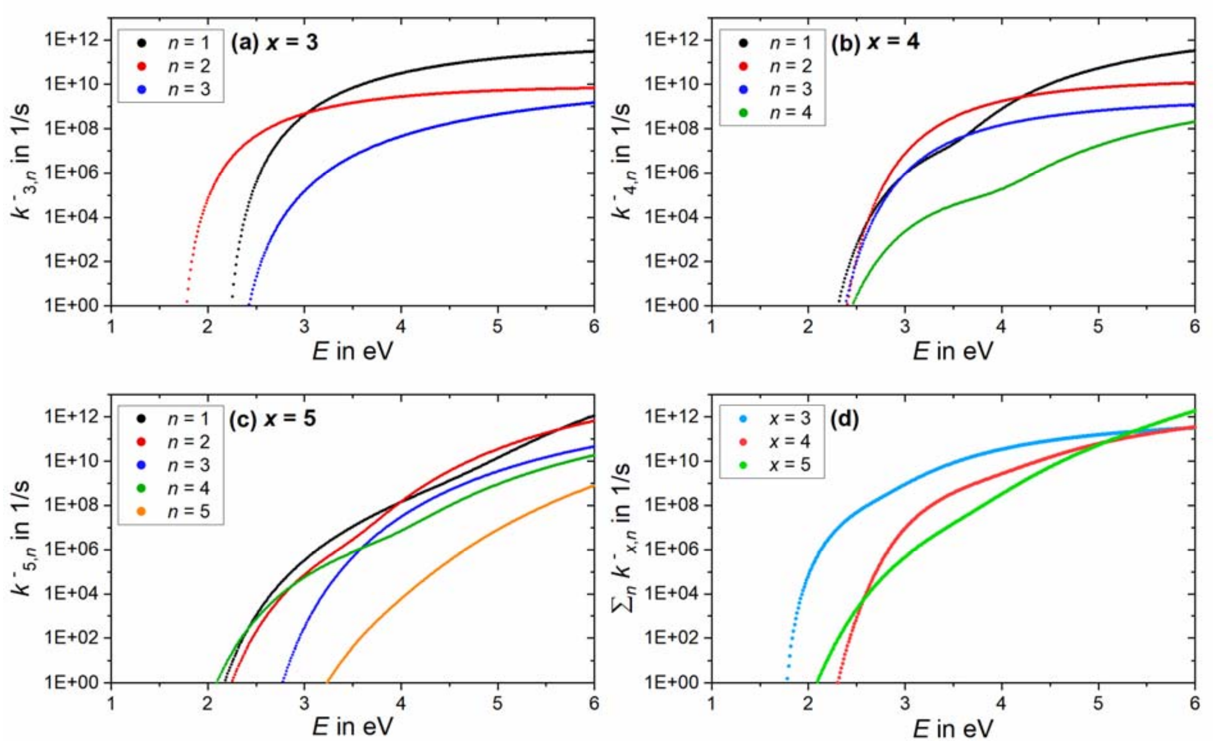

Figure S19: Method: CCSD/TZ//B3LYP-D3. (a)–(c) Calculated RRKM rate constants $k^-_{x,n}$ for the loss channels (3) of the precursor ions $(NaCl)_xCl^-$ with $x = 3$–5. (d) Total fragmentation rate constants $\sum_n k^-_{x,n}$ as sum of all loss channels n for cluster sizes $x = 3$–5.

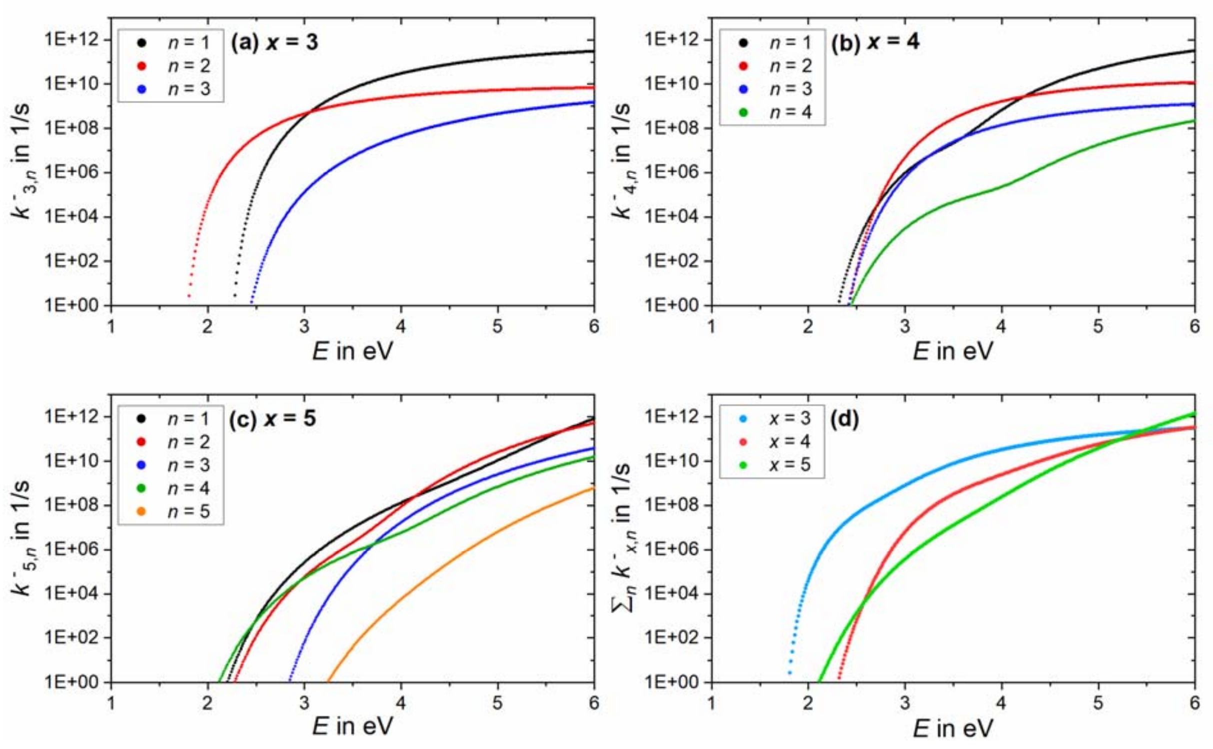

Figure S20: Method: CCSD(T)/TZ//B3LYP-D3. (a)–(c) Calculated RRKM rate constants $k^-_{x,n}$ for the loss channels (3) of the precursor ions $(NaCl)_xCl^-$ with $x = 3$–5. (d) Total fragmentation rate constants $\sum_n k^-_{x,n}$ as sum of all loss channels n for cluster sizes $x = 3$–5.

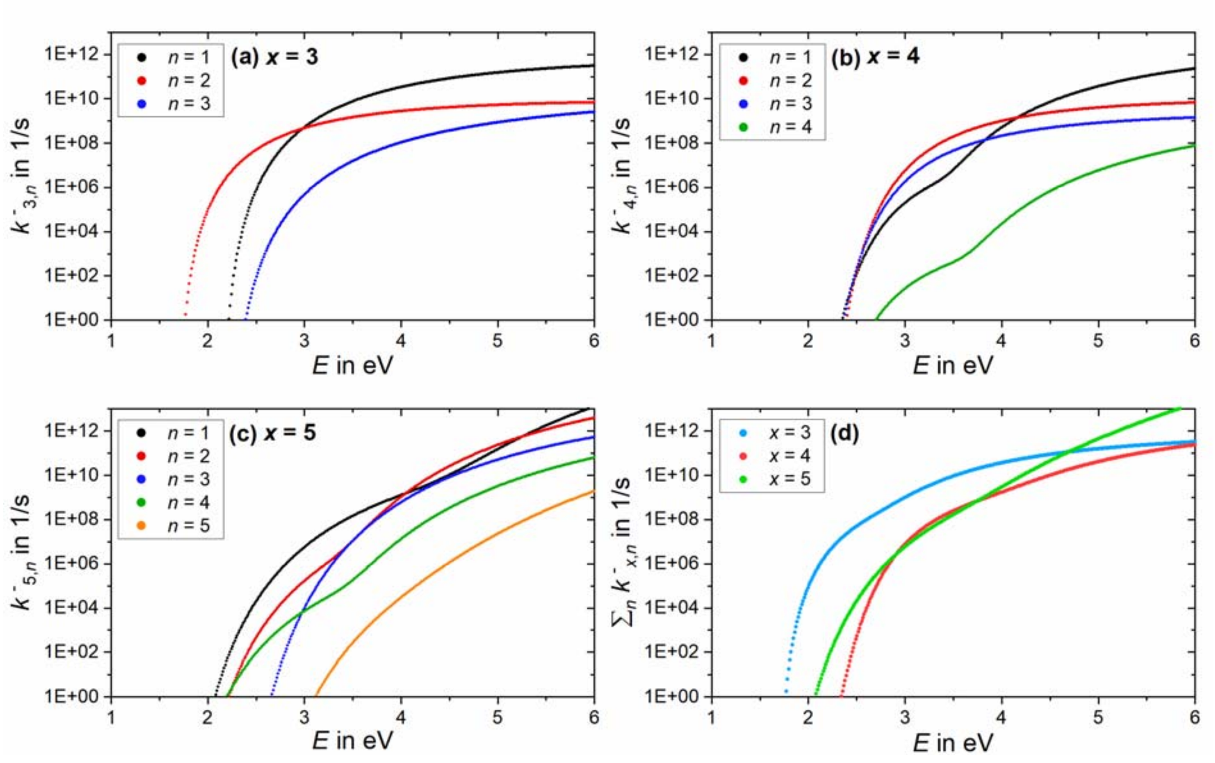

Figure S21: Method: CCSD/TZ//ωB97XD. (a)–(c) Calculated RRKM rate constants $k^-_{x,n}$ for the loss channels (3) of the precursor ions $(NaCl)_xCl^-$ with $x = 3–5$. (d) Total fragmentation rate constants $\sum_n k^-_{x,n}$ as sum of all loss channels n for cluster sizes $x = 3–5$.

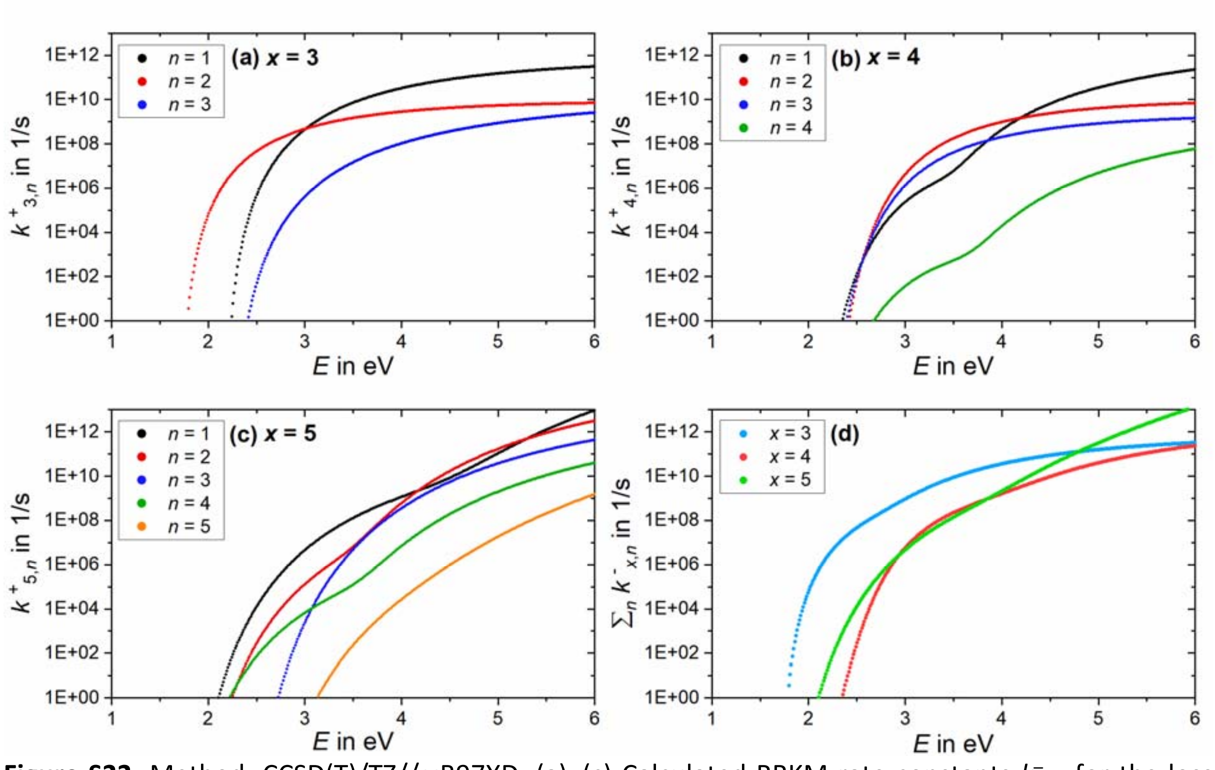

Figure S22: Method: CCSD(T)/TZ//ωB97XD. (a)–(c) Calculated RRKM rate constants $k^-_{x,n}$ for the loss channels (3) of the precursor ions $(NaCl)_xCl^-$ with $x = 3–5$. (d) Total fragmentation rate constants $\sum_n k^-_{x,n}$ as sum of all loss channels n for cluster sizes $x = 3–5$.

5. Mass Spectra

Figures S23 and S24 show mass spectra of the sodium chloride cluster cations and anions without any isolation. These mass spectra demonstrate, that only the pure sodium chloride cluster ions without contamination (e.g., water) are observed in the mass spectra. Figures S25–S30 show mass spectra of specific cluster sizes for a SORI Power of 1 %, representing the mass spectra used to obtain the SORI CID breakdown curves in Figures 4 and 5.

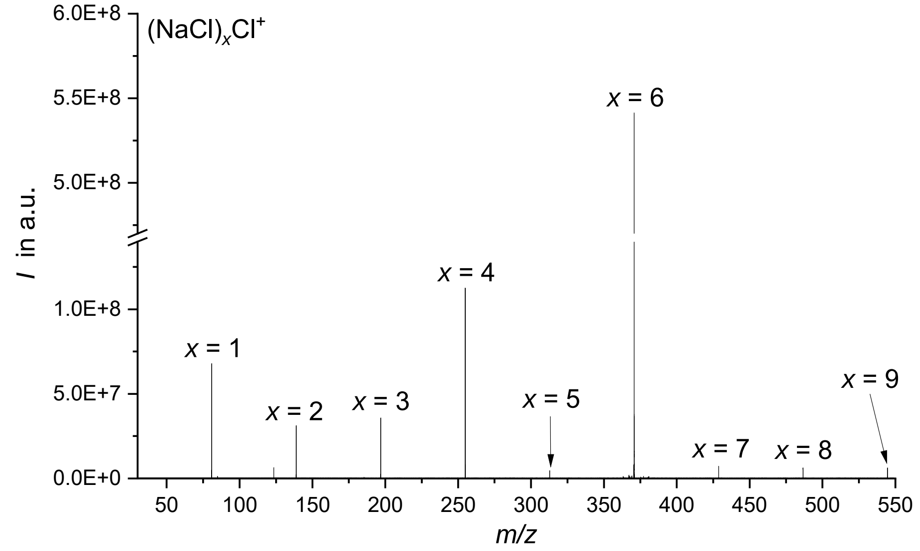

Figure S23: Mass spectrum of the sodium chloride cluster cations. The peak at $m/z \approx 123.6$ is the third harmonic of the $x = 6$ peak. Only the pure sodium chloride cluster cations without contamination (e.g., water) are observed in the mass spectra. The intensity distribution of the peaks depends significantly on the settings of the FT-ICR MS and does not represent the inherent stability of specific cluster sizes.

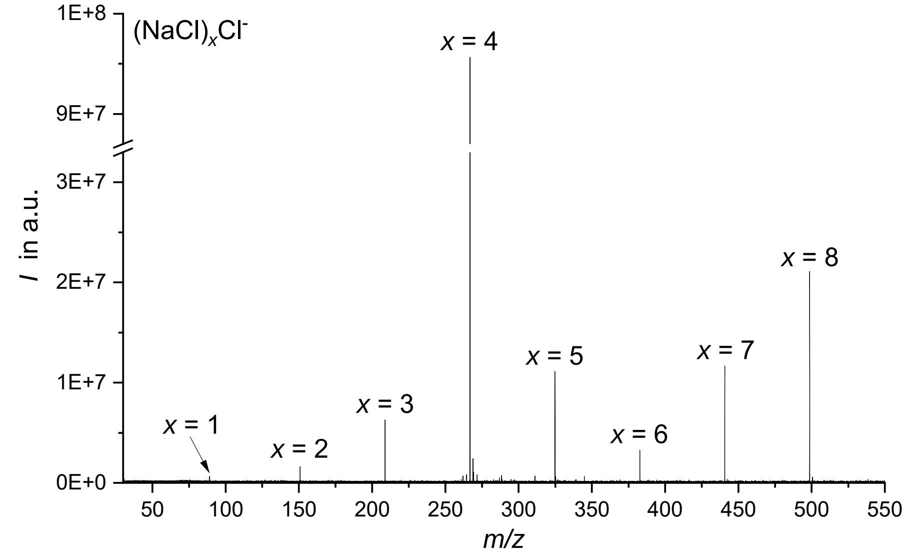

Figure S24: Mass spectrum of the sodium chloride cluster anions. Only the pure sodium chloride cluster anions without contamination (e.g. water) are observed in the mass spectra. The intensity distribution of the peaks depends significantly on the settings of the FT-ICR MS and does not represent the inherent stability of specific cluster sizes.

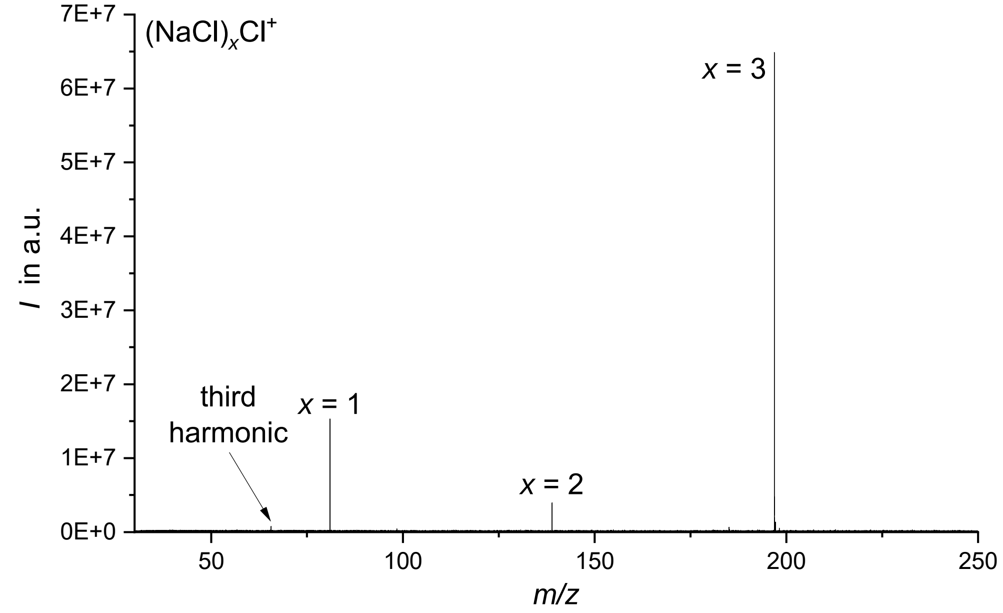

Figure S25: Mass spectrum of the cationic cluster size x = 3 at 1 % SORI Power.

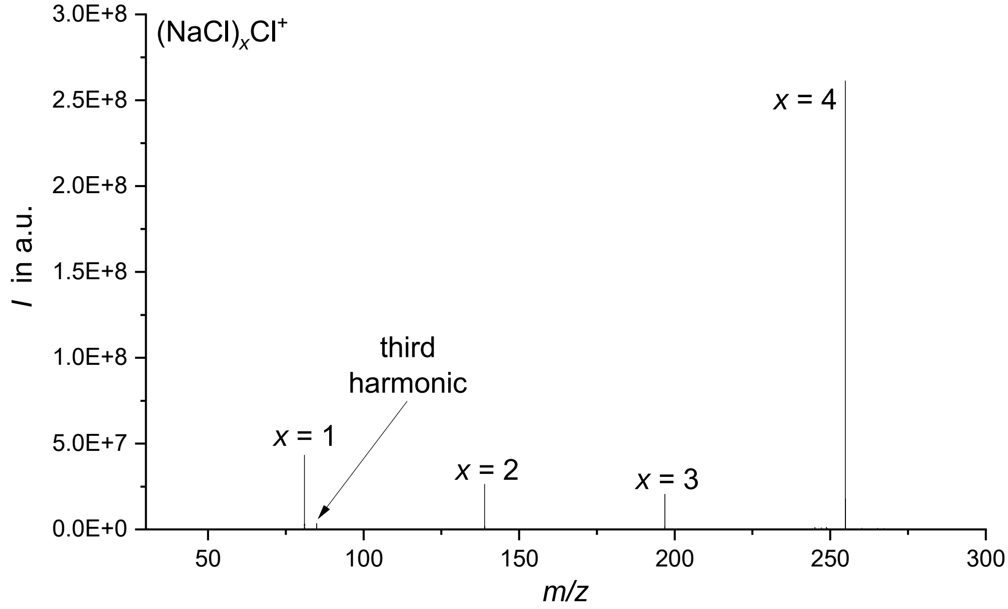

Figure S26: Mass spectrum of the cationic cluster size x = 4 at 1 % SORI Power.

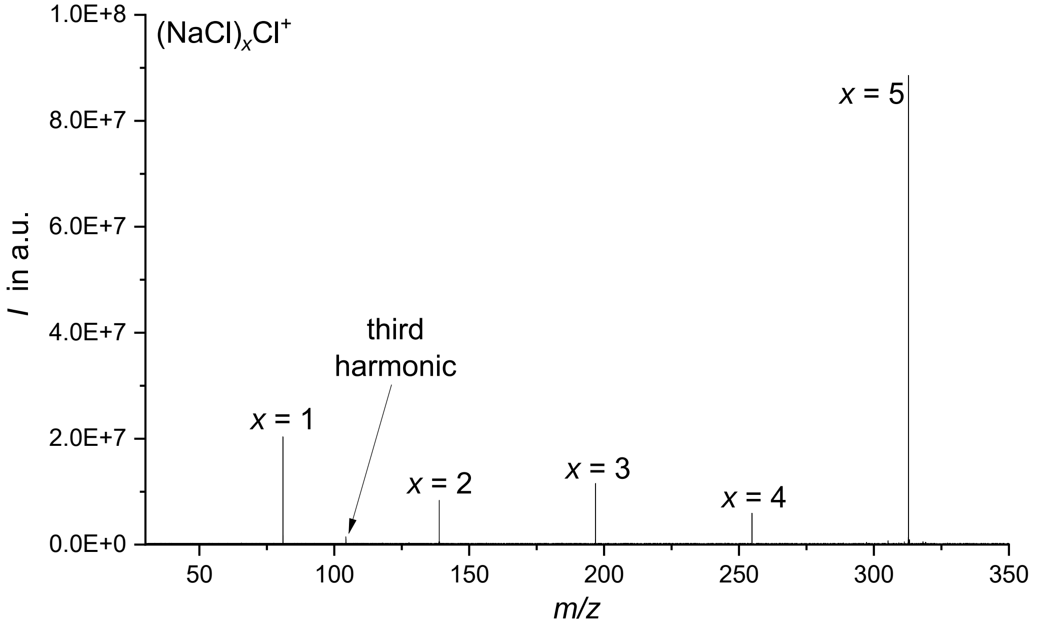

Figure S27: Mass spectrum of the cationic cluster size x = 5 at 1 % SORI Power.

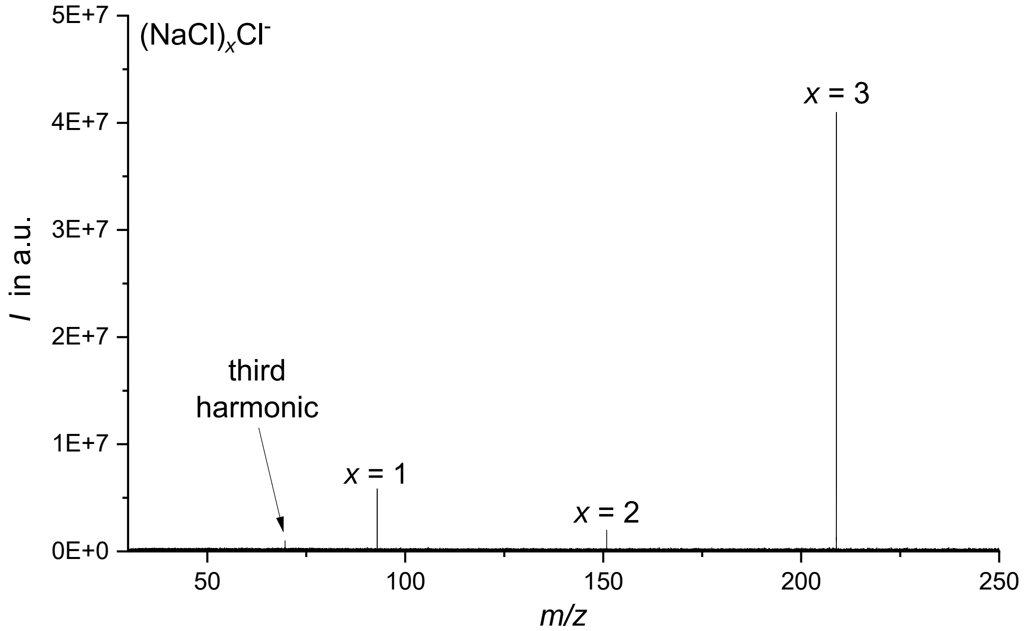

Figure S28: Mass spectrum of the anionic cluster size x = 3 at 1 % SORI Power.

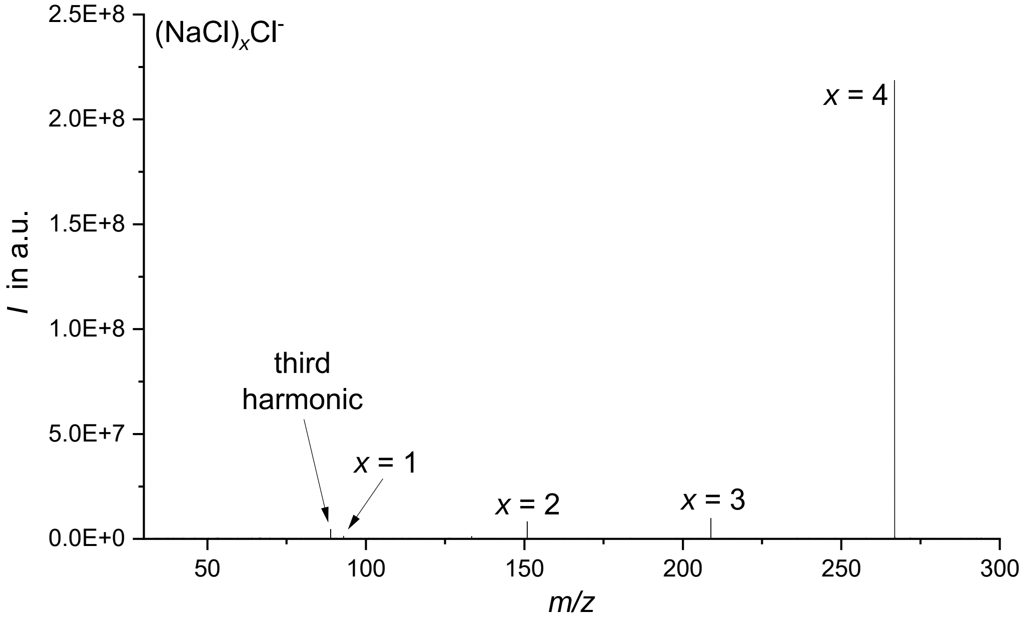

Figure S29: Mass spectrum of the anionic cluster size *x* = 4 at 1 % SORI Power.

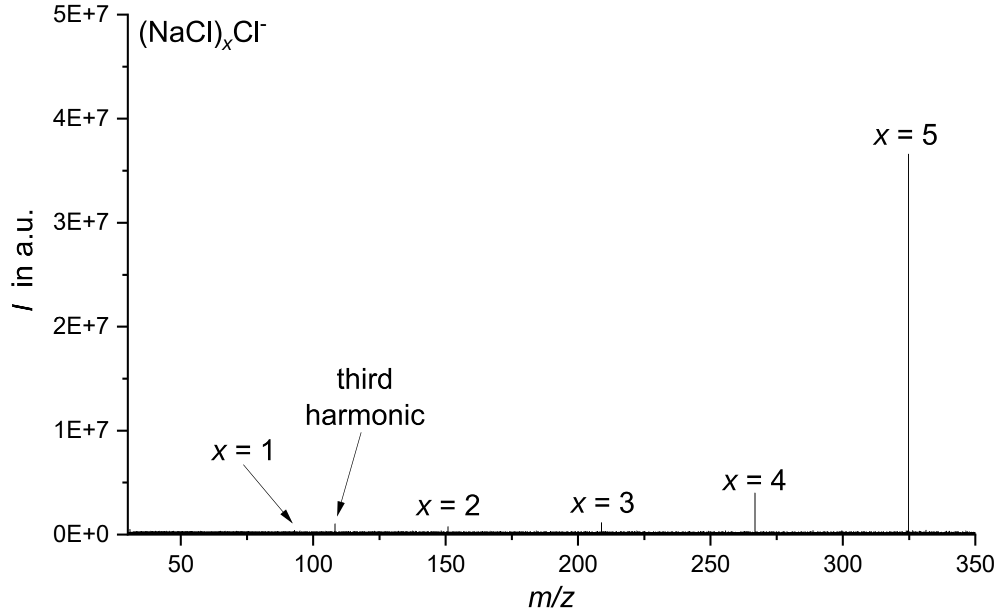

Figure S30: Mass spectrum of the anionic cluster size *x* = 5 at 1 % SORI Power.

6. Quantum Chemical Calculations

In the following chapters 5.1-5.6, the structures of the cationic clusters (chapter 5.1: B3LYP-D3/aug-cc-pVDZ; chapter 5.2: ωB97XD/aug-cc-pVDZ), the anionic clusters (chapter 5.3: B3LYP-D3/aug-cc-pVDZ; chapter 5.4: ωB97XD/aug-cc-pVDZ) and the neutral clusters (chapter 5.5: B3LYP-D3/aug-cc-pVDZ; chapter 5.6: ωB97XD/aug-cc-pVDZ) are listed.

6.1 Structures Cationic Clusters: B3LYP-D3/aug-cc-pVDZ

C1a
Na 0.000000 -0.000000 2.493268
Cl 0.000000 0.000000 0.000000
Na 0.000000 0.000000 -2.493268

C2a
Cl 0.000000 0.000000 1.747963
Na -0.000000 2.036912 -0.000000
Na -1.764017 -1.018456 -0.000000
Na 1.764017 -1.018456 -0.000000
Cl 0.000000 0.000000 -1.747963

C2b
Cl -0.000000 -2.552744 -0.000117
Na -0.000000 0.000000 0.001367
Cl 0.000000 2.552744 -0.000117
Na -0.859986 4.859979 -0.000503
Na 0.859986 -4.859979 -0.000503

C2c
Na -0.000000 1.782555 0.861439
Cl 0.000000 0.000000 2.623818
Na -0.000000 -1.782555 0.861439
Cl 0.000000 -0.000000 -1.284478
Na 0.000000 -0.000000 -3.792767

C3a
Cl 0.000000 2.274767 0.147216
Na 0.000000 0.000000 1.653838
Na 0.000000 -2.578971 -0.778796
Cl -1.970006 -1.137383 0.147216
Na -2.233454 1.289486 -0.778796
Na 2.233454 1.289486 -0.778796
Cl 1.970006 -1.137383 0.147216

C3c
Na -0.000000 1.729401 2.991697
Cl -0.000000 0.000000 4.823544
Na -0.000000 -1.729401 2.991697
Cl -0.000000 0.000000 0.906709
Na 0.000000 -0.000000 -1.661599
Cl 0.000000 -0.000000 -4.207422
Na 0.000000 -0.000000 -6.675260

C3d
Na -0.000000 7.307436 0.080484
Cl -0.000021 4.920715 -0.493598
Na 0.000022 2.418960 0.078272
Cl -0.000000 -0.000000 0.781747
Na -0.000022 -2.418960 0.078272
Cl 0.000021 -4.920715 -0.493598
Na 0.000000 -7.307436 0.080484

C4a
Cl 0.000000 2.772899 0.505198
Na -2.504504 2.571625 -0.134536
Cl -2.693266 0.000000 -0.419586
Na 0.000000 0.000000 0.273525
Na -2.504504 -2.571625 -0.134536
Cl -0.000000 -2.772899 0.505198
Na 2.504504 -2.571625 -0.134536
Cl 2.693266 -0.000000 -0.419586
Na 2.504504 2.571625 -0.134536

C4b
Na -0.427501 0.747500 1.811302
Cl -0.427501 -1.856514 1.894294
Na -1.956013 -2.784312 -0.000000
Cl -0.427501 -1.856514 -1.894294
Na -0.427501 0.747500 -1.811302
Cl -1.167425 2.595860 0.000000
Na 1.554331 -1.779157 -0.000000
Na 1.330772 3.437509 0.000000
Cl 1.974490 0.878378 -0.000000

C4c
Cl -0.682809 1.889685 -1.667630
Na -0.000000 3.458145 0.243044
Cl 1.540640 1.655316 1.393180
Na -0.000000 -0.000000 2.635742
Cl -1.540640 -1.655316 1.393180
Na -0.000000 -3.458145 0.243044
Na -1.630167 -0.506011 -1.136765
Na 1.630167 0.506011 -1.136765
Cl 0.682809 -1.889685 -1.667630

C4d
Na 0.000000 2.223010 0.101213
Cl 2.001024 1.155292 -1.202695
Na 1.925183 -1.111505 0.101213
Cl -0.000000 -2.310584 -1.202695
Na -1.925183 -1.111505 0.101213
Cl -2.001024 1.155292 -1.202695
Na 0.000000 0.000000 -2.657164
Cl 0.000000 0.000000 2.134512
Na 0.000000 0.000000 4.630863

C4g
Na 0.000000 1.738880 4.658142
Cl 0.000000 0.000000 2.561760
Na 0.000000 0.000000 0.000000
Cl 0.000000 0.000000 -2.561760
Na 1.738880 -0.000000 -4.658142
Cl 0.000000 0.000000 6.475113
Na -0.000000 -1.738880 4.658142
Cl 0.000000 0.000000 -6.475113
Na -1.738880 0.000000 -4.658142

C4h
Na 0.000000 0.000000 -5.051596
Cl 0.000000 2.260235 -3.867923
Na 0.000000 2.032623 -1.389267
Cl -0.000000 -2.260235 -3.867923
Na -0.000000 -2.032623 -1.389267
Cl 0.000000 0.000000 0.331537
Na 0.000000 0.000000 2.916339
Cl 0.000000 0.000000 5.455030
Na 0.000000 0.000000 7.926314

C4i
Cl 7.319231 0.199687 0.000000
Na 4.765452 0.492299 0.000000
Cl 2.311966 1.027021 0.000000
Na -0.000000 -0.009801 -0.000000
Cl -2.313993 -1.040780 -0.000000
Na -4.764626 -0.492975 -0.000000
Na 9.551378 1.214821 0.000000
Cl -7.316506 -0.190605 -0.000000
Na -9.553283 -1.197116 -0.000000

C5a
Na -2.118988 -0.754066 0.000000
Cl -0.644266 0.256020 2.053868
Na 0.635348 -2.357166 1.796154
Cl 2.416282 -1.520286 -0.000000
Na 1.404071 0.876555 -0.000000
Cl -0.644266 0.256020 -2.053868
Na -0.644266 2.845682 -2.251560
Cl 0.497497 3.467653 0.000000
Na -0.644266 2.845682 2.251560
Na 0.635348 -2.357166 -1.796154
Cl -1.151113 -3.170861 -0.000000

C5b
Cl -0.000000 2.242614 0.000000
Na -1.736104 1.002340 1.802050
Na 1.736104 1.002340 1.802050
Na -1.736104 1.002340 -1.802050
Na 1.736104 1.002340 -1.802050
Cl 0.000000 0.000000 3.463214
Na -0.000000 -2.004680 1.802050
Cl 0.000000 -0.000000 -3.463214
Na -0.000000 -2.004680 -1.802050
Cl -1.942160 -1.121307 0.000000
Cl 1.942160 -1.121307 0.000000

C5c
Na 2.159206 -0.000000 0.172085
Cl 1.941215 2.566541 -0.394166
Na 0.000000 3.213383 -1.985329
Cl -1.941215 2.566541 -0.394166
Na -2.159206 0.000000 0.172085
Cl -1.941215 -2.566541 -0.394166
Na 0.000000 -2.649420 1.509858
Cl 0.000000 0.000000 1.969281
Na 0.000000 2.649420 1.509858
Cl 1.941215 -2.566541 -0.394166
Na -0.000000 -3.213383 -1.985329

C5d
Cl 3.400379 -0.647211 0.167764

Na 2.004261 0.989947 1.658618
Cl 0.661138 1.926527 -0.824117
Na -1.472104 1.954793 1.068308
Cl -0.283491 -0.249984 2.194629
Na 1.136079 -2.016421 0.431007
Na 2.834008 0.829341 -1.879449
Cl -3.323860 0.806240 -0.299371
Na -2.564748 -1.512655 0.701508
Cl -0.835077 -2.188513 -1.240374
Na -1.348816 0.300448 -1.977722

C5e
Na 1.765593 1.100894 3.240741
Cl 0.000000 1.995221 -2.694528
Na -0.000000 -0.000000 -4.324788
Cl -1.939762 -1.417825 0.675263
Na -1.765593 -1.100894 3.240741
Cl -0.000000 -1.995221 -2.694528
Na 1.766464 3.159784 -1.232130
Cl 0.000000 0.000000 4.649202
Na -1.766464 -3.159784 -1.232130
Cl 1.939762 1.417825 0.675263
Na -0.000000 -0.000000 -0.636199

C5f
Na -0.540566 3.547501 0.615858
Cl -2.650037 2.065772 0.590984
Na -4.377886 0.485662 -0.528206
Cl 1.269390 1.783134 -0.006824
Cl -2.864190 -1.513904 -1.154128
Na -1.597207 -3.503193 -0.069481
Cl 0.259363 -2.192360 1.164032
Na 2.741828 -1.437021 0.953074
Na -0.846387 0.006952 0.052151
Cl 4.632710 -0.591665 -0.493842
Na 3.619945 1.594042 -1.178283

C5g
Na 0.000000 0.000000 -4.150138
Cl 2.005490 1.157870 -2.723446
Na 0.000000 2.164754 -1.354174
Cl -2.005490 1.157870 -2.723446
Na -1.874732 -1.082377 -1.354174
Cl 0.000000 0.000000 0.553769
Na 0.000000 0.000000 3.112315
Cl 0.000000 0.000000 5.658445
Na 0.000000 0.000000 8.126536
Na 1.874732 -1.082377 -1.354174
Cl -0.000000 -2.315740 -2.723446

C5h
Na -0.000000 4.214824 -1.061411
Cl 0.000000 -1.929626 -2.317052
Na -0.000000 -4.214824 -1.061411
Cl 0.000000 -4.495034 1.434303
Na -0.000000 -0.000000 -0.430141
Cl 0.000000 1.929626 -2.317052
Na 0.000000 -2.505729 2.961623
Cl 0.000000 0.000000 2.213803
Na 0.000000 2.505729 2.961623
Cl 0.000000 4.495034 1.434303
Na -0.000000 -0.000000 -4.063117

C5i
Na 0.773944 3.464432 -0.226102
Cl -0.776473 1.774045 0.904638
Cl 2.922834 2.064301 -0.589438
Na 4.790260 0.297772 -0.423949
Cl 3.555759 -1.717035 0.601430
Na 1.775366 -3.405121 -0.234298
Cl -0.322640 -1.945944 -0.312360
Na 1.310184 0.035728 0.602901
Na -2.329904 -0.176396 -0.085585
Cl -4.870780 -0.206809 -0.610273
Na -7.106024 -0.167822 0.376308

C5j
Na -4.153356 0.665113 -0.015216
Cl -6.376377 -0.590415 0.068436
Na -4.996416 -2.698079 0.027894
Cl -2.537825 -1.803230 -0.058337
Na -0.560812 -0.162791 -0.063834
Cl -1.930429 2.177562 -0.047796
Na 0.337636 3.318663 0.039449
Cl 1.844627 1.146811 0.012370
Na 4.249256 0.254089 0.020875
Cl 6.644355 -0.610959 0.017278
Na 8.764241 -1.871184 0.003271

C5k
Na -2.699772 -2.140847 -0.000000
Cl -2.783428 -4.685877 -0.000000
Na -5.295888 -4.410616 -0.000000
Cl -5.530980 -1.776378 -0.000000
Na -4.925851 0.700942 -0.000000
Cl -2.308154 0.537994 -0.000000
Na 0.000000 1.696747 0.000000
Cl 2.285093 2.735316 0.000000
Na 4.793097 2.457754 0.000000
Cl 7.350319 2.268138 0.000000
Na 9.654011 3.119085 0.000000

C5l
Na 2.056003 -6.796260 0.000000
Cl 3.930853 -5.067903 0.000000
Na 3.063645 -2.729902 0.000000
Cl -0.444018 -6.289420 0.000000
Na -0.859676 -3.830347 0.000000
Cl 0.641932 -1.648971 0.000000
Na -0.000000 0.867747 -0.000000
Cl -0.429640 3.333482 -0.000000
Na -1.773619 5.475249 -0.000000
Cl -3.092937 7.671263 -0.000000
Na -3.423192 10.106819 -0.000000

C6a
Na 2.483365 1.433771 1.396197
Cl 0.000000 2.489056 1.387921
Na -2.483365 1.433771 1.396197
Cl -2.155586 1.244528 -1.387921
Na 0.000000 2.867543 -1.396197
Cl 2.155586 1.244528 -1.387921
Na 2.483365 -1.433771 -1.396197
Cl 2.155586 -1.244528 1.387921
Na -0.000000 -2.867543 1.396197
Cl -0.000000 -2.489056 -1.387921
Na 0.000000 0.000000 0.000000
Cl -2.155586 -1.244528 1.387921
Na -2.483365 -1.433771 -1.396197

C6b
Na 0.000000 0.000000 2.707642
Cl 2.033193 1.173864 1.472455
Na 2.092183 1.207922 -1.507070
Cl -0.000000 2.771113 -1.678898
Na -2.092183 1.207922 -1.507070
Cl -2.399854 -1.385556 -1.678898
Na -0.000000 -2.415845 -1.507070
Cl -0.000000 -2.347729 1.472455
Na -0.000000 2.977109 0.923571
Cl -2.033193 1.173864 1.472455
Na -2.578252 -1.488554 0.923571
Cl 2.399854 -1.385556 -1.678898
Na 2.578252 -1.488554 0.923571

C6c
Na 2.120491 1.815451 0.324573
Cl 0.000000 3.220740 -0.320350
Na -2.120491 1.815451 0.324573
Na 0.000000 1.776975 -2.506751
Cl 1.966167 -0.000000 -1.845121
Cl -1.966167 0.000000 -1.845121
Cl 2.243404 -0.000000 2.234660
Na -0.000000 -1.776975 -2.506751
Cl -0.000000 -3.220740 -0.320350
Na -2.120491 -1.815451 0.324573
Na 2.120491 -1.815451 0.324573
Cl -2.243404 0.000000 2.234660
Na -0.000000 0.000000 3.501351

C6d
Na 1.867877 2.572302 0.284364
Cl 0.000000 2.633950 2.091952
Na 0.000000 0.000000 2.122477
Cl -2.666739 0.000000 0.198638
Na -1.661905 0.000000 -2.224710
Cl 0.000000 2.098420 -1.905756
Na 1.661905 -0.000000 -2.224710
Cl -0.000000 -2.098420 -1.905756
Na -1.867877 -2.572302 0.284364
Cl -0.000000 -2.633950 2.091952
Na 1.867877 -2.572302 0.284364
Na -1.867877 2.572302 0.284364
Cl 2.666739 -0.000000 0.198638

C6e
Na -2.783201 -0.885009 -1.259199
Cl -2.648719 1.714292 -1.213374
Na -2.350673 1.628385 1.378742
Cl 0.295588 2.034992 1.745924
Na 2.605131 2.695497 0.717407
Cl 2.571974 1.389897 -1.494627
Na 2.466209 -1.234622 -1.258706
Cl 2.028937 -2.255128 1.152539
Na -0.311667 -3.061591 0.264847
Cl -0.251452 -1.571545 -1.982484
Na -0.081934 1.241535 -1.607254
Cl -2.015743 -1.223679 1.419706
Na 0.486140 -0.521474 2.339561

C6f
Cl -0.583426 0.257265 1.940655
Na -0.583426 3.176073 1.781431
Cl -2.560890 3.214714 0.000000
Na -0.583426 3.176073 -1.781431
Cl -0.583426 0.257265 -1.940655
Na 1.620725 0.700831 0.000000
Cl 1.388401 3.292474 0.000000
Na 0.090763 -2.246829 1.797790
Cl 2.380267 -1.915524 0.000000
Na 2.272125 -4.561370 0.000000
Cl -0.339298 -4.212123 0.000000
Na 0.090763 -2.246829 -1.797790
Na -2.446405 0.620307 0.000000

C6g
Na 0.136118 0.998609 2.248565
Cl 0.136118 -1.617069 2.387575
Na -0.229759 -4.166106 2.309091
Cl -1.369440 -4.432726 0.000000
Na -1.252814 -1.621860 0.000000
Cl -1.473370 1.140656 -0.000000
Na -0.745786 3.729634 -0.000000
Cl 1.096309 3.445339 1.968167
Na 2.769998 3.663947 -0.000000
Cl 1.096309 3.445339 -1.968167

Na 0.136118 0.998609 -2.248565
Cl 0.136118 -1.617069 -2.387575
Na -0.229759 -4.166106 -2.309091

C6h
Na 1.678338 2.078510 1.423763
Cl 3.707758 1.589489 -0.106763
Na 1.801457 0.588746 -1.708781
Cl -0.267062 2.086369 -0.572157
Na -2.712926 2.853029 -1.025126
Cl -3.995151 1.054632 0.296433
Na -4.493749 -1.409992 -0.382088
Cl -2.154568 -2.412908 0.011507
Na 0.430298 -2.804392 0.091919
Cl 1.050006 -0.558949 1.832631
Na -1.487493 0.009831 0.989237
Na 3.693469 -0.939381 0.594197
Cl 2.364703 -2.002153 -1.450727

C6i
Na 2.418892 -1.288059 1.752061
Cl -0.326612 -1.322407 2.122567
Na -0.226389 -3.001009 0.000000
Cl -0.326612 -1.322407 -2.122567
Na -0.087787 1.165343 -0.000000
Cl -1.647514 2.525705 1.968244
Na -1.647514 0.436222 -3.448295
Cl -1.647514 2.525705 -1.968244
Na -1.647514 0.436222 3.448295
Cl 2.385327 -3.285427 0.000000
Na -2.692865 3.816464 -0.000000
Cl 2.510402 0.699514 0.000000
Na 2.418892 -1.288059 -1.752061

C6j
Na 4.161857 1.295272 -1.310646
Cl 2.465997 2.247894 0.395723
Na 3.018021 0.053809 1.876922
Cl 4.123684 -1.118137 -0.337436
Cl -0.614572 -1.200389 -1.698788
Na 0.010176 1.349645 0.004037
Cl -2.433845 2.232761 -0.469211
Na -3.007255 -0.003882 -1.878149
Na -4.153200 1.364128 1.259541
Cl -4.138661 -1.081412 0.374410
Na -1.728100 -2.007530 0.769883
Na 1.709618 -2.041653 -0.709875
Cl 0.590203 -1.087052 1.727724

C6k
Na 0.890364 -3.488119 2.178466
Cl -0.274611 -4.283765 -0.000000
Na 0.890364 -3.488119 -2.178466
Cl 0.386635 -0.964218 2.055877
Na -1.156608 1.331472 2.350084
Cl 0.890364 2.884693 1.959481
Na 0.631961 4.518776 0.000000
Cl 0.890364 2.884693 -1.959481
Na -1.156608 1.331472 -2.350084
Cl 0.386635 -0.964218 -2.055877
Na 1.308022 0.937985 0.000000
Cl -2.308136 0.807177 0.000000
Na -1.363067 -1.706568 -0.000000

C6l
Na 3.084191 -1.501853 0.541028
Cl 3.911710 0.945268 0.221220
Na 2.275337 0.823476 -1.877969
Cl 0.328791 2.295645 -0.541924
Na -2.004602 3.472318 -0.537454
Cl -3.410544 1.528307 0.357287
Na -4.615184 -0.762139 0.137978
Cl -2.597315 -2.277890 -0.343975
Na -0.172357 -3.108787 -0.001200
Cl 0.759910 -1.011473 1.914981
Na -1.075935 -0.053629 0.049808
Na 1.804125 1.522515 1.685070
Cl 1.463252 -1.733441 -1.605816

C6m
Cl 0.347612 1.517817 -1.355252
Na -2.170413 -0.431800 1.819453
Cl 0.441456 -0.430134 2.030080
Na 3.030372 -1.334329 1.269332
Cl 2.446338 -1.994501 -1.250572
Na -0.059491 -1.377848 -0.858802
Cl -3.782696 1.378487 0.438930
Na -2.045423 2.415496 -1.161963
Na 1.460279 1.850520 1.130825
Cl 3.904162 1.132941 0.642202
Na 3.199820 0.461636 -1.813985
Na -4.677673 -0.798033 -0.761827
Cl -2.539941 -2.112968 -0.261468

C6n
Na -0.104500 -2.948086 1.768679
Cl -2.092026 -3.011197 0.000000
Cl 1.905064 -2.920977 0.000000
Na 1.610954 -0.302749 0.000000
Cl -0.104500 -0.214496 -2.137027
Na -2.292635 -0.383742 0.000000
Na -0.104500 -2.948086 -1.768679
Cl -0.104500 -0.214496 2.137027
Na -0.594277 2.330999 1.897483
Cl 1.653664 2.709415 0.000000
Na 3.823242 3.941108 0.000000
Cl -2.386176 2.344406 0.000000
Na -0.594277 2.330999 -1.897483

C6o
Cl 3.972862 -0.950543 -0.987082
Na 2.539031 -2.265479 0.823921
Na 1.746518 -0.252972 -2.153719
Na 3.646763 1.130766 0.641022
Cl 2.210522 -0.183255 2.459963
Na -0.029927 0.405210 1.265099
Cl 0.062572 -1.707104 -0.592549
Na -2.541714 -1.770472 -0.847209
Cl -2.368515 1.505503 0.419200
Na -0.944995 3.190559 -0.962664
Cl 1.390560 2.110499 -0.852431
Na -4.874722 0.727743 0.874560
Cl -4.970970 -1.529153 -0.214812

C6p
Na 1.415231 -2.538532 1.734948
Cl 2.693547 -0.742969 0.000000
Cl -0.652796 -0.697070 1.930733
Cl 0.383999 -4.180316 0.000000
Na -1.592762 -2.475681 0.000000
Na 1.415231 -2.538532 -1.734948
Cl -0.652796 -0.697070 -1.930733
Na -1.855922 1.079220 0.000000
Cl 0.790020 2.910484 0.000000
Na 1.415231 1.029344 1.720153
Na 1.415231 1.029344 -1.720153
Na -1.207100 4.713819 0.000000
Cl -3.212358 3.213482 0.000000

C6q
Na 0.766557 0.134341 1.783697
Cl 0.766557 -2.458713 1.938708
Cl 2.140489 1.552038 0.000000
Na 0.766557 0.134341 -1.783697
Cl 0.766557 -2.458713 -1.938708
Na -1.008483 -2.596155 0.000000
Na 2.651122 -2.982741 0.000000
Cl -1.492807 0.166452 0.000000
Na -3.352654 1.918228 0.000000
Cl -5.230116 3.639481 0.000000
Na -7.333804 4.928165 0.000000
Na 4.586025 0.625469 0.000000
Cl 4.941762 -1.839259 0.000000

C6r
Na -0.393238 3.493055 -0.246006
Cl -1.732205 1.652981 0.940970
Cl 1.618924 2.151545 -1.147461
Na 4.021845 1.237692 -0.768233
Cl 2.534838 -1.709278 0.161827
Na 0.765612 -3.320994 -0.880009
Cl -1.321884 -1.864516 -0.841015
Na -3.331648 -0.260412 -0.047532
Cl -5.916226 -0.449430 -0.153149
Na -8.012620 0.051454 0.996266
Na 0.339351 0.072018 0.084957
Na 4.838394 -1.647246 1.427531
Cl 5.963340 0.460979 0.671962

C6s
Cl 0.846385 3.351224 -0.000000
Na -0.591075 1.099940 -0.000000
Cl -1.057996 -0.660068 1.928170
Cl 1.840566 1.954762 4.333344
Na 0.351785 -0.053117 3.992279
Na 1.840566 3.557219 2.403699
Na 1.840566 3.557219 -2.403699
Cl 1.840566 1.954762 -4.333344
Na 0.351785 -0.053117 -3.992279
Cl -1.057996 -0.660068 -1.928170
Na -1.404936 -2.508214 0.000000
Cl -1.784422 -5.069656 0.000000
Na -3.357853 -6.945951 0.000000

C6t
Na 11.272813 -3.345901 0.000000
Cl 8.869680 -2.836952 0.000000
Na 6.835416 -1.275770 0.000000
Cl 4.907575 0.352219 0.000000
Na 2.464484 0.933882 0.000000
Cl 0.000000 1.692571 0.000000
Na -1.525304 3.833897 0.000000
Cl -3.788602 2.672440 0.000000
Na -2.369504 0.348841 0.000000
Cl -4.335396 -1.312886 0.000000
Na -5.977424 1.120524 0.000000
Cl -8.192232 -0.160172 0.000000
Na -6.776609 -2.244812 0.000000

C6u
Na 8.057042 0.236806 -0.000718
Cl 5.567018 0.569338 -0.006417
Na 3.328255 -0.611688 -0.003422
Cl 1.071730 -1.772054 -0.000563
Na -1.411547 -1.468057 0.000031
Cl -3.990767 -1.276317 0.001078
Na -6.256895 -2.581812 0.000247
Cl -7.906525 -0.637695 0.001015
Na -5.515512 0.960348 0.001723
Cl -6.741891 3.194096 0.000343
Na -8.862682 1.821703 -0.000319
Cl 10.624826 0.143665 0.005094
Na 12.787282 1.301105 0.001607

C6v
Cl -2.287242 -3.190359 -0.000000

Na -1.013247 -0.922705 -0.000000
Cl -0.000000 1.356352 -0.000000
Na 1.998933 2.927860 0.000000
Cl 4.018677 4.449261 0.000000
Na 4.796534 6.840561 0.000000
Cl 5.769027 9.218110 0.000000
Na 7.655210 10.786906 0.000000
Na -4.907912 -3.567420 -0.000000
Cl -6.372943 -5.587985 -0.000000
Na -5.022164 -7.750157 -0.000000
Cl -2.479319 -7.945921 0.000000
Na -1.418210 -5.686938 0.000000

C7a
Cl 0.000000 2.397813 1.103080
Na 1.861243 1.074589 2.618182
Na -1.861243 1.074589 2.618182
Na 0.000000 0.000000 -0.291243
Cl 0.000000 0.000000 4.173511
Na -0.000000 -2.149178 2.618182
Cl -2.076567 -1.198907 1.103080
Na -2.522460 -1.456343 -1.494843
Cl -1.894245 1.093643 -2.319934
Na 0.000000 0.000000 -3.886966
Cl 1.894245 1.093643 -2.319934
Na 2.522460 -1.456343 -1.494843
Cl 2.076567 -1.198907 1.103080
Na 0.000000 2.912686 -1.494843
Cl -0.000000 -2.187285 -2.319934

C7b
Na 0.000000 0.000000 -2.909685
Cl 0.000000 2.654879 -2.853492
Na -1.829773 -2.645265 -0.955006
Cl -0.000000 0.000000 3.812849
Na 0.000000 2.582232 3.624254
Cl 2.115232 -0.000000 -0.646807
Na 1.829773 2.645265 -0.955006
Cl -0.000000 -3.048193 1.084167
Na -0.000000 0.000000 1.056657
Cl -0.000000 -2.654879 -2.853492
Na -0.000000 -2.582232 3.624254
Cl -2.115232 0.000000 -0.646807
Na -1.829773 2.645265 -0.955006
Cl 0.000000 3.048193 1.084167
Na 1.829773 -2.645265 -0.955006

C7c
Na -2.220272 -2.010759 -1.797734
Cl -3.589207 -1.461203 0.424789
Na -1.371975 -2.081135 1.667095
Na -3.218864 1.083129 0.019521
Cl 0.832248 -0.685808 2.242953
Na 0.369331 1.901662 1.988884
Cl -1.784496 3.041697 0.972607
Na -0.483155 3.121170 -1.347781
Cl 1.871060 2.389305 -0.255830
Na 1.024560 -0.369805 -1.128172
Na 2.296039 -2.587798 0.891553
Cl -1.337567 0.614097 -2.076814
Cl -0.006645 -2.942818 -0.564621
Cl 3.625840 -1.149424 -0.825567
Na 4.205159 1.243590 -0.165891

C7d
Cl 2.764806 -1.502901 -0.297609
Na 0.979757 3.010295 1.192143
Cl -1.149391 2.817380 -0.571713
Na -2.455784 1.674283 1.519507
Cl -3.986984 0.057784 0.196696
Na -2.748438 1.016688 -1.945166
Cl -0.870510 -0.995594 -2.078830
Na 0.813512 -3.059760 -1.426928
Cl -0.307953 -3.569205 0.923522
Na -2.165103 -1.819611 0.343620
Cl -0.029080 0.572343 2.069365
Na 1.283195 -1.751703 1.973159
Na 0.819356 0.798152 -1.051001
Na 4.497053 0.426896 -0.310552
Cl 2.916816 2.429155 -0.432174

C7e
Cl 0.407472 3.738417 2.007096
Na -1.321058 4.044756 0.000000
Na 2.126579 3.396130 0.000000
Cl 0.407472 3.738417 -2.007096
Na -0.087553 1.145689 -1.958702
Cl 1.886772 0.633287 0.000000
Cl -2.119311 1.388739 0.000000
Na -0.087553 1.145689 1.958702
Na 1.363095 -1.908671 -0.000000
Cl -0.687988 -1.785805 -2.051621
Na -2.347895 -1.193430 -0.000000
Cl -0.687988 -1.785805 2.051621
Na -0.087553 -4.301464 -2.268465
Cl 1.136182 -4.650754 -0.000000
Na -0.087553 -4.301464 2.268465

C7f
Na -2.438162 2.493693 0.000000
Cl -1.770454 -0.146049 0.000000
Cl -0.817802 3.202473 1.989602
Na 0.739312 4.080842 0.000000
Cl 2.302233 1.730179 0.000000
Na -1.526043 -2.191198 1.762258
Cl -1.544351 -4.135010 0.000000
Na 1.019878 -3.780352 0.000000
Cl 1.242097 -1.779168 -1.941566
Na 2.904642 -0.794605 0.000000
Cl 1.242097 -1.779168 1.941566
Na 0.539923 0.962890 1.959850
Na -1.526043 -2.191198 -1.762258
Cl -0.817802 3.202473 -1.989602
Na 0.539923 0.962890 -1.959850

C7g
Na -3.447801 1.332418 0.156457
Cl -3.830111 -1.124696 -0.500901
Na -1.436666 -1.106994 -1.686983
Na -2.530764 -1.915756 1.648173
Cl 1.077283 -0.723400 -2.570734
Na 2.079260 -2.576839 -0.944183
Cl -0.284122 -2.617085 0.421346
Na 0.955527 -0.462825 1.490877
Cl 2.119203 2.244531 0.415749
Na -0.350316 2.917627 1.173537
Na 1.006417 1.807710 -2.035355
Cl -1.476194 0.701504 2.210706
Cl -1.455884 2.390595 -1.212416
Na 4.353885 0.882539 0.529022
Cl 3.442474 -1.439489 1.021721

C7h
Cl -3.013383 0.000000 0.195948
Na -1.764191 1.815835 1.652739
Na -1.764191 -1.815835 1.652739
Cl -0.000000 -3.739243 1.188692
Na -0.000000 -4.116548 -1.353021
Cl 0.000000 -1.917855 -2.631009
Na 1.794779 -0.000000 -2.132785
Cl 3.013383 -0.000000 0.195948
Na 1.764191 -1.815835 1.652739
Na -1.794779 0.000000 -2.132785
Na 1.764191 1.815835 1.652739
Cl 0.000000 3.739243 1.188692
Cl -0.000000 -0.000000 2.726104
Cl 0.000000 1.917855 -2.631009
Na 0.000000 4.116548 -1.353021

C7i
Na -2.358654 -2.498363 1.149596
Cl -3.620277 -1.397146 -0.895606
Na -1.101132 -0.669930 -1.459415
Cl -1.492640 0.016707 2.052027
Cl 0.020847 -2.779361 -0.016888
Na 2.396728 -2.461035 -1.184328
Cl 3.647513 -1.367313 0.871186
Na 3.768162 1.002736 -0.103793
Cl 1.806668 2.501874 0.851993
Na 1.126051 -0.724859 1.500688
Na -0.709024 2.516193 1.653083
Cl -1.845850 2.479958 -0.817593
Na 0.673675 2.574954 -1.614103
Cl 1.511561 0.084299 -2.044387
Na -3.838803 0.972731 0.057140

C7j
Na -4.341754 -0.008031 -1.649736
Cl -2.032923 -1.160167 -1.653800
Cl -4.185667 1.277023 0.598076
Na -2.326536 3.066185 0.760009
Cl -0.244648 1.544418 0.995811
Na -0.477850 -3.219764 -0.627081
Cl 1.795860 -1.909687 -1.060352
Na 3.867886 -0.139697 -1.125891
Cl 2.355747 1.950189 -1.925858
Na 2.338100 2.447875 0.672922
Cl -0.672862 -2.527106 1.925355
Na -2.175850 -0.477038 1.358203
Na 0.293163 0.305124 -1.699602
Cl 3.717440 0.384041 1.504283
Na 1.690104 -1.292662 1.718471

C7k
Cl 2.958452 3.587396 -0.000000
Na 2.442612 1.039442 -0.000000
Na 0.996333 3.821880 1.790313
Na 0.996333 3.821880 -1.790313
Cl -0.943496 4.162039 -0.000000
Na -1.427764 1.601063 -0.000000
Cl 0.632737 0.959846 1.997559
Na -0.577465 -1.367776 2.165851
Cl -0.577465 -3.998573 1.944618
Na -2.448503 -3.651485 0.000000
Cl -2.276418 -0.972457 0.000000
Na -0.577465 -1.367776 -2.165851
Cl 0.632737 0.959846 -1.997559
Na 0.829153 -4.978312 0.000000
Cl -0.577465 -3.998573 -1.944618

C7l
Cl -1.618166 -1.481956 1.945242
Na -3.305576 -0.606076 -0.000000
Cl -1.618166 -1.481956 -1.945242
Na 0.217700 -1.730653 0.000000
Na -0.457551 -1.342019 4.252855
Cl 0.945906 0.762226 3.818150
Na 2.701131 0.336911 1.888911
Cl 1.227130 1.669778 0.000000
Na -0.766350 1.728130 -1.886463
Cl -2.648374 1.924381 -0.000000
Na -0.766350 1.728130 1.886463
Cl 2.852093 -1.578374 0.000000
Na 2.701131 0.336911 -1.888911
Na -0.457551 -1.342019 -4.252855
Cl 0.945906 0.762226 -3.818150

C7m
Na -4.043780 0.196582 0.902188
Cl -1.659575 1.378370 1.815451
Na -1.353966 2.024659 -0.910118
Cl -3.559170 0.812834 -1.648743
Na -2.550250 -1.634699 -1.731100
Cl -3.000787 -2.274543 0.817312
Na -0.780558 -1.154470 1.611580
Cl 2.028320 -0.636055 1.999035
Na 4.502081 -0.043308 1.197766
Cl 0.097042 -1.022933 -1.376963
Na 2.176096 -2.296124 -0.392737
Cl 4.188565 -0.823755 -1.235454
Na 2.149618 0.722669 -2.023092
Cl 1.244119 2.750191 -0.660636
Na 0.923057 1.900159 1.793692

C7n
Cl 0.093039 -1.298964 3.469053
Na 1.521754 -2.611525 1.761860
Na -1.835294 -1.895325 1.835216
Cl -1.939115 0.080807 0.000000
Na -1.884526 2.754784 0.000000
Cl -0.038012 3.331683 -1.892986
Na 0.692569 0.817298 -2.005826
Cl 0.093039 -1.298964 -3.469053
Na -1.835294 -1.895325 -1.835216
Na 0.692569 0.817298 2.005826
Na 1.521754 -2.611525 -1.761860
Cl 2.155632 -0.583170 -0.000000
Cl -0.417980 -3.500331 -0.000000
Na 1.267733 4.527353 0.000000
Cl -0.038012 3.331683 1.892986

C7o
Cl 1.659256 -2.782291 0.000000
Na 0.691583 -1.732013 2.376812
Cl -1.780244 -2.444866 -1.998261
Na -2.551724 -0.881449 0.000000
Cl -1.876188 1.689748 0.000000
Na -2.378279 4.312340 0.000000
Cl 0.213244 4.537879 0.000000
Na 0.361031 2.465539 1.716049
Cl 1.994798 0.549959 2.233061
Na 0.691583 -1.732013 -2.376812
Na 3.056557 -0.396682 0.000000
Cl 1.994798 0.549959 -2.233061
Na 0.361031 2.465539 -1.716049
Cl -1.780244 -2.444866 1.998261
Na -0.889248 -3.968885 0.000000

C7p
Na 2.461219 -1.737087 -0.638249
Cl 2.963295 0.410696 0.982775
Na 1.749576 1.743155 2.930947
Cl 0.000000 2.536131 0.096159
Na 2.340365 1.984002 -1.089246
Cl 2.305361 -0.075585 -2.766545
Cl -2.305361 0.075585 -2.766545
Na -2.461219 1.737087 -0.638249
Cl -2.963295 -0.410696 0.982775
Na -1.749576 -1.743155 2.930947
Cl -0.000000 -2.536131 0.096159
Na -2.340365 -1.984002 -1.089246
Na -0.000000 -0.000000 0.972755
Na 0.000000 0.000000 -3.841343
Cl -0.000000 -0.000000 3.673957

C7q
Na -1.095727 -3.627401 1.756721
Cl -3.091948 -3.703553 0.000000
Na -3.242656 -1.057228 0.000000
Na -1.095727 -3.627401 -1.756721
Cl 0.923788 -3.623147 0.000000
Na 0.658195 -0.985456 0.000000
Cl -3.333580 1.664731 0.000000
Na -1.474191 1.644924 -1.845986
Cl -1.095727 -0.926551 -2.139857
Na -1.474191 1.644924 1.845986
Cl -1.095727 -0.926551 2.139857
Cl 0.677158 1.928767 0.000000
Na 2.922854 3.150564 0.000000
Cl 5.206181 4.277466 0.000000
Na 7.598493 4.879825 0.000000

C7r
Cl 0.261734 -3.724504 1.944550
Na 1.480565 -2.051297 -0.000000
Na -1.134244 -1.537209 2.192899
Cl -2.216404 -0.560463 -0.000000
Na -0.479144 1.535264 0.000000
Na -1.134244 -1.537209 -2.192899
Cl 1.114966 0.009020 1.964603
Na 1.275622 2.196225 3.276990
Cl -0.354147 3.702846 1.994783
Na -0.523939 5.306306 0.000000
Cl -0.354147 3.702846 -1.994783
Na 1.275622 2.196225 -3.276990
Cl 1.114966 0.009020 -1.964603
Cl 0.261734 -3.724504 -1.944550
Na -0.495505 -5.203069 -0.000000

C7s
Cl -2.520163 1.475213 -0.000000
Na -2.379617 -0.500273 1.746375
Na -2.379617 -0.500273 -1.746375
Na 0.094734 1.941613 -0.000000
Cl -2.705429 -2.503603 0.000000
Na 0.710454 -2.235178 0.000000
Cl 0.295346 -0.518994 -2.079662
Na 1.727104 1.137216 -3.413780
Cl 1.727104 3.253188 -1.968891
Na 2.829735 4.487911 -0.000000
Cl 1.727104 3.253188 1.968891
Na 1.727104 1.137216 3.413780
Cl 0.295346 -0.518994 2.079662
Cl 0.785819 -4.786966 0.000000
Na -1.719640 -4.932007 0.000000

C7t
Na -1.203737 -1.235405 -2.007868
Cl -0.335947 1.203804 -1.424155
Cl 0.538646 -2.808639 -0.696177
Na 1.611098 0.192792 0.566341
Na -1.262351 -2.447025 1.339099
Cl -0.359761 -0.193513 2.290733
Na -2.036887 1.312450 0.875843
Cl -3.221005 -1.720492 -0.360897
Na 3.067493 -3.195201 -0.525680
Cl 4.132729 -1.021573 0.318010
Na 5.091544 1.382260 0.281300
Na -5.244830 -0.058876 -0.201706
Cl -4.382242 2.215939 0.409927
Na 1.058684 3.375478 -1.455142
Cl 2.928100 2.760286 0.192322

C7u
Na 2.515594 -1.290004 0.238638
Cl 2.961083 1.873090 -0.170870
Na 0.927880 1.737055 1.554583
Na 1.138029 1.283347 -2.059536
Cl 0.700423 -1.313085 -1.858087
Na -0.584566 -3.100465 -0.377954
Cl -3.104249 -2.544718 -0.343441
Na -5.248466 -1.343917 0.400478
Cl -4.349592 1.079645 0.641754
Na -3.291036 3.241688 -0.220608
Cl -0.824199 2.438458 -0.491552
Na -1.845858 -0.152642 0.084121
Cl 0.228367 -0.829863 1.726361
Cl 5.025971 -1.532188 0.616959
Na 5.402724 0.905596 0.193088

C7v
Na 1.851639 -0.763868 2.516463
Cl 0.103493 -2.771447 2.312478
Na -1.583510 -0.788573 2.007749
Na 1.223981 -3.211524 0.000000
Cl -3.194772 0.232294 0.000000
Na -1.583510 -0.788573 -2.007749
Cl 0.103493 -2.771447 -2.312478
Na 1.851639 -0.763868 -2.516463
Cl 0.103493 1.182523 -2.149216
Na 2.340243 1.952215 0.000000
Na -0.718861 2.705677 0.000000
Cl 2.919052 -0.820963 0.000000
Cl 0.103493 1.182523 2.149216
Na -5.614368 0.894900 0.000000
Cl 1.306467 4.260620 0.000000

C7w
Na 10.043533 6.617245 0.000000
Cl 8.350607 4.836921 0.000000
Na 5.978291 3.865084 0.000000
Cl 3.613007 2.982396 0.000000
Na 1.891162 1.155995 0.000000
Cl 0.090387 -0.676983 0.000000
Na -2.136392 -0.723712 1.779235
Na -2.136392 -0.723712 -1.779235
Cl -3.569593 0.644054 0.000000
Na -5.988258 -0.335448 0.000000
Cl -6.339466 -2.802347 0.000000
Na -4.006940 -3.863829 0.000000
Cl -2.136392 -3.320846 -1.940752
Cl -2.136392 -3.320846 1.940752
Na -0.356520 -3.429798 0.000000

C7x
Na 4.785647 -0.514231 0.000000
Cl 5.782009 -2.839941 0.000000
Na 4.075513 -4.678697 0.000000
Cl 2.297159 0.109201 0.000000
Cl 1.464562 -4.643436 0.000000
Na -0.812704 -5.913910 0.000000
Cl -3.157205 -5.016956 0.000000
Na -3.350609 -2.497532 0.000000
Cl -1.478536 -0.739649 0.000000
Na 0.813879 -2.054193 0.000000
Na -0.000000 1.567587 0.000000
Cl -0.452175 4.078601 0.000000
Na -2.259839 5.806545 0.000000
Cl -3.898646 7.790644 0.000000
Na -4.112965 10.234077 0.000000

C7y
Na 2.175570 -11.170815 0.000000
Cl 4.293266 -9.751622 0.000000
Na 3.780880 -7.307836 0.000000
Cl -0.228013 -10.319625 0.000000
Na -0.274598 -7.823462 0.000000
Cl 1.538284 -5.902888 0.000000
Na 1.304069 -3.311099 0.000000
Cl 1.339156 -0.820196 0.000000
Na 0.000000 1.345508 0.000000
Cl -1.459820 3.399966 0.000000

Na -1.303998 5.923288 0.000000
Cl -1.186819 8.456123 0.000000
Na -2.639504 10.501996 0.000000
Cl -3.867280 12.760671 0.000000
Na -3.705069 15.207755 0.000000

C8a
Cl 1.972566 -1.972566 1.428461
Na 0.000000 0.000000 1.680650
Cl 1.972566 1.972566 1.428461
Na 2.178688 2.178688 -1.379691
Cl -0.000000 3.619864 -1.543023
Na -2.178688 2.178688 -1.379691
Cl -1.972566 1.972566 1.428461
Na -0.000000 3.784771 1.136578
Na 3.784771 -0.000000 1.136578
Cl 3.619864 -0.000000 -1.543023
Na 2.178688 -2.178688 -1.379691
Cl -0.000000 -3.619864 -1.543023
Na -2.178688 -2.178688 -1.379691
Cl -3.619864 0.000000 -1.543023
Na -3.784771 0.000000 1.136578
Cl -1.972566 -1.972566 1.428461
Na -0.000000 -3.784771 1.136578

C8b
Na 0.190062 1.715201 -3.651309
Cl 0.000000 1.998529 -0.912622
Na 2.389725 -0.469184 -1.270250
Cl 2.003430 -0.203627 -3.830174
Na -2.389725 0.469184 -1.270250
Cl -0.000000 -1.998529 -0.912622
Na 0.000000 0.000000 1.015803
Cl 2.819980 0.472413 1.181984
Na 1.432944 2.865706 1.176444
Cl 0.122450 1.868047 3.303261
Na 1.954120 -0.210197 3.635247
Cl -0.122450 -1.868047 3.303261
Na -1.432944 -2.865706 1.176444
Cl -2.819980 -0.472413 1.181984
Na -1.954120 0.210197 3.635247
Na -0.190062 -1.715201 -3.651309
Cl -2.003430 0.203627 -3.830174

C8c
Na -0.497632 -1.037498 0.000000
Cl -2.663973 -0.617344 2.053446
Na -1.609688 1.911597 2.262554
Cl -0.226980 1.804185 0.000000
Na 2.078778 1.023806 -2.058160
Cl 3.691759 0.589777 -0.000000
Na 3.067764 -1.985723 -0.000000
Cl 0.249738 1.392956 -4.005265
Na -1.609688 1.911597 -2.262554
Cl -2.663973 -0.617344 -2.053446
Na -4.183146 -1.035222 0.000000
Cl 1.217913 -1.823455 1.965011
Na -0.491052 -1.136668 3.860571
Cl 0.249738 1.392956 4.005265
Na 2.078778 1.023806 2.058160
Na -0.491052 -1.136668 -3.860571
Cl 1.217913 -1.823455 -1.965011

C8d
Na -0.000000 0.000000 0.491863
Cl 1.929247 -0.575556 -3.539307
Na 0.543395 1.642166 -3.318032
Cl -0.000000 2.434005 -0.800051
Na 1.816313 2.654263 1.124383
Cl 2.767993 0.043751 0.927499
Na 2.625972 -1.522485 -1.214829
Cl -0.000000 -2.434005 -0.800051
Na -1.816313 -2.654263 1.124383
Cl -2.767993 -0.043751 0.927499
Na -0.543395 -1.642166 -3.318032
Cl -0.482281 -1.836047 3.257248
Na 1.820977 -0.543288 3.401491
Cl 0.482281 1.836047 3.257248
Na -1.820977 0.543288 3.401491
Cl -1.929247 0.575556 -3.539307
Na -2.625972 1.522485 -1.214829

C8e
Na -0.162373 0.845029 0.788526
Cl 0.593436 3.397224 0.388187
Na 2.977926 2.305946 0.094312
Cl 3.984014 0.549767 -1.648620
Na 1.897935 -0.852927 -2.365271
Cl -0.508823 -0.033064 -1.850898
Na -1.146772 -2.487350 -0.555737
Cl -3.767851 -2.021669 -0.907240
Na -3.797570 -0.656132 1.360060
Cl -3.295645 1.811089 0.241921
Na -1.878480 3.979747 0.082058
Cl 1.752770 -2.752764 -0.254908
Na 3.994410 -1.220659 0.274617
Cl 2.546973 0.256527 1.972300
Na -3.146400 0.288020 -1.993093
Na 1.220615 -2.010589 2.358316
Cl -1.278533 -1.330754 2.030926

C8f
Cl 0.094350 0.762554 2.236437
Na -1.692749 -1.405371 2.629654
Cl 0.135689 -3.275926 2.442681
Na -0.685735 -3.823288 -0.000000
Cl 0.135689 -3.275926 -2.442681
Na 1.934051 -1.409260 -2.123974
Cl 0.094350 0.762554 -2.236437
Na -1.692749 -1.405371 -2.629654
Cl -2.517620 -1.668494 -0.000000
Na -1.424093 0.737364 0.000000
Cl 1.347787 4.091779 0.000000
Na -0.526200 3.591945 -1.832995
Na 2.394688 1.748967 0.000000
Cl 3.285919 -0.689138 -0.000000
Na 1.934051 -1.409260 2.123974
Na -0.526200 3.591945 1.832995
Cl -2.391794 3.151751 0.000000

C8g
Na 1.405845 -1.855561 -1.086813
Cl 4.057731 -1.603496 -0.955196
Na 3.677734 -0.858517 1.551719
Cl 1.147206 -1.730165 1.700624
Na -1.423016 -2.391778 1.480405
Cl -2.430150 -0.359375 2.710106
Na 0.073225 0.723274 2.201793
Cl 2.971664 1.734876 1.048390
Na 3.731230 0.887771 -1.529715
Cl 1.164094 1.060197 -2.282858
Na 1.184670 3.043953 -0.412408
Cl -1.175997 2.500272 0.547373
Na -3.614150 1.343614 1.108241
Cl -1.392875 -2.121559 -1.225573
Na -1.434570 0.790069 -1.623878
Na -3.926512 -1.861199 -1.898369
Cl -4.131027 0.634668 -1.407614

C8h
Na 0.406253 -1.378553 2.701302
Cl 3.114951 -0.686607 2.640676
Na 2.461694 1.651261 3.482143
Cl 0.019556 1.868505 2.676755
Na -2.619142 1.008175 2.667553
Cl -2.914508 1.227501 -0.000000
Na -0.203911 1.467106 -0.000000
Cl 3.114951 -0.686607 -2.640676
Na 3.152473 -1.111044 0.000000
Cl 0.461354 -1.237202 0.000000
Na 0.406253 -1.378553 -2.701302
Cl -2.225433 -1.591207 -2.719254
Na -2.619142 1.008175 -2.667553
Cl -2.225433 -1.591207 2.719254
Na -2.464801 -1.637700 -0.000000
Na 2.461694 1.651261 -3.482143
Cl 0.019556 1.868505 -2.676755

C8i
Na -1.829489 -0.003751 1.270874
Cl -4.338088 -0.652131 0.592941
Na -3.039886 -2.539081 -0.635624
Cl -0.732487 -2.818938 0.771800
Na 0.579567 -1.803125 -1.324835
Cl 3.329463 -1.804138 0.775590
Na 2.679578 0.850749 0.808239
Cl 0.534441 0.526499 2.494329
Na 0.075088 3.249582 1.726248
Cl 1.996647 3.211574 -0.036945
Na 0.291119 1.957115 -1.751091
Cl -1.825668 2.535262 -0.011164
Na -3.703631 1.104646 -1.259031
Cl -1.669128 -0.561954 -2.130076
Na 1.226431 -2.121891 2.441116
Cl 2.277600 0.001885 -2.253772
Na 4.381472 -1.371246 -1.589165

C8j
Na -1.166896 1.598330 -1.712048
Cl 0.000000 4.044476 -0.992298
Na 1.894539 3.640437 -2.696876
Cl 1.721150 1.052661 -2.288123
Na 1.166896 -1.598330 -1.712048
Cl 1.652649 -1.202259 0.894951
Na 1.759441 1.423784 0.484624
Cl 1.256650 2.389679 2.881815
Na -0.671749 3.644658 1.609846
Cl -1.652649 1.202259 0.894951
Na 0.000000 0.000000 3.094754
Cl -1.256650 -2.389679 2.881815
Na 0.671749 -3.644658 1.609846
Cl -1.721150 -1.052661 -2.288123
Na -1.759441 -1.423784 0.484624
Na -1.894539 -3.640437 -2.696876
Cl -0.000000 -4.044476 -0.992298

C8k
Na -1.230820 0.911541 0.154368
Cl -2.610042 3.282597 -0.144172
Na -0.356040 4.428339 0.437643
Cl 1.214627 2.415138 0.823729
Na 2.404844 0.962962 -1.262088
Cl 0.149143 -0.467782 -1.741688
Na 0.765112 -2.434608 0.224411
Cl -1.437566 -3.826937 -0.342294
Na -2.802527 -2.339630 1.348861
Cl -3.763989 -0.618596 -0.611501
Na -4.648103 1.806322 -0.740787
Cl 3.019280 -1.348149 1.195501
Na 1.558165 0.375977 2.572931
Cl -0.830548 -0.769816 2.253496
Na -1.988134 -2.027468 -2.189773
Cl 4.903600 0.839530 -1.768540
Na 5.301451 -0.919958 -0.027115

C8l

Na 0.027822 0.869727 0.000000
Cl 2.857580 1.104417 0.000000
Na 1.760363 1.134105 2.702627
Cl 0.286539 -0.934797 2.071903
Na 1.801719 -2.066930 0.000000
Cl 0.286539 -0.934797 -2.071903
Na 1.760363 1.134105 -2.702627
Cl 0.286539 3.235559 -1.890594
Na -1.718569 3.470533 0.000000
Cl 0.286539 3.235559 1.890594
Na 2.108168 3.751916 0.000000
Cl -2.677432 0.976183 0.000000
Na -2.359817 -1.101908 -1.790808
Cl -2.896248 -2.970788 0.000000
Na -2.359817 -1.101908 1.790808
Cl 1.536284 -4.591680 0.000000
Na -0.968215 -4.729110 0.000000

C8m
Na -0.393597 1.627137 1.948134
Cl 0.042378 -1.132106 2.110575
Na 0.042378 -3.730699 1.916385
Cl -1.690877 -4.085324 0.000000
Na 0.042378 -3.730699 -1.916385
Cl 0.042378 -1.132106 -2.110575
Na 1.734348 -0.787499 0.000000
Cl 1.741678 1.828085 0.000000
Na -0.393597 1.627137 -1.948134
Cl -2.410043 1.233662 0.000000
Na -2.032610 -1.368738 0.000000
Cl -0.749453 4.257299 2.009928
Na -2.480552 3.964393 0.000000
Cl -0.749453 4.257299 -2.009928
Na 0.984466 4.448970 0.000000
Cl 2.339571 -3.677450 0.000000
Na 4.712686 -4.444466 0.000000

C8n
Na 1.291909 -0.178211 -0.000000
Cl 2.112389 -1.841759 2.049962
Na 1.559028 0.148470 3.549441
Cl 1.215370 2.276715 2.075354
Na -1.501984 2.123912 2.046980
Cl -1.415516 -0.507614 2.139690
Na -1.501984 2.123912 -2.046980
Cl -1.415516 -0.507614 -2.139690
Na -0.129765 -3.159359 -2.051738
Cl -1.585064 -3.884550 -0.000000
Na -0.129765 -3.159359 2.051738
Cl -1.818162 3.704940 0.000000
Na 0.890281 3.932429 0.000000
Cl 2.112389 -1.841759 -2.049962
Na -2.687787 -1.478105 -0.000000
Cl 1.215370 2.276715 -2.075354
Na 1.559028 0.148470 -3.549441

C8o
Na 0.570370 0.646189 0.000000
Cl 0.063806 3.762253 0.000000
Na -1.860228 3.018439 1.812820
Cl -1.178098 0.427129 2.073129
Na -1.178098 -2.215811 1.824853
Cl -3.053394 -2.516624 0.000000
Na -1.178098 -2.215811 -1.824853
Cl 0.895519 -2.496102 0.000000
Na 4.126545 -1.246387 0.000000
Cl 3.194366 1.186679 0.000000
Na 2.647259 3.705151 0.000000
Cl -1.178098 0.427129 -2.073129
Na -1.860228 3.018439 -1.812820
Cl -3.760424 2.750582 0.000000
Na -3.422256 0.127211 0.000000
Cl 4.803736 -3.686239 0.000000
Na 2.483278 -4.613031 0.000000

C8p
Na -2.651034 -0.984530 0.000000
Cl -1.345086 -3.340497 0.000000
Na 0.073800 -2.985412 2.155240
Cl -1.153062 -0.489760 2.581475
Na -1.645097 2.071039 2.069506
Cl 1.027397 2.511622 2.026891
Na 1.221401 -0.269016 -0.000000
Cl 2.330239 -1.608400 -2.065502
Na 0.073800 -2.985412 -2.155240
Cl -1.153062 -0.489760 -2.581475
Na 1.351939 0.282414 -3.518197
Cl 1.027397 2.511622 -2.026891
Na 2.007482 3.743339 -0.000000
Cl -3.154088 1.720362 0.000000
Na -1.645097 2.071039 -2.069506
Na 1.351939 0.282414 3.518197
Cl 2.330239 -1.608400 2.065502

6.2 Structures Cationic Clusters: ωB97XD/aug-cc-pVDZ

C1a
Na 0.000000 -0.000000 2.523338
Cl 0.000000 0.000000 0.000000
Na 0.000000 0.000000 -2.523338

C2a
Cl 0.000000 0.000000 1.757538
Na -0.000000 2.021445 -0.000000
Na -1.750623 -1.010723 -0.000000
Na 1.750623 -1.010723 -0.000000
Cl 0.000000 0.000000 -1.757538

C2b
Cl 0.000000 0.000000 2.586962
Na 0.000000 0.000000 5.073551
Na 0.000000 0.000000 0.000000
Cl 0.000000 0.000000 -2.586962
Na 0.000000 0.000000 -5.073551

C2c
Na -0.000000 1.770611 0.874703
Cl -0.000000 0.000000 2.661573
Na -0.000000 -1.770611 0.874703
Cl 0.000000 0.000000 -1.302555
Na 0.000000 0.000000 -3.849707

C3a
Cl 0.000000 2.293313 0.127876
Na 0.000000 0.000000 1.566539
Na 0.000000 -2.665341 -0.719806
Cl -1.986067 -1.146656 0.127876
Na -2.308253 1.332670 -0.719806
Na 2.308253 1.332670 -0.719806
Cl 1.986067 -1.146656 0.127876

C3b
Na 0.267255 -0.811519 -0.226046
Cl -2.417424 0.003310 -0.036237
Na -4.454043 -1.521021 0.101021
Cl 2.481607 -2.030603 0.035081
Na 3.482260 0.303463 0.145504
Cl 1.204261 1.753918 -0.076204
Na -1.255795 2.451566 0.099078

C3c
Na 0.000000 1.723767 3.039589
Cl 0.000000 0.000000 4.886898
Na -0.000000 -1.723767 3.039589
Cl 0.000000 0.000000 0.922463
Na -0.000000 -0.000000 -1.693293
Cl -0.000000 -0.000000 -4.270198
Na -0.000000 -0.000000 -6.764591

C3d
Na -0.000000 7.607263 -0.005807
Cl 0.011650 5.133393 -0.154397
Na -0.017976 2.536495 0.034327
Cl 0.000000 -0.000000 0.271887
Na 0.017976 -2.536495 0.034327
Cl -0.011650 -5.133393 -0.154397
Na -0.000000 -7.607263 -0.005807

C4a
Cl 0.000000 2.759279 0.390036
Na -2.574779 2.574765 0.000096
Cl -2.759173 0.000000 -0.390209
Na 0.000000 0.000000 0.000149
Na -2.574779 -2.574765 0.000096
Cl -0.000000 -2.759279 0.390036
Na 2.574779 -2.574765 0.000096
Cl 2.759173 -0.000000 -0.390209
Na 2.574779 2.574765 0.000096

C4b
Na -0.365994 0.745458 1.742544
Cl -0.365994 -1.882461 1.902633
Na -1.848530 -2.920032 -0.000000
Cl -0.365994 -1.882461 -1.902633
Na -0.365994 0.745458 -1.742544
Cl -1.276805 2.591388 0.000000
Na 1.566496 -1.753656 -0.000000
Na 1.176087 3.528914 0.000000
Cl 1.903926 0.949561 0.000000

C4c
Cl -0.747654 1.886156 -1.644453
Na 0.000000 3.535404 0.206022
Cl 1.527664 1.717476 1.395844
Na -0.000000 -0.000000 2.628725
Cl -1.527664 -1.717476 1.395844
Na -0.000000 -3.535404 0.206022
Na -1.588997 -0.590093 -1.136171
Na 1.588997 0.590093 -1.136171
Cl 0.747654 -1.886156 -1.644453

C4d
Na -0.000000 2.169723 0.105689
Cl 2.016481 1.164216 -1.222212
Na 1.879035 -1.084861 0.105689
Cl -0.000000 -2.328432 -1.222212
Na -1.879035 -1.084861 0.105689
Cl -2.016481 1.164216 -1.222212
Na 0.000000 0.000000 -2.638301
Cl 0.000000 0.000000 2.137574
Na 0.000000 0.000000 4.684332

C4e
Na 0.000000 1.173985 0.000000
Cl -1.764548 -0.825463 0.000000
Na -3.622222 0.915298 0.000000
Cl -2.038303 3.141354 0.000000

Na -1.936839 5.681578 0.000000
Cl 2.211356 -0.164474 0.000000
Na 0.480428 -2.417390 0.000000
Na 3.922597 -2.212722 0.000000
Cl 2.339517 -4.183666 0.000000

C4f
Na -1.460626 -1.204091 -0.000000
Cl -3.889323 0.136867 -0.000000
Na -2.416431 2.241198 -0.000000
Cl 0.000000 1.136319 0.000000
Na 2.604188 1.381952 0.000000
Cl -2.397786 -3.584077 -0.000000
Na -4.660065 -2.433153 -0.000000
Cl 5.176345 1.530208 0.000000
Na 7.649569 1.220602 0.000000

C4i
Cl 7.611859 0.059310 0.000000
Na 5.015111 0.287465 0.000000
Cl 2.496632 0.599929 0.000000
Na 0.000000 0.016822 0.000000
Cl -2.496741 -0.567584 0.000000
Na -5.017088 -0.266458 0.000000
Na 10.063396 0.399053 0.000000
Cl -7.616294 -0.060826 0.000000
Na -10.054398 -0.484527 0.000000

C5a
Na -2.090773 -0.921799 0.000000
Cl -0.662961 0.215828 2.057905
Na 0.742725 -2.285863 1.775920
Cl 2.496522 -1.361424 0.000000
Na 1.176374 0.896535 -0.000000
Cl -0.662961 0.215828 -2.057905
Na -0.662961 2.848157 -2.353780
Cl 0.300832 3.489678 -0.000000
Na -0.662961 2.848157 2.353780
Na 0.742725 -2.285863 -1.775920
Cl -0.982985 -3.271237 0.000000

C5b
Cl -0.000000 2.244458 0.000000
Na -1.727843 0.997571 1.794556
Na 1.727843 0.997571 1.794556
Na -1.727843 0.997571 -1.794556
Na 1.727843 0.997571 -1.794556
Cl 0.000000 0.000000 3.482239
Na -0.000000 -1.995141 1.794556
Cl 0.000000 -0.000000 -3.482239
Na -0.000000 -1.995141 -1.794556
Cl -1.943758 -1.122229 0.000000
Cl 1.943758 -1.122229 0.000000

C5c
Na 2.187488 -0.000000 0.236223
Cl 1.952625 2.585662 -0.403976
Na 0.000000 3.347861 -1.976658
Cl -1.952625 2.585662 -0.403976
Na -2.187488 0.000000 0.236223
Cl -1.952625 -2.585662 -0.403976
Na 0.000000 -2.642192 1.458241
Cl 0.000000 0.000000 1.981097
Na 0.000000 2.642192 1.458241
Cl 1.952625 -2.585662 -0.403976
Na -0.000000 -3.347861 -1.976658

C5e
Na 1.686731 1.165855 3.344636
Cl 0.000000 1.993080 -2.827461
Na 0.000000 0.000000 -4.500579
Cl -1.688240 -1.564828 0.737071
Na -1.686731 -1.165855 3.344636
Cl -0.000000 -1.993080 -2.827461
Na 1.512823 3.316924 -1.181990
Cl -0.000000 -0.000000 4.828464
Na -1.512823 -3.316924 -1.181990
Cl 1.688240 1.564828 0.737071
Na 0.000000 0.000000 -0.825679

C5f
Na 0.126532 3.394574 -0.786774
Cl 2.438291 2.254844 -0.498616
Na 4.462602 0.827661 0.377091
Cl -1.474802 1.502552 0.129300
Cl 3.211546 -1.355831 1.051475
Na 1.984564 -3.410225 -0.038481
Cl -0.065376 -2.161511 -1.034274
Na -2.550379 -1.244068 -0.882252
Na 0.964518 0.016193 0.173715
Cl -4.784950 -0.825139 0.281342
Na -3.944208 1.320091 1.266078

C5g
Na 0.000000 0.000000 -4.162486
Cl 2.019577 1.166004 -2.771365
Na -0.000000 2.120338 -1.386905
Cl -2.019577 1.166004 -2.771365
Na -1.836266 -1.060169 -1.386905
Cl 0.000000 0.000000 0.555827
Na 0.000000 0.000000 3.176591
Cl 0.000000 0.000000 5.751345
Na 0.000000 0.000000 8.248220
Na 1.836266 -1.060169 -1.386905
Cl -0.000000 -2.332007 -2.771365

C5i
Cl 0.573849 -1.886249 0.523798
Na -1.301810 -3.562912 -0.021734
Cl -3.304865 -1.961829 -0.530414
Na -1.338150 -0.009956 -0.005290
Na -4.955951 -0.043854 0.059935
Cl -3.326555 1.904410 0.600534
Na -1.389832 3.553600 -0.012625
Cl 0.491463 1.909593 -0.626313
Na 2.375997 0.054793 -0.058374
Cl 5.016784 0.138340 -0.030402
Na 7.458702 -0.152808 0.135140

C5j
Na 4.168600 0.623415 0.004143
Cl 6.487308 -0.496524 -0.004432
Na 5.172331 -2.655049 -0.003633
Cl 2.668610 -1.773903 0.005788
Na 0.626478 -0.161140 0.000699
Cl 1.937909 2.188664 0.001659
Na -0.385436 3.259953 -0.000727
Cl -1.868273 1.041365 -0.001835
Na -4.311304 0.116496 -0.001532
Cl -6.759750 -0.690591 -0.000709
Na -9.081458 -1.599420 0.000321

C5k
Na -3.526200 0.769843 -0.000198
Cl -4.607963 3.095850 -0.000122
Na -6.792521 1.806573 0.000303
Cl -5.889210 -0.710434 0.000151
Na -4.274736 -2.715444 -0.000117
Cl -1.945674 -1.467000 -0.000167
Na 0.688319 -1.494914 -0.000120
Cl 3.219497 -1.328636 0.000177
Na 5.565242 -0.314201 0.000175
Cl 7.942267 0.737819 -0.000043
Na 10.319751 1.441856 -0.000037

C6a
Na 0.000000 2.899951 1.335582
Cl -2.149027 1.240741 1.395717
Na -2.511431 -1.449976 1.335582
Cl -2.149027 -1.240741 -1.395717
Na -2.511431 1.449976 -1.335582
Cl -0.000000 2.481482 -1.395717
Na 2.511431 1.449976 -1.335582
Cl 2.149027 1.240741 1.395717
Na 2.511431 -1.449976 1.335582
Cl 2.149027 -1.240741 -1.395717
Na 0.000000 0.000000 0.000000
Cl -0.000000 -2.481482 1.395717
Na -0.000000 -2.899951 -1.335582

C6b
Na 0.000000 0.000000 2.733929
Cl 2.034663 1.174713 1.456234
Na 2.115207 1.221215 -1.453103
Cl -0.000000 2.785963 -1.693114
Na -2.115207 1.221215 -1.453103
Cl -2.412715 -1.392982 -1.693114
Na -0.000000 -2.442431 -1.453103
Cl -0.000000 -2.349427 1.456234
Na -0.000000 2.978990 0.907880
Cl -2.034663 1.174713 1.456234
Na -2.579881 -1.489495 0.907880
Cl 2.412715 -1.392982 -1.693114
Na 2.579881 -1.489495 0.907880

C6c
Na 2.151013 1.806011 0.295867
Cl -0.000000 3.228703 -0.314179
Na -2.151013 1.806011 0.295867
Na -0.000000 1.778636 -2.498519
Cl 1.981005 -0.000000 -1.856945
Cl -1.981005 0.000000 -1.856945
Cl 2.240899 -0.000000 2.245553
Na -0.000000 -1.778636 -2.498519
Cl -0.000000 -3.228703 -0.314179
Na -2.151013 -1.806011 0.295867
Na 2.151013 -1.806011 0.295867
Cl -2.240899 0.000000 2.245553
Na -0.000000 0.000000 3.583518

C6d
Na 1.824370 2.611928 0.269576
Cl 0.000000 2.672780 2.112079
Na 0.000000 -0.000000 1.946420
Cl -2.562325 0.000000 0.257829
Na -1.675037 0.000000 -2.220054
Cl 0.000000 2.115740 -1.911989
Na 1.675037 -0.000000 -2.220054
Cl -0.000000 -2.115740 -1.911989
Na -1.824370 -2.611928 0.269576
Cl -0.000000 -2.672780 2.112079
Na 1.824370 -2.611928 0.269576
Na -1.824370 2.611928 0.269576
Cl 2.562325 -0.000000 0.257829

C6e
Na 2.780250 0.720688 -1.364037
Cl 2.595781 -1.868802 -1.191258
Na 2.373505 -1.589667 1.393802
Cl -0.325924 -1.933007 1.683897
Na -2.743518 -2.658462 0.818107
Cl -2.701867 -1.346768 -1.406045
Na -2.426602 1.295456 -1.289887
Cl -1.923966 2.371896 1.118510
Na 0.424095 3.049481 0.140218
Cl 0.258793 1.463371 -2.034576

Na -0.014104 -1.306829 -1.322570
Cl 2.102240 1.211069 1.344711
Na -0.401442 0.647344 2.373542

C6f
Cl -0.571746 0.271940 1.933223
Na -0.571746 3.165019 1.760260
Cl -2.571947 3.261043 0.000000
Na -0.571746 3.165019 -1.760260
Cl -0.571746 0.271940 -1.933223
Na 1.482602 0.692390 0.000000
Cl 1.414132 3.304366 0.000000
Na 0.169189 -2.255452 1.747067
Cl 2.362397 -1.936066 0.000000
Na 2.244894 -4.616551 0.000000
Cl -0.358567 -4.244863 0.000000
Na 0.169189 -2.255452 -1.747067
Na -2.462647 0.670287 0.000000

C6g
Na 0.112431 1.009052 2.314095
Cl 0.112431 -1.640032 2.467895
Na -0.475787 -4.173449 2.413047
Cl -1.459651 -4.386747 -0.000000
Na -1.084588 -1.632867 -0.000000
Cl -1.409517 1.123521 0.000000
Na -0.618980 3.689287 0.000000
Cl 1.182636 3.441126 1.986087
Na 2.861514 3.748522 0.000000
Cl 1.182636 3.441126 -1.986087
Na 0.112431 1.009052 -2.314095
Cl 0.112431 -1.640032 -2.467895
Na -0.475787 -4.173449 -2.413047

C6h
Na -1.633071 -1.941269 1.467229
Cl -3.774743 -1.554987 0.057586
Na -2.029312 -0.692021 -1.775036
Cl 0.196759 -2.060785 -0.611040
Na 2.663514 -2.940030 -1.001947
Cl 3.971964 -1.128487 0.319182
Na 4.604881 1.369019 -0.246155
Cl 2.228489 2.405350 -0.104395
Na -0.394902 2.761731 0.002045
Cl -0.979423 0.688278 1.863048
Na 1.495195 -0.006760 0.836504
Na -3.565318 0.997386 0.687116
Cl -2.381331 1.943065 -1.504812

C6i
Na 1.881721 -1.906150 1.712011
Cl -0.779871 -1.146137 2.061202
Na -1.102211 -2.908153 0.000000
Cl -0.779871 -1.146137 -2.061202
Na 0.005214 0.950329 -0.000000
Cl -0.779871 2.877097 1.979815
Na -1.472638 0.923042 -3.505541
Cl -0.779871 2.877097 -1.979815
Na -1.472638 0.923042 3.505541
Cl 1.338242 -3.869329 0.000000
Na -0.866706 4.553423 -0.000000
Cl 2.522474 0.000161 0.000000
Na 1.881721 -1.906150 -1.712011

C6j
Na 4.393851 1.210307 -1.268599
Cl 2.507112 2.188191 0.241696
Na 3.037444 0.102794 1.857753
Cl 4.234124 -1.185515 -0.219296
Cl -0.578552 -1.029856 -1.724161
Na 0.000198 1.259762 -0.000826
Cl -2.506717 2.187657 -0.242926
Na -3.037771 0.101546 -1.857642
Na -4.393990 1.211359 1.267813
Cl -4.234311 -1.185301 0.220426
Na -1.735273 -1.901324 0.641608
Na 1.735354 -1.903348 -0.640248
Cl 0.578465 -1.027650 1.724351

C6k
Na 0.843847 -3.558800 2.256273
Cl -0.258463 -4.299558 -0.000000
Na 0.843847 -3.558800 -2.256273
Cl 0.380999 -0.994590 2.122525
Na -1.144943 1.288811 2.487998
Cl 0.843847 2.928446 1.998023
Na 0.745217 4.569303 0.000000
Cl 0.843847 2.928446 -1.998023
Na -1.144943 1.288811 -2.487998
Cl 0.380999 -0.994590 -2.122525
Na 1.001047 1.012588 0.000000
Cl -2.136579 0.853605 0.000000
Na -1.228530 -1.693726 -0.000000

C6l
Na 2.980686 -1.645105 0.291571
Cl 4.009270 0.741921 0.208704
Na 2.304278 0.972743 -1.818673
Cl 0.457468 2.369511 -0.311837
Na -1.860177 3.647235 -0.251359
Cl -3.336655 1.601295 0.245139
Na -4.651132 -0.661272 0.194975
Cl -2.684122 -2.248635 -0.313079
Na -0.258540 -3.092815 0.032643
Cl 0.782117 -1.241182 1.859622
Na -1.031804 -0.038735 -0.150028
Na 1.913522 1.244144 1.751054
Cl 1.162208 -1.498684 -1.721021

C6n
Na -0.167527 -2.962890 1.741694
Cl -2.168164 -3.001064 0.000000
Cl 1.867795 -2.954683 0.000000
Na 1.535823 -0.333475 0.000000
Cl -0.167527 -0.222159 -2.115937
Na -2.153501 -0.333312 0.000000
Na -0.167527 -2.962890 -1.741694
Cl -0.167527 -0.222159 2.115937
Na -0.531478 2.364016 1.850580
Cl 1.734051 2.664654 0.000000
Na 3.960773 3.899272 0.000000
Cl -2.357213 2.418817 0.000000
Na -0.531478 2.364016 -1.850580

C6o
Cl 4.070787 -0.926931 -0.919818
Na 2.448425 -2.295664 0.701652
Na 1.912928 -0.150931 -2.149525
Na 3.647494 1.033945 0.823752
Cl 2.107858 -0.341931 2.493580
Na 0.042748 0.387802 1.066391
Cl 0.093787 -1.657030 -0.793359
Na -2.589314 -1.492183 -0.880727
Cl -2.408213 1.412652 0.272441
Na -0.909877 3.309209 -0.810701
Cl 1.433498 2.148023 -0.684575
Na -4.970139 0.673927 0.935839
Cl -5.027418 -1.583439 -0.165532

C6p
Na 1.465974 -2.508627 1.724505
Cl 2.760900 -0.686605 0.000000
Cl -0.583618 -0.687484 1.954474
Cl 0.429206 -4.164160 0.000000
Na -1.543469 -2.424976 0.000000
Na 1.465974 -2.508627 -1.724505
Cl -0.583618 -0.687484 -1.954474
Na -1.669703 1.121074 0.000000
Cl 0.614845 2.895861 0.000000
Na 1.465974 1.050848 1.724647
Na 1.465974 1.050848 -1.724647
Na -1.509437 4.697601 0.000000
Cl -3.376195 3.020487 0.000000

C6r
Na -0.280008 3.615690 -0.187199
Cl -1.733880 1.782250 0.925793
Cl 1.692255 2.187471 -1.097455
Na 4.001091 0.954153 -0.726642
Cl 2.587925 -1.610966 0.136581
Na 0.780163 -3.201676 -0.972286
Cl -1.353819 -1.805716 -0.774884
Na -3.374810 -0.188246 0.066968
Cl -5.999761 -0.448513 0.035539
Na -8.389389 -0.262068 0.618227
Na 0.290946 0.170964 0.076778
Na 4.950774 -1.731620 1.439865
Cl 6.115137 0.311404 0.570142

C6s
Cl 0.001019 3.448086 -0.000000
Na -0.000397 0.742325 -0.000000
Cl -0.000632 -1.067580 1.958819
Cl 0.000534 2.674199 4.598859
Na 0.000534 0.151047 4.264983
Na 0.001855 4.160125 2.551024
Na 0.001855 4.160125 -2.551024
Cl 0.000534 2.674199 -4.598859
Na 0.000534 0.151047 -4.264983
Cl -0.000632 -1.067580 -1.958819
Na 0.000943 -2.955218 0.000000
Cl 0.005680 -5.592159 0.000000
Na -0.015375 -8.061798 0.000000

C6t
Na 12.195509 -1.387567 -0.000263
Cl 9.773076 -0.855439 -0.000198
Na 7.257341 -0.201786 0.000104
Cl 4.786854 0.450832 0.000337
Na 2.283399 0.870603 0.000146
Cl -0.279677 1.452551 -0.000115
Na -2.021926 3.454254 -0.000228
Cl -4.200740 2.108975 0.000038
Na -2.586018 -0.052448 0.000259
Cl -4.407630 -1.917485 0.000337
Na -6.203414 0.268698 0.000067
Cl -8.358094 -1.138862 -0.000403
Na -6.773474 -3.107183 -0.000078

C6u
Na -8.332777 0.240086 0.018525
Cl -5.807842 0.031453 0.191064
Na -3.401197 -0.842602 0.128590
Cl -0.968595 -1.668362 0.049905
Na 1.552842 -1.502834 -0.008951
Cl 4.184886 -1.327740 -0.054885
Na 6.517318 -2.553294 -0.065761
Cl 8.119322 -0.537880 -0.047932
Na 5.737552 0.928324 -0.032630
Cl 6.811843 3.259133 0.026026
Na 9.001431 1.982167 0.013178
Cl -10.917847 0.534906 -0.136164
Na -13.272444 1.297637 -0.096244

C7a
Cl 0.000000 2.391102 1.084222

Na 1.818204 1.049741 2.629911
Na -1.818204 1.049741 2.629911
Na 0.000000 0.000000 -0.341706
Cl 0.000000 0.000000 4.248422
Na -0.000000 -2.099481 2.629911
Cl -2.070755 -1.195551 1.084222
Na -2.544641 -1.469149 -1.544241
Cl -1.902929 1.098656 -2.302877
Na 0.000000 0.000000 -3.830923
Cl 1.902929 1.098656 -2.302877
Na 2.544641 -1.469149 -1.544241
Cl 2.070755 -1.195551 1.084222
Na 0.000000 2.938299 -1.544241
Cl -0.000000 -2.197313 -2.302877

C7b
Na 0.000000 0.000000 -2.729812
Cl 0.000000 2.685987 -2.903485
Na -1.798473 -2.656562 -0.992576
Cl -0.000000 -0.000000 3.874193
Na 0.000000 2.594638 3.695896
Cl 2.082985 -0.000000 -0.707599
Na 1.798473 2.656562 -0.992576
Cl -0.000000 -2.959785 1.102542
Na -0.000000 -0.000000 1.074610
Cl -0.000000 -2.685987 -2.903485
Na -0.000000 -2.594638 3.695896
Cl -2.082985 0.000000 -0.707599
Na -1.798473 2.656562 -0.992576
Cl 0.000000 2.959785 1.102542
Na 1.798473 -2.656562 -0.992576

C7c
Na -2.276136 -1.831312 -1.859051
Cl -3.754559 -1.212273 0.281603
Na -1.641713 -2.057986 1.583939
Na -3.072225 1.302259 -0.037545
Cl 0.617926 -0.677909 2.160414
Na 0.547909 2.017593 2.032554
Cl -1.552297 3.221736 0.950173
Na -0.195513 3.131651 -1.335347
Cl 2.115351 2.258053 -0.223636
Na 0.975776 -0.394801 -0.752696
Na 2.024624 -2.830938 0.992698
Cl -1.202562 0.699886 -2.048623
Cl -0.232315 -3.016132 -0.532278
Cl 3.513325 -1.454051 -0.679901
Na 4.402480 0.942783 -0.481988

C7d
Cl -2.114848 2.256607 -0.219708
Na -2.023188 -2.830503 0.992990
Cl 0.232578 -3.018036 -0.535420
Na 1.643747 -2.067739 1.583279
Cl 3.753648 -1.212229 0.281365
Na 2.273932 -1.824362 -1.861303
Cl 1.200199 0.703106 -2.050729
Na 0.193257 3.134719 -1.333471
Cl 1.552864 3.225138 0.950911
Na 3.066327 1.301007 -0.037041
Cl -0.611907 -0.679604 2.157027
Na -0.544183 2.015836 2.033683
Na -0.976083 -0.395758 -0.755292
Na -4.404142 0.942694 -0.478719
Cl -3.514083 -1.453503 -0.676704

C7e
Cl 0.511661 3.722965 2.026533
Na -1.182477 4.079443 0.000000
Na 2.191477 3.314816 0.000000
Cl 0.511661 3.722965 -2.026533
Na -0.067419 1.136274 -1.886809
Cl 1.898939 0.562331 0.000000
Cl -2.099374 1.464749 0.000000
Na -0.067419 1.136274 1.886809
Na 1.144224 -1.947912 -0.000000
Cl -0.741527 -1.753934 -2.065822
Na -2.400701 -1.118628 -0.000000
Cl -0.741527 -1.753934 2.065822
Na -0.067419 -4.293321 -2.371351
Cl 0.994794 -4.679840 -0.000000
Na -0.067419 -4.293321 2.371351

C7f
Na -2.405010 2.640051 0.000000
Cl -1.692149 -0.078399 0.000000
Cl -0.783593 3.247857 2.004974
Na 0.809102 4.046372 0.000000
Cl 2.312789 1.693886 0.000000
Na -1.512748 -2.199011 1.767987
Cl -1.612122 -4.134216 0.000000
Na 0.974510 -3.803511 0.000000
Cl 1.228254 -1.819829 -1.937165
Na 2.915615 -0.848830 0.000000
Cl 1.228254 -1.819829 1.937165
Na 0.444582 0.921308 1.825800
Na -1.512748 -2.199011 -1.767987
Cl -0.783593 3.247857 -2.004974
Na 0.444582 0.921308 -1.825800

C7i
Na -2.430870 -2.459988 1.059060
Cl -3.596063 -1.399505 -1.052520
Na -1.061364 -0.561796 -1.511162
Cl -1.731841 0.100992 2.074904
Cl 0.004725 -2.622658 0.002681
Na 2.435743 -2.450110 -1.062506
Cl 3.604079 -1.390571 1.047472
Na 3.719738 0.872016 -0.168178
Cl 1.737736 2.326501 0.970700
Na 1.067592 -0.562556 1.522014
Na -0.828844 2.573850 1.592090
Cl -1.747186 2.330262 -0.967131
Na 0.819071 2.573735 -1.587261
Cl 1.729723 0.106649 -2.080782
Na -3.722880 0.862268 0.163169

C7j
Na -4.406173 0.028099 1.719752
Cl -2.046391 1.193383 1.622845
Cl -4.221640 -1.335940 -0.511223
Na -2.371531 -3.111508 -0.947382
Cl -0.214876 -1.571815 -0.861676
Na -0.508974 3.236156 0.468022
Cl 1.796357 1.981456 0.926250
Na 3.917732 0.266711 1.016068
Cl 2.508962 -1.790384 2.042042
Na 2.441904 -2.453466 -0.508901
Cl -0.775286 2.417912 -2.035257
Na -2.204183 0.432627 -1.110708
Na 0.389450 -0.228598 1.685176
Cl 3.719145 -0.431548 -1.564489
Na 1.557538 1.114333 -1.732423

C7k
Cl 3.060928 3.593639 -0.000000
Na 2.518626 1.055768 -0.000000
Na 1.073201 3.798956 1.769945
Na 1.073201 3.798956 -1.769945
Cl -0.869897 4.189811 -0.000000
Na -1.220590 1.594432 -0.000000
Cl 0.677770 0.955175 1.998991
Na -0.687977 -1.346616 2.210128
Cl -0.687977 -4.010402 1.957375
Na -2.499237 -3.588633 0.000000
Cl -2.300622 -0.907114 0.000000
Na -0.687977 -1.346616 -2.210128
Cl 0.677770 0.955175 -1.998991
Na 0.631670 -5.149879 0.000000
Cl -0.687977 -4.010402 -1.957375

C7l
Cl -1.522640 -1.438217 1.979930
Na -3.293815 -0.795153 0.000000
Cl -1.522640 -1.438217 -1.979930
Na 0.369932 -1.366901 -0.000000
Na -0.376380 -1.361442 4.356479
Cl 0.946714 0.798739 3.849545
Na 2.723047 0.473552 1.892546
Cl 1.020101 1.542999 -0.000000
Na -0.954322 1.485352 -1.923270
Cl -2.826063 1.776269 0.000000
Na -0.954322 1.485352 1.923270
Cl 3.047880 -1.414521 -0.000000
Na 2.723047 0.473552 -1.892546
Na -0.376380 -1.361442 -4.356479
Cl 0.946714 0.798739 -3.849545

C7m
Na -4.042841 0.808347 0.003730
Cl -1.692631 2.312890 0.000009
Na -0.920962 0.445001 -1.828409
Cl -3.249187 -0.781930 -2.003951
Na -2.637207 -2.405012 -0.001884
Cl -3.244043 -0.786336 2.005920
Na -0.919010 0.443699 1.824148
Cl 1.818094 1.226928 1.939510
Na 3.798191 0.971147 0.002845
Cl 0.079112 -1.692025 -0.004797
Na 2.193961 -1.515642 1.767991
Cl 4.130720 -1.613355 0.000572
Na 2.200938 -1.509394 -1.772550
Cl 1.820050 1.230718 -1.936512
Na 0.849117 2.921205 0.002968

C7n
Cl 0.059372 -1.332634 3.488740
Na 1.550840 -2.580587 1.772712
Na -1.813821 -1.975584 1.816078
Cl -1.812234 0.116279 -0.000000
Na -1.838783 2.874970 -0.000000
Cl -0.026827 3.369409 -1.908692
Na 0.510486 0.767024 -1.918722
Cl 0.059372 -1.332634 -3.488740
Na -1.813821 -1.975584 -1.816078
Na 0.510486 0.767024 1.918722
Na 1.550840 -2.580587 -1.772712
Cl 2.152040 -0.570111 0.000000
Cl -0.386943 -3.517971 0.000000
Na 1.316026 4.546080 -0.000000
Cl -0.026827 3.369409 1.908692

C7p
Na 2.479292 -1.793437 -0.538669
Cl 2.859520 0.399277 1.000353
Na 1.466371 1.981138 2.762397
Cl 0.000000 2.647828 0.263119
Na 2.224532 1.890060 -1.100980
Cl 2.121174 -0.217019 -2.749472
Cl -2.121174 0.217019 -2.749472
Na -2.479292 1.793437 -0.538669
Cl -2.859520 -0.399277 1.000353
Na -1.466371 -1.981138 2.762397
Cl -0.000000 -2.647828 0.263119
Na -2.224532 -1.890060 -1.100980
Na -0.000000 -0.000000 0.934961

Na 0.000000 0.000000 -4.214525
Cl -0.000000 -0.000000 3.641103

C7q
Na -1.161800 -3.671731 1.729626
Cl -3.172377 -3.725083 0.000000
Na -3.123012 -1.046582 0.000000
Na -1.161800 -3.671731 -1.729626
Cl 0.878345 -3.680355 0.000000
Na 0.567324 -1.038107 0.000000
Cl -3.333647 1.695061 0.000000
Na -1.448973 1.643590 -1.806154
Cl -1.161800 -0.958482 -2.120621
Na -1.448973 1.643590 1.806154
Cl -1.161800 -0.958482 2.120621
Cl 0.734708 1.889215 0.000000
Na 3.030967 3.147715 0.000000
Cl 5.334093 4.300215 0.000000
Na 7.655552 5.215481 0.000000

C7r
Cl 0.185629 -3.732678 1.993524
Na 1.162200 -2.026938 -0.000000
Na -1.061858 -1.456121 2.374622
Cl -1.975083 -0.669850 -0.000000
Na -0.274579 1.494357 0.000000
Na -1.061858 -1.456121 -2.374622
Cl 1.211999 0.049373 2.023885
Na 1.381891 2.299628 3.283637
Cl -0.410849 3.640655 1.986928
Na -0.968347 5.201512 0.000000
Cl -0.410849 3.640655 -1.986928
Na 1.381891 2.299628 -3.283637
Cl 1.211999 0.049373 -2.023885
Cl 0.185629 -3.732678 -1.993524
Na -0.556988 -5.188895 -0.000000

C7s
Cl -2.808094 0.692261 -0.000000
Na -2.048275 -1.125784 1.732259
Na -2.048275 -1.125784 -1.732259
Na -0.291376 1.714593 0.000000
Cl -1.606087 -3.176895 -0.000000
Na 1.200739 -2.146453 -0.000000
Cl 0.528993 -0.343282 -2.025396
Na 1.370659 1.690426 -3.426283
Cl 0.528993 3.652497 -1.981562
Na 0.672512 5.317031 0.000000
Cl 0.528993 3.652497 1.981562
Na 1.370659 1.690426 3.426283
Cl 0.528993 -0.343282 2.025396
Cl 2.226851 -4.514750 -0.000000
Na -0.116365 -5.425710 -0.000000

C7u
Na -2.485212 -0.996880 -0.035252
Cl -3.005983 1.806771 -0.020975
Na -0.921526 1.644675 -1.760750
Na -1.046270 1.619580 1.857163
Cl -0.416940 -0.961369 1.826109
Na 0.330443 -2.983619 0.045521
Cl 2.918839 -2.718603 0.146743
Na 5.199270 -1.576239 -0.166034
Cl 4.424286 0.930539 -0.160427
Na 3.395814 3.274945 0.033205
Cl 0.843079 2.589699 0.116416
Na 1.811744 -0.176182 0.079014
Cl -0.305108 -0.941293 -1.772737
Cl -4.951155 -1.707030 -0.105808
Na -5.522382 0.741164 -0.098185

C7w
Na 10.777621 6.245206 0.000000
Cl 8.692890 4.900226 0.000000
Na 6.387104 3.703890 0.000000
Cl 4.119126 2.515741 0.000000
Na 2.041355 1.066403 0.000000
Cl -0.069691 -0.511835 0.000000
Na -2.312355 -0.611514 1.754900
Na -2.312355 -0.611514 -1.754900
Cl -3.848969 0.691262 0.000000
Na -6.307970 -0.290842 0.000000
Cl -6.563749 -2.784536 0.000000
Na -4.163081 -3.737645 0.000000
Cl -2.312355 -3.213263 -1.963140
Cl -2.312355 -3.213263 1.963140
Na -0.563343 -3.267042 0.000000

C7x
Na -1.872976 -4.266242 0.000160
Cl -4.402853 -4.586695 -0.000468
Na -5.716984 -2.424172 -0.000463
Cl -0.661600 -1.967101 0.000522
Cl -5.012753 0.130134 -0.000180
Na -5.623685 2.703949 -0.000383
Cl -4.043846 4.680607 -0.000309
Na -1.525096 4.280367 0.000111
Cl -0.383490 1.946356 0.000330
Na -2.305326 0.109580 0.000246
Na 1.391919 -0.153106 0.000598
Cl 3.960266 -0.476586 0.000586
Na 6.457780 -0.071429 0.000420
Cl 9.051485 0.232026 0.000056
Na 11.501408 -0.115182 -0.001518

C8a
Cl 1.984842 -1.984842 1.437249
Na 0.000000 0.000000 1.720112
Cl 1.984842 1.984842 1.437249
Na 2.242484 2.242484 -1.347846
Cl -0.000000 3.641586 -1.559198
Na -2.242484 2.242484 -1.347846
Cl -1.984842 1.984842 1.437249
Na -0.000000 3.781075 1.106283
Na 3.781075 -0.000000 1.106283
Cl 3.641586 -0.000000 -1.559198
Na 2.242484 -2.242484 -1.347846
Cl -0.000000 -3.641586 -1.559198
Na -2.242484 -2.242484 -1.347846
Cl -3.641586 0.000000 -1.559198
Na -3.781075 0.000000 1.106283
Cl -1.984842 -1.984842 1.437249
Na -0.000000 -3.781075 1.106283

C8b
Na 0.019176 1.722181 -3.780286
Cl -0.000000 1.926951 -0.944001
Na 2.180542 -0.082844 -1.322603
Cl 2.015712 -0.021522 -3.935148
Na -2.180542 0.082844 -1.322603
Cl 0.000000 -1.926951 -0.944001
Na 0.000000 0.000000 1.170813
Cl 2.771326 0.592903 1.242769
Na 1.330423 2.917444 1.230393
Cl -0.007028 1.876512 3.346523
Na 1.955301 -0.039894 3.735048
Cl 0.007028 -1.876512 3.346523
Na -1.330423 -2.917444 1.230393
Cl -2.771326 -0.592903 1.242769
Na -1.955301 0.039894 3.735048
Na -0.019176 -1.722181 -3.780286
Cl -2.015712 0.021522 -3.935148

C8c
Na -0.566451 -0.956127 0.000000
Cl -2.659496 -0.540155 2.016518
Na -1.508946 1.963166 2.288648
Cl -0.083463 1.802088 0.000000
Na 2.010850 0.895293 -2.000703
Cl 3.735167 0.506640 -0.000000
Na 3.017467 -2.029114 -0.000000
Cl 0.300286 1.400403 -4.051309
Na -1.508946 1.963166 -2.288648
Cl -2.659496 -0.540155 -2.016518
Na -4.239477 -1.121817 0.000000
Cl 1.144982 -1.832295 1.952231
Na -0.552911 -1.086693 3.856295
Cl 0.300286 1.400403 4.051309
Na 2.010850 0.895293 2.000703
Na -0.552911 -1.086693 -3.856295
Cl 1.144982 -1.832295 -1.952231

C8e
Na -0.715057 0.984056 1.065432
Cl -0.378448 3.565485 0.268942
Na 1.867970 2.287296 -0.517163
Cl 3.999435 1.002643 -1.558195
Na 2.472120 -1.009579 -2.193843
Cl 0.067435 0.069590 -1.605040
Na -0.415698 -2.459193 -0.404493
Cl -2.899970 -2.585007 -1.403413
Na -3.696288 -1.514937 0.885472
Cl -3.518768 1.130650 0.069425
Na -2.927962 3.729435 0.122327
Cl 2.377973 -2.509357 0.145149
Na 4.042014 -0.280732 0.711209
Cl 2.000473 0.873836 1.997653
Na -2.558055 -0.092161 -2.173943
Na 1.335043 -1.640361 2.542122
Cl -1.261894 -1.552414 2.059829

C8f
Cl 0.070286 0.776856 2.133485
Na -1.686946 -1.380827 2.586645
Cl 0.061663 -3.318171 2.500607
Na -0.721293 -3.766168 0.000000
Cl 0.061663 -3.318171 -2.500607
Na 1.948316 -1.507927 -2.237922
Cl 0.070286 0.776856 -2.133485
Na -1.686946 -1.380827 -2.586645
Cl -2.568615 -1.590009 0.000000
Na -1.677264 0.940116 -0.000000
Cl 1.636115 3.991134 -0.000000
Na -0.288110 3.692696 -1.795526
Na 2.013322 1.418202 -0.000000
Cl 3.194724 -0.921899 0.000000
Na 1.948316 -1.507927 2.237922
Na -0.288110 3.692696 1.795526
Cl -2.242248 3.473972 -0.000000

C8g
Na 1.430642 -1.681505 -1.039513
Cl 4.124979 -1.592101 -0.926982
Na 3.746993 -0.773115 1.552699
Cl 1.230701 -1.656551 1.763630
Na -1.312122 -2.425035 1.622587
Cl -2.345672 -0.274365 2.670160
Na 0.127989 0.836914 2.084446
Cl 2.866051 1.796321 0.963213
Na 3.715594 0.880900 -1.538467
Cl 1.104871 0.907490 -2.298832
Na 1.097221 3.055663 -0.619146
Cl -1.242040 2.573350 0.429035
Na -3.648640 1.418463 1.111853
Cl -1.357389 -2.211485 -1.116472
Na -1.516619 0.638153 -1.521738

Na -3.908699 -2.011570 -1.849336
Cl -4.208321 0.496897 -1.356530

C8h
Na 0.387654 -1.146500 2.706207
Cl 3.136281 -0.663875 2.642785
Na 2.508546 1.640283 3.633767
Cl 0.027220 1.773046 2.734653
Na -2.660655 0.978989 2.672158
Cl -2.932783 1.242602 0.000000
Na -0.241141 1.419934 0.000000
Cl 3.136281 -0.663875 -2.642785
Na 3.157148 -1.243327 -0.000000
Cl 0.443923 -1.245680 -0.000000
Na 0.387654 -1.146500 -2.706207
Cl -2.240434 -1.601940 -2.731453
Na -2.660655 0.978989 -2.672158
Cl -2.240434 -1.601940 2.731453
Na -2.393795 -1.594290 0.000000
Na 2.508546 1.640283 -3.633767
Cl 0.027220 1.773046 -2.734653

C8i
Na -1.961794 -0.187191 1.089188
Cl -4.447098 -0.847549 0.257787
Na -2.885296 -2.534643 -0.985992
Cl -0.839308 -2.934628 0.753354
Na 0.778700 -1.411652 -0.779185
Cl 3.281301 -1.957146 0.627253
Na 2.884362 0.927027 0.595830
Cl 0.585741 0.294489 2.037608
Na 0.017409 3.076130 1.932830
Cl 1.947925 3.369159 0.213798
Na 0.205461 2.066112 -1.410056
Cl -2.028820 2.566051 0.281270
Na -3.745779 1.175141 -1.254649
Cl -1.433694 -0.304482 -1.952175
Na 1.217643 -2.344915 2.388456
Cl 2.420022 0.297071 -2.095609

Na 4.283733 -1.510046 -1.791256

C8j
Na -1.118516 1.557333 -1.784801
Cl 0.000000 3.999258 -0.868192
Na 1.888177 3.714144 -2.648108
Cl 1.630684 1.102010 -2.386874
Na 1.118516 -1.557333 -1.784801
Cl 1.614588 -1.091755 0.835647
Na 1.403856 1.598229 0.410300
Cl 1.146086 2.442405 2.931326
Na -0.877824 3.588465 1.713022
Cl -1.614588 1.091755 0.835647
Na 0.000000 0.000000 3.036915
Cl -1.146086 -2.442405 2.931326
Na 0.877824 -3.588465 1.713022
Cl -1.630684 -1.102010 -2.386874
Na -1.403856 -1.598229 0.410300
Na -1.888177 -3.714144 -2.648108
Cl -0.000000 -3.999258 -0.868192

C8k
Na -1.235522 0.924632 0.157662
Cl -2.573709 3.363837 -0.143470
Na -0.265401 4.472685 0.365800
Cl 1.170253 2.350628 0.875218
Na 2.493898 0.744846 -1.057128
Cl 0.136352 -0.486804 -1.739276
Na 0.594324 -2.380615 0.256873
Cl -1.546492 -3.878323 -0.379816
Na -2.873423 -2.350251 1.296328
Cl -3.734948 -0.502123 -0.585434
Na -4.661392 1.947949 -0.746332
Cl 3.018950 -1.334646 1.040770
Na 1.550743 0.296268 2.584459
Cl -0.826426 -0.876229 2.229777
Na -2.026282 -2.007267 -2.172802
Cl 4.991968 0.923941 -1.682940
Na 5.440227 -0.968681 -0.089593

C8m
Na -0.355350 1.668387 1.864644
Cl -0.017945 -1.130454 2.102596
Na -0.017945 -3.744162 1.868836
Cl -1.799837 -4.078141 0.000000
Na -0.017945 -3.744162 -1.868836
Cl -0.017945 -1.130454 -2.102596
Na 1.653052 -0.848209 0.000000
Cl 1.773190 1.793824 0.000000
Na -0.355350 1.668387 -1.864644
Cl -2.385285 1.306430 0.000000
Na -1.955859 -1.332136 0.000000
Cl -0.649471 4.311960 2.024239
Na -2.369429 4.042640 0.000000
Cl -0.649471 4.311960 -2.024239
Na 1.068084 4.448256 0.000000
Cl 2.267914 -3.753098 0.000000
Na 4.636237 -4.681225 0.000000

C8o
Na 0.559600 0.750533 0.000000
Cl 0.205441 3.691115 0.000000
Na -1.821549 3.121627 1.791488
Cl -1.217545 0.498670 2.058568
Na -1.217545 -2.149745 1.805067
Cl -3.097366 -2.440722 0.000000
Na -1.217545 -2.149745 -1.805067
Cl 0.905309 -2.475205 0.000000
Na 3.880881 -1.501361 0.000000
Cl 3.238380 1.062248 0.000000
Na 2.844542 3.617371 0.000000
Cl -1.217545 0.498670 -2.058568
Na -1.821549 3.121627 -1.791488
Cl -3.732141 2.899446 0.000000
Na -3.249041 0.262685 0.000000
Cl 4.706319 -3.916464 0.000000
Na 2.365433 -4.791346 0.000000

6.3 Structures Anionic Clusters: B3LYP-D3/aug-cc-pVDZ

A1a
Cl 0.000000 -0.000000 2.522373
Na 0.000000 0.000000 0.000000
XX 0.000000 0.000000 -2.522373

A2a
Na -0.000000 2.332996 0.188463
Cl 0.000000 -0.000000 1.258113
Cl 0.000000 -4.629091 -0.751003
Na -0.000000 -2.332996 0.188463
Cl 0.000000 4.629091 -0.751003

A2b
Na 0.000000 0.000000 1.353892
Cl -0.000000 2.319104 0.000000
Na 0.000000 -0.000000 -1.353892
Cl -2.008403 -1.159552 0.000000
Cl 2.008403 -1.159552 0.000000

A2c
Cl -0.000000 2.083864 -1.153723
Na 0.000000 0.000000 -2.521982
Cl -0.000000 -2.083864 -1.153723
Na -0.000000 -0.000000 0.855523
Cl -0.000000 -0.000000 3.385742

A3a
Cl -2.423353 1.399123 -0.774518
Na 0.000000 1.918691 0.131225
Na -1.661635 -0.959345 0.131225
Cl 2.423353 1.399123 -0.774518
Cl -0.000000 -2.798247 -0.774518
Na 1.661635 -0.959345 0.131225
Cl 0.000000 0.000000 2.068823

A3b
Cl 0.000000 1.522469 -0.000000
Na -2.367404 2.353699 0.000000
Cl -3.943013 0.381122 0.000000
Na -1.518598 -0.822323 0.000000
Cl 0.369623 -2.531921 -0.000000
Na 2.199485 -0.552061 -0.000000
Cl 4.664667 -0.005344 -0.000000

A3c
Cl 2.667563 0.627022 0.000000
Na 2.721246 3.133037 0.000000
Cl 0.366739 4.002389 0.000000
Na 0.000000 1.266614 0.000000
Cl -2.048667 -0.305861 0.000000
Na -1.647473 -2.840351 0.000000
Cl -1.680430 -5.332509 0.000000

A3d
Na 2.060095 4.077514 0.000000
Cl 0.000000 2.526068 0.000000
Na 0.000000 0.000000 0.000000
Cl -0.000000 -2.526068 -0.000000
Na -2.060095 -4.077514 -0.000000
Cl -4.063577 -5.522769 -0.000000
Cl 4.063577 5.522769 0.000000

A4a
Cl 2.565175 2.565175 -0.393550
Na 0.000000 2.493197 0.104962
Cl -2.565175 2.565175 -0.393550
Na -2.493197 0.000000 0.104962
Cl -2.565175 -2.565175 -0.393550
Na -0.000000 -2.493197 0.104962
Cl 2.565175 -2.565175 -0.393550
Na 2.493197 -0.000000 0.104962
Cl 0.000000 0.000000 1.302535

A4b
Na -0.528122 1.760764 -1.488818
Cl 0.000000 3.742396 0.077063
Na 1.135526 1.605795 1.161686
Cl -0.000000 -0.000000 2.858909
Na -1.135526 -1.605795 1.161686
Cl -0.000000 -3.742396 0.077063
Cl -1.892527 -0.477559 -1.294843
Cl 1.892527 0.477559 -1.294843
Na 0.528122 -1.760764 -1.488818

A4c
Cl 2.183104 -3.086782 -0.000000
Na 0.604929 -1.693377 1.553992

Na 0.604929 -1.693377 -1.553992
Cl -1.675022 -1.852959 0.000000
Cl 0.604929 0.836479 -2.130185
Na 0.683425 2.417663 -0.000000
Na -1.799571 0.780502 0.000000
Cl 0.604929 0.836479 2.130185
Cl -1.778577 3.388811 0.000000

A4d
Cl 0.000000 2.440551 0.010034
Na 1.693227 0.977585 -1.341085
Cl 2.113579 -1.220276 0.010034
Na -1.693227 0.977585 -1.341085
Cl -2.113579 -1.220276 0.010034
Na -0.000000 -1.955170 -1.341085
Cl 0.000000 0.000000 -3.242874
Na 0.000000 0.000000 2.002441
Cl 0.000000 0.000000 4.520359

A4e
Na 0.355996 -2.893597 0.000000
Cl 1.380534 -0.265639 0.000000
Na -0.794480 -0.235086 1.746076
Cl -1.743059 1.571727 -0.000000
Na -0.794480 -0.235086 -1.746076
Cl -0.794480 -2.740118 -2.347388
Cl -0.794480 -2.740118 2.347388
Na 0.910105 2.548299 -0.000000
Cl 2.160394 4.701805 -0.000000

A4f
Cl -2.238011 -1.296989 0.000000
Na -3.447078 -3.508561 0.000000
Cl -1.746467 -5.371527 -0.000000
Na -0.129715 -3.242884 -0.000000
Cl 1.786839 -1.555498 -0.000000
Na -0.000000 0.386429 0.000000
Cl -0.165906 2.960022 0.000000
Na 1.807071 4.594333 0.000000
Cl 3.508659 6.409728 -0.000000

A4g
Cl 1.333006 -2.659342 -0.000000
Na 3.851230 -2.790104 -0.000000
Cl 4.882997 -0.491372 -0.000000
Na 2.345098 0.249875 -0.000000
Cl 0.000000 1.238851 0.000000
Na -0.981008 -1.241329 0.000000
Cl -3.495491 -0.868829 0.000000
Na -3.045680 1.791016 0.000000
Cl -4.124397 4.068691 0.000000

A4h
Cl -0.701100 -3.701295 2.053476
Na 0.367671 -2.153411 0.000000
Cl 2.052902 -0.191177 0.000000
Na 0.908423 2.115694 0.000000
Cl 1.793087 4.753042 0.000000
Na -1.597683 -4.815974 0.000000
Cl -0.701100 -3.701295 -2.053476
Na -0.701100 5.001765 0.000000
Cl -1.782049 2.744912 0.000000

A4i
Na -0.000000 6.808773 -0.225150
Cl -0.000000 8.991661 0.921948
Cl -0.000000 4.458543 -1.295781
Na -0.000000 2.284112 -0.029113
Cl -0.000000 -0.000000 1.076713
Na -0.000000 -2.284112 -0.029113
Cl -0.000000 -4.458543 -1.295781
Na -0.000000 -6.808773 -0.225150
Cl -0.000000 -8.991661 0.921948

A5a
Na 1.637677 -0.000000 -2.285466
Cl 0.000000 2.078678 -2.647282
Cl -0.000000 -2.078678 -2.647282
Na -1.637677 0.000000 -2.285466
Cl -1.977291 0.000000 0.272799
Na -0.000000 -2.038039 0.234712
Cl 1.977291 -0.000000 0.272799
Na 0.000000 2.038039 0.234712
Cl 0.000000 2.621781 2.799360
Na 0.000000 0.000000 2.788248
Cl -0.000000 -2.621781 2.799360

A5b
Cl 2.587507 -0.000000 0.251519
Na 1.631124 2.358406 -0.376733
Cl 0.000000 3.338870 -2.163211
Na -1.631124 2.358406 -0.376733
Na 1.631124 -2.358406 -0.376733
Cl -0.000000 -3.338870 -2.163211
Na -1.631124 -2.358406 -0.376733
Cl -2.587507 0.000000 0.251519
Cl 0.000000 -2.654331 1.794258
Na 0.000000 0.000000 1.869907
Cl 0.000000 2.654331 1.794258

A5c
Cl 2.528754 3.480531 -0.000000
Na -0.000000 3.038048 0.000000
Cl -2.528754 3.480531 -0.000000
Na 2.889355 0.938808 0.000000
Cl 4.091610 -1.329445 -0.000000
Na 1.785720 -2.457832 0.000000
Cl -0.000000 -4.302173 -0.000000
Na -1.785720 -2.457832 0.000000
Cl -4.091610 -1.329445 -0.000000
Na -2.889355 0.938808 0.000000
Cl 0.000000 0.000000 0.000000

A5d
Na -1.444100 -0.901433 2.204763
Cl 0.717983 -2.034557 3.008864
Na 1.744362 0.202503 2.045258
Cl 3.163801 0.933498 -0.000000
Cl -2.897618 -0.517872 0.000000
Na -1.444100 -0.901433 -2.204763
Cl -0.578283 1.679047 -2.436084
Na -1.443696 1.854379 0.000000
Cl -0.578283 1.679047 2.436084
Na 1.744362 0.202503 -2.045258
Cl 0.717983 -2.034557 -3.008864

A5e
Cl 1.212052 0.880849 -2.379082
Na 2.805712 1.162923 -0.330655
Cl 1.231631 2.229703 1.411263
Cl 2.895389 -1.358838 0.505135
Na 1.060907 -1.599926 -1.524748
Cl -0.871126 -2.800791 -0.328737
Na -2.750147 -0.982600 0.032829
Cl -3.155586 1.157067 -1.404911
Cl -1.843734 0.030583 2.292524
Na -0.926367 1.851020 -0.210169
Na 0.631112 -0.645576 1.884080

A5f
Na 0.000000 2.104387 -0.000000
Cl -1.779249 1.529019 1.897945
Na 0.000000 0.000000 3.032903
Cl 2.213794 0.776365 1.897945
Na 1.822453 -1.052193 -0.000000
Cl -0.434545 -2.305384 1.897945
Na -1.822453 -1.052193 -0.000000
Cl -2.213794 0.776365 -1.897945
Na 0.000000 0.000000 -3.032903
Cl 0.434545 -2.305384 -1.897945
Cl 1.779249 1.529019 -1.897945

A5g
Na 0.130864 0.555720 1.884283
Cl 0.195235 -2.229662 1.634003
Na 1.938449 -1.885934 -0.302991
Cl 4.144120 -0.494801 -0.378709
Na 2.382480 1.361869 -0.941326
Cl 0.844676 2.796718 0.589045
Cl -2.444190 0.733086 1.693674
Na -1.775523 1.360974 -0.888953
Cl 0.356929 -0.230970 -1.655767
Na -2.093384 -1.506130 0.173221
Cl -3.473932 -0.500929 -1.833220

A5h
Na -0.541678 -0.560397 2.196113
Cl 0.080763 1.999150 2.023633
Na 2.046676 2.298810 -0.000000
Cl 4.494359 2.773416 -0.000000
Cl -0.541678 -3.128855 2.466841
Na -1.338435 -3.211021 0.000000
Cl -0.541678 -3.128855 -2.466841
Na -0.541678 -0.560397 -2.196113
Cl 0.080763 1.999150 -2.023633
Cl -2.313421 -0.511676 0.000000
Na -1.570779 2.029402 0.000000

A5i
Cl -1.038689 1.044980 3.632220
Na 0.007993 -0.459467 1.822960
Na -1.038689 2.897122 1.787296
Cl -1.980698 4.531019 0.000000
Na -1.038689 2.897122 -1.787296
Cl -1.038689 1.044980 -3.632220
Na 0.007993 -0.459467 -1.822960
Cl 0.604168 1.450832 -0.000000
Cl 0.016100 -2.438420 0.000000
Na 1.832619 -4.289482 -0.000000
Cl 3.585840 -6.012456 -0.000000

A5j
Cl 2.794123 3.217235 -0.000000
Na 1.663535 1.730996 1.790753
Cl 1.663535 -0.255784 3.483095
Na 0.202365 -1.547014 1.830086
Cl -1.117523 -2.949225 0.000000
Na 0.202365 -1.547014 -1.830086
Cl 1.663535 -0.255784 -3.483095
Na 1.663535 1.730996 -1.790753
Cl -0.258055 0.577204 -0.000000
Na -2.892207 -0.717295 0.000000
Cl -5.288881 -0.107609 0.000000

A5k
Na 0.000000 2.055095 -0.030436
Cl 0.000000 0.000000 1.913931
Na -1.779765 -1.027548 -0.030436
Cl -0.000000 -2.446951 -1.266264
Na 1.779765 -1.027548 -0.030436
Cl 2.119122 1.223476 -1.266264
Cl -2.119122 1.223476 -1.266264
Na 0.000000 0.000000 -3.298247
Cl 0.000000 0.000000 -5.795513
Na 0.000000 0.000000 4.505604

Cl 0.000000 0.000000 6.958224

A6a
Cl 0.829308 1.393451 3.623401
Na 0.794962 -0.687007 2.040333
Cl -1.008696 -2.894600 2.033693
Na 0.506860 -3.660653 -0.000000
Cl -1.008696 -2.894600 -2.033693
Na 0.794962 -0.687007 -2.040333
Cl 0.829308 1.393451 -3.623401
Na -0.289126 2.812034 -1.778754
Cl -0.555417 4.696056 0.000000
Na -0.289126 2.812034 1.778754
Cl -0.993237 0.742277 0.000000
Na -2.137235 -1.618894 -0.000000
Cl 2.307767 -1.769892 -0.000000

A6b
Cl 2.374352 1.956790 0.377676
Na 0.000000 2.844098 -0.309245
Cl -2.374352 1.956790 0.377676
Cl 0.000000 1.866358 -2.738501
Na 1.775181 -0.000000 -1.704660
Na -1.775181 0.000000 -1.704660
Na 1.914250 -0.000000 2.100653
Cl -0.000000 -1.866358 -2.738501
Na -0.000000 -2.844098 -0.309245
Cl -2.374352 -1.956790 0.377676
Cl 2.374352 -1.956790 0.377676
Na -1.914250 0.000000 2.100653
Cl -0.000000 -0.000000 3.854034

A6c
Na 2.051779 0.907469 0.000000
Cl 2.124153 -1.738985 -0.000000
Cl -1.544276 4.191389 0.000000
Cl 1.409036 1.441627 2.569629
Na -0.956087 2.309408 -1.695858
Cl -1.209747 0.221995 0.000000
Na 0.695651 -1.187788 -2.239726
Cl -1.161652 -2.956490 -2.592543
Na -1.322110 -2.601569 -0.000000
Cl -1.161652 -2.956490 2.592543
Na 0.695651 -1.187788 2.239726
Na -0.956087 2.309408 1.695858
Cl 1.409036 1.441627 -2.569629

A6d
Cl -0.178697 0.983723 2.554465
Na 0.023751 -1.606458 2.305270
Cl -1.454661 -1.841817 -0.000000
Na -1.503238 0.902123 0.000000
Cl -1.710464 3.575947 0.000000
Na 0.431822 3.393970 1.650252
Cl 2.240137 4.293061 0.000000
Na 0.431822 3.393970 -1.650252
Cl -0.178697 0.983723 -2.554465
Na 0.023751 -1.606458 -2.305270
Cl 0.800225 -4.075358 -2.495496
Cl 0.800225 -4.075358 2.495496
Na 0.100530 -4.235936 -0.000000

A6e
Cl 1.855279 0.233561 2.013680
Na 1.770401 -1.960866 0.183643
Cl 3.835467 -1.229112 -1.281589
Na 2.247267 0.865648 -1.287078
Cl 0.855989 3.174253 -1.089372
Na -1.869713 1.415553 -0.988591
Cl -0.038681 -0.551036 -1.384451
Na -0.681518 -0.595670 2.012866
Cl -0.710088 -3.124800 1.198668
Na -2.121546 -2.112218 -0.886818
Cl -3.871100 -0.309749 -1.513938
Cl -1.892388 1.745985 1.775975
Na 0.601827 2.481669 1.400291

A6f
Cl 0.000000 0.000000 3.148724
Na 1.806571 1.043024 1.405591
Cl 2.375030 1.371224 -1.859904
Na 0.000000 2.417462 -1.605329
Cl -2.375030 1.371224 -1.859904
Na -2.093583 -1.208731 -1.605329
Cl -0.000000 -2.742448 -1.859904
Na -0.000000 -2.086049 1.405591
Cl 0.000000 3.065648 0.939571
Na -1.806571 1.043024 1.405591
Cl -2.654929 -1.532824 0.939571
Na 2.093583 -1.208731 -1.605329
Cl 2.654929 -1.532824 0.939571

A6g
Cl 2.709936 1.564582 1.552476
Na 0.000000 2.374487 1.194234
Cl -2.709936 1.564582 1.552476
Na -2.056366 1.187244 -1.194234
Cl 0.000000 3.129165 -1.552476
Na 2.056366 1.187244 -1.194234
Cl 2.709936 -1.564582 -1.552476
Na 2.056366 -1.187244 1.194234
Cl -0.000000 -3.129165 1.552476
Na -0.000000 -2.374487 -1.194234
Cl 0.000000 0.000000 0.000000
Na -2.056366 -1.187244 1.194234
Cl -2.709936 -1.564582 -1.552476

A6h
Cl -0.056372 3.889866 2.259559
Na 1.543133 1.849464 2.024499
Cl 2.769570 2.366284 -0.293222
Na 0.000000 3.188833 -0.274193
Na -1.554135 -0.454291 -1.655947
Cl 0.000000 0.000000 0.897036
Na -1.543133 -1.849464 2.024499
Cl -2.769570 -2.366284 -0.293222
Cl 0.056372 -3.889866 2.259559
Na -0.000000 -3.188833 -0.274193
Cl 0.746889 -1.937410 -2.475911
Cl -0.746889 1.937410 -2.475911
Na 1.554135 0.454291 -1.655947

A6i
Na -0.492723 1.651369 -0.609355
Cl -1.926531 -0.277297 1.875860
Na 0.692845 -0.229099 1.901197
Cl 2.991917 -1.493104 1.555734
Na 2.622171 -1.562547 -1.139023
Cl 0.064875 -1.033047 -1.316487
Na -3.971075 0.668437 0.201008
Cl -2.854540 2.654750 -1.024919
Cl 1.557399 2.190603 1.045595
Na 3.772588 0.915250 0.441814
Cl 4.552789 0.022514 -1.865889
Cl -4.688836 -1.722828 -0.638715
Na -2.155645 -1.971323 -0.225644

A6j
Na -1.789448 -2.886540 -0.000000
Cl -1.966239 -0.309265 -0.000000
Cl -0.180585 -3.369166 2.053561
Na 1.502439 -3.523894 -0.000000
Cl 2.487254 -1.140682 -0.000000
Cl -0.180585 -3.369166 -2.053561
Na 0.494307 -0.483595 -1.761992
Cl 0.494307 2.083492 -2.145877
Na 1.032835 3.572469 0.000000
Cl 0.494307 2.083492 2.145877
Na 0.494307 -0.483595 1.761992
Cl -1.229130 4.958832 0.000000
Na -1.609765 2.356234 0.000000

A6k
Cl 2.692540 -0.539175 0.000000
Na 2.035457 2.061371 0.000000
Cl 0.559667 2.241149 2.133939
Na -1.661350 2.608978 0.000000
Cl -3.676287 4.119960 0.000000
Na 1.963073 -3.026257 0.000000
Cl 0.283436 -3.190244 2.054807
Na -1.410948 -2.944555 0.000000
Cl 0.283436 -3.190244 -2.054807
Na 0.283436 -0.367569 -1.943074
Cl 0.559667 2.241149 -2.133939
Na 0.283436 -0.367569 1.943074
Cl -1.668588 -0.365441 0.000000

A6l
Cl 1.385478 -2.947611 1.226039
Na -0.178647 -0.742727 1.749507
Cl -2.227226 0.947483 2.074081
Na -3.321253 -0.137207 -0.237017
Cl -5.316116 -1.341663 -1.142378
Na -0.408622 2.493995 0.879541
Cl -0.660920 -0.300409 -1.085310
Cl 0.574899 3.718283 -1.185189
Na 1.564770 1.335752 -1.476997
Cl 3.183905 -0.879174 -1.829027
Na 0.957106 -2.325909 -1.341211
Cl 1.891855 1.064113 1.685639
Na 3.191931 -1.027303 0.822037

A6m
Na -3.707717 -1.021216 -0.058853
Cl -2.276695 -0.894025 2.173657
Cl -3.628681 1.534821 -0.732901
Cl -1.935333 -2.032495 -1.612376
Na -1.034182 0.813130 -1.700791
Na -0.127011 -1.840538 0.422550
Na -2.112237 1.644888 1.481959
Cl 1.400142 -0.254525 -1.791817
Na 2.279432 1.267084 0.496549
Cl 2.245620 -1.826762 1.556252
Cl 0.023300 2.628739 0.453693
Cl 4.860710 1.046780 -0.082281
Na 3.636798 -1.176353 -0.586128

A6n
Na -2.211926 -2.545107 0.000000
Cl -0.552555 -2.931565 2.019266
Na 1.283508 -3.033779 0.000000
Cl 3.386821 -1.587618 0.000000
Na 2.561436 0.879981 0.000000
Cl 1.042914 1.792684 -1.984580
Na -0.552555 -0.228438 -2.100921
Cl -0.552555 -2.931565 -2.019266
Cl -2.240712 0.078648 0.000000
Na -0.552555 -0.228438 2.100921
Cl 1.042914 1.792684 1.984580
Cl -2.082737 5.120447 0.000000
Na -0.596047 3.094582 0.000000

A6o
Cl -0.176069 1.094262 2.454198
Na -0.176069 3.596460 1.624896
Cl 1.406720 4.866601 -0.000000

Na -0.176069 3.596460 -1.624896
Cl -0.176069 1.094262 -2.454198
Na 0.868568 -1.128723 -1.703179
Cl 2.431100 -2.442361 0.000000
Na 0.868568 -1.128723 1.703179
Cl -2.341421 3.345594 -0.000000
Na -1.783238 0.743233 -0.000000
Cl -1.169989 -1.938293 0.000000
Na 0.384057 -4.395054 0.000000
Cl 0.034905 -6.850667 0.000000

A6p
Cl 1.721840 -4.068644 0.000000
Na 1.371541 -1.491282 0.000000
Cl 1.578279 1.714624 0.000000
Na 2.926951 3.925177 0.000000
Cl 3.945318 6.157481 0.000000
Na -0.543502 1.383925 1.696533
Cl -0.543502 -1.172606 1.955728
Cl -2.517993 1.838444 0.000000
Na -2.635409 -1.023922 0.000000
Cl -3.446894 -3.505482 0.000000
Na -0.875196 -3.855147 0.000000
Cl -0.543502 -1.172606 -1.955728
Na -0.543502 1.383925 -1.696533

A6q
Cl 0.413709 4.608805 2.504050
Na 0.075344 2.189989 3.193563
Cl -0.297565 -0.144942 4.154617
Na -0.473767 -1.048996 1.774871
Cl -0.116435 1.088144 0.000000
Na 0.075344 2.189989 -3.193563
Cl -0.297565 -0.144942 -4.154617
Na -0.473767 -1.048996 -1.774871
Cl -0.869042 -2.954088 -0.000000
Na 0.106371 -5.373334 -0.000000
Cl 0.997391 -7.660780 -0.000000
Cl 0.413709 4.608805 -2.504050
Na 0.313068 4.017072 0.000000

A6r
Cl -0.144826 4.013911 -0.151992
Na -1.590325 1.931421 0.288228
Na 1.797979 2.285663 -0.261508
Cl 4.173459 1.331252 -0.722448
Na 2.497604 -1.716395 -0.139459
Cl 0.922572 -2.878339 -1.890390
Na -0.952176 -1.352765 -1.073572
Cl -3.167052 0.156831 -0.933316
Na -5.437392 -0.516330 0.126848
Cl -7.592819 -0.959277 1.222446
Cl 0.446447 0.144323 0.719603
Cl 4.398899 -2.263473 1.650641
Na 5.173077 0.071236 1.222440

A7a
Na -2.042085 0.000000 0.963601
Cl -2.101381 2.720224 0.963010
Na -0.000000 2.483175 2.504480
Cl 2.101381 2.720224 0.963010
Na 2.042085 -0.000000 0.963601
Cl 2.101381 -2.720224 0.963010
Na -0.000000 -2.483175 2.504480
Cl -2.101381 -2.720224 0.963010
Na 0.000000 -2.747057 -1.037245
Cl 0.000000 0.000000 -0.768909
Na 0.000000 2.747057 -1.037245
Cl 0.000000 -2.609499 -3.669225
Cl -0.000000 0.000000 3.375510
Cl 0.000000 2.609499 -3.669225
Na 0.000000 0.000000 -3.501967

A7b
Na 2.402048 -1.753713 -0.065906
Cl 0.447017 -3.495197 -0.870673
Cl 1.286991 -1.137427 2.587787
Na 0.439029 1.293431 1.794212
Cl 1.456135 3.541710 0.161981
Na -0.922514 2.522359 -0.887715
Cl -2.789919 0.982833 -2.241855
Na -3.406623 0.467830 0.294269
Cl -2.069836 2.254052 1.628115
Cl 0.549145 0.199638 -0.844279
Cl -3.057018 -2.098701 0.469601
Na -1.357087 -1.474193 -1.705145
Na -0.608415 -2.486624 1.361724
Na 2.905562 1.619287 -0.898104
Cl 4.532072 -0.368799 -0.821657

A7c
Na -0.000000 1.989604 1.628490
Cl -0.000000 2.604068 4.184264
Na -0.000000 0.000000 4.206563
Cl -0.000000 -2.604068 4.184264
Na -0.000000 -1.989604 1.628490
Cl 0.000000 -2.221755 -1.174103
Na 0.000000 -1.701855 -3.718388
Cl 2.040657 -0.000000 -3.885297
Na 0.000000 1.701855 -3.718388
Cl -2.040657 0.000000 -3.885297
Na -1.846869 0.000000 -1.057531
Cl -1.971824 0.000000 1.550760
Cl 0.000000 2.221755 -1.174103
Na 1.846869 -0.000000 -1.057531
Cl 1.971824 -0.000000 1.550760

A7d
Na -1.923174 -0.873275 1.895218
Cl -3.653645 0.003079 -0.000000
Na -1.923174 -0.873275 -1.895218
Cl 0.026214 -1.285090 0.000000
Cl -0.970257 -1.563618 4.203746
Na 0.888008 -0.004182 3.282396
Cl 3.174124 0.084921 2.009735
Na 1.435550 1.391821 0.000000
Cl -0.399849 2.059062 -2.046752
Na -2.014471 2.073563 -0.000000
Cl -0.399849 2.059062 2.046752
Na 2.679229 -1.523034 0.000000
Cl 3.174124 0.084921 -2.009735
Cl -0.970257 -1.563618 -4.203746
Na 0.888008 -0.004182 -3.282396

A7e
Na 0.329512 -1.596971 1.999030
Cl -1.988140 -0.319090 2.047347
Na -1.985338 -2.036681 -0.400046
Cl 2.033399 0.316189 2.418030
Na 3.119438 0.744146 -0.030406
Cl 4.084169 -1.268302 -1.415583
Na 1.724970 -2.260825 -1.323750
Cl 0.000847 -0.184329 -1.203489
Na -0.044991 2.408152 -1.795863
Cl 2.037923 3.133026 -0.429439
Cl 0.098717 -3.653241 0.293340
Cl -1.984667 3.146540 -0.156792
Na -0.002689 1.822848 1.372410
Na -3.263683 0.858464 -0.037764
Cl -4.202802 -1.131408 -1.413398

A7f
Na -1.560371 2.141649 -0.847372
Cl -4.000800 1.063931 -0.369400
Na -3.274715 -1.263387 -1.169293
Cl -2.487391 -2.753323 0.852262
Na -1.758735 -0.139621 1.663331
Cl -1.028926 2.426600 1.856082
Na 1.464965 2.077721 1.038935
Cl 3.874970 1.089278 0.329028
Na 1.866151 0.215534 -1.712520
Cl 2.556792 -2.544424 -1.186750
Na 3.293854 -1.323169 1.020314
Cl 0.888771 -0.942950 2.017554
Na 0.065941 -2.492992 -0.090416
Cl -0.801066 -0.589634 -1.844575
Cl 0.934827 2.757988 -1.591422

A7g
Na -2.591198 0.000000 0.251550
Cl -1.931030 1.925777 1.924251
Cl -1.931030 -1.925777 1.924251
Na 0.000000 -3.399515 0.886826
Cl 0.000000 -4.263897 -1.558072
Na 0.000000 -1.844594 -2.430714
Cl 1.954305 -0.000000 -2.310483
Na 2.591198 -0.000000 0.251550
Cl 1.931030 -1.925777 1.924251
Cl -1.954305 0.000000 -2.310483
Cl 1.931030 1.925777 1.924251
Na 0.000000 3.399515 0.886826
Na -0.000000 0.000000 2.646655
Na 0.000000 1.844594 -2.430714
Cl 0.000000 4.263897 -1.558072

A7h
Na 1.924557 -1.870978 0.892409
Cl 4.409784 -1.261763 1.287245
Na 3.792221 0.796172 -0.182467
Cl 2.894034 3.224361 -0.171093
Na 0.674306 1.957811 -0.759423
Cl 0.019348 0.076203 1.308193
Na -1.824218 1.824763 1.917170
Cl -3.858870 0.130194 1.704977
Na -2.038326 -1.781001 0.847472
Cl -2.743941 -1.372497 -1.906619
Na -0.164869 -1.844684 -1.860945
Cl 1.888271 -0.265143 -1.972460
Cl -0.075925 -3.552079 0.212352
Cl -1.881512 3.030051 -0.463581
Na -3.370053 0.903499 -0.852691

A7i
Na 0.000000 2.361998 -1.138976
Cl -2.124746 1.226723 -2.671570
Cl 2.124746 1.226723 -2.671570
Cl 0.000000 0.000000 0.106057
Na 0.000000 0.000000 -3.612345
Cl -0.000000 -2.453445 -2.671570
Na 2.045551 -1.180999 -1.138976
Cl 2.804730 -1.619312 1.440418
Na 1.659209 0.957945 2.123483
Cl 0.000000 0.000000 4.013695
Na -1.659209 0.957945 2.123483
Cl -2.804730 -1.619312 1.440418
Na -2.045551 -1.180999 -1.138976
Cl 0.000000 3.238624 1.440418
Na -0.000000 -1.915890 2.123483

A7j
Cl 2.141126 -2.994212 0.000000
Na -0.404808 -3.571517 0.000000
Cl -1.085952 -2.647731 2.352402
Na 1.720059 -0.896456 1.658245
Na 1.720059 -0.896456 -1.658245
Cl -1.085952 -2.647731 -2.352402

Na -2.052688 -0.292559 -1.933455
Na -2.052688 -0.292559 1.933455
Cl 0.084815 1.234456 2.045222
Cl 0.084815 1.234456 -2.045222
Na 1.772108 2.604130 0.000000
Cl 3.412313 0.551994 0.000000
Cl -3.430310 0.920313 0.000000
Na -1.322584 2.705746 0.000000
Cl 0.280671 4.762360 0.000000

A7k
Na 0.713100 3.980366 -0.000000
Cl 1.684653 1.583430 -0.000000
Cl -0.979430 3.818451 2.056883
Cl -0.979430 3.818451 -2.056883
Na -2.526589 3.158232 -0.000000
Cl -2.417592 0.563098 -0.000000
Na -0.192081 1.051413 1.898785
Cl -0.192081 -1.449836 2.661628
Na 1.568263 -3.119407 1.639797
Cl -0.229945 -4.348674 0.000000
Na -1.394002 -1.954973 0.000000
Cl -0.192081 -1.449836 -2.661628
Na -0.192081 1.051413 -1.898785
Cl 3.600399 -3.212966 0.000000
Na 1.568263 -3.119407 -1.639797

A7l
Na 2.795874 1.335592 0.486992
Cl 0.941467 -3.454984 -0.602540
Na -1.351177 -2.356254 0.425663
Cl -3.918133 -2.398448 0.290236
Na -3.788911 0.135494 -0.279688
Cl -3.607074 2.269351 1.174143
Na -1.062039 1.838085 1.346218
Cl 0.952756 3.367455 0.451260
Na 0.274777 2.281149 -1.844068
Cl -1.515964 0.304997 -1.820279
Na 0.760255 -1.116251 -1.842310
Cl 2.450744 0.838245 -2.242643
Cl 0.290388 -0.463229 1.495862
Cl 4.371817 -0.471052 1.570806
Na 2.423762 -2.105971 1.217524

A7m
Cl -2.608076 2.621466 0.000000
Na -1.823057 -0.045090 0.000000
Na -0.603676 3.147752 1.673064
Cl 1.152422 4.257639 0.000000
Na 2.103610 1.484495 0.000000
Cl -1.778306 -1.986687 1.978273
Na -1.565486 -3.669323 0.000000
Cl 0.995184 -4.077819 0.000000
Na 1.061071 -1.804109 -1.698082
Cl 3.104035 -0.963895 0.000000
Na 1.061071 -1.804109 1.698082
Cl 0.576275 0.920020 2.202193
Cl -1.778306 -1.986687 -1.978273
Na -0.603676 3.147752 -1.673064
Cl 0.576275 0.920020 -2.202193

A7n
Na 0.425441 -3.612485 1.632609
Cl 1.531632 -1.621891 0.000000
Cl -1.374074 -1.875652 2.440799
Na -1.952882 -0.997197 -0.000000
Cl -0.947518 1.508405 -0.000000
Cl -1.374074 -1.875652 -2.440799
Na 0.532169 0.125061 2.039460
Cl 1.112791 2.186916 3.499450
Na -0.113763 3.658138 1.774704
Cl -0.387234 5.522755 -0.000000
Na -0.113763 3.658138 -1.774704
Cl 1.112791 2.186916 -3.499450
Na 0.532169 0.125061 -2.039460
Na 0.425441 -3.612485 -1.632609
Cl 0.497278 -5.607474 0.000000

A7o
Na -1.566344 2.494507 1.616319
Cl -3.583567 1.643933 0.000000
Na -2.232367 -0.590673 -0.000000
Cl -0.437533 -2.764213 -0.000000
Cl -1.566344 0.167914 3.000502
Na 0.559115 -0.945215 2.085729
Cl 3.118230 -1.923003 -2.026697
Na 0.559115 -0.945215 -2.085729
Cl -1.566344 0.167914 -3.000502
Na -1.566344 2.494507 -1.616319
Cl -0.756000 4.427761 0.000000
Na 2.135216 -3.332963 -0.000000
Cl 3.118230 -1.923003 2.026697
Cl 0.899419 0.847227 0.000000
Na 3.307651 -0.171037 0.000000

A7p
Na -1.566344 2.494507 1.616319
Cl -3.583567 1.643933 -0.000000
Na -2.232367 -0.590673 -0.000000
Cl -0.437533 -2.764213 0.000000
Cl -1.566344 0.167914 3.000502
Na 0.559115 -0.945215 2.085729
Cl 3.118230 -1.923003 -2.026697
Na 0.559115 -0.945215 -2.085729
Cl -1.566344 0.167914 -3.000502
Na -1.566344 2.494507 -1.616319
Cl -0.756000 4.427761 -0.000000
Na 2.135216 -3.332963 0.000000
Cl 3.118230 -1.923003 2.026697
Cl 0.899419 0.847227 -0.000000
Na 3.307651 -0.171037 0.000000

A7q
Na 3.941909 -0.905760 -0.888861
Cl 2.378206 0.795320 -2.022837
Cl 4.049446 0.115591 1.576351
Na 2.068923 -1.641098 1.743101
Cl -0.018437 -0.136169 1.365452
Cl 2.257458 -2.937789 -0.551060
Na 2.071151 1.961251 0.434805
Cl 0.314015 3.877240 0.443235
Na -1.571410 2.214701 -0.277315
Cl -3.881061 1.274450 0.863876
Na -3.857877 -0.657813 -1.033425
Cl -1.829020 0.309824 -2.359854
Na 0.329650 -0.956087 -1.447014
Na -2.516754 -0.818666 1.805213
Cl -3.571872 -2.778573 0.467099

A7r
Na -3.609054 -1.302481 0.611824
Cl -1.870171 -3.240247 0.350848
Cl -1.808418 0.289343 1.672212
Na 0.375272 1.945119 1.270322
Cl -1.071027 4.039950 0.603482
Na -2.224502 2.065236 -0.686248
Cl 0.098767 1.111431 -1.864120
Na 2.476230 0.112695 -1.999412
Cl 1.739145 -2.125453 -0.638782
Cl -3.688677 -0.057233 -1.691864
Cl 1.951149 0.093613 2.592633
Na 0.095388 -1.681285 1.562279
Na -1.110904 -1.261917 -1.445641
Na 3.640993 -0.766507 0.807083
Cl 4.879959 0.463921 -1.102191

A7s
Na -0.360707 -0.528417 -1.554907
Cl 0.094686 -2.980180 -0.432863
Na 2.402779 -2.126181 0.560256
Cl 2.795100 0.114121 2.049896
Na 0.734397 1.732275 1.686206
Cl -0.269956 4.112723 1.152303
Na -0.899642 2.788147 -0.979201
Cl -2.535521 1.081587 -2.296277
Na -3.757913 0.107191 -0.154721
Cl -1.416484 0.203661 1.200412
Na -1.929772 -2.424142 1.160044
Cl -4.488163 -2.235924 0.705026
Cl 1.427035 1.447894 -1.511714
Cl 4.471025 -1.822837 -1.022640
Na 3.690743 0.573151 -0.476805

A7t
Na 3.139424 -0.127709 -0.967866
Cl 2.008177 -2.414581 0.245392
Na -0.248963 -0.948639 -1.865825
Cl 0.496494 0.634396 1.176156
Na -2.067633 1.607157 1.338157
Cl -1.417345 4.042877 0.599537
Na -0.084268 2.437788 -1.014625
Cl -2.382442 0.769399 -1.492774
Na -3.520114 -1.305236 -0.370334
Cl -2.974152 -0.872207 2.174939
Cl 1.479158 0.896708 -2.721205
Cl -1.713567 -3.065727 -1.050036
Na -0.567275 -1.961021 1.170633
Cl 4.875188 0.504888 0.887620
Na 2.774675 -0.468501 1.988617

A7u
Na 1.042348 2.708479 -1.420061
Cl 2.701386 1.008777 -2.405683
Cl 1.799055 2.916029 1.059009
Na -0.148040 1.159217 1.855894
Cl -2.728193 0.637284 2.101329
Na -2.733331 -1.370201 0.046111
Cl -0.696440 -2.914024 -0.532294
Na 1.269092 -2.706929 1.206014
Cl 1.657605 -0.599224 2.677486
Cl -1.028027 1.104249 -1.250829
Cl 3.177789 -2.159574 -0.492889
Na 0.988554 -1.077766 -1.836131
Na 3.121782 0.428016 0.432807
Cl -4.909981 -0.429231 -1.158797
Na -3.498976 1.532560 -0.280510

A7v
Na -1.042347 -2.708479 -1.420062
Cl -2.701386 -1.008776 -2.405683
Cl -1.799055 -2.916029 1.059009
Na 0.148040 -1.159217 1.855894
Cl 2.728193 -0.637284 2.101329
Na 2.733332 1.370201 0.046111
Cl 0.696440 2.914024 -0.532293
Na -1.269092 2.706929 1.206015
Cl -1.657606 0.599224 2.677486
Cl 1.028026 -1.104249 -1.250829
Cl -3.177789 2.159574 -0.492889
Na -0.988554 1.077766 -1.836131
Na -3.121782 -0.428016 0.432806
Cl 4.909982 0.429231 -1.158797
Na 3.498976 -1.532560 -0.280510

A7w
Na -2.611131 -1.880405 0.000000

Cl -2.481453 -0.895671 2.502903
Na -2.481453 1.606128 -1.692533
Cl -0.410240 1.155360 0.000000
Na 2.230646 1.578270 0.000000
Cl 4.795991 2.141963 0.000000
Na 4.309996 -0.447709 0.000000
Cl 2.654014 -0.491729 2.095959
Na 0.226618 -1.355956 1.851780
Cl -2.481453 -0.895671 -2.502903
Cl -0.349076 -3.212513 0.000000
Na 0.226618 -1.355956 -1.851780
Cl 2.654014 -0.491729 -2.095959
Na -2.481453 1.606128 1.692533
Cl -4.006401 2.851432 0.000000

A7x
Na -2.611131 -1.880405 0.000000
Cl -2.481453 -0.895671 2.502903
Na -2.481453 1.606128 -1.692533
Cl -0.410240 1.155360 0.000000
Na 2.230646 1.578270 0.000000
Cl 4.795991 2.141963 0.000000
Na 4.309996 -0.447709 0.000000
Cl 2.654014 -0.491729 2.095959
Na 0.226618 -1.355956 1.851780
Cl -2.481453 -0.895671 -2.502903
Cl -0.349076 -3.212513 0.000000
Na 0.226618 -1.355956 -1.851780
Cl 2.654014 -0.491729 -2.095959
Na -2.481453 1.606128 1.692533
Cl -4.006401 2.851432 0.000000

A7y
Na 2.687572 -1.582817 0.493941
Cl 2.377093 -1.394622 -2.157718
Na -0.112711 -2.324456 -1.356679
Cl -2.577629 -2.204323 -2.073035
Na -3.300096 -0.284518 -0.492320
Cl -2.574371 0.108552 2.460556
Na -0.138125 -0.872457 2.263232
Cl 2.572261 0.118859 2.571675
Na 2.191227 2.101307 0.918154
Cl -0.022072 0.837298 -0.138337
Na 1.639918 1.149953 -2.252980
Cl 3.001612 3.156212 -1.282558
Cl 0.664962 -3.142632 1.092427
Cl -3.988506 2.261504 -0.798203
Na -2.122962 2.213497 0.929226

A7z
Na -0.000000 2.875449 -0.479639
Cl 0.000000 1.774497 1.955620
Cl -2.332314 1.948180 -1.217011
Na -1.906245 0.000000 -2.987711
Cl -0.000000 -0.000000 -4.726877
Na 1.906245 -0.000000 -2.987711
Cl 2.332314 -1.948180 -1.217011
Na -0.000000 -2.875449 -0.479639
Cl -2.332314 -1.948180 -1.217011
Cl 2.332314 1.948180 -1.217011
Cl -0.000000 -1.774497 1.955620
Na 1.887834 -0.000000 0.694131
Na -1.887834 0.000000 0.694131
Na 0.000000 0.000000 4.119493
Cl 0.000000 0.000000 6.606998

A7za
Cl 2.355738 -0.074994 -0.000000
Na 0.438528 0.540596 1.933241
Cl 0.438528 -2.098936 2.604809
Na 0.951397 -2.544115 0.000000
Cl 0.438528 -2.098936 -2.604809
Na -2.143681 -1.955477 -2.091728
Cl -2.364001 -3.457100 0.000000
Na -2.143681 -1.955477 2.091728
Cl -2.378163 0.661470 2.316331
Na -2.340939 1.863950 0.000000
Cl 0.165210 2.721203 -0.000000
Na 0.438528 0.540596 -1.933241
Cl -2.378163 0.661470 -2.316331
Na 2.998808 2.632824 -0.000000
Cl 4.887701 4.253359 -0.000000

A7zb
Na -2.059473 -1.420456 0.000000
Cl -1.622996 -0.252189 2.430696
Na 1.042073 -0.258938 2.068009
Cl 3.418382 0.690937 2.569527
Na 3.296614 1.132963 0.000000
Cl 3.418382 0.690937 -2.569527
Na 1.042073 -0.258938 -2.068009
Cl -1.622996 -0.252189 -2.430696
Na -1.622996 2.256766 -1.629270
Cl 0.546887 1.922723 0.000000
Na -1.622996 2.256766 1.629270
Cl -3.185015 3.521716 0.000000
Cl 1.408453 -1.988737 0.000000
Cl -2.444011 -4.068465 0.000000
Na 0.052848 -4.117295 0.000000

A8a
Cl -2.600223 2.600223 1.516962
Na -0.000000 2.908772 1.214048
Cl -0.000000 3.677270 -1.516962
Na -2.056813 2.056813 -1.214048
Cl -2.600223 -2.600223 1.516962
Na -2.056813 -2.056813 -1.214048
Cl 0.000000 0.000000 0.000000
Na 2.056813 2.056813 -1.214048
Cl 2.600223 2.600223 1.516962
Na 2.908772 -0.000000 1.214048
Cl 3.677270 -0.000000 -1.516962
Na 2.056813 -2.056813 -1.214048
Cl -0.000000 -3.677270 -1.516962
Na -0.000000 -2.908772 1.214048
Cl 2.600223 -2.600223 1.516962
Cl -3.677270 0.000000 -1.516962
Na -2.908772 0.000000 1.214048

A8b
Cl 0.000000 0.000000 0.000000
Na 0.000000 2.596392 0.827393
Cl -0.617070 1.953373 3.382182
Na 1.606866 0.471686 2.900067
Cl -2.954334 -1.858778 1.106224
Na -0.000000 -2.596392 0.827393
Cl 1.858778 -2.954334 -1.106224
Na 2.596392 -0.000000 -0.827393
Cl 1.953373 0.617070 -3.382182
Na 0.471686 -1.606866 -2.900067
Cl -1.953373 -0.617070 -3.382182
Na -0.471686 1.606866 -2.900067
Cl -1.858778 2.954334 -1.106224
Na -2.596392 0.000000 -0.827393
Cl 2.954334 1.858778 1.106224
Cl 0.617070 -1.953373 3.382182
Na -1.606866 -0.471686 2.900067

A8c
Cl -0.102240 0.096509 -0.447850
Na 2.640494 1.563596 -0.941511
Cl 3.601594 -1.046497 -1.634595
Na 1.145103 -1.982168 -1.582553
Cl -1.185536 -3.186757 -1.901324
Na -2.572880 -0.829671 -1.490507
Cl -3.488190 -1.616480 1.090026
Na -3.170166 0.986139 1.222516
Cl -1.292114 2.436320 2.281294
Na -0.927086 2.770786 -0.733887
Cl 1.301377 3.606019 -1.824066
Na -1.146891 -2.757503 0.795734
Cl 1.292063 -2.579736 1.786464
Na 0.655216 0.628321 2.108278
Cl -3.422894 1.727119 -1.337350
Cl 3.243854 1.166897 1.746762
Na 3.456707 -1.312018 0.993827

A8d
Cl -0.273622 1.709307 2.763064
Na -2.645747 0.328413 2.114169
Cl -2.460474 -1.779850 3.616285
Na -0.048583 -1.551818 2.714122
Cl 2.666077 -1.414683 2.675617
Na 2.489198 -1.116051 0.000000
Cl -0.272168 -0.996829 0.000000
Na -2.645747 0.328413 -2.114169
Cl -3.234005 1.779422 -0.000000
Na -0.414775 1.843193 -0.000000
Cl -0.273622 1.709307 -2.763064
Na 2.251365 1.193637 -2.506151
Cl 2.666077 -1.414683 -2.675617
Na 2.251365 1.193637 2.506151
Cl 2.873186 1.755879 -0.000000
Cl -2.460474 -1.779850 -3.616285
Na -0.048583 -1.551818 -2.714122

A8e
Cl -0.322860 0.476092 0.000000
Na -1.573340 2.835670 0.000000
Cl 0.805112 4.011921 0.000000
Na 1.993522 2.132346 1.670580
Cl 1.993522 -0.128225 -3.037183
Na -0.149983 -1.275407 -2.117410
Cl 0.968244 -3.051356 0.000000
Na -1.634595 -3.590003 0.000000
Cl -2.638592 -2.254925 2.024855
Na -3.291256 -0.440580 0.000000
Cl -4.035892 2.079184 0.000000
Na 2.683532 -0.874451 0.000000
Cl 1.993522 -0.128225 3.037183
Na -0.149983 -1.275407 2.117410
Cl -2.638592 -2.254925 -2.024855
Cl 3.958737 1.480479 0.000000
Na 1.993522 2.132346 -1.670580

A8f
Cl -0.099548 0.284947 0.342462
Na -0.585722 2.805451 1.170840
Cl -3.185073 2.184782 1.252402
Na -2.736400 -0.464887 1.660878
Cl -1.363134 -2.738506 2.258238
Na 1.023319 -1.786147 1.661897
Cl 1.025994 -2.804026 -1.456905
Na 3.333405 -1.636642 -0.973581
Cl 3.287380 0.785199 -1.942110
Na 2.892650 1.460214 0.697054
Cl 1.802981 3.574829 1.774245
Na -1.437637 -2.630653 -0.499380
Cl -3.743128 -1.380052 -0.696089
Na 0.646968 0.386549 -2.280725
Cl 3.567969 -1.108822 1.610674
Na -2.988204 1.108584 -1.295309
Cl -1.389452 1.691817 -3.234587

A8g
Cl 0.065893 -0.092622 0.000000

Na 0.825312 -2.857245 1.609109
Cl 2.619484 -3.787075 0.000000
Na 0.825312 -2.857245 -1.609109
Cl -1.519764 -3.294875 -0.000000
Na -1.972959 -1.063877 -1.652677
Cl -0.301758 3.327057 -2.057258
Na 1.288241 1.064743 -2.060313
Cl 3.070600 2.081567 0.000000
Na 1.241421 3.927645 -0.000000
Cl -0.301758 3.327057 2.057258
Na 1.288241 1.064743 2.060313
Cl 0.067285 -1.125379 3.479109
Na -1.972959 -1.063877 1.652677
Cl 0.067285 -1.125379 -3.479109
Cl -3.617256 0.360560 -0.000000
Na -1.754446 2.293705 -0.000000

A8h
Cl -0.626964 0.034936 -0.000000
Na -2.121451 0.485439 2.242777
Cl -2.337474 -1.786693 3.381488
Na -0.161710 -2.437767 2.085405
Cl 2.171328 -0.944134 2.546083
Na 1.344092 1.493253 2.054263
Cl 2.171328 -0.944134 -2.546083
Na 1.344092 1.493253 -2.054263
Cl -1.028433 2.878256 -2.544094
Na -0.459488 3.387450 0.000000
Cl -1.028433 2.878256 2.544094
Na 2.383469 -1.661692 -0.000000
Cl 0.791793 -3.817280 -0.000000
Na -2.121451 0.485439 -2.242777
Cl 2.194667 2.964916 0.000000
Na -0.161710 -2.437767 -2.085405
Cl -2.337474 -1.786693 -3.381488

A8i
Cl 1.014424 -2.145830 -1.110384
Na 3.615841 -1.939113 -0.666322
Cl 3.551867 -1.212167 1.858875
Na 0.853204 -1.511299 1.639015
Cl -1.716633 -2.238386 1.808359
Na -2.177832 0.247453 2.429835
Cl 0.363494 1.082372 2.337472
Na 3.033023 1.289601 0.759669
Cl 4.041435 0.314415 -1.838127
Na 1.353386 0.660105 -2.101629
Cl 1.652932 3.056875 -0.700691
Na -0.746183 2.421084 0.218450
Cl -3.414780 2.159885 1.078695
Na -1.760210 -1.917453 -0.964585
Cl -1.333943 0.793738 -1.925529
Cl -4.338868 -1.775405 -1.638863
Na -3.892936 0.694763 -1.113228

A8j
Cl -0.176771 0.488716 -0.618092
Na -0.431936 3.250534 -0.537316
Cl -2.857945 3.438840 0.331799
Na -3.233314 0.957059 1.010813
Cl -2.067238 -0.772390 2.701072
Na 0.413594 -0.170499 2.102131
Cl 1.175923 -2.747907 1.221451
Na 3.501595 -1.471038 1.191549
Cl 3.779727 -0.895046 -1.371374
Na 2.813035 1.566828 -0.548074
Cl 1.939838 3.870061 -1.375981
Na -1.829104 -2.322274 0.423149
Cl -4.033539 -0.950807 -0.688868
Na -2.058817 -0.498983 -2.303353
Cl 2.873141 0.880812 2.154996
Cl -0.956683 -2.891043 -2.309409
Na 1.324972 -1.962628 -1.409362

A8k
Cl 0.000000 -0.000000 1.519292
Na 0.000000 1.789409 3.614261
Cl 0.000000 3.592572 1.721472
Na -1.607176 1.932858 0.047412
Cl -3.238592 0.000000 -0.888489
Na -1.607176 -1.932858 0.047412
Cl 0.000000 -2.141159 -2.051113
Na 1.607176 -1.932858 0.047412
Cl 3.238592 -0.000000 -0.888489
Na -0.000000 -1.789409 3.614261
Cl 0.000000 -0.000000 5.493871
Na -1.537417 0.000000 -3.151602
Cl 0.000000 2.141159 -2.051113
Na 1.607176 1.932858 0.047412
Cl -0.000000 -3.592572 1.721472
Cl 0.000000 0.000000 -5.298349
Na 1.537417 -0.000000 -3.151602

A8l
Cl 1.898015 -0.014167 1.584374
Na 2.061007 2.226882 -0.085150
Cl -0.059150 1.873597 -2.093905
Na -2.327579 0.200756 -1.842143
Cl -0.768204 -1.894089 -1.188995
Na -3.157195 -1.540894 0.554706
Cl -4.590830 -1.143623 -1.615349
Na 4.054519 -1.009480 0.354852
Cl 3.855040 0.811929 -1.538905
Na 1.488171 -0.639046 -1.725105
Cl 2.878188 -3.201873 -0.293557
Na 0.602851 -2.551676 1.036956
Cl -1.480398 -1.904863 2.661692
Na -0.637934 0.675423 2.133729
Cl 0.567110 3.661431 1.576628
Cl -2.820550 1.398192 0.746275
Na -1.279001 3.277031 -0.177879

A8m
Cl 1.610361 -1.183973 -2.258147
Na 0.274679 -3.691070 -0.555742
Cl -1.311365 -4.092059 -2.555138
Na -1.143905 -1.481793 -2.485136
Cl -1.610361 1.183973 -2.258147
Na -1.882290 0.865480 0.340768
Cl -1.326429 -1.654266 0.716775
Na 0.000000 -2.001256 3.012194
Cl 1.912881 -3.245365 1.574463
Na 1.882290 -0.865480 0.340768
Cl -0.000000 -0.000000 4.640221
Na 0.000000 2.001256 3.012194
Cl -1.912881 3.245365 1.574463
Na 1.143905 1.481793 -2.485136
Cl 1.326429 1.654266 0.716775
Cl 1.311365 4.092059 -2.555138
Na -0.274679 3.691070 -0.555742

A8n
Cl 1.556473 -0.163623 -0.455219
Na 3.813749 1.458748 -0.514488
Cl 2.583699 3.715494 -0.612882
Na 0.320059 2.816185 0.345124
Cl -0.587734 1.748338 2.647395
Na 0.181272 -0.717841 1.962668
Cl -2.182449 -1.622333 1.133760
Na -0.472454 -2.907107 -0.414299
Cl -1.571773 -1.991281 -2.701888
Na 3.430230 -2.043733 0.263468
Cl 5.473104 -0.541279 -0.181849
Na -2.941708 1.177638 1.321396
Cl -1.799326 2.013038 -1.163640
Na -0.271573 0.268436 -2.288122
Cl 1.462913 -3.352350 1.388914
Cl -5.286800 0.620410 0.233912
Na -3.515739 -0.711331 -1.121616

A8o
Na 2.860166 -1.951113 0.386007
Cl 4.966388 -0.702680 -0.494996
Na 3.665489 1.445624 -1.105293
Cl 1.066447 0.492261 -0.719907
Na -0.435452 -1.508004 -1.774233
Cl 0.878913 -3.559936 -0.619512
Na -0.394875 -2.952589 1.573966
Cl 1.527554 -1.670493 2.838844
Na 0.250781 0.431973 1.876371
Cl -2.151621 -0.984183 1.088384
Na -4.029618 -0.664836 -1.102755
Cl -2.661145 -1.501895 -3.097116
Cl -5.270633 1.348043 -0.028444
Na -3.099523 1.629963 1.301121
Cl -0.858258 2.895007 1.820612
Cl 2.890060 3.941525 -1.265225
Na 0.583851 3.170798 -0.417447

A8p
Cl -0.000000 0.000000 0.936206
Na -0.000000 1.829156 3.484278
Cl -0.000000 0.000000 5.274195
Na 0.000000 -3.195605 0.159938
Cl 2.030836 -2.659153 -1.581244
Na 1.877532 -0.000000 -1.204273
Cl 0.000000 -0.000000 -3.889585
Na -0.000000 2.498286 -3.257080
Cl -2.030836 2.659153 -1.581244
Na 0.000000 3.195605 0.159938
Cl -0.000000 4.193458 2.530817
Na -0.000000 -2.498286 -3.257080
Cl -2.030836 -2.659153 -1.581244
Na -1.877532 0.000000 -1.204273
Cl 2.030836 2.659153 -1.581244
Na -0.000000 -1.829156 3.484278
Cl -0.000000 -4.193458 2.530817

A8q
Cl -1.646105 2.619845 0.000000
Na -3.041192 0.418425 0.000000
Cl -3.132204 -0.483180 2.495245
Na -0.721678 0.945716 2.705316
Cl 1.700271 2.026543 2.436308
Na 2.436119 -0.583807 1.976122
Cl -0.172737 -1.007913 0.000000
Na -1.153477 -2.201287 -2.184989
Cl -3.132204 -0.483180 -2.495245
Na -0.721678 0.945716 -2.705316
Cl 0.659895 -1.466731 -3.832580
Na 2.436119 -0.583807 -1.976122
Cl 3.967340 -1.315168 -0.000000
Na 0.985158 2.564831 -0.000000
Cl 1.700271 2.026543 -2.436308
Cl 0.659895 -1.466731 3.832580
Na -1.153477 -2.201287 2.184989

A8r
Na 0.026815 2.081287 0.913254
Cl 1.968823 1.149921 2.806875
Na 3.736287 1.332262 0.809214
Cl 5.003018 -0.748681 -0.067152
Na 2.926917 -1.936287 -1.035706
Cl 1.065562 -1.009502 -2.706114
Na -0.758464 -1.886721 -0.883129
Cl 1.202733 -3.166748 0.614796

Na 0.984325 -1.230642 2.375757
Cl -1.351036 -0.285488 1.506341
Na -3.543465 0.895756 -0.887673
Cl -3.383637 -1.872340 -1.254014
Cl -1.513895 2.452301 -1.388082
Na 0.762438 1.562529 -2.381100
Cl 2.222155 2.890458 -0.660226
Cl -5.385315 0.844340 1.014669
Na -3.869665 -1.211133 1.294784

A8s
Cl -1.222089 0.604423 -0.017373
Na -2.908239 2.726937 0.132367
Cl -0.984640 4.469779 -0.258720
Na 0.731447 2.476340 -0.475035
Cl 1.717452 0.853372 -2.515277
Na -0.361857 -0.825045 -2.111702
Cl 1.390876 -2.509158 -0.187178
Na -1.027408 -3.573326 0.061399
Cl -1.899556 -2.448969 2.257170
Na -3.467366 -0.938154 0.347427
Cl -5.010326 1.203417 0.488782

Na 3.202866 -0.657260 -0.922696
Cl 2.103517 1.199602 1.774815
Na 0.218574 -0.793755 1.753721
Cl -2.440832 -2.557290 -1.884791
Cl 5.699426 -0.396020 -0.029215
Na 4.610611 0.936475 1.789100

A8t
Cl -1.221904 0.603718 0.014295
Na -2.910374 2.725885 -0.130516
Cl -0.987572 4.469636 0.260122
Na 0.730464 2.476648 0.472045
Cl 2.100402 1.202787 -1.778859
Na 0.221461 -0.795478 -1.754755
Cl 1.391776 -2.507205 0.191898
Na -1.025088 -3.573114 -0.060371
Cl -2.438820 -2.557279 1.885519
Na -3.464963 -0.939584 -0.348675
Cl -5.010707 1.199890 -0.488269
Na 3.204321 -0.656202 0.929110
Cl 1.715627 0.856236 2.515456
Na -0.360745 -0.824562 2.110012

Cl -1.895081 -2.452407 -2.257982
Na 4.606684 0.935721 -1.789854
Cl 5.698081 -0.394344 0.028589

A8u
Cl 1.435179 -0.629829 -1.141432
Na 2.374142 -3.026245 -0.261186
Cl 0.477652 -4.515773 -1.180756
Na -0.848047 -2.212434 -1.320814
Cl -1.356633 -1.512602 1.415126
Na -3.016249 1.032702 -0.332447
Cl -1.429011 3.157367 -0.499621
Na 0.082705 1.486739 -2.120132
Cl 2.525472 2.925372 -1.523199
Na 0.928996 3.508807 0.517492
Cl 1.972832 1.983469 2.350140
Na 3.339027 0.898322 0.145902
Cl 3.583951 -1.579235 1.589948
Na 1.027542 -0.432406 1.828269
Cl -2.112374 -0.188263 -2.542328
Na -3.765491 -1.373600 2.133451
Cl -5.176415 0.435921 1.15001

6.4 Structures Anionic Clusters: ωB97XD/aug-cc-pVDZ

A1a
Cl -0.000000 0.000000 -2.542373
Na 0.000000 0.000000 0.000000
Cl -0.000000 0.000000 2.542373

A2a
Na 0.000000 2.492163 0.127058
Cl 0.000000 -0.000000 0.857115
Cl 0.000000 -4.908178 -0.510771
Na -0.000000 -2.492163 0.127058
Cl 0.000000 4.908178 -0.510771

A2b
Na 0.000000 0.000000 1.369881
Cl -0.000000 2.326128 0.000000
Na 0.000000 -0.000000 -1.369881
Cl -2.014486 -1.163064 0.000000
Cl 2.014486 -1.163064 0.000000

A2c
Cl 0.000000 2.091337 -1.144909
Na -0.000000 -0.000000 -2.520825
Cl -0.000000 -2.091337 -1.144909
Na 0.000000 0.000000 0.827760
Cl 0.000000 0.000000 3.385331

A3a
Cl -2.441127 1.409385 -0.779135
Na 0.000000 1.899317 0.133092
Na -1.644857 -0.949659 0.133092
Cl 2.441127 1.409385 -0.779135
Cl -0.000000 -2.818770 -0.779135
Na 1.644857 -0.949659 0.133092
Cl 0.000000 0.000000 2.079050

A3b
Cl 0.000000 1.490348 -0.000000
Na -2.388448 2.313411 0.000000
Cl -3.947159 0.318450 0.000000
Na -1.488965 -0.843880 0.000000
Cl 0.409317 -2.565455 0.000000
Na 2.171378 -0.503754 -0.000000
Cl 4.641747 0.131743 -0.000000

A3c
Cl 2.753781 1.254319 0.000000
Na 2.239264 3.717258 0.000000
Cl -0.257835 4.051110 0.000000
Na -0.000000 1.301193 0.000000
Cl -1.715253 -0.684715 0.000000
Na -1.383405 -3.253026 0.000000
Cl -1.334484 -5.763048 0.000000

A3d
Na 1.554547 4.651864 0.000000
Cl -0.000000 2.552964 0.000000
Na 0.000000 0.000000 0.000000
Cl -0.000000 -2.552964 0.000000
Na -1.554547 -4.651864 0.000000
Cl -3.055091 -6.636617 -0.000000
Cl 3.055091 6.636617 -0.000000

A4a
Cl 2.580216 2.580216 -0.393249
Na 0.000000 2.490183 0.103183
Cl -2.580216 2.580216 -0.393249
Na -2.490183 0.000000 0.103183
Cl -2.580216 -2.580216 -0.393249
Na -0.000000 -2.490183 0.103183
Cl 2.580216 -2.580216 -0.393249
Na 2.490183 -0.000000 0.103183
Cl 0.000000 0.000000 1.305936

A4b
Na -0.485493 1.757660 -1.509787
Cl 0.000000 3.775963 0.049352
Na 1.105530 1.620090 1.165834
Cl -0.000000 -0.000000 2.897261
Na -1.105530 -1.620090 1.165834
Cl -0.000000 -3.775963 0.049352
Cl -1.901488 -0.473136 -1.275426
Cl 1.901488 0.473136 -1.275426
Na 0.485493 -1.757660 -1.509787

A4c
Cl 2.179748 -3.132719 -0.000000
Na 0.553233 -1.743321 1.538129
Na 0.553233 -1.743321 -1.538129
Cl -1.700563 -1.905384 -0.000000
Cl 0.553233 0.835893 -2.051693
Na 0.726838 2.566829 0.000000
Na -1.580967 0.779311 0.000000
Cl 0.553233 0.835893 2.051693
Cl -1.748927 3.457230 0.000000

A4d
Cl 0.000000 2.425582 0.056483
Na 1.674445 0.966741 -1.322999
Cl 2.100616 -1.212791 0.056483
Na -1.674445 0.966741 -1.322999
Cl -2.100616 -1.212791 0.056483
Na -0.000000 -1.933482 -1.322999
Cl 0.000000 0.000000 -3.239688
Na 0.000000 0.000000 1.871103
Cl 0.000000 0.000000 4.427701

A4e
Na 0.375900 -2.879193 -0.000000
Cl 1.385346 -0.261461 -0.000000
Na -0.774679 -0.235982 1.711797
Cl -1.767393 1.580808 0.000000
Na -0.774679 -0.235982 -1.711797
Cl -0.774679 -2.743636 -2.361524
Cl -0.774679 -2.743636 2.361524
Na 0.858225 2.533755 0.000000
Cl 2.135379 4.696833 0.000000

A4f
Cl 2.032407 -1.437626 0.000130
Na 4.521415 -1.940375 0.000310
Cl 5.775630 0.257898 0.000528
Na 3.216545 1.104180 0.000204
Cl 1.052411 2.492459 -0.000343
Na -0.266747 0.191824 -0.000572
Cl -2.669096 -0.846330 -0.001278
Na -5.190052 -0.260212 -0.000081
Cl -7.667397 0.118917 0.001052

A4g
Cl -2.406946 1.672576 0.003161
Na -4.756445 0.677308 -0.004741
Cl -4.552197 -1.837764 -0.002965
Na -1.961329 -1.162830 0.003651
Cl 0.619727 -1.077909 0.004140
Na 0.341200 1.567475 0.002029
Cl 2.739678 2.424209 -0.002814
Na 3.458897 -0.207579 -0.000407
Cl 5.487647 -1.746883 -0.001866

A4h
Cl 4.222469 0.002686 -2.013149
Na 2.347594 -0.499097 -0.012922
Cl -0.047559 -1.547811 -0.076614
Na -2.399615 -0.414448 -0.036485
Cl -4.974158 -1.462629 0.175881
Na 5.436324 0.672589 0.083721
Cl 3.991767 0.268459 2.099774
Na -5.378326 1.012156 0.004820
Cl -3.196387 2.240283 -0.211215

A4i
Na -7.350683 -0.101246 -0.000000
Cl -9.691337 0.727719 0.000000
Cl -4.864605 -0.916856 -0.000000
Na -2.458694 -0.097764 -0.000000
Cl 0.000000 0.634982 0.000000
Na 2.458583 -0.097709 -0.000000
Cl 4.864518 -0.916349 -0.000000
Na 7.350727 -0.101041 -0.000000
Cl 9.691467 0.727878 0.000000

A5a
Na 1.620923 -0.000000 -2.241018
Cl 0.000000 2.086222 -2.637625
Cl -0.000000 -2.086222 -2.637625
Na -1.620923 0.000000 -2.241018
Cl -1.950667 0.000000 0.329863
Na 0.000000 -2.028926 0.202933
Cl 1.950667 -0.000000 0.329863
Na 0.000000 2.028926 0.202933
Cl 0.000000 2.657848 2.767502
Na 0.000000 0.000000 2.655156
Cl -0.000000 -2.657848 2.767502

A5b
Cl 2.412725 -0.000000 0.069203
Na 1.599777 2.508001 -0.320328
Cl 0.000000 3.676990 -2.042257
Na -1.599777 2.508001 -0.320328
Na 1.599777 -2.508001 -0.320328
Cl -0.000000 -3.676990 -2.042257
Na -1.599777 -2.508001 -0.320328
Cl -2.412725 0.000000 0.069203
Cl 0.000000 -2.681712 1.865790
Na 0.000000 0.000000 1.612853
Cl 0.000000 2.681712 1.865790

A5c
Cl 2.530318 3.482683 0.000000
Na 0.000000 2.992731 -0.000000
Cl -2.530318 3.482683 -0.000000
Na 2.846256 0.924805 -0.000000
Cl 4.094140 -1.330267 0.000000
Na 1.759083 -2.421170 -0.000000
Cl -0.000000 -4.304833 0.000000
Na -1.759083 -2.421170 -0.000000
Cl -4.094140 -1.330267 0.000000
Na -2.846256 0.924805 -0.000000
Cl 0.000000 0.000000 0.000000

A5e
Cl -0.892258 -0.841550 -2.343339
Na -2.676933 -1.313824 -0.480131
Cl -1.162722 -2.301893 1.344304
Cl -3.111190 1.160179 0.368786
Na -1.191948 1.624695 -1.472798
Cl 0.556811 2.768553 0.072074
Na 2.688964 1.149882 0.150567
Cl 3.282132 -0.792735 -1.477996
Cl 1.828978 -0.206517 2.232325
Na 1.078964 -1.671406 -0.200697
Na -0.674481 0.541322 1.699914

A5f
Na 0.000000 1.955998 -0.000000
Cl -2.002877 1.148030 1.849362
Na 0.000000 0.000000 3.040142
Cl 1.995662 1.160527 1.849362
Na 1.693944 -0.977999 -0.000000
Cl 0.007215 -2.308557 1.849362
Na -1.693944 -0.977999 -0.000000
Cl -1.995662 1.160527 -1.849362
Na 0.000000 0.000000 -3.040142
Cl -0.007215 -2.308557 -1.849362
Cl 2.002877 1.148030 -1.849362

A5g
Na 0.883630 0.005024 1.393244
Cl 0.430027 -2.999051 0.633469
Na 1.954056 -1.813080 -1.052547
Cl 3.485259 -0.000421 0.162520
Na 1.956503 1.811367 -1.057327
Cl 0.433803 2.994887 0.634324
Cl -1.611097 -0.000576 2.172549
Na -1.824815 1.558108 -0.055721
Cl 0.189111 -0.000342 -1.845621
Na -1.823270 -1.556939 -0.059581
Cl -3.668701 0.002603 -1.218933

A5h
Na -0.575056 -0.691518 2.214512
Cl 0.166994 1.866089 2.037463
Na 2.060237 2.526803 0.000000
Cl 4.325993 3.620200 0.000000
Cl -0.575056 -3.273058 2.497875
Na -1.334532 -3.303681 -0.000000
Cl -0.575056 -3.273058 -2.497875
Na -0.575056 -0.691518 -2.214512
Cl 0.166994 1.866089 -2.037463
Cl -2.290532 -0.633757 -0.000000
Na -1.460026 1.893314 0.000000

A5i
Cl -0.716483 1.253346 3.654870
Na 0.313123 -0.255480 1.819691
Na -0.716483 3.101162 1.781258
Cl -1.646994 4.771941 0.000000
Na -0.716483 3.101162 -1.781258
Cl -0.716483 1.253346 -3.654870
Na 0.313123 -0.255480 -1.819691
Cl 0.903895 1.661126 0.000000
Cl 0.394542 -2.265903 -0.000000
Na 0.987258 -4.842453 -0.000000
Cl 1.664703 -7.223152 -0.000000

A5j
Cl 2.761285 3.279905 -0.000000
Na 1.653514 1.747799 1.785581
Cl 1.653514 -0.236601 3.500186
Na 0.193940 -1.508602 1.808862
Cl -1.093529 -2.985944 0.000000
Na 0.193940 -1.508602 -1.808862
Cl 1.653514 -0.236601 -3.500186
Na 1.653514 1.747799 -1.785581
Cl -0.255484 0.568731 -0.000000
Na -2.856095 -0.816784 -0.000000
Cl -5.262061 -0.170529 -0.000000

A5k
Na 0.000000 2.026260 -0.065031
Cl 0.000000 0.000000 1.915043
Na -1.754792 -1.013130 -0.065031
Cl -0.000000 -2.430211 -1.337290
Na 1.754792 -1.013130 -0.065031
Cl 2.104624 1.215105 -1.337290
Cl -2.104624 1.215105 -1.337290
Na 0.000000 0.000000 -3.179119
Cl 0.000000 0.000000 -5.713290
Na 0.000000 0.000000 4.569785
Cl 0.000000 0.000000 7.036511

A6a
Cl 0.767935 1.376814 3.669822
Na 0.732072 -0.670675 1.998473
Cl -0.995746 -2.913817 2.046467
Na 0.544106 -3.616700 -0.000000
Cl -0.995746 -2.913817 -2.046467
Na 0.732072 -0.670675 -1.998473
Cl 0.767935 1.376814 -3.669822
Na -0.255386 2.821783 -1.779026
Cl -0.566186 4.714637 0.000000
Na -0.255386 2.821783 1.779026
Cl -0.914658 0.713933 0.000000
Na -2.113085 -1.660609 -0.000000
Cl 2.334801 -1.723621 -0.000000

A6b
Cl 2.363145 1.957184 0.374118
Na 0.000000 2.874885 -0.377929
Cl -2.363145 1.957184 0.374118
Cl 0.000000 1.859083 -2.776605
Na 1.732212 -0.000000 -1.637835
Na -1.732212 0.000000 -1.637835
Na 1.892862 -0.000000 2.120652
Cl -0.000000 -1.859083 -2.776605
Na -0.000000 -2.874885 -0.377929
Cl -2.363145 -1.957184 0.374118
Cl 2.363145 -1.957184 0.374118
Na -1.892862 0.000000 2.120652
Cl 0.000000 0.000000 3.921001

A6c
Na 1.859948 0.841790 0.000000
Cl 1.952937 -1.839799 -0.000000
Cl -1.548052 4.255371 0.000000
Cl 1.433859 1.476981 2.608192
Na -0.900846 2.383924 -1.692710
Cl -1.072438 0.245836 0.000000
Na 0.544241 -1.081877 -2.255770
Cl -1.163973 -3.003298 -2.626583
Na -0.949261 -2.841263 -0.000000
Cl -1.163973 -3.003298 2.626583
Na 0.544241 -1.081877 2.255770
Na -0.900846 2.383924 1.692710
Cl 1.433859 1.476981 -2.608192

A6d
Cl 0.279180 0.931139 2.486246
Na -0.447829 -1.610371 2.327975
Cl -1.891374 -1.411069 0.000000
Na -0.995648 1.188898 -0.000000
Cl -0.765762 3.875262 -0.000000
Na 1.296707 3.260336 1.631292
Cl 3.262023 3.847681 -0.000000
Na 1.296707 3.260336 -1.631292
Cl 0.279180 0.931139 -2.486246
Na -0.447829 -1.610371 -2.327975
Cl -0.447829 -4.209547 -2.528611
Cl -0.447829 -4.209547 2.528611
Na -1.115656 -4.110280 0.000000

A6e
Cl 1.863259 0.622181 1.841107
Na 2.002050 -1.872196 0.232350

Cl 3.972694 -0.912326 -1.238019
Na 2.158217 1.038842 -1.063833
Cl 0.356848 3.142461 -1.227467
Na -1.948069 1.241963 -1.028859
Cl 0.021099 -0.648121 -1.277054
Na -0.572164 -0.541166 1.683706
Cl -0.294203 -3.191477 1.318331
Na -1.908964 -2.401409 -0.752769
Cl -3.791156 -0.740147 -1.417857
Cl -2.113337 1.628153 1.734854
Na 0.245433 2.687392 1.340656

A6f
Cl 0.000000 0.000000 2.945331
Na 1.799504 1.038944 1.210960
Cl 2.359566 1.362296 -1.723830
Na 0.000000 2.517445 -1.548148
Cl -2.359566 1.362296 -1.723830
Na -2.180172 -1.258723 -1.548148
Cl -0.000000 -2.724593 -1.723830
Na -0.000000 -2.077888 1.210960
Cl 0.000000 3.188479 0.960233
Na -1.799504 1.038944 1.210960
Cl -2.761304 -1.594239 0.960233
Na 2.180172 -1.258723 -1.548148
Cl 2.761304 -1.594239 0.960233

A6g
Cl 2.718063 1.569275 1.535744
Na 0.000000 2.385087 1.159132
Cl -2.718063 1.569275 1.535744
Na -2.065546 1.192544 -1.159132
Cl 0.000000 3.138549 -1.535744
Na 2.065546 1.192544 -1.159132
Cl 2.718063 -1.569275 -1.535744
Na 2.065546 -1.192544 1.159132
Cl -0.000000 -3.138549 1.535744
Na -0.000000 -2.385087 -1.159132
Cl 0.000000 0.000000 0.000000
Na -2.065546 -1.192544 1.159132
Cl -2.718063 -1.569275 -1.535744

A6h
Cl 0.005643 3.636879 2.423568
Na 1.709312 1.705450 2.002699
Cl 2.799550 2.360868 -0.334760
Na 0.000000 2.985736 -0.162400
Na -1.507974 -0.504617 -1.745034
Cl -0.000000 0.000000 0.734674
Na -1.709312 -1.705450 2.002699
Cl -2.799550 -2.360868 -0.334760
Cl -0.005643 -3.636879 2.423568
Na -0.000000 -2.985736 -0.162400
Cl 0.751672 -1.953078 -2.517786
Cl -0.751672 1.953078 -2.517786
Na 1.507974 0.504617 -1.745034

A6i
Na -0.800003 1.659678 -0.344618
Cl -1.929933 -0.075492 1.744947
Na 0.769886 -0.347090 1.591801
Cl 3.201364 -1.435508 1.565848
Na 2.871938 -1.548034 -1.116575
Cl 0.219991 -1.101533 -1.146092
Na -4.185284 0.576917 0.214144
Cl -3.146550 2.526606 -1.149781
Cl 1.500106 2.203576 1.004207
Na 3.804219 1.007672 0.445648
Cl 4.724232 0.133705 -1.848271
Cl -4.753594 -1.900009 -0.510633
Na -2.175799 -1.892133 -0.265293

A6j
Na -1.685059 -2.996161 0.000000
Cl -1.923859 -0.409936 0.000000
Cl -0.078473 -3.387921 2.070351
Na 1.601305 -3.445847 0.000000
Cl 2.502688 -1.036657 0.000000
Cl -0.078473 -3.387921 -2.070351
Na 0.409064 -0.525597 -1.698637
Cl 0.409064 2.091338 -2.091412
Na 0.972473 3.710409 -0.000000
Cl 0.409064 2.091338 2.091412
Na 0.409064 -0.525597 1.698637
Cl -1.344109 4.990747 -0.000000
Na -1.545968 2.313087 -0.000000

A6k
Cl 2.596483 -0.493564 0.000000
Na 1.962674 2.143093 0.000000
Cl 0.483379 2.252520 2.125361
Na -1.599286 2.578639 0.000000
Cl -3.624209 4.130950 0.000000
Na 1.983319 -3.042925 0.000000
Cl 0.337183 -3.208733 2.065728
Na -1.348977 -2.965040 0.000000
Cl 0.337183 -3.208733 -2.065728
Na 0.337183 -0.387130 -1.834515
Cl 0.483379 2.252520 -2.125361
Na 0.337183 -0.387130 1.834515
Cl -1.695343 -0.391699 0.000000

A6m
Na -3.689655 -1.065645 -0.024740
Cl -2.310594 -0.824457 2.219623
Cl -3.739588 1.454732 -0.834065
Cl -1.877105 -2.064690 -1.545785
Na -1.085581 0.663899 -1.683761
Na -0.058976 -1.560205 0.484259
Na -2.276432 1.684404 1.399009
Cl 1.449106 -0.408326 -1.776365
Na 2.333057 1.207465 0.422700
Cl 2.280379 -1.433974 1.745338
Cl -0.057384 2.435760 0.307799
Cl 4.954706 1.031271 -0.175723
Na 3.696512 -1.224043 -0.506009

A6o
Cl 0.086789 1.111214 2.340501
Na 0.086789 3.687758 1.602640
Cl 1.696256 4.981269 -0.000000
Na 0.086789 3.687758 -1.602640
Cl 0.086789 1.111214 -2.340501
Na 0.768089 -1.309557 -1.667998
Cl 2.201496 -2.811560 0.000000
Na 0.768089 -1.309557 1.667998
Cl -2.089733 3.527508 -0.000000
Na -1.492912 0.915293 -0.000000
Cl -1.331512 -1.864368 0.000000
Na -0.072004 -4.441037 0.000000
Cl -0.743806 -6.851585 0.000000

A6p
Cl 1.720791 -3.822242 0.000000
Na 1.243388 -1.257342 0.000000
Cl 1.446275 1.722463 0.000000
Na 3.371691 3.556241 0.000000
Cl 4.843799 5.535847 0.000000
Na -0.719669 1.418400 1.683354
Cl -0.719669 -1.143794 1.940219
Cl -2.693607 1.908341 0.000000
Na -2.770153 -0.945875 0.000000
Cl -3.548281 -3.448470 0.000000
Na -0.915029 -3.584548 0.000000
Cl -0.719669 -1.143794 -1.940219
Na -0.719669 1.418400 -1.683354

A6q
Cl 4.645633 2.427724 0.758265
Na 2.150472 2.882884 0.355526
Cl -0.018729 4.027448 -0.451497
Na -0.998351 1.675691 -0.625233
Cl 1.207618 0.000190 0.000701
Na 2.149620 -2.883488 -0.354756
Cl -0.018261 -4.026835 0.457548
Na -0.996543 -1.674294 0.630603
Cl -2.962016 -0.000293 0.002815
Na -5.625074 -0.000582 -0.001065
Cl -8.095503 0.000090 -0.004895
Cl 4.643618 -2.428332 -0.764307
Na 4.243501 -0.000198 -0.002958

A7a
Na -1.933672 0.000000 1.065376
Cl -2.103424 2.735603 0.967090
Na 0.000000 2.553864 2.495617
Cl 2.103424 2.735603 0.967090
Na 1.933672 -0.000000 1.065376
Cl 2.103424 -2.735603 0.967090
Na -0.000000 -2.553864 2.495617
Cl -2.103424 -2.735603 0.967090
Na 0.000000 -2.722767 -1.013788
Cl 0.000000 -0.000000 -0.800598
Na 0.000000 2.722767 -1.013788
Cl 0.000000 -2.630366 -3.678664
Cl -0.000000 0.000000 3.282475
Cl 0.000000 2.630366 -3.678664
Na 0.000000 -0.000000 -3.537998

A7b
Na 2.411311 -1.757951 -0.035052
Cl 0.419233 -3.507512 -0.782653
Cl 1.466454 -1.018483 2.584733
Na 0.543492 1.395014 1.733965
Cl 1.426771 3.543506 0.083131
Na -0.966020 2.462379 -0.888555
Cl -2.829325 0.904174 -2.255742
Na -3.446581 0.465874 0.292359
Cl -2.048611 2.230293 1.629130
Cl 0.503387 0.152437 -0.815997
Cl -3.072764 -2.100682 0.563011
Na -1.446444 -1.556030 -1.646095
Na -0.544739 -2.270869 1.389266
Na 2.846637 1.560452 -0.950629
Cl 4.524608 -0.397120 -0.937838

A7c
Na -0.000000 1.983840 1.624833
Cl -0.000000 2.634182 4.175321
Na -0.000000 -0.000000 4.074031
Cl -0.000000 -2.634182 4.175321
Na -0.000000 -1.983840 1.624833
Cl 0.000000 -2.209034 -1.170015
Na 0.000000 -1.678928 -3.723605
Cl 2.057080 -0.000000 -3.875506
Na 0.000000 1.678928 -3.723605
Cl -2.057080 0.000000 -3.875506
Na -1.758480 0.000000 -1.051057
Cl -1.945418 0.000000 1.590257
Cl 0.000000 2.209034 -1.170015
Na 1.758480 -0.000000 -1.051057
Cl 1.945418 -0.000000 1.590257

A7d
Na 0.013124 1.351285 1.344476
Cl -1.959599 3.245270 0.293945

Na -3.162413 0.951123 -0.134692
Cl -4.156441 -0.906652 -1.662503
Na -1.901503 -1.882432 -0.735540
Cl -2.051748 -0.531992 1.924971
Na -0.018461 -2.176350 2.131493
Cl 2.047694 -0.570910 1.918730
Na 3.214703 0.892244 -0.114482
Cl 2.033014 3.200977 0.292936
Na 0.031975 2.878887 -1.378604
Cl -0.017666 0.175152 -1.310096
Na 1.846295 -1.898966 -0.752208
Cl 4.123042 -0.973838 -1.681715
Cl -0.033644 -3.712930 -0.009496

A7f
Na -1.440490 2.022881 -0.974059
Cl -0.747874 -0.668617 -1.977331
Na -3.194762 -1.323076 -1.206824
Cl -3.917636 1.000767 -0.354147
Na -1.992597 0.063772 1.596210
Cl -1.076906 2.622086 1.695161
Na 1.434171 2.022546 0.977821
Cl 1.068431 2.630597 -1.688349
Na 1.994183 0.076819 -1.598389
Cl 2.535970 -2.674356 -0.950497
Na 3.195712 -1.320245 1.205748
Cl 3.914063 1.006556 0.355307
Cl -2.526312 -2.682866 0.943481
Na 0.004564 -2.403077 -0.004384
Cl 0.749757 -0.677450 1.978882

A7h
Na -1.992587 -1.722669 -0.786420
Cl -4.500954 -1.104018 -1.279641
Na -3.791554 0.931400 0.190660
Cl -2.780125 3.317714 0.395956
Na -0.579097 1.886613 0.726453
Cl -0.088907 0.204693 -1.539467
Na 1.940333 1.836283 -1.861522
Cl 3.900223 0.052023 -1.682439
Na 1.874183 -1.670615 -0.854746
Cl 2.655164 -1.446309 1.887295
Na 0.107432 -2.118197 1.935718
Cl -1.866500 -0.385680 1.819908
Cl -0.025252 -3.545164 -0.304000
Cl 2.010937 2.944053 0.557172
Na 3.516022 0.799520 0.874283

A7i
Na 0.000000 2.348301 -1.147552
Cl -2.134014 1.232073 -2.668446
Cl 2.134014 1.232073 -2.668446
Cl 0.000000 0.000000 0.105846
Na 0.000000 0.000000 -3.597215
Cl -0.000000 -2.464147 -2.668446
Na 2.033689 -1.174151 -1.147552
Cl 2.788314 -1.609834 1.448993
Na 1.635738 0.944394 2.100931
Cl 0.000000 0.000000 4.029448
Na -1.635738 0.944394 2.100931
Cl -2.788314 -1.609834 1.448993
Na -2.033689 -1.174151 -1.147552
Cl 0.000000 3.219668 1.448993
Na -0.000000 -1.888788 2.100931

A7j
Cl 0.634738 -3.789880 0.000000
Na -1.865317 -3.147790 0.000000
Cl -1.960512 -1.878740 2.316156
Na 0.991389 -1.593509 1.635792
Na 0.991389 -1.593509 -1.635792
Cl -1.960512 -1.878740 -2.316156
Na -1.960512 0.698035 -1.900568
Na -1.960512 0.698035 1.900568
Cl 0.617505 1.141582 2.028407
Cl 0.617505 1.141582 -2.028407
Na 2.652628 1.522083 0.000000
Cl 3.172619 -1.071704 0.000000
Cl -2.730641 2.405259 0.000000
Na -0.011446 3.047520 0.000000
Cl 2.361425 4.169492 0.000000

A7k
Na 1.936897 3.712667 0.000000
Cl 2.225765 1.148944 0.000000
Cl 0.286080 4.051396 2.075170
Cl 0.286080 4.051396 -2.075170
Na -1.358539 3.904154 0.000000
Cl -2.004158 1.375312 0.000000
Na 0.188900 1.187934 1.807786
Cl -0.456953 -1.312313 2.520786
Na 0.286080 -3.715075 1.620895
Cl -1.860508 -4.059355 -0.000000
Na -1.711754 -1.368696 -0.000000
Cl -0.456953 -1.312313 -2.520786
Na 0.188900 1.187934 -1.807786
Cl 2.099341 -4.715552 -0.000000
Na 0.286080 -3.715075 -1.620895

A7n
Na 0.439903 -3.608827 1.665494
Cl 1.401460 -1.600440 0.000000
Cl -1.369320 -1.896635 2.541205
Na -1.695053 -1.132458 0.000000
Cl -0.909017 1.497826 -0.000000
Cl -1.369320 -1.896635 -2.541205
Na 0.452712 0.146869 2.111644
Cl 1.090663 2.214695 3.558426
Na -0.106506 3.652734 1.760315
Cl -0.393409 5.547060 -0.000000
Na -0.106506 3.652734 -1.760315
Cl 1.090663 2.214695 -3.558426
Na 0.452712 0.146869 -2.111644
Na 0.439903 -3.608827 -1.665494
Cl 0.537762 -5.594687 0.000000

A7o
Na -1.665747 2.363300 1.582344
Cl -3.702073 1.547088 -0.000000
Na -2.143346 -0.607210 -0.000000
Cl -0.162707 -2.852817 0.000000
Cl -1.665747 -0.065758 2.875391
Na 0.579702 -1.024916 2.012901
Cl 3.259103 -1.613224 -2.035801
Na 0.579702 -1.024916 -2.012901
Cl -1.665747 -0.065758 -2.875391
Na -1.665747 2.363300 -1.582344
Cl -0.893860 4.378888 -0.000000
Na 2.466562 -3.132378 0.000000
Cl 3.259103 -1.613224 2.035801
Cl 0.688172 0.887982 -0.000000
Na 3.214683 0.130637 0.000000

A7p
Na -4.466795 -1.015609 0.948818
Cl -4.063132 -1.220152 -1.752723
Na -1.512353 -1.366901 -0.982008
Cl 1.197440 -1.453310 -0.779967
Cl -2.029438 -1.744854 1.713360
Na 0.001030 -0.003841 1.492268
Cl 2.030089 1.737576 1.719183
Na 1.512466 1.371097 -0.977733
Cl -1.197945 1.455684 -0.773232
Na -3.869117 1.375512 -0.991990
Cl -5.565278 1.363623 0.992840
Na 3.867718 -1.370827 -0.996704
Cl 4.063289 1.227663 -1.748670
Cl 5.564241 -1.367765 0.987798
Na 4.468186 1.012945 0.953164

A7q
Na -3.993854 -0.787376 0.768325
Cl -2.459959 0.922402 1.941523
Cl -3.941457 0.098856 -1.751908
Na -1.989698 -1.690236 -1.681515
Cl 0.094287 -0.144427 -1.203217
Cl -2.368384 -2.898949 0.638439
Na -1.978645 1.863403 -0.590749
Cl -0.249893 3.822890 -0.516926
Na 1.465470 2.038943 0.425369
Cl 3.910819 1.357550 -0.634796
Na 3.743685 -0.754622 1.045702
Cl 1.739244 0.132631 2.504576
Na -0.509487 -0.938133 1.515381
Na 2.667220 -0.612970 -1.885132
Cl 3.660544 -2.720900 -0.717172

A7r
Na -3.545407 -1.470038 0.499906
Cl -1.696581 -3.312567 0.514718
Cl -1.930749 0.308832 1.589010
Na 0.186695 2.083974 1.138724
Cl -1.460305 4.059180 0.434350
Na -2.337113 1.886895 -0.792275
Cl 0.207702 1.154278 -1.685558
Na 2.574146 0.072455 -2.014167
Cl 1.789323 -2.083986 -0.539709
Cl -3.507356 -0.365975 -1.891169
Cl 1.851253 0.355668 2.611634
Na 0.103826 -1.535878 1.669135
Na -0.920655 -1.325373 -1.315158
Na 3.591719 -0.583525 0.882120
Cl 4.971108 0.448477 -1.077459

A7s
Na 0.431539 -0.535234 1.419872
Cl -0.077017 -2.985585 0.315636
Na -2.497770 -2.144439 -0.470960
Cl -2.854204 0.057663 -2.051794
Na -0.875409 1.790542 -1.493425
Cl 0.255123 4.171485 -1.111166
Na 1.052823 2.667595 0.869292
Cl 2.558493 0.993900 2.399897
Na 3.818661 0.028511 0.271063
Cl 1.480095 0.335370 -1.110494
Na 1.965006 -2.334034 -1.256481
Cl 4.533017 -2.239796 -0.787704
Cl -1.411988 1.433066 1.358806
Cl -4.557995 -1.797682 1.128069
Na -3.779751 0.575863 0.442347

A7t
Na -3.053600 0.119836 0.887230
Cl -1.940643 -2.374307 0.010310
Na 0.149514 -0.940905 1.817992
Cl -0.586417 0.633807 -1.163131
Na 2.037549 1.542155 -1.338468
Cl 1.460603 4.039547 -0.690169
Na 0.158443 2.489858 1.021025
Cl 2.352359 0.769553 1.422085
Na 3.507004 -1.328279 0.346671
Cl 2.927315 -0.929004 -2.213586
Cl -1.439338 1.051265 2.749151
Cl 1.730062 -3.078321 1.095382
Na 0.593056 -1.934801 -1.100942
Cl -4.895384 0.385029 -0.994708

Na -2.787009 -0.716833 -1.966296

A7u
Na -0.980922 -2.604949 -1.577047
Cl -2.693539 -0.908978 -2.444798
Cl -1.675005 -3.078656 0.877422
Na 0.271009 -1.286187 1.675511
Cl 2.855926 -0.686892 2.084284
Na 2.626591 1.358863 0.038714
Cl 0.557050 3.003279 -0.359487
Na -1.417428 2.611565 1.372622
Cl -1.592547 0.355094 2.674863
Cl 1.023400 -0.899871 -1.160162
Cl -3.335875 2.083742 -0.321465
Na -1.096266 1.207150 -1.706412
Na -3.000574 -0.566877 0.420397
Cl 4.848487 0.600632 -1.282971
Na 3.616297 -1.443380 -0.328390

A7v
Na -3.923790 -1.785729 0.427465
Cl -5.592737 -0.006312 1.279776
Na -3.955704 1.768166 0.358061
Cl -2.988090 3.830169 -0.871827
Na -0.753955 2.547209 -0.988776
Cl 1.810007 2.360240 -0.511818
Na 4.245929 1.603222 0.352929
Cl 6.015448 0.021854 -0.748136
Na 4.247139 -1.620731 0.263257
Cl 3.341660 -0.066080 2.277872
Na 1.168993 -0.007207 0.717844
Cl -1.609187 0.001808 0.002264
Cl 1.813699 -2.279080 -0.683470
Cl -2.962093 -3.850436 -0.803415
Na -0.763887 -2.523730 -1.039980

A7w
Na -3.136958 0.469995 0.000000
Cl -2.459052 1.081418 2.546414
Na -0.746964 2.887869 -1.694259
Cl 0.398751 1.053174 0.000000
Na 2.680352 -0.512816 0.000000
Cl 5.020104 -1.793806 0.000000
Na 2.921215 -3.387597 0.000000
Cl 1.615109 -2.186601 2.049855
Na -0.746964 -1.046565 1.799118
Cl -2.459052 1.081418 -2.546414
Cl -2.467807 -2.067810 0.000000
Na -0.746964 -1.046565 -1.799118
Cl 1.615109 -2.186601 -2.049855
Na -0.746964 2.887869 1.694259
Cl -0.924590 4.855627 0.000000

A7x
Na -3.217755 1.756186 -0.637788
Cl -0.914657 1.666640 0.733440
Na 1.753635 1.131101 1.150617
Cl 0.913654 -1.673513 0.722757
Na 0.000161 0.001999 -1.324525
Cl -2.559157 -0.733773 -1.615345
Cl -5.788812 1.553525 -0.777536
Na -5.041536 -0.915998 -0.264451
Cl -4.112146 -1.945659 1.930707
Na -1.755943 -1.140933 1.143281
Cl 2.557492 0.744042 -1.609558
Na 5.040955 0.923572 -0.261081
Cl 4.110311 1.926426 1.945640
Cl 5.792066 -1.541401 -0.789829
Na 3.222414 -1.750190 -0.641024

A7y
Na 2.583074 -1.770462 0.403013
Cl 2.333728 -1.433725 -2.247040
Na -0.300994 -1.964947 -1.405016
Cl -2.804270 -1.887283 -2.105712
Na -3.693134 -0.203313 -0.324421
Cl -2.623449 0.071811 2.327700
Na -0.134683 -0.878203 2.155020
Cl 2.607507 -0.207063 2.612684
Na 2.481894 1.886453 1.044804
Cl 0.166175 0.929223 -0.120220
Na 1.967256 1.180881 -2.188836
Cl 3.488338 2.989481 -1.067295
Cl 0.430100 -3.172490 0.910518
Cl -4.138816 2.419249 -0.645978
Na -2.067809 2.199005 0.833690

A7z
Na 0.000000 2.932253 -0.422809
Cl 0.000000 1.775672 1.980748
Cl -2.309821 1.957604 -1.223690
Na -1.887632 0.000000 -3.011950
Cl 0.000000 -0.000000 -4.798377
Na 1.887632 -0.000000 -3.011950
Cl 2.309821 -1.957604 -1.223690
Na -0.000000 -2.932253 -0.422809
Cl -2.309821 -1.957604 -1.223690
Cl 2.309821 1.957604 -1.223690
Cl -0.000000 -1.775672 1.980748
Na 1.799886 -0.000000 0.656960
Na -1.799886 0.000000 0.656960
Na -0.000000 0.000000 4.139768
Cl -0.000000 0.000000 6.647766

A7za
Cl 1.722101 1.511598 0.000000
Na -0.107982 0.655133 1.875271
Cl 1.784482 -1.135201 2.703302
Na 2.298307 -1.277768 -0.000000
Cl 1.784482 -1.135201 -2.703302
Na -0.107982 -2.865869 -2.196786
Cl 0.881293 -3.906838 -0.000000
Na -0.107982 -2.865869 2.196786
Cl -2.188732 -1.220226 2.313890
Na -3.033086 -0.300239 -0.000000
Cl -1.797122 2.045773 0.000000
Na -0.107982 0.655133 -1.875271
Cl -2.188732 -1.220226 -2.313890
Na 0.339143 3.909475 0.000000
Cl 0.537714 6.412677 0.000000

A7zb
Na -1.837998 -1.499777 0.000000
Cl -1.626254 -0.227157 2.455052
Na 1.035996 -0.177119 2.003390
Cl 3.464248 0.675638 2.552035
Na 3.368774 1.233714 0.000000
Cl 3.464248 0.675638 -2.552035
Na 1.035996 -0.177119 -2.003390
Cl -1.626254 -0.227157 -2.455052
Na -1.626254 2.272368 -1.626094
Cl 0.588257 1.886238 0.000000
Na -1.626254 2.272368 1.626094
Cl -3.062484 3.701857 0.000000
Cl 1.147233 -2.076880 0.000000
Cl -2.542701 -4.134982 0.000000
Na -0.050895 -4.346648 0.000000

A8a
Cl -2.611395 2.611395 1.503163
Na -0.000000 2.892855 1.173729
Cl -0.000000 3.693071 -1.503163
Na -2.045557 2.045557 -1.173729
Cl -2.611395 -2.611395 1.503163
Na -2.045557 -2.045557 -1.173729
Cl 0.000000 0.000000 0.000000
Na 2.045557 2.045557 -1.173729
Cl 2.611395 2.611395 1.503163
Na 2.892855 -0.000000 1.173729
Cl 3.693071 -0.000000 -1.503163
Na 2.045557 -2.045557 -1.173729
Cl 0.000000 -3.693071 -1.503163
Na -0.000000 -2.892855 1.173729
Cl 2.611395 -2.611395 1.503163
Cl -3.693071 0.000000 -1.503163
Na -2.892855 0.000000 1.173729

A8b
Cl 0.000000 0.000000 0.000000
Na 0.000000 2.635577 0.811298
Cl -0.656944 1.951707 3.348842
Na 1.546457 0.488941 2.738534
Cl -2.832670 -2.043042 1.006839
Na -0.000000 -2.635577 0.811298
Cl 2.043042 -2.832670 -1.006839
Na 2.635577 -0.000000 -0.811298
Cl 1.951707 0.656944 -3.348842
Na 0.488941 -1.546457 -2.738534
Cl -1.951707 -0.656944 -3.348842
Na -0.488941 1.546457 -2.738534
Cl -2.043042 2.832670 -1.006839
Na -2.635577 0.000000 -0.811298
Cl 2.832670 2.043042 1.006839
Cl 0.656944 -1.951707 3.348842
Na -1.546457 -0.488941 2.738534

A8c
Cl 1.558037 -0.767466 -0.695618
Na 4.236211 -1.080830 -1.981107
Cl 3.552329 -3.787989 -2.575805
Na 1.146284 -3.299728 -1.567206
Cl -1.475507 -2.861370 -1.289013
Na -1.156930 -0.105031 -0.946907
Cl -1.628215 -0.242130 1.856333
Na 0.110893 1.711644 1.538544
Cl 2.702902 1.744001 1.888778
Na 2.345208 1.931789 -0.993760
Cl 4.214061 1.413588 -2.773815
Na -0.397364 -2.449922 1.150853
Cl 1.924536 -3.778938 1.383068
Na 3.191694 -0.880302 1.479204
Cl -0.387816 2.509994 -0.949925
Cl 5.473885 -1.780536 0.430754
Na 4.188300 -3.984163 -0.029594

A8d
Cl -0.196774 1.564446 2.837653
Na -2.616207 0.313099 2.023762
Cl -2.510919 -1.729217 3.647716
Na -0.056552 -1.363417 2.825405
Cl 2.673618 -1.459422 2.654101
Na 2.290313 -1.120655 0.000000
Cl -0.457435 -1.025205 -0.000000
Na -2.616207 0.313099 -2.023762
Cl -3.081945 1.963330 -0.000000
Na -0.281793 1.784476 0.000000
Cl -0.196774 1.564446 -2.837653
Na 2.357195 1.154743 -2.567478
Cl 2.673618 -1.459422 -2.654101
Na 2.357195 1.154743 2.567478
Cl 2.716277 1.745592 0.000000
Cl -2.510919 -1.729217 -3.647716
Na -0.056552 -1.363417 -2.825405

A8e

Cl -0.239830 0.565265 0.000000
Na -1.345284 3.066963 0.000000
Cl 1.128942 3.992763 0.000000
Na 2.072770 1.919082 1.614598
Cl 2.072770 -0.453962 -2.869652
Na -0.217761 -1.392426 -2.031251
Cl 0.600205 -3.160684 0.000000
Na -2.066850 -3.434501 0.000000
Cl -2.835115 -1.969375 2.038038
Na -3.157632 -0.095205 0.000000
Cl -3.846734 2.466617 0.000000
Na 2.566143 -1.007975 0.000000
Cl 2.072770 -0.453962 2.869652
Na -0.217761 -1.392426 2.031251
Cl -2.835115 -1.969375 -2.038038
Cl 4.072084 1.252799 0.000000
Na 2.072770 1.919082 -1.614598

A8f
Cl 0.164770 0.451292 -0.189207
Na 0.658687 3.130363 -0.595254
Cl 3.233199 2.506305 -0.604139
Na 2.731302 0.047111 -1.702561
Cl 1.361342 -2.047493 -2.797065
Na -1.036356 -1.390244 -1.897280
Cl -1.149166 -2.927951 0.599826
Na -3.540559 -1.713276 0.455912
Cl -3.333450 0.314689 2.072538
Na -2.660688 1.588376 -0.287139
Cl -1.746913 3.992359 -0.895056
Na 1.461952 -2.476923 -0.075970
Cl 3.844559 -1.417574 0.269474
Na -0.770913 -0.507304 2.295832
Cl -3.544472 -0.534329 -1.895572
Na 2.800725 0.715855 1.518961
Cl 1.400387 0.054846 3.625229

A8g
Cl 0.092690 -0.166619 0.000000
Na 0.848106 -2.756886 1.583102
Cl 2.702029 -3.660836 0.000000
Na 0.848106 -2.756886 -1.583102
Cl -1.469839 -3.317557 0.000000
Na -1.963916 -1.090457 -1.657584
Cl -0.352023 3.291954 -2.071008
Na 1.276764 1.081367 -2.064315
Cl 3.016724 2.087819 -0.000000
Na 1.167323 3.924127 -0.000000
Cl -0.352023 3.291954 2.071008
Na 1.276764 1.081367 2.064315
Cl 0.056605 -1.071952 3.506367
Na -1.963916 -1.090457 1.657584
Cl 0.056605 -1.071952 -3.506367
Cl -3.628340 0.278335 0.000000
Na -1.678441 2.131508 -0.000000

A8h
Cl 0.066177 0.568836 -0.000000
Na 0.327350 1.991512 2.404504
Cl -2.015052 2.252510 3.391897
Na -2.501784 0.008029 2.117935
Cl -0.782526 -2.104803 2.652195
Na 1.640825 -1.250849 2.130319
Cl -0.782526 -2.104803 -2.652195
Na 1.640825 -1.250849 -2.130319
Cl 2.840588 1.162014 -2.618205
Na 3.124258 0.666534 -0.000000
Cl 2.840588 1.162014 2.618205
Na -1.247296 -2.232506 0.000000
Cl -3.702639 -1.092059 0.000000
Na 0.327350 1.991512 -2.404504
Cl 3.026489 -2.051838 -0.000000
Na -2.501784 0.008029 -2.117935
Cl -2.015052 2.252510 -3.391897

A8i
Cl 1.037954 -1.945412 -1.411962
Na 3.644672 -1.790468 -0.881635
Cl 3.568655 -1.492394 1.720395
Na 0.890331 -1.789310 1.383314
Cl -1.680033 -2.548668 1.494866
Na -2.140435 -0.099517 2.282427
Cl 0.408345 0.746766 2.312277
Na 2.929288 1.137783 0.901925
Cl 4.061744 0.618032 -1.694485
Na 1.361429 0.936864 -1.951083
Cl 1.629241 3.189575 -0.356252
Na -0.771769 2.439994 0.480115
Cl -3.398905 2.067054 1.379253
Na -1.779490 -1.790089 -1.179089
Cl -1.364928 0.994857 -1.789226
Cl -4.403665 -1.563339 -1.705621
Na -3.915201 0.852013 -0.957535

A8j
Cl -0.079683 0.613255 -0.004669
Na -0.380771 3.337491 0.014595
Cl -2.982143 2.884367 0.004183
Na -2.760890 0.609332 1.607740
Cl -1.574969 -1.454512 2.851311
Na 0.879805 -1.101636 2.025216
Cl 1.080748 -3.033129 0.001040
Na 3.520686 -1.919792 0.012858
Cl 3.446435 -0.270009 -2.027922
Na 2.767771 1.511111 0.007003
Cl 2.081987 4.074410 0.013848
Na -1.720704 -2.172790 -0.011057
Cl -4.150760 -0.958741 -0.020836
Na -2.729649 0.618586 -1.618159
Cl 3.428404 -0.267491 2.049643
Cl -1.551819 -1.448836 -2.869546
Na 0.898052 -1.105112 -2.029285

A8k
Cl -0.000000 -0.000000 1.502330
Na -0.000000 1.785101 3.590962
Cl -0.000000 3.598358 1.692404
Na -1.596012 1.926070 0.059777
Cl -3.258081 0.000000 -0.865244
Na -1.596012 -1.926070 0.059777
Cl 0.000000 -2.130339 -2.059047
Na 1.596012 -1.926070 0.059777
Cl 3.258081 -0.000000 -0.865244
Na -0.000000 -1.785101 3.590962
Cl -0.000000 -0.000000 5.492171
Na -1.541203 0.000000 -3.119823
Cl 0.000000 2.130339 -2.059047
Na 1.596012 1.926070 0.059777
Cl -0.000000 -3.598358 1.692404
Cl 0.000000 0.000000 -5.295155
Na 1.541203 -0.000000 -3.119823

A8l
Cl 1.920173 0.023878 1.606900
Na 2.009363 2.162011 -0.154811
Cl 0.007455 1.740956 -2.139258
Na -2.333832 0.123698 -1.885659
Cl -0.792190 -1.944099 -1.070830
Na -3.086769 -1.490524 0.584110
Cl -4.588879 -1.255508 -1.619237
Na 4.094841 -0.948897 0.386549
Cl 3.897126 0.809670 -1.553141
Na 1.568100 -0.785784 -1.600231
Cl 2.853229 -3.105475 -0.317866
Na 0.620572 -2.419961 1.173189
Cl -1.464016 -1.707470 2.784102
Na -0.653712 0.853097 2.089294
Cl 0.454890 3.576700 1.533862
Cl -2.882551 1.397897 0.611675
Na -1.299383 3.222603 -0.339305

A8m
Cl 1.409408 -1.455378 -1.822283
Na 0.000000 -3.658902 -0.759288
Cl -1.721696 -3.843508 -2.732766
Na -1.389747 -1.258527 -2.208268
Cl -1.409408 1.455378 -1.822283
Na -1.347908 1.043296 0.882903
Cl -1.731044 -1.627927 0.581457
Na -0.725957 -2.460626 2.871678
Cl 1.266504 -3.701831 1.690288
Na 1.347908 -1.043296 0.882903
Cl -0.000000 -0.000000 3.548106
Na 0.725957 2.460626 2.871678
Cl -1.266504 3.701831 1.690288
Na 1.389747 1.258527 -2.208268
Cl 1.731044 1.627927 0.581457
Cl 1.721696 3.843508 -2.732766
Na 0.000000 3.658902 -0.759288

A8n
Cl -1.503662 -0.000401 0.005505
Na -3.587160 1.787413 0.000878
Cl -1.689214 3.600680 -0.001944
Na -0.060510 1.928000 -1.596291
Cl 0.857950 -0.001653 -3.258270
Na -0.066107 -1.926063 -1.593021
Cl 2.058690 -2.134208 -0.003226
Na -0.055649 -1.926156 1.598605
Cl 0.873937 0.000898 3.256417
Na -3.597615 -1.787648 0.017872
Cl -5.490990 0.005849 0.008197
Na 3.113905 -0.004646 -1.547966
Cl 2.061456 2.130634 -0.003773
Na -0.056357 1.925138 1.594913
Cl -1.694752 -3.596749 0.010281
Cl 5.294633 -0.003510 -0.013827
Na 3.123275 0.000356 1.533860

A8o
Na 2.674381 -1.926556 0.357801
Cl 5.010940 -0.982724 -0.418252
Na 3.900057 1.256507 -1.092517
Cl 1.215755 0.417489 -0.719769
Na -0.635667 -1.480973 -1.646752
Cl 0.693191 -3.595359 -0.603443
Na -0.491709 -2.935396 1.612567
Cl 1.410093 -1.652312 2.876625
Na 0.187355 0.438844 1.807008
Cl -2.165689 -0.886025 0.935243
Na -4.182467 -0.514690 -1.131231
Cl -2.788411 -1.322209 -3.132939
Cl -5.400752 1.431236 0.112156
Na -3.104519 1.751196 1.229091
Cl -0.786004 2.954901 1.696094
Cl 3.276585 3.809032 -1.235342
Na 0.932838 3.142116 -0.379272

A8p
Cl 0.000000 0.000000 0.922739
Na 0.000000 1.796537 3.478232
Cl 0.000000 0.000000 5.316813
Na -0.000000 -3.002365 0.188760
Cl 2.061025 -2.674267 -1.587115
Na 1.796044 -0.000000 -1.387538
Cl -0.000000 -0.000000 -3.700093

Na 0.000000 2.589972 -3.209572
Cl -2.061025 2.674267 -1.587115
Na 0.000000 3.002365 0.188760
Cl 0.000000 4.163266 2.506342
Na -0.000000 -2.589972 -3.209572
Cl -2.061025 -2.674267 -1.587115
Na -1.796044 0.000000 -1.387538
Cl 2.061025 2.674267 -1.587115
Na -0.000000 -1.796537 3.478232
Cl -0.000000 -4.163266 2.506342

A8q
Cl -1.298012 2.579153 0.000000
Na -2.918647 0.471957 0.000000
Cl -3.202354 -0.204454 2.538708
Na -0.600395 0.994896 2.478942
Cl 1.946209 1.916012 2.398025
Na 2.296999 -0.765514 1.918210
Cl -0.349229 -1.115540 0.000000
Na -1.396873 -2.104659 -2.315977
Cl -3.202354 -0.204454 -2.538708
Na -0.600395 0.994896 -2.478942
Cl 0.504280 -1.368282 -3.856151
Na 2.296999 -0.765514 -1.918210
Cl 3.761638 -1.782927 -0.000000
Na 1.375426 2.711044 -0.000000
Cl 1.946209 1.916012 -2.398025
Cl 0.504280 -1.368282 3.856151
Na -1.396873 -2.104659 2.315977

A8s
Cl 1.244070 0.645475 0.033909
Na 2.940864 2.771093 -0.184334
Cl 0.987889 4.518092 0.188026
Na -0.684801 2.476719 0.554993
Cl -1.722643 0.857675 2.540387
Na 0.275392 -0.913008 2.020856
Cl -1.323446 -2.518645 0.260489
Na 1.110132 -3.592461 -0.063865
Cl 1.934410 -2.439863 -2.261466
Na 3.452099 -0.929279 -0.314601
Cl 5.034380 1.215480 -0.547680
Na -3.171945 -0.521116 0.759898
Cl -2.213853 1.089757 -1.596935
Na -0.189152 -0.839553 -1.664292
Cl 2.495476 -2.530222 1.871786
Cl -5.770620 -0.450909 -0.017806
Na -4.761340 0.949763 -1.836115

A8t
Na 3.510383 1.070180 0.592431
Cl 5.214262 -0.998881 0.864456
Na 3.107082 -2.440173 0.380579
Cl 1.368720 -0.563642 1.241757
Na -0.366187 1.464116 1.555050
Cl 1.677449 3.269948 1.181683
Na 1.161777 2.861252 -1.339773
Cl 2.993995 1.211097 -2.171691
Na 1.114245 -0.604885 -1.633954
Cl -0.968066 1.204836 -1.328944
Na -4.404756 -0.492692 1.194628
Cl -2.179654 -0.262025 2.585736
Cl -3.376102 -2.145505 -0.691989
Na -0.993463 -1.922248 0.588165
Cl 0.996214 -3.226818 -1.119796
Cl -5.492199 1.337729 -0.343301
Na -3.491673 0.332215 -1.673899

A8u
Cl -1.449246 0.623034 -1.130940
Na -2.443817 3.023900 -0.354409
Cl -0.425050 4.495450 -1.043743
Na 0.843718 2.147821 -1.294804
Cl 1.446781 1.272246 1.374950
Na 3.036817 -0.922882 -0.297430
Cl 1.453924 -3.116811 -0.361049
Na -0.030150 -1.533128 -2.052333
Cl -2.493020 -2.940123 -1.539024
Na -0.962858 -3.449297 0.576575
Cl -2.092077 -1.897439 2.360219
Na -3.371345 -0.915701 0.117538
Cl -3.734202 1.603192 1.473914
Na -1.158253 0.541704 1.742274
Cl 2.100013 0.153385 -2.599050
Na 3.868146 1.340854 2.190383
Cl 5.333770 -0.343875 1.058503

6.5 Structures Neutral Clusters: B3LYP-D3/aug-cc-pVDZ

N1a
Na 0.000000 0.000000 -1.450473
Cl 0.000000 0.000000 0.938541

N2a
Na 0.000000 1.604474 0.000000
Cl -0.000000 -0.000000 2.002027
Na -0.000000 -1.604474 0.000000
Cl 0.000000 -0.000000 -2.002027

N3a
Na 0.000000 2.319280 -0.000000
Cl 2.358237 1.361529 -0.000000
Na 2.008556 -1.159640 0.000000
Cl -2.358237 1.361529 -0.000000
Na -2.008556 -1.159640 0.000000
Cl -0.000000 -2.723058 -0.000000

N3b
Na 0.000000 1.473273 0.676710
Cl 1.830544 -0.000000 -0.942481
Na -0.000000 -1.473273 0.676710
Cl -1.830544 0.000000 -0.942481
Na 0.000000 -0.000000 -2.718573
Cl -0.000000 0.000000 2.768297

N4a
Na 1.241580 1.241580 1.241580
Cl 1.427410 -1.427410 1.427410
Na -1.241580 -1.241580 1.241580
Cl -1.427410 1.427410 1.427410
Na -1.241580 1.241580 -1.241580
Cl -1.427410 -1.427410 -1.427410
Cl 1.427410 1.427410 -1.427410
Na 1.241580 -1.241580 -1.241580

N4b
Cl 2.466692 2.466692 -0.000000
Na 0.000000 3.038193 0.000000
Cl -2.466692 2.466692 -0.000000
Na -3.038193 0.000000 0.000000
Cl -2.466692 -2.466692 -0.000000
Na -0.000000 -3.038193 0.000000
Cl 2.466692 -2.466692 -0.000000
Na 3.038193 -0.000000 0.000000

N4c
Na -0.126338 -1.833101 1.495014
Cl 0.607600 -3.778214 0.000000
Na -0.126338 -1.833101 -1.495014
Cl -2.179699 -0.785166 0.000000
Na -1.262475 1.643979 0.000000
Cl 1.358731 0.184866 0.000000
Cl -0.126338 3.962466 0.000000
Na 2.040150 2.665207 0.000000

N4d
Cl -0.064560 -1.791912 1.823009
Na 1.014984 0.157832 0.000000
Cl 2.254388 2.420831 0.000000
Na -0.064560 3.416418 0.000000
Cl -1.915114 1.665052 0.000000
Na -1.917574 -0.906053 0.000000
Cl -0.064560 -1.791912 -1.823009
Na 0.642368 -3.444107 0.000000

N4e
Na 0.000000 1.656797 0.376710
Cl 0.000000 2.289103 -2.095935
Na 0.000000 0.000000 -3.199002
Cl -0.000000 -2.289103 -2.095935
Na -0.000000 -1.656797 0.376710
Cl -1.803459 0.000000 1.734948
Cl 1.803459 -0.000000 1.734948
Na 0.000000 -0.000000 3.561361

N4f
Cl 0.520052 -1.988336 2.308006
Na -0.498814 -2.522633 0.000000
Cl 0.520052 -1.988336 -2.308006
Na 0.520052 0.475603 -1.738906
Cl -1.787678 0.519131 -0.000000
Na 0.520052 0.475603 1.738906
Cl 1.239503 2.515082 -0.000000
Na -1.301547 3.027955 -0.000000

N4g
Cl 0.000000 -0.000000 -1.394634
Na 0.000000 2.440559 -2.204412
Cl 0.000000 4.001374 -0.246489
Na 0.000000 1.982973 1.372750
Cl 0.000000 0.000000 2.963879
Na -0.000000 -1.982973 1.372750
Cl -0.000000 -4.001374 -0.246489
Na -0.000000 -2.440559 -2.204412

N4h
Na -0.000000 0.000000 -1.220119
Cl -0.000000 2.523214 -1.855947
Na 1.683599 3.009378 -0.070594
Cl 2.019302 0.976253 1.450204
Na 0.000000 -0.000000 2.615422
Cl -2.019302 -0.976253 1.450204
Na -1.683599 -3.009378 -0.070594
Cl 0.000000 -2.523214 -1.855947

N5a
Na -0.784148 1.789395 1.786713
Cl 1.256354 1.453528 0.000000
Na 1.740540 -0.951859 -1.684511
Cl -0.784148 -0.779445 -2.431546
Na -0.784148 1.789395 -1.786713
Cl -2.703096 1.861674 -0.000000
Na -1.965453 -0.782644 -0.000000
Cl -0.784148 -0.779445 2.431546
Na 1.740540 -0.951859 1.684511
Cl 3.049118 -2.333764 0.000000

N5b
Na 0.796130 1.328002 1.701888
Cl -1.041458 2.341329 0.000000
Na 0.796130 1.328002 -1.701888
Cl 0.796130 -1.307190 -1.962713
Na -0.920452 -2.028329 0.000000
Cl 0.796130 -1.307190 1.962713
Na 2.608059 -1.109521 0.000000
Cl 2.852998 1.529376 0.000000
Cl -3.463795 -1.549586 0.000000
Na -3.187150 0.935069 0.000000

N5c
Na 0.000000 0.000000 -1.545671
Cl 0.000000 2.760424 -0.810618
Na -1.862445 -1.838417 0.740221
Cl 0.000000 0.000000 1.970172
Na 1.862445 1.838417 0.740221
Cl 3.019319 -0.000000 -0.632331
Na 1.862445 -1.838417 0.740221
Cl -3.019319 0.000000 -0.632331
Na -1.862445 1.838417 0.740221
Cl -0.000000 -2.760424 -0.810618

N5d
Na -0.870116 -0.939423 2.454120
Cl -1.720947 -0.610013 -0.000000
Na -0.870116 -0.939423 -2.454120
Cl 0.321592 -1.745146 -4.607638
Na 1.353748 0.555003 -4.576126
Cl 0.321592 1.746485 -2.545147
Na -0.295643 1.707450 0.000000
Cl 0.321592 1.746485 2.545147
Na 1.353748 0.555003 4.576126
Cl 0.321592 -1.745146 4.607638

N5e
Na -2.471576 -0.787350 1.356884
Cl -0.497559 -0.885537 -0.524251
Na 2.101145 -1.183131 -0.528368
Cl 2.571943 1.729520 -0.330453
Na -0.026874 1.773140 -0.071481
Cl -2.543001 1.976646 0.568569
Na -3.583750 0.345265 -1.251962
Cl -4.559622 -1.577793 0.087678
Na 4.805385 0.702051 0.433737
Cl 4.494849 -1.792820 0.238050

N5f
Cl 2.501296 3.442739 0.000000
Na 0.000000 3.813479 -0.000000
Cl -2.501296 3.442739 -0.000000
Na -3.626834 1.178430 -0.000000
Cl -4.047182 -1.315009 0.000000
Na -2.241507 -3.085170 -0.000000
Cl -0.000000 -4.255459 0.000000
Na 2.241507 -3.085170 -0.000000
Cl 4.047182 -1.315009 0.000000
Na 3.626834 1.178430 -0.000000

N6a
Cl 0.000000 -0.000000 2.165804
Na 0.000000 1.960578 -0.000000
Cl -2.713484 2.030330 -0.000000
Na -2.615664 0.000000 -1.721877
Cl 0.000000 -0.000000 -2.165804
Na 2.615664 -0.000000 -1.721877
Cl 2.713484 2.030330 0.000000
Na 2.615664 0.000000 1.721877
Cl 2.713484 -2.030330 -0.000000
Na -0.000000 -1.960578 -0.000000
Cl -2.713484 -2.030330 -0.000000
Na -2.615664 -0.000000 1.721877

N6b
Na 0.000000 2.474170 1.262056
Cl 0.000000 2.742634 -1.457361
Na 2.142694 1.237085 -1.262056
Cl 2.375191 1.371317 1.457361
Na 2.142694 -1.237085 1.262056
Cl -0.000000 -2.742634 1.457361
Na -2.142694 -1.237085 1.262056
Cl -2.375191 1.371317 1.457361
Na -2.142694 1.237085 -1.262056
Cl -2.375191 -1.371317 -1.457361
Na -0.000000 -2.474170 -1.262056
Cl 2.375191 -1.371317 -1.457361

N6d
Na 1.223268 1.640726 0.707232
Cl -0.749260 2.938157 -0.424444
Na -2.534201 1.710596 0.959993
Cl -0.703631 -0.110834 1.970776
Cl -3.578423 -0.099968 -0.642400
Na -2.359853 -1.977967 0.720165
Na -1.024668 0.116274 -1.666424
Cl -0.426028 -2.899361 -0.707192
Na 1.286424 -1.644795 0.797836
Cl 3.392115 -0.021231 1.404161
Na 4.043421 0.031629 -1.083139
Cl 1.654739 0.273173 -1.882801

N6e
Na -0.056066 1.373067 1.684285
Cl 1.937172 2.058300 0.000000
Na 1.826880 -1.617346 0.000000
Cl -0.056066 -1.216764 1.920242
Na -1.837728 -1.878394 0.000000
Cl -4.383689 -1.407303 0.000000
Na -4.100847 1.074710 0.000000
Cl -1.899349 2.392307 0.000000
Na -0.056066 1.373067 -1.684285
Cl -0.056066 -1.216764 -1.920242
Cl 4.419434 -1.476891 0.000000
Na 4.283426 1.014983 0.000000

N6f
Cl 0.352272 1.387783 0.000000
Na -1.611232 0.741148 1.770089
Cl -3.582704 1.048152 0.000000
Na -1.611232 0.741148 -1.770089
Cl -1.611232 -1.856759 -1.980041
Na -3.375987 -1.656218 0.000000
Cl -1.611232 -1.856759 1.980041
Na 0.258931 -2.225259 0.000000
Cl 2.823858 -2.231849 0.000000
Na 3.495563 0.200459 0.000000
Cl 4.251337 2.686843 0.000000
Na 1.882220 3.469997 0.000000

N6g
Cl 1.446823 -1.363292 -0.025747
Na 1.283756 1.253277 -0.022685
Cl 1.738437 3.832807 -0.009383
Na -1.181158 3.971478 -0.003600
Cl -1.446823 1.363292 -0.025747
Na -1.283756 -1.253277 -0.022685
Cl -1.738437 -3.832807 -0.009383
Na -1.256866 -6.360568 0.029348
Cl 1.256866 -6.560565 0.033148
Na 1.181158 -3.971478 -0.003600
Cl -1.256866 6.560565 0.033148
Na 1.256866 6.360568 0.029348

N6h
Cl 1.420057 3.822238 -0.754428
Na 2.944637 1.951024 0.117505
Cl 4.507319 0.002376 0.611608
Na 2.949430 -1.949568 0.115645
Cl 1.424268 -3.820679 -0.755111
Na -0.699683 -2.461208 -0.551280
Cl -3.208093 -2.001521 0.294728
Na -4.309789 -0.002454 -0.878157
Cl -3.210788 1.998885 0.293437
Na -0.702404 2.459940 -0.553636
Cl 0.256379 0.000274 0.349682
Na -2.019957 -0.000163 1.688237

N6i
Cl 2.080986 0.137872 0.000000
Na 4.366491 -0.994310 0.000000
Cl 5.549572 -3.227461 0.000000
Na 3.516022 -4.770020 -0.000000
Cl 1.034108 -4.356146 -0.000000
Na 0.212955 -1.922854 -0.000000
Cl -1.815633 -0.209186 -0.000000
Na -3.664343 1.638432 -0.000000
Cl -1.591708 3.833845 0.000000
Na -0.000000 1.766802 0.000000
Cl -5.638949 3.307676 -0.000000
Na -3.841343 5.075387 0.000000

N6j
Cl 0.000000 0.000000 -0.077569
Na 0.000000 2.288441 -1.397734
Cl 0.940184 4.295083 -2.727365
Na 0.065778 5.603977 -0.752968
Cl -1.064669 3.804910 0.718271
Na -0.722589 1.681118 2.223533
Cl 1.064669 -3.804910 0.718271
Na -0.065778 -5.603977 -0.752968
Cl -0.940184 -4.295083 -2.727365
Na -0.000000 -2.288441 -1.397734
Na 0.722589 -1.681118 2.223533
Cl -0.000000 -0.000000 4.001505

N6k
Cl -2.063616 -0.407715 -0.000000
Na -0.298807 -2.374994 -0.000000
Cl 0.878024 -4.638124 -0.000000
Na 3.282400 -3.825599 -0.000000
Cl 1.903841 -0.515854 -0.000000
Na -0.000000 1.299739 0.000000
Cl -1.257674 3.578755 0.000000
Na -3.317203 5.119928 0.000000
Cl -5.343282 3.620444 0.000000
Na -3.639142 1.682417 0.000000
Na 4.465848 -0.371494 -0.000000
Cl 5.563645 -2.627505 -0.000000

N6l
Na -3.307463 -0.791744 1.529115
Cl -5.545997 -1.644462 0.600495
Na -4.821823 0.285944 -0.883894
Cl -3.554739 1.949412 0.763582
Na -1.169147 1.811283 -0.270159
Cl 1.307865 1.733758 -1.092340
Na 3.704844 1.111316 -0.227826
Cl 3.348008 -1.724899 -0.810741
Na 0.882181 -0.972503 -1.249694
Cl -1.663580 -0.877446 -0.652797
Cl 5.714787 0.732562 1.358340
Na 5.319788 -1.705362 0.845081

N6m

Na 0.000000 1.894514 -0.000000
Cl 0.555569 4.400731 -0.000000
Na 3.043562 4.817093 -0.000000
Cl 5.173227 3.411559 -0.000000
Na 4.217843 1.069842 -0.000000
Cl 1.920285 -0.031418 -0.000000
Na -0.000423 -1.897055 0.000000
Cl -5.172873 -3.409570 0.000000
Na -4.221008 -1.066664 0.000000
Cl -1.920065 0.029353 0.000000
Na -3.043107 -4.813559 0.000000
Cl -0.554116 -4.403353 0.000000

N6n
Cl -2.251552 -0.114104 0.000000
Na -4.232643 1.503184 0.000000
Cl -4.576306 4.006973 0.000000
Na -2.201952 4.936276 0.000000
Cl 0.131267 3.960091 0.000000
Na 0.000000 1.399477 0.000000
Cl 1.596890 -0.716729 0.000000
Na 2.682798 -4.017102 0.000000
Cl 0.216558 -4.669333 0.000000
Na -0.693784 -2.287803 0.000000
Cl 5.063087 -3.023518 0.000000
Na 4.167486 -0.673801 0.000000

N6o
Cl 2.514890 4.355918 -0.000000
Na -0.000000 4.608656 -0.000000
Cl -2.514890 4.355918 -0.000000
Na -3.991214 2.304328 -0.000000
Cl -5.029781 0.000000 -0.000000
Na -3.991214 -2.304328 -0.000000
Cl -2.514890 -4.355918 -0.000000
Na -0.000000 -4.608656 -0.000000
Cl 2.514890 -4.355918 -0.000000
Na 3.991214 -2.304328 -0.000000
Cl 5.029781 -0.000000 -0.000000
Na 3.991214 2.304328 -0.000000

N7a
Na 0.000000 2.344056 -1.277277
Cl -1.996063 1.152427 -2.588535
Na -2.030012 -1.172028 -1.277277
Cl -2.312645 -1.335206 1.292262
Na -1.746910 1.008579 2.435779
Cl 0.000000 2.670412 1.292262
Na 1.746910 1.008579 2.435779
Cl 0.000000 0.000000 4.180324
Na -0.000000 -2.017158 2.435779
Cl -0.000000 -2.304855 -2.588535
Na 0.000000 0.000000 -3.926015
Cl 1.996063 1.152427 -2.588535
Na 2.030012 -1.172028 -1.277277
Cl 2.312645 -1.335206 1.292262

N7b
Cl -2.221730 -1.546164 -0.000000
Na -0.663254 -3.762922 -0.000000
Cl 0.934890 -3.082105 2.019879
Na -0.517514 -0.853763 2.088877
Cl -1.354126 1.635211 2.472795
Na -2.270657 1.213996 0.000000
Cl -1.354126 1.635211 -2.472795
Na 0.934890 2.741832 -1.732318
Cl 1.332071 0.456415 0.000000
Na 2.320968 -2.019037 -0.000000
Cl 0.934890 -3.082105 -2.019879
Na -0.517514 -0.853763 -2.088877
Na 0.934890 2.741832 1.732318
Cl 1.584609 4.495894 0.000000

N7c
Cl 1.714224 0.202016 -0.000000
Na 1.634160 2.280162 1.866045
Cl -0.797764 1.778394 2.672841
Na -0.797764 -0.764094 2.126718
Cl 0.268048 -3.203612 2.020098
Na 1.825715 -2.451452 0.000000
Cl 0.268048 -3.203612 -2.020098
Na -1.461978 -3.484697 0.000000
Cl -2.409744 -0.970365 0.000000
Na -1.196695 2.273157 -0.000000
Cl 1.211532 4.026984 -0.000000
Na 1.634160 2.280162 -1.866045
Cl -0.797764 1.778394 -2.672841
Na -0.797764 -0.764094 -2.126718

N7d
Cl 0.326600 0.207322 1.993762
Na 0.326600 -2.428599 1.680470
Cl 2.375582 -2.676925 0.000000
Na 2.430630 -0.043755 0.000000
Cl 3.125376 2.501492 0.000000
Na 1.105435 2.803590 1.728858
Cl -0.702521 3.616633 0.000000
Na 1.105435 2.803590 -1.728858
Cl 0.326600 0.207322 -1.993762
Na 0.326600 -2.428599 -1.680470
Cl -1.423417 -3.595260 0.000000
Na -1.620899 1.063373 0.000000
Cl -4.042251 0.109245 0.000000
Na -3.652121 -2.341155 0.000000

N7e
Na 1.074493 -3.136892 0.000000
Cl -0.602863 -3.091488 2.034363
Na -2.339320 -3.029431 0.000000
Cl -0.602863 -3.091488 -2.034363
Na -0.602863 -0.350108 -1.937313
Cl -2.775178 -0.437542 0.000000
Na -0.602863 -0.350108 1.937313
Cl 1.658242 -0.542541 0.000000
Na 3.887732 0.749663 0.000000
Cl 3.644382 3.223026 0.000000
Na 1.056757 2.874946 0.000000
Cl -0.682936 2.311837 -2.004346
Na -2.405703 2.185394 0.000000
Cl -0.682936 2.311837 2.004346

N7f
Cl 1.269635 0.750943 2.172102
Na -1.354074 0.384612 1.954604
Cl -1.354074 -2.217142 2.310396
Na -1.047894 -3.425161 0.000000
Cl 1.686647 -3.438472 0.000000
Na 1.340347 -1.864223 2.076825
Na 1.340347 -1.864223 -2.076825
Cl 1.269635 0.750943 -2.172102
Na 1.913479 2.129916 -0.000000
Cl -1.354074 -2.217142 -2.310396
Na -1.354074 0.384612 -1.954604
Cl -2.459153 1.826540 0.000000
Cl 1.199864 4.635788 -0.000000
Na -1.237599 4.113124 -0.000000

N7g
Na -1.923501 -1.493241 -1.890182
Cl -2.566478 1.045468 -2.238531
Na -3.488451 0.772449 0.329359
Cl -1.855708 2.534727 1.401593
Na 0.394777 1.369582 2.100183
Cl 1.043741 -1.112162 2.424879
Na 2.317676 -1.344341 -0.031596
Cl 1.704276 2.100463 -0.195117
Na -0.754556 2.512911 -1.072117
Cl -3.280892 -1.825168 0.470226
Na -0.872595 -2.506838 1.286168
Cl 0.277838 -2.658564 -1.141516
Na 4.181665 1.685815 -0.767401
Cl 4.771037 -0.729454 -0.692037

N7h
Cl 1.555366 -2.461933 0.000000
Na 0.882855 -1.693857 2.473729
Cl -1.757990 -2.106827 2.383351
Na -1.196116 -3.004217 0.000000
Cl -1.757990 -2.106827 -2.383351
Na 0.882855 -1.693857 -2.473729
Cl 0.850199 0.898108 -2.282736
Na -1.757990 0.545460 -1.996989
Cl -2.279807 2.186066 -0.000000
Na -1.757990 0.545460 1.996989
Cl 0.850199 0.898108 2.282736
Na 2.370407 0.891142 -0.000000
Cl 2.764870 3.456616 -0.000000
Na 0.228491 3.230208 -0.000000

N7i
Na -1.434224 -2.742509 0.000000
Cl -0.603297 -2.038756 2.394253
Na -2.380357 -0.049557 2.104748
Cl -3.617714 -1.010680 0.000000
Na -2.380357 -0.049557 -2.104748
Cl -0.603297 -2.038756 -2.394253
Na 1.192431 -0.145425 -1.868592
Cl -0.603297 1.876387 -2.233065
Na 0.141792 3.008450 0.000000
Cl -0.603297 1.876387 2.233065
Na 1.192431 -0.145425 1.868592
Cl 2.799160 -1.348012 -0.000000
Na 4.469183 0.581999 -0.000000
Cl 2.713514 2.387092 -0.000000

N7j
Na -1.319602 2.626174 0.941215
Cl -3.340004 0.928711 1.557921
Na -0.352492 0.473491 -1.920695
Cl -2.213738 -1.457007 -1.500240
Na 0.112338 -2.939683 -1.193655
Cl 1.756195 -1.018290 -1.892551
Na 2.531811 -1.100697 0.897168
Cl 0.455786 -3.025868 1.385351
Na -1.252625 -1.023726 1.732341
Cl -0.156896 3.028179 -1.414598
Na 2.334697 1.948271 -0.376011
Cl 0.538664 0.880833 1.522323
Cl 4.346673 0.756960 0.667575
Na -4.197179 -0.128357 -0.583842

N7k
Na 0.000000 0.000000 -0.554080
Cl 2.111908 1.219310 -1.782304
Na 0.000000 2.849389 -1.729384
Cl 0.000000 2.885709 0.894719
Na 1.970250 1.137524 1.675961
Cl 2.499098 -1.442855 0.894719
Na 2.467643 -1.424694 -1.729384
Cl -0.000000 -2.438621 -1.782304
Na -2.467643 -1.424694 -1.729384
Cl -2.111908 1.219310 -1.782304
Cl -2.499098 -1.442855 0.894719
Na -0.000000 -2.275049 1.675961
Cl 0.000000 0.000000 3.124982
Na -1.970250 1.137524 1.675961

N7l
Cl 0.000000 0.000000 1.601667
Na 0.000000 2.606592 1.317440
Cl 0.000000 5.200902 1.672887
Na 0.000000 5.223980 -1.260490
Cl 0.000000 2.606716 -1.411111
Na -0.000000 -0.000000 -1.150966
Cl -0.000000 -2.606716 -1.411111
Na -0.000000 -5.223980 -1.260490
Cl 0.000000 -7.807828 -1.431292
Na 0.000000 -7.706298 1.088316
Cl -0.000000 -5.200902 1.672887
Na -0.000000 -2.606592 1.317440
Cl 0.000000 7.807828 -1.431292
Na 0.000000 7.706298 1.088316

N7m
Cl 1.084575 0.306330 -0.503701
Na 3.043240 -1.444391 -0.280750
Cl 4.618766 -3.494157 -0.314675
Na 6.290322 -1.812814 0.559789
Cl 4.927387 0.378011 0.709065
Na -0.465903 2.984154 -0.378193
Cl -2.630713 1.760442 -1.181940
Na -4.720274 0.442748 -0.282278
Cl -6.567369 -0.499579 1.261383
Na -5.293503 -2.666154 1.047276
Cl -3.344639 -2.152771 -0.554676
Na -1.323146 -0.591785 -1.064124
Na 3.041019 2.206536 0.516335
Cl 1.542034 4.272240 0.508155

N8a
Cl 0.000000 2.126810 1.316061
Na 0.000000 1.919225 -1.368002
Cl -2.126810 0.000000 -1.316061
Na -1.919225 0.000000 1.368002
Cl -2.021672 0.000000 4.070993
Na 0.000000 1.732443 3.951308
Cl 2.021672 0.000000 4.070993
Na -0.000000 -1.732443 3.951308
Cl -0.000000 -2.126810 1.316061
Na -0.000000 -1.919225 -1.368002
Cl 2.126810 -0.000000 -1.316061
Na 1.919225 -0.000000 1.368002
Cl -0.000000 -2.021672 -4.070993
Na 1.732443 -0.000000 -3.951308
Cl 0.000000 2.021672 -4.070993
Na -1.732443 0.000000 -3.951308

N8b
Na 1.636036 -1.059827 1.912255
Cl 0.006210 -1.471289 4.014273
Na -0.602552 1.126055 3.820754
Cl -0.482606 -1.763374 0.000000
Na 1.636036 -1.059827 -1.912255
Cl 3.502189 -0.860440 -0.000000
Na 2.909205 1.717902 -0.000000
Cl 1.177752 1.790688 1.978301
Na -1.097867 1.421120 0.000000
Cl -2.686216 0.949650 -2.213047
Na -1.950802 -1.569503 -2.225659
Cl 0.006210 -1.471289 -4.014273
Na -0.602552 1.126055 -3.820754
Cl 1.177752 1.790688 -1.978301
Cl -2.686216 0.949650 2.213047
Na -1.950802 -1.569503 2.225659

N8c
Cl -2.961053 0.643841 -1.214341
Na 1.022628 -2.510142 -1.282920
Cl 0.117331 -1.993182 -3.685423
Na -1.760553 -0.177457 -3.400327
Cl -0.117331 1.993182 -3.685423
Na -1.022628 2.510142 -1.282920
Cl 0.081983 2.171975 1.057264
Na 2.011674 0.240050 1.083237
Cl 2.961053 -0.643841 -1.214341
Na 1.760553 0.177457 -3.400327
Na -2.011674 -0.240050 1.083237
Cl -0.081983 -2.171975 1.057264
Na 0.081983 -1.722014 3.680466
Cl -2.016707 -0.080896 3.790439
Na -0.081983 1.722014 3.680466
Cl 2.016707 0.080896 3.790439

N8d
Cl -1.000546 3.142318 2.023580
Na -1.982945 1.596499 -0.000000
Cl -1.000546 3.142318 -2.023580
Na 0.398797 4.039681 -0.000000
Cl 1.988919 1.850521 -0.000000
Na 3.064482 -0.575341 0.000000
Cl 2.204338 -1.238717 2.407127
Na 0.571959 0.994622 2.305909
Cl -1.772341 -0.649456 1.942396
Na -2.698308 -2.159714 0.000000
Cl -1.772341 -0.649456 -1.942396
Na 0.571959 0.994622 -2.305909
Cl 2.204338 -1.238717 -2.407127
Na 0.061249 -2.649201 2.016797
Na 0.061249 -2.649201 -2.016797
Cl -0.883165 -4.094791 0.000000

N8e
Cl -1.042410 3.283798 0.687185
Na -2.475773 1.887798 -0.962128
Cl -4.093421 -0.203980 -0.901347
Na -0.565406 1.584599 2.664690
Cl 1.863129 0.891650 1.836242
Na 1.178364 2.210281 -0.543144
Cl -0.456058 0.742402 -2.441270
Na -2.159353 -1.271697 -2.373724
Cl -0.876291 -2.646559 -0.571103
Na -2.855851 -1.617312 0.979681
Cl -1.428337 -0.879340 3.020293
Na 0.750069 -1.720087 1.512527
Cl 2.962327 -2.384797 0.126554
Na 1.694813 -1.016099 -1.811660
Na 4.029986 -0.014871 0.502357
Cl 3.331924 1.169255 -1.736236

N8f
Na 1.927147 1.571274 0.346501
Cl 4.306081 0.261208 0.333984
Na 2.845709 -1.490543 1.637664
Cl 0.817358 -0.024886 2.511555
Na -1.210080 1.816603 2.273497
Cl -0.055159 3.391237 0.519693
Na -3.801213 -0.514129 -0.303336
Cl -2.338931 -2.716535 -0.502588
Na -1.071543 -1.788056 1.598030
Cl -3.094634 0.049894 2.177700
Cl 1.245067 0.844378 -2.324222
Na -0.625830 -1.234251 -1.925216
Cl -2.661840 0.718597 -2.316854
Na -0.862818 2.495138 -1.823302
Na 3.180000 -0.877097 -1.757218
Cl 1.535288 -2.510266 -0.429434

N8g
Cl -0.000000 0.000000 0.972928
Na -0.000000 1.866227 3.196274
Cl -0.000000 0.000000 4.948624
Na -0.000000 -1.866227 3.196274
Cl -0.000000 -3.582425 1.172735
Na -1.633307 -1.973517 -0.286049
Cl 0.000000 -1.976153 -2.693153
Na 1.742837 -0.000000 -3.162971
Cl 3.302466 -0.000000 -1.091725
Na 1.633307 -1.973517 -0.286049
Na -1.633307 1.973517 -0.286049
Cl 0.000000 1.976153 -2.693153
Na -1.742837 0.000000 -3.162971
Cl -3.302466 0.000000 -1.091725
Cl 0.000000 3.582425 1.172735
Na 1.633307 1.973517 -0.286049

N8h
Cl -0.284758 0.711548 0.000000
Na -2.447077 1.326428 1.737253
Cl -2.744134 3.323526 0.000000
Na -2.447077 1.326428 -1.737253
Cl -2.447077 -1.179088 -2.419740
Na 0.283015 -1.311054 -2.029132
Cl 2.878013 -0.909282 -2.013274
Na 2.876505 1.009616 0.000000
Cl 2.878013 -0.909282 2.013274
Na 0.283015 -1.311054 2.029132
Cl -2.447077 -1.179088 2.419740
Na -2.302323 -2.319015 0.000000
Cl 0.217298 -3.242074 0.000000
Na 2.880582 -2.602831 0.000000
Na -0.001110 3.549720 0.000000
Cl 2.515556 3.598409 0.000000

N8i
Na -1.477214 -2.325128 -0.764595
Cl -3.971488 -2.655390 -0.289909
Na -3.722132 -0.212948 0.427338
Cl -1.457922 0.004495 -2.255893
Na 1.119828 0.062750 -1.810785
Cl 1.315732 -2.439608 -0.485383
Na 1.111664 -1.443506 2.020539
Cl -1.329612 -0.637032 2.086428
Na -1.085876 1.924446 1.778231
Cl -3.361076 2.476126 0.547227
Na -1.711618 2.473191 -1.481630
Cl 0.711132 2.578772 -0.075560
Na 3.198857 1.810842 0.447186
Cl 3.488130 -0.295430 2.062980
Cl 3.755567 0.522733 -1.811533
Na 3.879413 -1.601406 -0.273745

N8j
Na -3.348775 1.866841 0.000000
Cl -3.725485 0.147208 1.994928
Na -3.923759 -1.618668 0.000000
Cl -3.725485 0.147208 -1.994928
Na -1.151992 -0.529598 -1.975028
Cl 1.439821 -0.369613 -2.212899
Na 1.439821 2.163376 -1.680691
Cl -0.716135 2.223583 0.000000
Na 1.439821 2.163376 1.680691
Cl 1.439821 -0.369613 2.212899
Na -1.151992 -0.529598 1.975028
Cl -1.342235 -2.416291 0.000000
Na 1.489843 -2.300485 0.000000
Cl 3.417863 2.558287 0.000000
Na 3.920667 -0.103270 0.000000
Cl 4.044190 -2.640282 0.000000

N8k
Na -2.390311 -0.549942 -1.843983
Cl -3.127622 2.008404 -1.144766

Na -0.674203 2.294796 -0.127702
Cl 0.570860 1.066642 -2.168658
Na 1.529897 -1.292366 -1.436524
Cl -0.669545 -2.616751 -1.441941
Na -1.400010 -2.039671 1.058518
Cl -1.464136 0.604661 2.097634
Na 1.102281 0.719633 2.483223
Cl 1.873077 2.789412 1.102259
Na 2.880091 1.830128 -1.130052
Cl 4.191219 -0.507763 -1.220563
Na 4.181692 -1.579478 1.085048
Cl 1.747752 -1.749975 1.830923
Cl -3.940810 -1.693930 0.236773
Na -3.963394 0.770364 1.006180

N8l
Na -0.545324 2.242737 0.716983
Cl -1.480350 -0.002570 1.999283
Na 1.480744 -0.819546 -1.579222
Cl 0.834122 1.841341 -1.648609
Na 3.121482 1.921502 -0.285185
Cl 2.001038 2.044364 2.073974
Na 1.016887 -0.329721 2.561021
Cl 1.548900 -2.399821 0.991975
Na 4.060028 -2.195499 0.534370
Cl 4.210734 -0.334222 -1.194303
Cl -2.948510 2.429959 -0.563707
Na -1.760031 -2.174362 0.179187
Cl -4.351403 -1.977604 0.201323
Na -3.990350 0.450014 0.890255
Na -1.721344 0.901592 -2.330938
Cl -0.890003 -1.599323 -2.304123

N8m
Cl -0.964417 1.026482 1.807505
Na -0.563495 -1.522173 0.981018
Cl 1.285338 -0.279665 -1.289988
Na 0.249501 2.256537 -0.381067
Cl 2.651128 2.666588 0.704741
Na 1.738460 0.705008 2.269515
Cl 1.766057 -1.961999 2.160160
Na 3.108235 -2.149945 -0.116970
Cl 5.246136 -1.027740 -0.948335
Na 3.864743 1.107682 -1.053960
Na -3.286906 1.564311 0.477614
Cl -2.160578 -2.399635 -0.995574
Na -4.605278 -1.937672 -0.330211
Cl -5.432018 0.159617 0.751860
Cl -1.986562 2.044841 -1.830892
Na -1.131299 -0.376869 -2.401493

N8n
Cl -1.984366 -1.198360 0.000000
Na -1.420838 -3.162960 1.875637
Cl -0.579467 -4.765817 0.000000
Na -1.420838 -3.162960 -1.875637
Cl 0.806801 -2.077839 -2.678973
Na 0.238219 0.400718 -2.044997
Cl -1.420838 2.448954 -1.970494
Na -2.769724 1.323304 0.000000
Cl -1.420838 2.448954 1.970494
Na 0.238219 0.400718 2.044997
Cl 0.806801 -2.077839 2.678973
Na 1.323006 -2.617029 0.000000
Cl 2.009264 0.521083 0.000000
Na -0.196131 3.859987 0.000000
Cl 2.186471 4.878922 0.000000
Na 3.383989 2.683043 0.000000

N8o
Cl 1.337187 -0.159808 1.648250
Na 2.851277 -2.310624 0.740914
Cl 1.675651 -2.550509 -1.577974
Na -0.649473 -1.669792 -0.833048
Cl -2.255316 -2.151047 1.286010
Na -1.211232 0.058499 2.337840
Cl -2.016857 2.361521 1.380011
Na 0.237382 2.380054 -0.057145
Cl -0.759752 0.730933 -1.915618
Na -3.158677 1.505878 -0.933655
Na 3.709688 1.298834 1.268421
Cl 4.822956 -0.734664 0.122683
Na 2.587810 0.038575 -1.683607
Cl 2.737671 2.631288 -0.754574
Cl -5.348563 0.136319 -1.049244
Na -4.665014 -1.709476 0.490074

N8p
Na 3.461568 0.759705 -1.010163
Cl 4.971985 -1.344994 -0.799246
Na 2.895665 -2.163512 0.449875
Cl 1.112428 -0.886962 -1.441936
Na -0.296287 1.318816 -1.551334
Cl 1.784082 2.981902 -1.467067
Na 1.456842 2.983384 1.118331
Cl 2.755319 0.834428 1.865728
Na 0.446844 -0.333500 1.958556
Cl 0.380260 -2.925455 1.579756
Na -0.842452 -2.446083 -0.710993
Cl -2.778796 -0.715524 -1.423296
Na -3.492874 0.796659 0.880472
Cl -1.082542 1.771572 1.198804
Cl -6.013971 0.310095 0.666256
Na -5.373761 -0.954201 -1.411380

N8q
Na -0.516295 2.504296 -0.860865
Cl 1.188919 4.444674 -0.520702
Na 2.476864 2.496403 0.525285
Cl 1.610218 0.653093 -1.439338
Na -0.140081 -1.301990 -1.608681
Cl -2.206032 0.416774 -1.854629
Na -2.568928 -0.162350 1.392778
Cl -0.634235 1.617274 1.775539
Na 1.077253 -0.303676 1.902068
Cl 3.644683 0.110517 1.600136
Na 3.495604 -1.055073 -0.796732
Cl 2.085790 -3.228004 -1.519266
Na 0.960403 -4.203004 0.536657
Cl -0.621443 -2.284773 1.170071
Cl -5.112667 -0.507238 0.988694
Na -4.715636 0.136357 -1.400384

N8r
Cl -1.542006 1.266698 0.000000
Na -1.324191 3.880086 0.000000
Cl -1.732812 6.467174 0.000000
Na -1.206810 8.986201 0.000000
Cl 1.309901 9.143194 0.000000
Na 1.191961 6.555878 0.000000
Cl 1.406570 3.942890 0.000000
Na 1.206810 1.331437 0.000000
Cl 1.542006 -1.266698 0.000000
Na 1.324191 -3.880086 0.000000
Cl 1.732812 -6.467174 0.000000
Na -1.191961 -6.555878 0.000000
Cl -1.406570 -3.942890 0.000000
Na -1.206810 -1.331437 0.000000
Cl -1.309901 -9.143194 0.000000
Na 1.206810 -8.986201 0.000000

N8s
Cl -0.000000 -0.000000 0.485440
Na -0.812647 -1.673322 2.863524
Cl -0.000000 -0.000000 4.604485
Na 0.812647 1.673322 2.863524
Cl 1.517858 3.634449 1.272999
Na 0.529678 2.230371 -0.798607
Cl 0.000000 4.190670 -2.430184
Na -1.089634 6.488032 -2.835347
Cl -0.419643 7.920699 -0.872288
Na 0.759181 5.783701 -0.026236
Na -0.529678 -2.230371 -0.798607
Cl -1.517858 -3.634449 1.272999
Na -0.759181 -5.783701 -0.026236
Cl -0.000000 -4.190670 -2.430184
Na 1.089634 -6.488032 -2.835347
Cl 0.419643 -7.920699 -0.872288

6.6 Structures Neutral Clusters: ωB97XD/aug-cc-pVDZ

N1a
Na 0.000000 0.000000 -1.458648
Cl 0.000000 0.000000 0.943831

N2a
Na 0.000000 1.603431 0.000000
Cl -0.000000 -0.000000 2.014710
Na -0.000000 -1.603431 0.000000
Cl 0.000000 -0.000000 -2.014710

N3a
Na 0.000000 2.329987 -0.000000
Cl 2.373080 1.370099 0.000000
Na 2.017828 -1.164993 0.000000
Cl -2.373080 1.370099 -0.000000
Na -2.017828 -1.164993 -0.000000
Cl -0.000000 -2.740197 0.000000

N3b
Na -0.000000 1.463121 0.645222
Cl 1.837539 -0.000000 -0.924305
Na -0.000000 -1.463121 0.645222
Cl -1.837539 0.000000 -0.924305
Na 0.000000 0.000000 -2.698098
Cl -0.000000 -0.000000 2.759446

N4a
Na 1.223849 1.223849 1.223849
Cl 1.438020 -1.438020 1.438020
Na -1.223849 -1.223849 1.223849
Cl -1.438020 1.438020 1.438020
Na -1.223849 1.223849 -1.223849
Cl -1.438020 -1.438020 -1.438020
Cl 1.438020 1.438020 -1.438020
Na 1.223849 -1.223849 -1.223849

N4b
Cl 2.478677 2.478677 -0.000000
Na 0.000000 3.109557 0.000000
Cl -2.478677 2.478677 -0.000000
Na -3.109557 0.000000 0.000000
Cl -2.478677 -2.478677 -0.000000
Na -0.000000 -3.109557 0.000000
Cl 2.478677 -2.478677 -0.000000
Na 3.109557 -0.000000 0.000000

N4c
Na -0.144621 -1.830198 1.486917
Cl 0.670311 -3.766265 0.000000
Na -0.144621 -1.830198 -1.486917
Cl -2.204848 -0.828358 0.000000
Na -1.184889 1.587313 0.000000
Cl 1.308761 0.208505 0.000000
Cl -0.144621 3.974631 0.000000
Na 2.046564 2.709017 0.000000

N4d
Cl -0.082131 -1.790128 1.842819
Na 0.782587 0.263778 0.000000
Cl 2.229798 2.451978 0.000000
Na -0.082131 3.475085 0.000000
Cl -1.809821 1.545101 0.000000
Na -1.950373 -1.080802 0.000000
Cl -0.082131 -1.790128 -1.842819
Na 0.854721 -3.302241 0.000000

N4e
Na 0.000000 1.623045 0.386156
Cl 0.000000 2.306852 -2.086822
Na 0.000000 -0.000000 -3.182988
Cl -0.000000 -2.306852 -2.086822
Na -0.000000 -1.623045 0.386156
Cl -1.815918 0.000000 1.720875
Cl 1.815918 -0.000000 1.720875
Na 0.000000 0.000000 3.541785

N4g
Cl 0.000000 0.000000 -1.291512
Na -0.000000 2.458494 -2.200677
Cl -0.000000 4.032222 -0.251683
Na -0.000000 1.926360 1.289938
Cl -0.000000 -0.000000 2.973482
Na -0.000000 -1.926360 1.289938
Cl -0.000000 -4.032222 -0.251683
Na -0.000000 -2.458494 -2.200677

N5a
Na -0.794991 1.760894 1.773287
Cl 1.267452 1.433350 -0.000000
Na 1.743311 -0.896512 -1.662386
Cl -0.794991 -0.798395 -2.444940
Na -0.794991 1.760894 -1.773287
Cl -2.729343 1.844788 0.000000
Na -1.966673 -0.789758 0.000000
Cl -0.794991 -0.798395 2.444940
Na 1.743311 -0.896512 1.662386
Cl 3.097189 -2.288942 -0.000000

N5b
Na 1.150849 1.092124 1.708017
Cl -0.536924 2.375201 0.012414
Na 1.155381 1.117714 -1.697142
Cl 0.504296 -1.433830 -2.001205
Na -1.333301 -1.590314 -0.006197
Cl 0.506943 -1.464221 1.985733
Na 2.270100 -1.660986 -0.012472
Cl 3.195161 0.842300 0.006931
Cl -3.798469 -0.724829 -0.000637
Na -3.043678 1.667958 0.002793

N5c
Na 0.000000 0.000000 -1.431204
Cl 0.000000 2.865271 -0.747512
Na -1.832149 -1.832062 0.749479
Cl 0.000000 0.000000 1.980309
Na 1.832149 1.832062 0.749479
Cl 2.864033 -0.000000 -0.749520
Na 1.832149 -1.832062 0.749479
Cl -2.864033 0.000000 -0.749520
Na -1.832149 1.832062 0.749479
Cl -0.000000 -2.865271 -0.747512

N5d
Na -0.107907 -1.140604 2.644940
Cl -0.249750 -1.361911 -0.000000
Na -0.107907 -1.140604 -2.644940
Cl 0.046226 -1.415528 -5.236533
Na 0.185396 1.106160 -5.161016
Cl 0.046226 1.690940 -2.628401
Na -0.054762 1.322387 0.000000
Cl 0.046226 1.690940 2.628401
Na 0.185396 1.106160 5.161016
Cl 0.046226 -1.415528 5.236533

N5e
Na -2.966843 -0.248587 1.485914
Cl -0.559664 -0.903466 0.000422
Na 2.101351 -1.114035 0.001031
Cl 2.535341 1.683018 0.000280
Na -0.110126 1.783065 0.000477
Cl -2.705126 2.050264 -0.000694
Na -2.966105 -0.250131 -1.486608
Cl -4.548834 -1.622512 0.000188
Na 4.963093 0.719786 -0.000652
Cl 4.617397 -1.783251 -0.000301

N5f
Cl 2.519767 3.468162 -0.000000
Na -0.000000 3.918055 -0.000000
Cl -2.519767 3.468162 -0.000000
Na -3.726291 1.210745 -0.000000
Cl -4.077069 -1.324720 -0.000000
Na -2.302975 -3.169773 -0.000000
Cl -0.000000 -4.286884 -0.000000
Na 2.302975 -3.169773 -0.000000
Cl 4.077069 -1.324720 -0.000000
Na 3.726291 1.210745 -0.000000

N6a
Cl 0.000000 -0.000000 2.152625
Na 0.000000 1.858079 -0.000000
Cl -2.723206 2.045019 0.000000
Na -2.634441 0.000000 -1.698318
Cl 0.000000 -0.000000 -2.152625
Na 2.634441 -0.000000 -1.698318
Cl 2.723206 2.045019 0.000000
Na 2.634441 0.000000 1.698318
Cl 2.723206 -2.045019 0.000000
Na -0.000000 -1.858079 -0.000000
Cl -2.723206 -2.045019 -0.000000
Na -2.634441 -0.000000 1.698318

N6b
Na -0.000000 2.511611 1.226573
Cl -0.000000 2.757172 -1.472012
Na 2.175119 1.255806 -1.226573
Cl 2.387781 1.378586 1.472012
Na 2.175119 -1.255806 1.226573
Cl -0.000000 -2.757172 1.472012
Na -2.175119 -1.255806 1.226573
Cl -2.387781 1.378586 1.472012
Na -2.175119 1.255806 -1.226573
Cl -2.387781 -1.378586 -1.472012
Na -0.000000 -2.511611 -1.226573
Cl 2.387781 -1.378586 -1.472012

N6c
Na -2.084814 1.797619 0.675686
Cl -1.978122 0.029600 -1.670975
Na 0.026015 1.735063 -2.177696
Cl 0.051187 3.254830 -0.068864
Na 2.149523 1.736311 0.664445
Cl 1.977875 -0.030057 -1.671148
Na -0.026324 -1.735664 -2.177586
Cl -0.051224 -3.254792 -0.068234
Cl 3.011780 -0.041923 2.281096
Cl -3.011315 0.042471 2.281460
Na -2.149435 -1.736148 0.664992
Na 2.084756 -1.797381 0.675914

N6d
Na -1.287690 -1.580282 0.568139
Cl 0.667473 -2.798957 -0.725168
Na 2.424722 -1.823604 0.909831
Cl 0.621837 -0.001420 1.933115
Cl 3.697110 0.000406 -0.474437
Na 2.425365 1.824169 0.913233
Na 1.082328 0.000906 -1.523384
Cl 0.668670 2.799166 -0.723098
Na -1.284005 1.574470 0.568197
Cl -3.408252 -0.000206 1.479642
Na -4.157052 0.005781 -0.971753
Cl -1.731563 0.000079 -1.790459

N6e
Na -0.088455 1.411038 1.685477
Cl 1.990496 1.897649 0.000000
Na 1.813539 -1.360150 0.000000
Cl -0.088455 -1.176099 1.954119
Na -1.815687 -1.885050 0.000000
Cl -4.395471 -1.475004 0.000000
Na -4.146386 1.019900 0.000000
Cl -1.944609 2.418048 0.000000
Na -0.088455 1.411038 -1.685477
Cl -0.088455 -1.176099 -1.954119
Cl 4.444466 -1.512739 0.000000
Na 4.452212 0.986146 0.000000

N6f
Cl 0.373074 1.506822 0.000000
Na -1.574470 0.624272 1.737415
Cl -3.572019 0.929366 0.000000
Na -1.574470 0.624272 -1.737415
Cl -1.574470 -1.978900 -1.990542
Na -3.329050 -1.759348 0.000000
Cl -1.574470 -1.978900 1.990542
Na 0.257732 -2.390720 0.000000
Cl 2.818747 -2.103864 0.000000
Na 3.281440 0.404311 0.000000
Cl 4.221918 2.855622 0.000000
Na 1.868157 3.686987 0.000000

N6g
Cl 1.456597 -1.381144 -0.142159
Na 1.247933 1.260969 -0.128477
Cl 1.726618 3.872174 -0.023087
Na -1.094820 4.013492 -0.005648
Cl -1.456597 1.381144 -0.142159
Na -1.247933 -1.260969 -0.128477
Cl -1.726618 -3.872174 -0.023087
Na -1.273738 -6.428573 0.142526
Cl 1.247933 -6.615563 0.159810
Na 1.094820 -4.013492 -0.005648
Cl -1.247933 6.615563 0.159810
Na 1.273738 6.428573 0.142526

N6h
Cl 1.428889 3.912802 -0.674009
Na 2.860247 1.903553 0.100399
Cl 4.476790 0.000181 0.675462
Na 2.860406 -1.903253 0.100169

Cl 1.429299 -3.912742 -0.674113
Na -0.697197 -2.532937 -0.621633
Cl -3.179138 -2.000064 0.323176
Na -4.501951 -0.000131 -0.650341
Cl -3.179272 1.999912 0.323044
Na -0.697404 2.532640 -0.622047
Cl 0.350645 -0.000024 0.105465
Na -1.875248 0.000029 1.571322

N6j
Cl -0.000000 0.000000 -0.031896
Na 0.106869 2.373330 -1.311585
Cl 0.900390 4.351610 -2.799342
Na 0.000000 5.702009 -0.853027
Cl -0.927206 3.870390 0.757742
Na -0.688330 1.655225 2.215672
Cl 0.927206 -3.870390 0.757742
Na -0.000000 -5.702009 -0.853027
Cl -0.900390 -4.351610 -2.799342
Na -0.106869 -2.373330 -1.311585
Na 0.688330 -1.655225 2.215672
Cl 0.000000 -0.000000 4.049020

N6k
Cl -2.141461 -0.384721 0.000000
Na -0.437298 -2.433130 -0.000000
Cl 0.724738 -4.710804 -0.000000
Na 3.085372 -3.713579 -0.000000
Cl 1.903461 -0.699574 -0.000000
Na 0.000000 1.181138 0.000000
Cl -1.156983 3.550010 0.000000
Na -3.104457 5.287817 0.000000
Cl -5.202339 3.880045 0.000000
Na -3.507559 1.903791 0.000000
Na 4.516732 -0.469618 -0.000000
Cl 5.514897 -2.771462 -0.000000

N6l
Na -4.242320 -0.253432 1.477805
Cl -5.796003 -1.644959 -0.022930
Na -4.221586 -0.246627 -1.495409
Cl -3.979716 2.043686 -0.003182
Na -1.379709 1.805556 0.011363
Cl 1.256698 1.656377 0.020658
Na 3.843644 1.000526 0.008511
Cl 3.371861 -1.779556 0.027368
Na 0.834242 -1.020200 0.023045
Cl -1.821856 -0.903121 0.009044
Cl 6.444683 0.851770 -0.034503
Na 5.976060 -1.632307 -0.019836

N6n
Cl -1.464784 1.810424 -0.003002
Na -4.047369 2.072195 0.000594
Cl -6.103316 0.580012 0.003770
Na -5.100064 -1.781491 0.001657
Cl -2.732645 -2.735140 -0.002258
Na -0.968817 -0.848264 -0.003953
Cl 1.708954 -0.615477 -0.001845
Na 1.213739 2.150451 -0.000180
Cl 3.613325 3.023821 0.001150
Na 4.561608 0.624562 -0.000620
Na 3.313178 -2.699686 0.002335
Cl 5.643465 -1.751608 0.002294

N6o
Cl 2.536468 4.393291 0.000000
Na -0.000000 4.726794 0.000000
Cl -2.536468 4.393291 -0.000000
Na -4.093523 2.363397 0.000000
Cl -5.072935 0.000000 0.000000
Na -4.093523 -2.363397 0.000000
Cl -2.536468 -4.393291 0.000000
Na -0.000000 -4.726794 0.000000
Cl 2.536468 -4.393291 -0.000000
Na 4.093523 -2.363397 0.000000
Cl 5.072935 -0.000000 0.000000
Na 4.093523 2.363397 0.000000

N7a
Na -2.024234 1.168692 -1.327691
Cl -2.015187 -1.163469 -2.646915
Na -0.000000 -2.337384 -1.327691
Cl 0.000000 -2.637404 1.289919
Na -1.726618 -0.996864 2.524239
Cl -2.284059 1.318702 1.289919
Na 0.000000 1.993727 2.524239
Cl 0.000000 0.000000 4.299395
Na 1.726618 -0.996864 2.524239
Cl 2.015187 -1.163469 -2.646915
Na 0.000000 0.000000 -3.942637
Cl 0.000000 2.326937 -2.646915
Na 2.024234 1.168692 -1.327691
Cl 2.284059 1.318702 1.289919

N7b
Cl -2.242743 -1.521719 -0.000000
Na -0.680108 -3.741655 -0.000000
Cl 0.921948 -3.099067 2.032943
Na -0.462631 -0.805204 1.979457
Cl -1.381707 1.660547 2.466085
Na -2.333766 1.237135 0.000000
Cl -1.381707 1.660547 -2.466085
Na 0.921948 2.735512 -1.715753
Cl 1.316918 0.417713 0.000000
Na 2.316353 -2.106015 -0.000000
Cl 0.921948 -3.099067 -2.032943
Na -0.462631 -0.805204 -1.979457
Na 0.921948 2.735512 1.715753
Cl 1.702269 4.466287 0.000000

N7c
Cl 1.618357 0.062648 -0.000000
Na 1.739271 2.235382 1.877774
Cl -0.670553 1.822310 2.749019
Na -0.670553 -0.697362 2.083345
Cl 0.039278 -3.268826 2.027933
Na 1.630787 -2.666386 0.000000
Cl 0.039278 -3.268826 -2.027933
Na -1.711580 -3.286666 0.000000
Cl -2.373355 -0.673570 0.000000
Na -1.039362 2.169272 -0.000000
Cl 1.359305 3.961905 -0.000000
Na 1.739271 2.235382 -1.877774
Cl -0.670553 1.822310 -2.749019
Na -0.670553 -0.697362 -2.083345

N7d
Cl 0.318050 0.179302 1.974933
Na 0.318050 -2.493520 1.672652
Cl 2.364034 -2.769365 0.000000
Na 2.331229 -0.110995 0.000000
Cl 3.207107 2.397652 0.000000
Na 1.182134 2.753505 1.706471
Cl -0.602002 3.673121 0.000000
Na 1.182134 2.753505 -1.706471
Cl 0.318050 0.179302 -1.974933
Na 0.318050 -2.493520 -1.672652
Cl -1.530192 -3.538298 0.000000
Na -1.590269 1.149983 0.000000
Cl -4.073071 0.285349 0.000000
Na -3.744382 -2.188056 0.000000

N7e
Na 1.020869 -3.102760 0.000000
Cl -0.635528 -3.104920 2.046019
Na -2.355095 -3.041089 0.000000
Cl -0.635528 -3.104920 -2.046019
Na -0.657694 -0.369286 -1.860483
Cl -2.785181 -0.423519 0.000000
Na -0.657694 -0.369286 1.860483
Cl 1.629371 -0.478824 0.000000
Na 3.962400 0.761722 0.000000
Cl 3.697025 3.235737 0.000000
Na 1.101694 2.766521 0.000000
Cl -0.657694 2.303450 -2.026711
Na -2.344580 2.225296 0.000000
Cl -0.657694 2.303450 2.026711

N7h
Cl 1.546019 -2.360033 0.000000
Na 0.797471 -1.630616 2.511474
Cl -1.829394 -2.051720 2.401549
Na -1.211504 -2.898921 0.000000
Cl -1.829394 -2.051720 -2.401549
Na 0.797471 -1.630616 -2.511474
Cl 0.797471 0.953771 -2.223055
Na -1.836223 0.628890 -2.005889
Cl -2.281949 2.283666 -0.000000
Na -1.836223 0.628890 2.005889
Cl 0.797471 0.953771 2.223055
Na 2.479721 0.614028 -0.000000
Cl 3.040472 3.149488 -0.000000
Na 0.437302 2.932635 -0.000000

N7i
Na 1.998664 -1.687076 0.000000
Cl 1.605567 -1.132009 2.620127
Na -0.789773 -2.206020 2.254677
Cl -0.232810 -3.439519 0.000000
Na -0.789773 -2.206020 -2.254677
Cl 1.605567 -1.132009 -2.620127
Na 0.329987 1.081620 -1.853154
Cl -2.167357 0.097143 -2.279645
Na -2.841018 1.194378 -0.000000
Cl -2.167357 0.097143 2.279645
Na 0.329987 1.081620 1.853154
Cl 2.382229 1.562474 -0.000000
Na 1.556638 3.976286 -0.000000
Cl -0.893005 3.147797 -0.000000

N7j
Na -1.192981 -2.772315 -1.054019
Cl -3.287301 -1.041197 -1.388848
Na -0.473870 -0.524060 1.339271
Cl -2.248841 1.586667 1.283447
Na 0.213913 2.890787 1.315433
Cl 1.712916 0.812110 1.918762
Na 2.500699 1.137497 -0.880428
Cl 0.527453 3.106218 -1.267829
Na -1.350314 1.176210 -1.526713
Cl -0.073301 -3.135035 1.327186
Na 2.374231 -1.801941 0.580595
Cl 0.473670 -0.754476 -1.419798
Cl 4.336240 -0.725653 -0.721791
Na -4.298423 0.127751 0.641388

N7k
Na 0.000000 0.000000 -0.675861
Cl 2.110922 1.218742 -2.137976
Na -0.000000 2.797331 -1.897950
Cl -0.000000 2.541506 0.847377
Na 1.803773 1.041409 2.136680
Cl 2.201009 -1.270753 0.847377
Na 2.422560 -1.398665 -1.897950
Cl -0.000000 -2.437483 -2.137976

Na -2.422560 -1.398665 -1.897950
Cl -2.110922 1.218742 -2.137976
Cl -2.201009 -1.270753 0.847377
Na -0.000000 -2.082817 2.136680
Cl 0.000000 0.000000 3.845701
Na -1.803773 1.041409 2.136680

N7l
Cl -0.000000 0.000000 1.614182
Na 0.000000 2.629046 1.287683
Cl 0.000000 5.259654 1.649350
Na 0.000000 5.281159 -1.178884
Cl 0.000000 2.631426 -1.420128
Na 0.000000 0.000000 -1.120307
Cl -0.000000 -2.631426 -1.420128
Na -0.000000 -5.281159 -1.178884
Cl 0.000000 -7.878502 -1.443925
Na 0.000000 -7.800554 1.081300
Cl -0.000000 -5.259654 1.649350
Na -0.000000 -2.629046 1.287683
Cl 0.000000 7.878502 -1.443925
Na 0.000000 7.800554 1.081300

N7m
Cl 1.179095 0.322931 0.001271
Na 3.131129 -1.528128 0.001271
Cl 4.480699 -3.747902 -0.001727
Na 6.429779 -2.124241 -0.002228
Cl 5.254859 0.208953 0.001170
Na -0.098289 2.962527 -0.000487
Cl -2.547436 1.936997 0.001966
Na -4.835841 0.557539 0.000675
Cl -7.267537 -0.378031 -0.003524
Na -6.076393 -2.607889 -0.001329
Cl -3.546990 -1.975795 0.002706
Na -1.388455 -0.462753 0.002728
Na 3.415181 2.141409 -0.000239
Cl 2.073885 4.319723 -0.002114

N7n
Cl 5.359661 0.000000 3.297605
Na 4.250012 0.000000 0.993255
Cl 2.698841 0.000000 -1.039782
Na 0.203632 0.000000 -1.604570
Cl -2.352598 0.000000 -1.660456
Na -4.352501 0.000000 -0.066537
Cl -5.992478 0.000000 1.896237
Na -5.993128 0.000000 4.453476
Cl -5.474937 0.000000 6.957575
Na -3.474376 0.000000 8.550629
Cl -1.196413 0.000000 9.713724
Na 1.296015 0.000000 9.142480
Cl 3.623594 0.000000 8.082280
Na 4.731644 0.000000 5.777164

N8a
Cl 0.000000 2.121293 1.317716
Na 0.000000 1.835879 -1.390608
Cl -2.121293 0.000000 -1.317716
Na -1.835879 0.000000 1.390608
Cl -2.036487 0.000000 4.094969
Na 0.000000 1.710953 3.966804
Cl 2.036487 0.000000 4.094969
Na -0.000000 -1.710953 3.966804
Cl -0.000000 -2.121293 1.317716
Na -0.000000 -1.835879 -1.390608
Cl 2.121293 -0.000000 -1.317716
Na 1.835879 -0.000000 1.390608
Cl -0.000000 -2.036487 -4.094969
Na 1.710953 -0.000000 -3.966804
Cl 0.000000 2.036487 -4.094969
Na -1.710953 0.000000 -3.966804

N8b
Na 1.617072 -0.989107 1.897277
Cl 0.039165 -1.474937 4.042932
Na -0.637145 1.105145 3.811522
Cl -0.452202 -1.652288 -0.000000
Na 1.617072 -0.989107 -1.897277
Cl 3.531499 -0.860388 0.000000
Na 2.931888 1.709657 0.000000
Cl 1.148708 1.787242 1.957897
Na -1.047070 1.281747 -0.000000
Cl -2.726138 0.919077 -2.214781
Na -1.924474 -1.573171 -2.273604
Cl 0.039165 -1.474937 -4.042932
Na -0.637145 1.105145 -3.811522
Cl 1.148708 1.787242 -1.957897
Cl -2.726138 0.919077 2.214781
Na -1.924474 -1.573171 2.273604

N8d
Cl -1.013460 3.141453 2.043532
Na -1.936413 1.574519 0.000000
Cl -1.013460 3.141453 -2.043532
Na 0.366347 4.026054 0.000000
Cl 1.992927 1.857293 0.000000
Na 3.086433 -0.569513 -0.000000
Cl 2.215598 -1.246885 2.417653
Na 0.588151 0.987288 2.291118
Cl -1.763573 -0.625458 1.941798
Na -2.707293 -2.152046 -0.000000
Cl -1.763573 -0.625458 -1.941798
Na 0.588151 0.987288 -2.291118
Cl 2.215598 -1.246885 -2.417653
Na 0.042753 -2.646094 2.017093
Na 0.042753 -2.646094 -2.017093
Cl -0.915921 -4.111716 -0.000000

N8e
Cl -1.476690 -2.306451 -2.139411
Na -2.367873 -2.260692 0.319085
Cl -3.739414 -0.512580 1.802908
Na -1.219880 0.238139 -2.864342
Cl 1.383472 0.398900 -2.333045
Na 1.054309 -2.091405 -1.040651
Cl -0.000365 -2.133527 1.631475
Na -1.424985 -0.463557 3.104876
Cl -0.327507 1.706766 2.067687
Na -2.705409 1.725987 0.772236
Cl -1.885043 2.477294 -1.595298
Na 0.665772 2.446985 -0.525832
Cl 3.143699 2.289350 0.526241
Na 2.003265 0.012802 1.531703
Na 3.861852 0.483710 -1.231178
Cl 3.489139 -1.757712 0.067910

N8f
Na 2.002788 1.512130 0.041997
Cl 4.351928 0.099087 0.010311
Na 2.947376 -1.341423 1.700234
Cl 1.064991 0.349614 2.490867
Na -0.928138 2.165524 2.141553
Cl 0.127076 3.466234 0.087488
Na -3.877258 -0.512711 -0.016041
Cl -2.491973 -2.755378 -0.082798
Na -0.908697 -1.515536 1.690306
Cl -2.847153 0.401600 2.255463
Cl 1.079263 0.477336 -2.471799
Na -0.898868 -1.410742 -1.763744
Cl -2.834609 0.537899 -2.226797
Na -0.910847 2.289443 -2.045856
Na 2.950324 -1.256901 -1.754845
Cl 1.306743 -2.530958 -0.058598

N8g
Cl 0.000000 -0.000000 0.972325
Na 0.000000 1.847659 3.150629
Cl 0.000000 -0.000000 4.943452
Na -0.000000 -1.847659 3.150629
Cl -0.000000 -3.603895 1.149310
Na -1.624281 -1.973314 -0.291552
Cl -0.000000 -1.979909 -2.661003
Na 1.750515 -0.000000 -3.142377
Cl 3.324223 -0.000000 -1.074232
Na 1.624281 -1.973314 -0.291552
Na -1.624281 1.973314 -0.291552
Cl 0.000000 1.979909 -2.661003
Na -1.750515 0.000000 -3.142377
Cl -3.324223 0.000000 -1.074232
Cl 0.000000 3.603895 1.149310
Na 1.624281 1.973314 -0.291552

N8h
Cl -0.249930 0.723020 0.000000
Na -2.405519 1.231352 1.710275
Cl -2.850934 3.233751 0.000000
Na -2.405519 1.231352 -1.710275
Cl -2.405519 -1.286461 -2.402673
Na 0.309264 -1.336724 -1.982462
Cl 2.886664 -0.785941 -2.030856
Na 2.747266 1.110243 0.000000
Cl 2.886664 -0.785941 2.030856
Na 0.309264 -1.336724 1.982462
Cl -2.405519 -1.286461 2.402673
Na -2.248574 -2.466432 0.000000
Cl 0.331751 -3.266554 0.000000
Na 2.969448 -2.455138 0.000000
Na -0.149789 3.604343 0.000000
Cl 2.372457 3.724880 0.000000

N8j
Na -3.427316 1.889956 0.000000
Cl -3.731289 0.195566 2.010805
Na -3.919373 -1.564407 0.000000
Cl -3.731289 0.195566 -2.010805
Na -1.118724 -0.387757 -1.811285
Cl 1.506188 -0.394569 -2.108325
Na 1.506188 2.175616 -1.691013
Cl -0.718649 2.105706 0.000000
Na 1.506188 2.175616 1.691013
Cl 1.506188 -0.394569 2.108325
Na -1.118724 -0.387757 1.811285
Cl -1.363511 -2.405887 0.000000
Na 1.450965 -2.442187 0.000000
Cl 3.450851 2.595516 0.000000
Na 3.671674 -0.171263 0.000000
Cl 4.019180 -2.730624 0.000000

N8l
Na -0.803580 2.340364 -0.208570
Cl -1.470367 0.733210 2.063317
Na 1.597943 -1.424767 -1.207438
Cl 0.408410 0.809375 -2.101307
Na 2.719342 1.818907 -1.280952
Cl 1.735134 3.031698 0.855726
Na 1.110722 0.976072 2.353699
Cl 1.771865 -1.559184 1.819008
Na 4.263001 -1.508524 1.142521
Cl 4.201066 -0.440058 -1.182704
Cl -3.217741 1.909485 -1.271239
Na -1.273547 -1.991117 1.175415
Cl -3.815036 -1.792706 0.314961
Na -3.962028 0.706634 0.927476
Na -2.206167 -0.671772 -1.731880
Cl -0.548775 -2.850864 -1.254996

N8m
Cl -0.960346 0.873677 1.852868
Na -0.364528 -1.526331 0.736625
Cl 1.282835 -0.290957 -1.305237
Na 0.231345 2.193404 -0.241657
Cl 2.625174 2.620375 0.873573
Na 1.726904 0.598050 2.383073
Cl 1.870846 -2.063786 2.098164
Na 3.252957 -2.116200 -0.161126
Cl 5.309161 -0.903945 -1.083844
Na 3.809422 1.163857 -0.993240
Na -3.276762 1.383349 0.449131
Cl -2.276702 -2.220743 -1.057745
Na -4.745951 -1.986475 -0.235170
Cl -5.558041 0.174350 0.753763
Cl -1.954080 2.156648 -1.779504
Na -1.157058 -0.243794 -2.481692

N8n
Cl -1.908668 -1.088077 -0.000000
Na -1.425954 -3.183145 1.887408
Cl -0.587981 -4.748861 0.000000
Na -1.425954 -3.183145 -1.887408
Cl 0.778345 -2.095586 -2.729080
Na 0.027168 0.319772 -2.013518
Cl -1.425954 2.524787 -2.002986
Na -2.779316 1.508248 -0.000000
Cl -1.425954 2.524787 2.002986
Na 0.027168 0.319772 2.013518
Cl 0.778345 -2.095586 2.729080
Na 1.217702 -2.470456 0.000000
Cl 1.849528 0.542719 0.000000
Na 0.051762 3.661007 -0.000000
Cl 2.427394 4.762427 0.000000
Na 3.557795 2.523188 0.000000

N8o
Cl 1.283711 -0.135349 1.577533
Na 2.967293 -2.327465 0.747306
Cl 1.687535 -2.683175 -1.479813
Na -0.535369 -1.685827 -0.558250
Cl -2.385130 -2.066762 1.394024
Na -1.289067 0.141990 2.406575
Cl -2.046232 2.408233 1.343234
Na 0.274126 2.285194 0.015916
Cl -0.620425 0.616694 -1.888563
Na -3.056875 1.437458 -0.994208
Na 3.724848 1.345828 1.194452
Cl 4.829373 -0.680566 0.048439
Na 2.431449 0.010140 -1.602945
Cl 2.723780 2.675223 -0.794892
Cl -5.315370 0.167620 -1.229271
Na -4.759416 -1.673918 0.381907

N8p
Na 3.343614 0.958627 -1.046556
Cl 5.022555 -1.046230 -0.981694
Na 3.137857 -1.892285 0.572500
Cl 1.109167 -0.793962 -1.326465
Na -0.494232 1.307548 -1.410247
Cl 1.538652 3.069602 -1.407609
Na 1.314990 2.940959 1.197987
Cl 2.736007 0.799011 1.807558
Na 0.419683 -0.429010 1.537115
Cl 0.715794 -3.063693 1.550720
Na -0.558466 -2.725206 -0.732042
Cl -2.662130 -0.942114 -1.140709
Na -3.626833 0.705429 0.982663
Cl -1.198160 1.754769 1.286608
Cl -6.131292 0.354122 0.442525
Na -5.283893 -1.069296 -1.458316

N8q
Na -0.535737 2.468826 -0.931514
Cl 1.143784 4.447685 -0.655987
Na 2.223996 2.567059 0.763840
Cl 1.537856 0.605159 -1.354783
Na -0.092487 -1.465145 -1.594176
Cl -2.251437 0.279653 -1.732196
Na -2.520373 -0.109587 1.226000
Cl -0.527785 1.649244 1.743609
Na 1.093109 -0.365271 1.419734
Cl 3.654707 0.244462 1.635987
Na 3.652125 -0.870596 -0.780602
Cl 2.262925 -3.149114 -1.351431
Na 1.038176 -4.237756 0.613917
Cl -0.709252 -2.368585 1.023386
Cl -5.130968 -0.432766 1.099292
Na -4.827637 0.040875 -1.347556

N8r
Cl -9.222112 1.319629 0.145469
Na -9.090332 -1.204851 0.128716
Cl -6.540248 -1.715513 0.011814
Na -6.622071 1.108060 0.028321
Cl -3.981825 1.414783 -0.062727
Na -3.918360 -1.289686 -0.060812
Cl -1.279887 -1.552989 -0.090725
Na -1.343204 1.168872 -0.085423
Cl 1.280074 1.552650 -0.094513
Na 1.343519 -1.169288 -0.092368
Cl 3.982292 -1.414866 -0.077025
Na 3.918575 1.289473 -0.070133
Cl 6.540294 1.715609 0.005350
Na 6.622243 -1.107844 0.021782
Cl 9.221374 -1.319297 0.156113
Na 9.089688 1.205254 0.139569

N8s
Cl 0.000000 0.000000 0.874945
Na -0.000168 -1.814604 3.185406
Cl 0.000000 0.000000 4.989570
Na 0.000168 1.814604 3.185406
Cl 0.000145 3.990921 1.661062
Na 0.001382 2.345900 -0.427349
Cl 0.000726 4.122882 -2.379190
Na 0.000347 6.463671 -3.528292
Cl -0.000000 8.102615 -1.606062
Na 0.000326 5.930789 -0.169505
Na -0.001382 -2.345900 -0.427349
Cl -0.000145 -3.990921 1.661062
Na -0.000326 -5.930789 -0.169505
Cl -0.000726 -4.122882 -2.379190
Na -0.000347 -6.463671 -3.528292
Cl -0.000000 -8.102615 -1.606062
